# Revisiting symbolic addition: a step-by-step introduction to manual direct methods

**DOI:** 10.1107/S2056989026003300

**Published:** 2026-04-10

**Authors:** Thomas E. Weirich

**Affiliations:** a RWTH Aachen University, Gemeinschaftslabor für Elektronenmikroskopie (GFE), Ahornstr. 55, D-52074 Aachen, Germany; Harvard University, USA

**Keywords:** direct methods, symbolic addition, triplet relationships, Sayre equation

## Abstract

Since the mid-1980s, direct methods have become the standard approach for solving crystal structures. However, because the software is now highly automated, the underlying concepts are often less transparent to beginners. This paper therefore revisits the symbolic addition method as a pedagogical framework for understanding phase determination in crystallography. The method is introduced by providing a brief explanation of triplet phase relationships derived from Sayre’s equation, as well as their probabilistic interpretation within the formalism of direct methods. This is followed by three fully worked, two-dimensional examples of increasing complexity, which illustrate the method’s underlying principles. Starting from subsets of strong, normalized structure factors, the examples describe the complete symbolic addition procedure, including fixing the origin, propagating the phase algebraically through triplet relationships and evaluating trial phase sets using Fourier synthesis. The shown examples provide a transparent, step-by-step demonstration of how phase relationships lead from diffraction intensities to interpretable Fourier maps. Thus, this method and the provided materials can be used as an effective teaching tool to show the principles behind modern, direct methods for determining the atomic structure of crystals.

## Abstract 2

The conceptual basis of modern direct methods software, used in crystal structure determination, is demonstrated using the manual symbolic addition method with three fully worked two-dimensional examples.

## Introduction

In view of the large number of scientific articles, monographs and textbooks that have been written and published on methods for solving the phase problem in crystallography, it seems redundant to add a teaching-focused article on this topic. Such an attempt feels also unnecessary in light of the fact that the phase problem, which kept crystallographers busy for almost four decades, from the discovery of X-ray diffraction in 1912 until the early 1960s, is no longer an issue for the structural investigation of most organic and inorganic materials that form large enough and well diffracting crystals. Thus, more than 60 years later, only two of the leading figures from that time are likely to be remembered from text books on this matter: Herbert A. Hauptman and Jerome Karle, who jointly received the Nobel Prize for Chemistry in 1985 *‘for their outstanding achievements in the development of direct methods for the determination of crystal structures’*. Today, these so-called ‘direct methods’ are used thousands of times a year to determine crystal structures from X-ray diffraction data (Hauptman, 1991 ▸). and, more recently, also from electron diffraction (Ito *et al.*, 2021 ▸). The two most well known and advanced programs that every crystallographer knows by name, and which use very advanced versions of this direct methods approach are *SHELX* (Sheldrick, 2008 ▸) and *SIR* (Burla *et al.*, 2015 ▸). In contrast, the first of all direct methods programs, which paved the way to modern high-throughput X-ray crystallography, the program *MULTAN* (MULtiple TANgent formula method; Germain & Woolfson, 1968 ▸; Germain *et al.*, 1970 ▸), is not known to many. However, those new to the principles of direct methods as developed by Hauptman & Karle likely find it difficult to gain a full understanding of the theory and its implications, as the authors treated the phase problem as a multivariate statistical issue (Karle & Hauptman, 1950 ▸, 1957 ▸; Hauptman & Karle, 1952 ▸, 1954 ▸, 1957 ▸). It is sometimes read in literature that this was also the main reason why their approach was largely ignored at the beginning because understanding their probabilistic approach required a level of mathematical training that was rare among crystallographers at that time (Schechter, 1985 ▸). Nonetheless, during this early period of the development of direct methods, a significant amount of experience in the field of structural analysis was gained, particularly by the way structures were solved by Isabella and Jerome Karle. The most significant outcome of their investigations was a method known as ‘symbolic addition’. Using this method, Isabella Karle was able to demonstrate experimentally for the first time that the approach for solving crystal structures that had been developed by her husband, Jerome Karle, and Herbert Hauptman, was practicable (Karle & Karle, 1963 ▸; Massa, 2018 ▸; Schlick, 2021 ▸).

This method employs a systematic procedure involving symbols assigned to the unknown structure factor phases and an addition formula to deduce the structure factor phases. It was initially used for the analysis of centrosymmetric structures and could later applied to non-centrosymmetric structures as well (Karle & Karle, 1963 ▸, 1964 ▸, 1966 ▸). The interest in this predominantly manual approach dropped, however, when the first programs for automated symbolic addition methods for structure solution, such as LSAM (Logical Symbolic Addition Method; see Woolfson, 2014 ▸), or those by other authors (Schenk, 1971*a* ▸,*b* ▸,*c* ▸), became available in the 1970s. The introduction of *MULTAN* (Germain & Woolfson, 1968 ▸; Germain *et al.*, 1970 ▸) is generally considered as the final breakthrough, as it made determining the initial structural model of a structure a handy routine procedure (Hauptman, 1991 ▸). The latter program only required a limited number of parameters as input, such as the unit cell, the space group, the cell contents and intensities. Then, the user just had to wait until the program had found a reasonable trial model for the structure under investigation. Similarly to modern direct methods software, *MULTAN* first assigned phases to fix the unit-cell origin, then selected a few of the strongest reflections, and assigned them random phases. During the subsequent phase extension procedure, the program generated new phases for additional reflections using the weighted tangent formula (Germain *et al.*, 1971 ▸). The sets of phases thereby obtained were subsequently ranked by some figures of merit, and the most promising phase set was used to calculate a preliminary Fourier map. If these maps showed a distribution of maxima that could be interpreted in terms of a meaningful structure, the trial model was accepted and passed to the next step, the structure refinement. A summary of the historical background that led to this direct methods software can be found in several articles by M. M. Woolfson (1971 ▸, 1983 ▸, 1987 ▸, 2014 ▸). Hence, it cannot be denied that the field of crystal structure determination has undergone a radical transformation by the introduction of automated direct methods software. What was once the fine art of crystallography, carried out by a relatively small group of experts, became something that any scientist could do after some training (Hauptman, 1991 ▸; Cranswick, 2008 ▸). In subsequent years, the extreme efficiency of software designed for solving crystal structures, together with a growing level of automation in measuring diffraction data (*e.g.* Light, 2004 ▸; Rigaku Corporation, 2016 ▸), has led to a situation where ‘one does not need to know much about crystallography to solve a routine structure using modern software’ (Berry & Guzei, 2026 ▸). Hence, today’s users no longer need to bother much about the theory behind the software, which extracts the structural information for them from the measured diffraction data. Instead, users learn to follow well-established, predefined protocols, which simplify the workflow and often provide nearly instant results for both beginners and more experienced users alike. However, as Jerome Karle pointed out in his Nobel lecture in 1985, this approach has the intrinsic limitation that a crystallographic problem can only be solved if it falls within the capabilities of the software being used (see Karle, 1985 ▸, p. 233). If a structural problem falls outside the abilities of the software, it is necessary to have a good understanding of the method used and its limitations in order to identify the problem and find a workaround. In other words, crystallography training should not only cover how to operate advanced X-ray diffractometers, but also the fundamental concepts underlying the applied protocols (Irmer, 2025 ▸; Zheng *et al.*, 2025 ▸; Wheeler, 2026 ▸). This may raise the question for both beginners and teachers alike of how deeply one needs to get into the theory behind direct methods. The answer to this question can be found in the various teaching texts on this subject, which all avoid the statistical derivation developed by Hauptman and Karle, and instead try to present the key concepts necessary for the practical application as simply as possible. (*e.g.* Buerger, 1970 ▸; Schenk, 1984 ▸; Woolfson, 1997 ▸; Stout & Jensen, 1989 ▸; Glusker & Trueblood, 2010 ▸; Ladd & Palmer, 2013 ▸; Massa, 2004 ▸; Zou *et al.*, 2011 ▸).

However, to help beginners in X-ray crystallography to understand how their direct methods software works, it might be helpful to take a step back in automation and describe the principles by the manual symbolic addition method. Although many textbooks cover the key phase relationships of direct methods (*e.g.* Woolfson, 1997 ▸; Stout & Jensen, 1989 ▸; Glusker & Trueblood, 2010 ▸; Ladd & Palmer, 2013 ▸), they usually just illustrate symbolic addition and do not show the whole process from diffraction data to an interpretable Fourier map. So, students may learn about the triplet relationships without seeing how these propagate through a fully developed example. The present article attempts to close this gap by providing three start-to-finish examples of increasing complexity, which can be used in courses on X-ray crystallography or structural chemistry for teaching the conceptual ideas of direct methods. The given examples are all restricted to centrosymmetric, two-dimensional cases in order to allow an easy tracking of the algebraic logic of the phase determination via triplet relationships.

## Triplet relationships

In 1952, three articles by D. Sayre (1952 ▸), W. Cochran (1952 ▸) and W. H. Zachariasen (1952 ▸) were published in the same edition of the journal *Acta Crystallographica*. All of these articles showed that the probability of the phase sum of three related structure factors being zero increased with the magnitudes of the three structure factors (Woolfson, 2014 ▸). Among these, David Sayre’s article most clearly outlined the underlying principles of what is now understood as the core concept behind direct methods. He proved that, for well-resolved atom peaks, the structure factor phases of a structure consisting of just one atom type remain unchanged when the electron density is squared. This led Sayre to the conclusion that the structure factor phases are predominantly determined by the positions of the atoms and not by their actual shape. This statement can be understood by assuming a one-dimensional structure consisting of two identical atoms at positions *x*_1_ = 0.25 and *x*_2_ = 0.75, and an at-random atom scattering factor (*e.g.*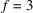). Using the standard structure factor formula (Buerger, 1970 ▸) with Laue index *h* = 2, one obtains for the real and imaginary part:
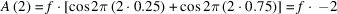

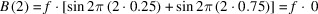

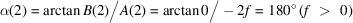
The subsequent calculation for the structure factor phase shows that the phase angle 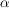 is always 180°, regardless of whether the atom scattering factor is taken as 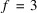 or its squared value, 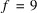. It should be noted, however, that this holds strictly only under the aforementioned restriction, that the two atoms have the same atom scattering factors. The above result from conventional structure factor calculation is also obtained in a more visual way using graphical Fourier coefficient analysis, as described in (Weirich, 2026*a* ▸).

Interestingly, Sayre’s statement also appears to apply when the square root of the electron density or the electrostatic potential is taken. The latter occurs in electron diffraction, where the intensities 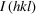 are often found to be proportional to 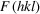 due to dynamical and secondary diffraction (Weirich, 2003 ▸). This probably laid the grounds for automated direct methods for solving several metal-rich structures, including the relatively large Ti_45_Se_16_ structure, using selected area electron diffraction data (Weirich *et al.*, 2000 ▸, 2001 ▸).

**Figure 1:**
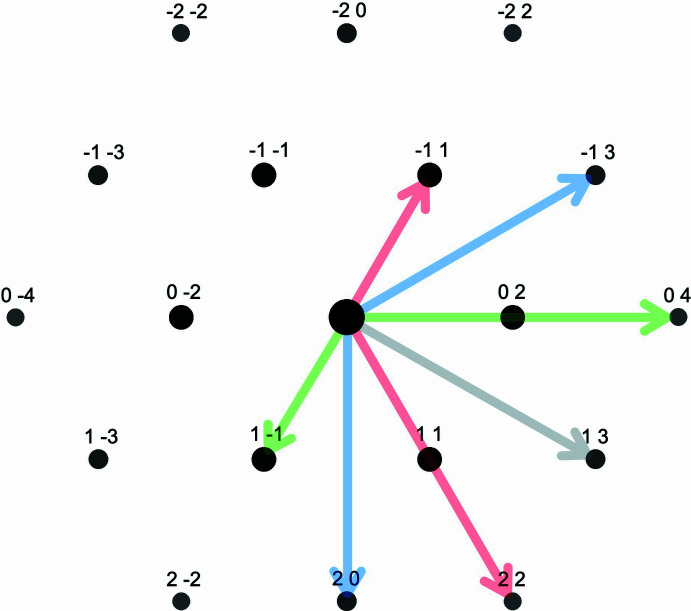
Calculated diffraction pattern for the two-dimensional structure in Fig. 2 ▸. The shown vectors correspond to the first three pairs of convoluted structure factors in eq. (2). Note that the addition of each pair of vectors (−1,1) + (2,2), (−1,3) + (2,0), and (0,4) + (1,−1) yields the (same) vector that points to diffraction spot (1,3). The latter corresponds to the left side in eq. (2).

Moreover, Sayre also proposed a formula for calculating a given structure factor from the sum of convoluted pairs of other structure factors whose indices are related to the target structure factor. For a two-dimensional structure, Sayre’s equation can be written as shown in equation (1).
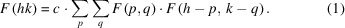
Thus, Sayre’s equation may look for the example of a structure factor with indices (1,3) as follows if the scaling factor *c* is disregarded.
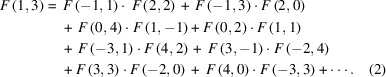
As shown in equation 2, the structure factor *F*(1,3) can be calculated from the sum of the convolution of other structure factors, if the indices of each product agrees with equation 3,
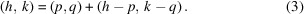
Fig. 1 ▸ provides a graphical interpretation of the latter equation using the diffraction pattern of a hypothetical two-dimensional structure. Here, the indices of each pair of structure factors (*p, q*) and (*h* − *p*, *k* − *q*) represent two vectors in the pattern. As seen, each pair yields the same endpoint (*h, k*), *i.e.* the diffraction spot that corresponds to the structure factor on the left-hand side of equations 1 and 2, respectively.

Sayre further argued that it could be imagined that pairs of these convoluted structure factors become so strong that they dominate the entire summation and thus determine the result almost alone. In this case, all other terms except the strongest pair of structure factors could be ignored, which leads to the following formula for a group of three related structure factors, which is therefore termed a triplet relationship:
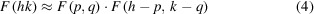
Recalling that each structure factor is a complex quantity, composed of a structure factor amplitude |*F*(*hk*)| and a structure factor phase α(*hk*) (see for example Buerger, 1970 ▸; Glusker & Trueblood, 2010 ▸; Ladd & Palmer, 2013 ▸), one can write for each part:
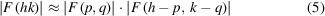

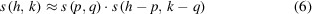
In this equation *s* denotes the signs of the structure factors. In the case of a centrosymmetric structure (or a centrosymmetric projection of a structure), where phases can only flip signs by 180° between *s* being positive (*s* = + = 0°) and *s* negative (*s* = − = 180°), formula (6) becomes stricter and the ‘approximately’ sign can be replaced by an ‘equals’ sign:
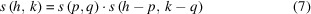
It should be noted that the latter relationship also appears in one of the other three articles published in *Acta Crystallographica* in 1952. There, William H. Zachariasen derived the same relationship and used it in a procedure quite similar to symbolic addition to determine the structure of metaboric acid (Zachariasen, 1952 ▸). However, as all authors agreed, the sign relationship in equation 7 states that for a triplet of related structure factors with all large magnitudes the signs (the phase angles) must be in balance on each side. Thus, for a centrosymmetric structure four configurations exist (Table 1 ▸).

**Figure 2:**
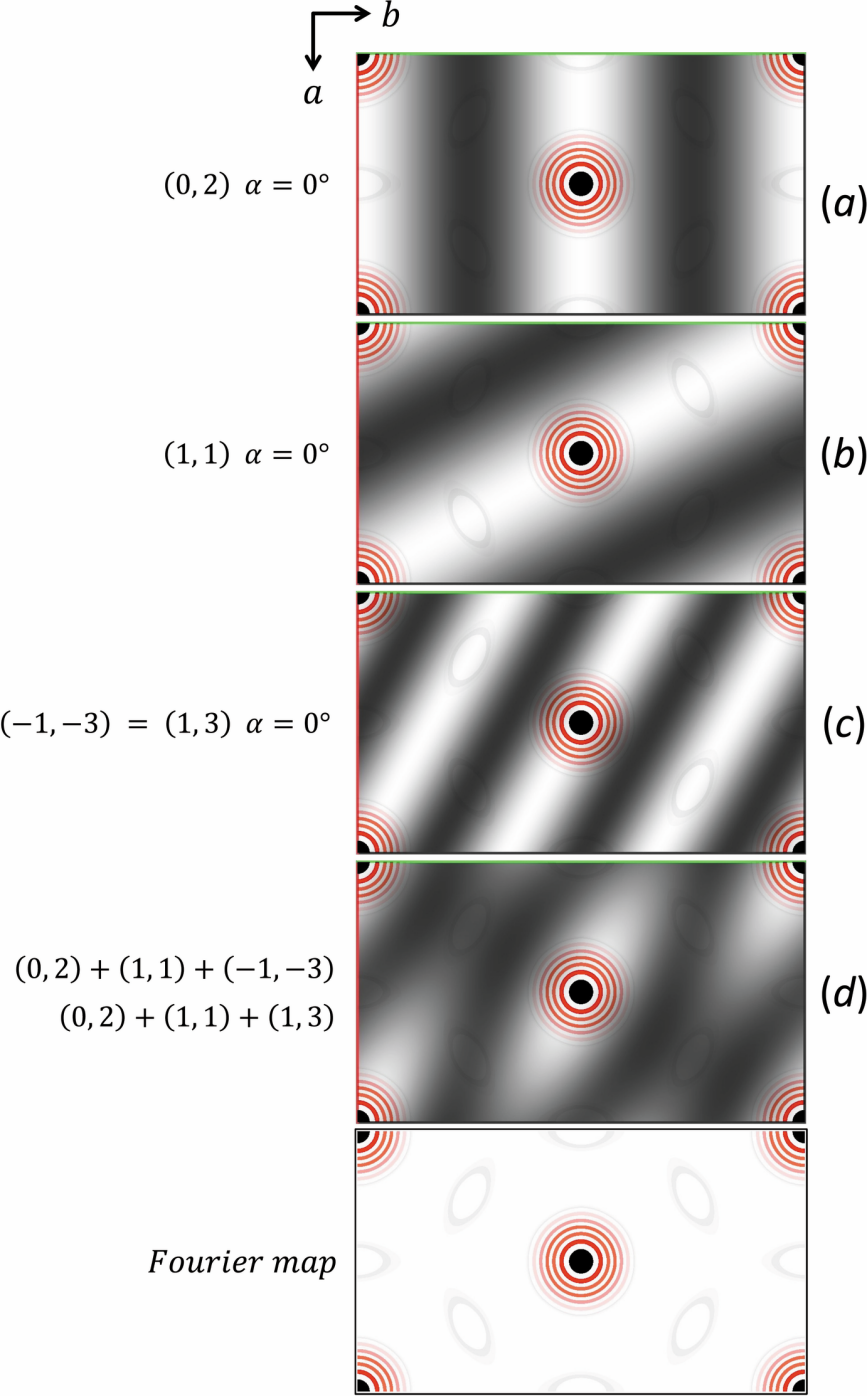
The image at the bottom shows the Fourier map of a two-dimensional unit cell of a one-atom structure, with atom positions at *xy =* (0, 0) and (1/2, 1/2). The corresponding diffraction pattern is shown in Fig. 1 ▸. For this structure, a triplet with all strong structure factors is (0, 2) (1, 1) (1, 3). As shown in (*a*) and (*b*), the two Fourier waves (0, 2) and (1, 1) have both a 0° phase angle and begin with a maximum at the top left corner of the unit cell, producing bright maxima at the positions of the atoms. According to Table 1 ▸ and Equation 8, the Fourier waves (1, 3) and its Friedel counterpart (−1, −3) must have the same zero phase angle to agree with Sayre’s proposal. The sum of the corresponding Fourier waves (*a*) to (*c*) is shown in map (*d*), which exhibits pronounced maxima at the positions of the atoms.

As seen from the last column of Table 1 ▸, the product of the three signs yields always plus and the sum of the three structure factor phase angles (modulo 360°) in a strong triplet is always zero. Therefore, one can write for the phases of the three structure factors:
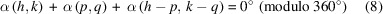
If ‘anomalous scattering’ is absent, which is usually the case, the structure factors *F*(*hk*) and *F*(−*h* − *k*) have the same amplitude and phase value (Friedel’s law). Thus, inverting the signs of the indices of the structure factor simply means that the corresponding Fourier wave traverses the unit cell in the opposite direction. This relation allows always to replace α(*hk*) in equation 8 by α(−*h* − *k*) if needed. Fig. 2 ▸ illustrates the latter for the triplet (0,2) (1,1) (1,3) of a hypothetical two-dimensional structure.

However, equation 7 and the thereof derived equation 8 assume a strict relation between the three structure factors in the triplet, which means that the value of a structure factor phase can always be calculated from the values of the other two structure factor phases. Although this assumption will be used in the examples discussed later, it should be kept in mind that this relationship is not inherently deterministic, as implied by the equality sign. In fact, the equality sign correctly means ‘approximately equal’ or ‘highly probable’. The reason for this is explained in the following. In the probabilistic theory of Hauptman & Karle, equation 7 is known as the Σ_2_ formula which makes the same statement as Sayre’s or Zachariasen’s equations under the condition of atomicity and for structure factors with large magnitudes (Karle, 1985 ▸; Cochran & Woolfson, 1954 ▸),
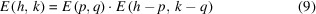
The *E*s in equation 9 are the so-called normalized structure factors, which are calculated according to equation 10 from the structure factor amplitudes |*F*(*hk*)| and the atomic scattering factors *f* (Karle, 1985 ▸). Normalized structure factors were introduced to compensate for the systematic decrease in the magnitude of the structure factors resulting from the fall-off of the atomic scattering factors with increasing scattering angle. As a result, it becomes easier to identify the structure factors with large magnitudes in lists of *E* values. This approach will also be used throughout the examples presented in Section 3. However, in effect this normalization procedure simulates point scattering atoms that have a constant scattering power over all diffraction angles (Karle, 1985 ▸),
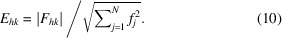
The corresponding structure factor phase 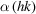 of 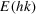 in the Hauptman & Karle theory are given by equation 11 (Karle, 1985 ▸; Hauptman, 1991 ▸),
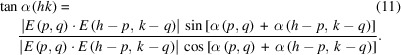
For the aforementioned centrosymmetric case there exist only two scenarios for equation 11: either the sum of both phases inside the brackets becomes zero [which is only possible if α(*p*,*q*) = α(*h* − *p*,*k* − *q*) = 180°], or the sum of the phases is 180°. The latter means that one of the two phases inside the brackets is zero and the other is 180°. In the first case of 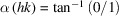 with both numerator and denominator positive α(*h,k*) becomes zero, and in the second case of 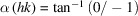 with numerator positive and denominator negative α(*h,k*) is 180°. Thus in agreement with Sayre’s proposal, the sum of the three structure factor phases is always zero. To check that this relationship is very likely, direct methods make use of the function in equation 12 that allows one to estimate the probability that *E*(*hk*) is positive (Woolfson, 1954*b* ▸; Cochran & Woolfson, 1955 ▸),
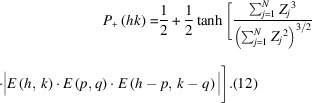
In the latter equation, *Z_j_* are the atomic numbers of the *j*th atom in the unit cell containing *N* atoms (Karle, 1985 ▸). For the case where the structure contains only one type of atom, the quotient inside the brackets of the tangens hyperbolicus becomes
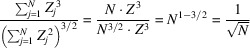
This yields the simplified formula in equation 13 (Ladd & Palmer, 2013 ▸),
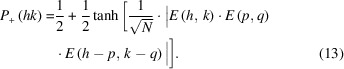
The last equation shows that the probability of correctly predicting the phase value of the structure factor *E*(*hk*) *via* the triplet relationship decreases by 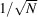 for structures with *N* atoms. To counterbalance this effect and increase the probability that the triplet relationship provides the correct phase value, triplets that form large products *E*(*h*, *k*) · *E*(*p*, *q*) · *E*(*h* − *p*, *k* − *q*) are used for phase assignment. In practice, this is achieved by eliminating ll *E* values that fall below a certain threshold. In the following examples, this threshold was set to *E*(*hk*) > 1 in all cases to ensure that the relationship in equation 7 could be applied.

## Symbolic addition

The relationship in equation 8 is the key formula on which the here presented symbolic addition method is based. Since the phase angles of the structure factors are unknown in the beginning, they are replaced by variable names such as *A*, *B*, *C* and so on,
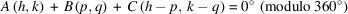
However, before solving this and other similar triplet equations, it is necessary to define a position in the unit cell to which all these unknown phase values refer. In a centrosymmetric, two-dimensional primitive unit cell, this unit-cell origin can be defined by assigning arbitrary phase values to two structure factors. There are, however, some restrictions to consider. Table 2 ▸ shows by which angle the structure factor phases are changed when the origin of a two-dimensional unit cell with square, rectangular or oblique symmetry is moved from one position to another. Similar tables for the corresponding three-dimensional cases can be found in books by Ladd & Palmer (2013 ▸), Stout & Jensen (1989 ▸), and a review article by Woolfson (1971 ▸). A complete guide for selecting the origin fixing phases for all 230 space groups is found in the *International Tables for Crystallography* Volume IV (Karle, 1973 ▸). In the present context it is important to interpret Table 2 ▸ correctly, as it only shows the amount by which the phases of a structure factor change when the origin is shifted. The table does not state, however, that the phases must be fixed to these values, since the actual phase value of a structure factor is obtained through calculation of the structure factor from the atomic coordinates (for the one-dimensional case see, for example, Weirich 2026*a* ▸). Thus, Table 2 ▸ shows, that structure factors with *hk* all even do not change their phase value when the origin of the unit cell is shifted, *e.g.* from the cell corner (*x* = 0, *y* = 0) to the center (*x* = 1/2, *y* = 1/2) of the unit cell. All of these all even *hk* structure factors are origin-independent (crystallographers name them therefore *structure invariant*). These *hk* all even structure factors cannot be used for fixing the origin of the unit cell at a certain position, because combining them with any of the other possibilities *hk* odd/even, even/odd or odd/odd will always lead to an ambiguity between two origin positions. Thus, for the task of fixing the unit-cell origin, any two other combinations from the remaining parity groups will be permitted, as long as the two *hk* values do not add up to all even. Hence, an assignment of phases to structure factors (1, 2) and (2, 5) will fix the origin, whereas the assignment to the pair (1, 2) and (3, 2) will not.

As mentioned earlier, the phases assigned to the two origin fixing reflections form the basis were all the other phase values refer to and from which they can be derived. This will be demonstrated using a simple example. Assuming three triplets with unknown structure factor phases, which are expressed according to equation 9 in algebraic form:
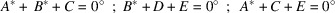
With the origin fixing structure factor phases 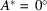 and 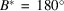, all unknown phase values can be determined as shown below. In addition to the standard calculation rule that +180° − 180° = 0°, the following calculation rules for the phase angles apply:

180° + 180° = −180° − 180° = 0° and +180° = −180°.

Beginners can easily remember these rules by thinking of a wall clock where the hand starts at 12 o’clock, which corresponds to 0° phase angle. In this analogy, the 6 o’clock position corresponds to a phase angle of 180°. Adding or subtracting two six-hour intervals (each corresponding to +180 or −180 degrees, respectively) will bring the hand back to the 12 o’clock position, which corresponds to 0°. This example also shows that, in order to finish with the hand at the 12 o’clock position, either two phases can have 180° or all phases must be zero.
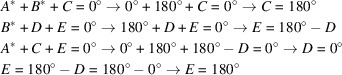
Proof:
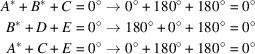
It may be worth emphasizing that in practice the success of the method outlined above largely depends on the careful selection of the origin-fixing structure factors. These should appear in as many triplet relationships as possible to keep the number of unknown phases in the triplets low. At the same time, these structure factors should have large magnitudes because this makes the phase assignment more reliable as stated by equation 13. Therefore, both criteria must be considered when selecting the origin-defining structure factors. As outlined in Section 2, the selection of valid structure factors that participate in triplets is typically achieved through the calculation of normalized structure factors 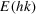, from which only those above a specified threshold are considered for assigning phases.

### Step-by-step examples

**Figure 3:**
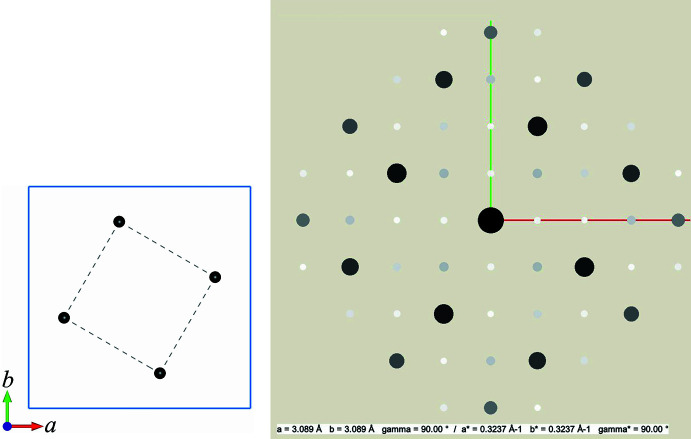
The image on the left shows the unit cell of the two-dimensional model used in Example 1 with carbon atoms shown as black spots (for CIF file, see supporting information). The lattice parameter of the square unit cell (indicated by the light-blue frame) has been set to *a *= 3.089 Å, in order that the by dashed lines indicated in plane C—C distances agree with the average C—C single bond distance of 1.54 Å. The image on the right shows the corresponding calculated diffraction pattern with intensities proportional to the squares of the structure factors (a* = red, b* = green). Note that the inner eight strong diffraction spots resemble the diffraction pattern of a square unit cell formed by the four atoms in the model.

The first two examples discussed next do not represent actual structures, since the goal of these is only to illustrate the principles of the symbolic addition method by simple and easy to follow cases that do not require much time to work through. Both model structures were created using standard presentation software. The pixel graphics obtained in this way were then used to determine the absolute 2D atomic coordinates in pixel units. These coordinates were then turned into fractional coordinates of the atoms (see Tables A5 and B5 in Appendices A and B, respectively). To make the models as realistic as possible, the scale factor for the unit cells was set so that the largest in-plane atom distances match the average single C—C bond distance in organic compounds, which is 1.54 Å. The final obtained models are shown in Figs. 3 ▸ and 5, together with their calculated diffraction patterns. All crystallographic calculations such as calculations of normalized structure factors *E*(*hk*) and possible triplets, were performed using the educational software *EasyDiSi* (Weirich, 2026*b* ▸). *VESTA* (Momma & Izumi, 2011 ▸) was used to produce all molecular graphics for structure representation.

#### Example 1

**Figure 4:**
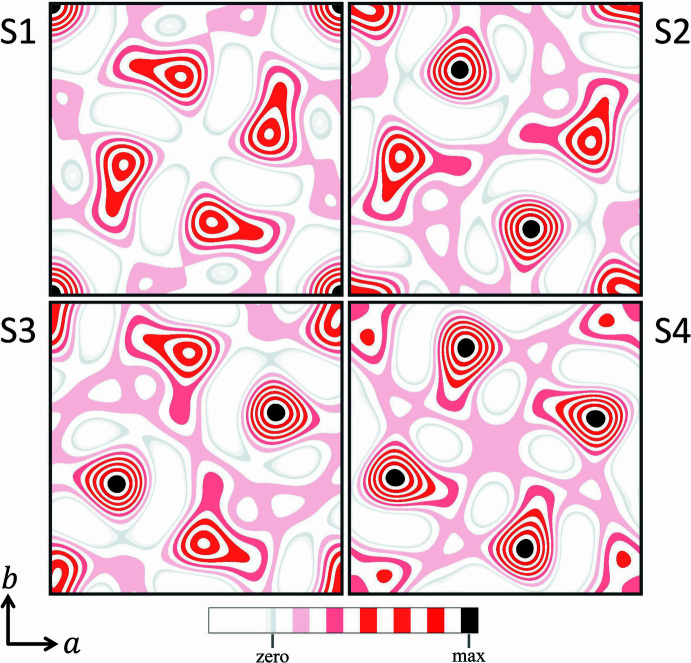
Fourier maps of the four potential solutions for the two-dimensional model structure of Example 1. The maps were calculated with the 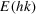 values and phases listed in Table A3, with *A* = *B* = 0° (solution S1), *A* = 0° and *B* = 180° (S2), *A* = 180° and *B* = 0° (S3), and *A* = *B* = 180° (S4). Visual comparison with the structure model in Fig. 3 ▸ shows that the maxima in Fourier map S4 correspond with the positions of the carbon atoms, and thus indicate the correct solution. When the locations of the peak maxima in map S4 are checked against those of the initial model, it is found that all four positions are within 0.04 Å of their correct position (see Table A5).

Calculations of normalized structure factors for *E*(*hk*) > 1 and crystallographic resolution of 0.7 Å, yielded initially 48 triplets. This number could be reduced to just four unique triplets by manually removing duplicate entries that contained the structure factors but in different order. The normalized structure factors were then ordered according to their *hk* parity (even-even, even-odd, *etc*.) and Friedel pairs *hk* and *−h−k* were labeled with characters from *A* to *H*. The corresponding Σ_2_ lists (Karle & Karle, 1966 ▸) of normalized structure factors and the algebraic forms of the triplets are shown in Tables A1 and A2 in Appendix A. The origin of the two-dimensional unit cell was fixed by assigning phase values to the structure factors C* (*h* even, *k* odd)and E* (*h* odd, *k* even), as these have large magnitudes and participate most frequently in the triplets lists in Table A1. To allow a direct comparison of the results after phase assignment with the initial model, the phase values of the structure factors for C* and E* (both are 0°) were taken from the list of calculated structure factors. The subsequent evaluation of the algebraic forms of the four triplets is as follows:
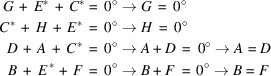
Substituting *G* and *H* with zero, and *D* and *F* with *A* and *B*, in the list in Table A1 shows that only *A* and *B* remain as undetermined phases. This ambiguity can be resolved by calculating Fourier maps with assigning trial phases to *A* and *B.* This procedure works in a similar manner to Woolfson’s method of permutation syntheses (Woolfson 1954*a* ▸), but with a much smaller number of undetermined phases. As each phase value in the case of a centrosymmetric structure is binary [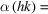 0° or 180°], the number of permutations is given by 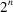, where *n* refers to the number of unknown phases. Hence, for the present case it is necessary to calculate four Fourier maps, of which one would represent the correct solution (see Table A3 in Appendix A). A corresponding table containing the full list of phase values is provided in Table A4. The Fourier maps calculated from these values are shown in Fig. 4 ▸. Comparison of the Fourier maps with the initial structural model in Fig. 3 ▸ shows that Fourier map S4 represents the correct solution. The latter is also verified by the assigned phases for the trial set S4 in Table A4, which has the same phase values as calculated from the model. However, since the Fourier map of solution S4 was calculated using only 16 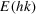 values rather than all 60 structure factors, it is worth considering whether the atomic positions have been correctly determined. Comparing the atomic positions determined from E-Map S4 with those of the initial model, however, reveals that all four positions are within 0.04 Å of their correct position (see Table A5).

#### Example 2

**Figure 5:**
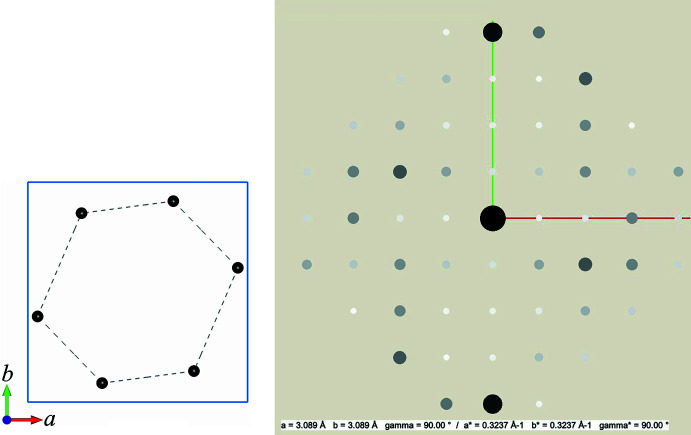
The left image shows the unit cell of the two-dimensional model used in Example 2 with carbon atoms shown as black marbles (for CIF file, see supporting information). As before, the model has been scaled so that the longest C–C distances in plane match with the average C–C single bond distances. The corresponding calculated diffraction pattern, with intensities proportional to the square of the structure factor amplitude, is shown in the image on the right (a* = red, b* = green).

Calculations for the model of Example 2 were performed similar as for Example 1, which yielded 96 triplets with *E*(*hk*) > 1 at a crystallographic resolution of 0.7 Å. Assigning letters to the unknown phases and removing duplicate entries from the initial list of triplets led to a unique set of eight triplet relations. The corresponding lists of normalized structure factors and the algebraic forms of the triplets are given in Tables B1 and B2 in Appendix B. For the present case, the origin of the two-dimensional unit cell was fixed by assigning zero to the structure factor phase of E* (*h* even, *k* odd) and 180° to H* (*h* odd, *k* even). Again, the criterion for selecting these structure factors was that they have large magnitudes and participate with the highest frequency in the triplets in Table B2. A subsequent evaluation of the algebraic forms of the triplets yields:
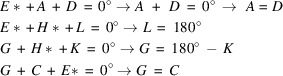

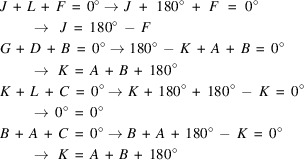
This leads to the following substitutions for Table B1
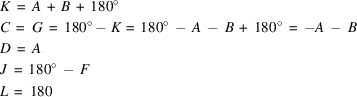
This procedure leaves only *A*, *B* and *F* as undetermined, which requires 2^3^ Fourier maps to be calculated to obtain the correct solution (see Table B3). Table B4 gives the complete list of phase values after assigning the trial phases. The Fourier maps calculated from this list are shown in Figure B1. Comparison of the latter with the initial structural model in Fig. 5 ▸ shows that Fourier map S6 is the correct solution. The latter is also confirmed by the assigned phases of trial set S6 in Table B4, which shows all phase values the same as calculated from the model. To judge the quality of the solution, the positions of the atom peaks in the S6 Fourier map are compared with those of the model structure in Fig. 5 ▸. The last column in Table B5 shows, that all six atoms in the unit cell are located within 0.19 Å to the positions of the model.

#### Hexa­methyl­benzene

**Figure 6:**
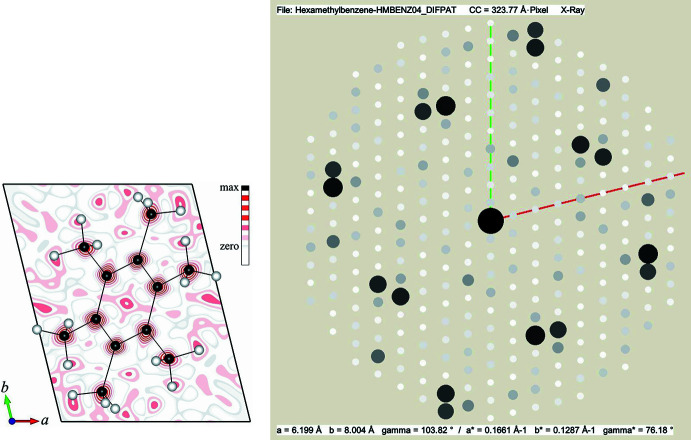
The left image shows the Fourier map of the projected structure of hexa­methyl­benzene C_6_(CH_3_)_6_ as calculated from the 48 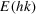 values in Table C1 together with the projected molecule as overlay (C = black, H = white marbles). The corresponding calculated diffraction pattern with intensities proportional to the square of the structure factor amplitude is shown in the image on the right. The reciprocal unit cell axes are indicated in the diffraction pattern as red and green lines for a* and b*, respectively.

The following example uses the triclinic structure of hexa­methyl­benzene C_6_(CH_3_)_6_ in projection along the *a* axis as a structure that truly exists. The crystal structure of hexa­methyl­benzene was first determined by Kathleen Lonsdale (1928 ▸, 1929 ▸) and is mentioned in the textbook by Glusker & Trueblood (2010 ▸, p. 124) for demonstrating the principles of triplet sign relationships. However, for the present case the data from Hubig *et al.* (2001 ▸) are used, which refer to a different unit-cell origin that locates the whole molecule in the center of the unit cell. According to the latter authors, the compound crystallizes in space group *P*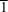 (space group No. 2) with lattice parameters *a* = 5.2602 (2) Å, *b* = 6.1990 (3) Å, *c* = 8.0040 (3) Å, α = 103.8180 (10)°, β = 98.7180 (10)°, γ = 100.1920 (10)°. Hence, the projected structure for the present use case has the assigned parameters *a*′ = 6.199 Å, *b*′ = 8.004 Å, γ′ = 103.82° with plane group symmetry *p*2 (No. 2). The corresponding projected structure and its calculated diffraction pattern are shown in Fig. 6 ▸. Calculations for this model yielded 48 structure factors with *E*(*hk*) > 1 at a crystallographic resolution of 0.7 Å (Table C1). After assigning characters to all phase values, 25 unique triplet relationships could be obtained (Table C2). For later comparison with the original structure, the origin of the two-dimensional unit cell was fixed by assigning 180° to both, F* (*h* even, *k* odd) and P* (*h* odd, *k* even). As for the other examples, these structure factors have large magnitudes and participate with the highest frequency in the triplets in Table C2. The subsequent evaluation of the algebraic forms can be carried out in a similar way, as shown earlier. From the expressions containing the origin fixing structure factors F* and P* it can readily be derived that *Z* = 0°, *C* = *T*, *U* = *A*, and *W* = *D*. Moreover, it can be derived that *G* = *L* = *N* = 180° − *C*, *E* = *R* = 180° − *A*, *Q* = *H* = 180° − *D*, and *V* = 180° − *S*. Substitution of these in Table C2 yields *X* = *A* + *C*, *B* = *A* + *C*, *M* = 180° + *C* − *A*, *J* = 180° + *D* − *C*, *K* = 180° + *C* – *D*, and *Y* = *C* + *D*. Thus, only *A*, *C*, *D*, and *S* are left who cannot be further replaced. Accordingly 2^4^ Fourier maps are required for identifying the correct solution. The corresponding required phase permutations are listed in Table C3.

Table C4 in Appendix C provides a comprehensive list of phase values obtained with the substitutions from Table C3 and the thereof calculated Fourier maps are shown in Figure C1. Comparison of the latter with the calculated map from the structural model in Fig. 6 ▸ shows that the correct solution is represented by phase set S3 (see also the allocated phases for trial set S3 in Table C4). In this case 48 *E*(*hk*) values out of 308 structure factors were used to calculate the Fourier maps. The thereby determined projected positions of the 12 carbon atoms in the unit cell in Table C5 agree within less than 0.13 Å with the model of Hubig and coworkers (Hubig, *et al.* 2001 ▸).

## Discussion

Although current direct methods software employs more sophisticated probabilistic algorithms (*e.g.* Giacovazzo, 2010 ▸; Ladd & Palmer, 2013 ▸; Burla *et al.*, 2015 ▸), the underlying logical structure of phasing diffraction data could be demonstrated by solving three projected structures with varying degrees of complexity using triplet phase relationships. Beyond the introduction of triplet relationships, the worked examples also show several other important aspects. Thus, it was demonstrated that selecting origin-defining structure factors at the initial stage is crucial for symbolic addition, because the selected structure factors must firstly participate most frequently in the employed triplet relations, and secondly, they must have large, normalized structure factor magnitudes to enable reliable phase assignment. Hence, for the real-case example of the hexa­methyl­benzene projection, it was shown that careful selection of origin-fixing structure factors allowed the reduction of the number of independent unknown phases to just four after evaluating 25 triplet relationships. On the other hand, a less strategic selection of origin-defining structure factors would have led to a larger number of undetermined variables and a significantly larger number of required Fourier trials, since the latter increases by a factor of 2*^n^*, where *n* is the number of independent variables. Furthermore, for all three worked examples, it was demonstrated that, although calculated from a small subset of large normalized structure factors, the resulting Fourier maps reproduce atomic positions within 0.04–0.19 Å of the initial model coordinates. This finding demonstrates the redundancies inherent in strong triplets and provides clear evidence for the approach of using dominant triplet relationships for assigning the structure factor phases.

For the didactic purpose of providing a basic introduction to direct methods, this presentation is focused on two-dimensional centrosymmetric projections using ideal simulated data. However, as the inventors of the symbolic addition method, Isabella and Jerome Karle, repeatedly proved in the 1960s that this concept works equally well with three-dimensional diffraction data, without changing the logical structure of the symbolic addition procedure (Karle & Karle, 1963 ▸, 1964 ▸, 1966 ▸). Unlike the examples shown here, non-centrosymmetric structures have phase angles that are not restricted to 0 and 180°, and triplet relationships in fact take the form of approximate equalities rather than strict sign constraints. Consequently, the effort required to solve acentric structures using symbolic addition increases, as the structure factor phases can take any value between 0° and 360°. For example, the Fourier map of the acentric l-arginine dihydrate structure required the Karles to assign 400 phases with |*E*| > 1.0 (Karle & Karle, 1964 ▸). The significant increase in algebraic complexity involved in doing all this manually was one of the main reasons why others developed computer software to automate this labor-intensive process (*e.g.* Schenk, 1971*a* ▸,*b* ▸,*c* ▸; Woolfson, 1983 ▸).

## Conclusion

Although the manual symbolic addition procedure is today only of historical interest, its methodology remains a valuable teaching tool for demonstrating crystallographic phase determination. Thus, working through the three provided examples introduces beginners to the entire process of structure determination via triplet relationships from subsets of normalized structure factors, covering every step from defining the unit cell origin to the final Fourier maps. As the provided data sets range from early beginner to more advanced levels, they are suitable for use in various teaching scenarios. At each level, students could initially be given only the listings of the *E* values and their frequencies in triplet relationships, along with the predefined triplets. After evaluating the algebraic forms through symbolic addition, they could then be supplied with the complete structure factor lists and the corresponding Fourier maps to identify their solution. Using printouts of the Fourier maps and determining the fractional coordinates from them with a ruler would finally allow the coordinates to be verified numerically against the initial model. This material is, however, also suitable for independent study, as the text provides sufficient explanation of the context and theory behind the symbolic addition method, and describes all stages from start to finish. Thus, in light of the frequent treatment of structure solution as a routine computational task, the fully worked examples in this publication may contribute to the greater value of symbolic addition in crystallography courses, as the examples clearly outline the principles on which modern direct methods software is based. Moreover, the understanding gained from manually working through the examples may not only teach users the conceptual ideas behind direct methods but also enable them to identify reasons for issues and develop workarounds when routine automated direct methods fail or when diffraction datasets are examined that are beyond the standard.

## Supplementary Material

Appendices A, B, C. DOI: 10.1107/S2056989026003300/oi2035sup2.docx

Supporting information file. DOI: 10.1107/S2056989026003300/oi2035sup3.docx

Supporting information file. DOI: 10.1107/S2056989026003300/oi2035sup4.docx

Supporting information file. DOI: 10.1107/S2056989026003300/oi2035figA1sup5.tif

Supporting information file. DOI: 10.1107/S2056989026003300/oi2035figC1asup6.tif

Supporting information file. DOI: 10.1107/S2056989026003300/oi2035figC1bsup7.tif

Zip archive. DOI: 10.1107/S2056989026003300/oi2035sup8.zip

Crystal structure: contains datablock(s) Example-1. DOI: 10.1107/S2056989026003300/oi2035sup9.cif

Crystal structure: contains datablock(s) Model-2. DOI: 10.1107/S2056989026003300/oi2035sup10.cif

Crystal structure: contains datablock(s) Hexamethylbenzene-HMBENZ04. DOI: 10.1107/S2056989026003300/oi2035sup11.cif

## Figures and Tables

**Table 1:** The four possible sign configurations for a triplet of a centrosymmetric structure or centrosymmetric structure projection. As shown in the last column the product of all phase’s values modulo 360° yields always zero (+)

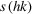	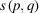	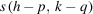	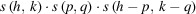
+ (0 °)	+ (0 °)	+ (0 °)	+ (0 °)
+ (0 °)	− (180 °)	− (180 °)	+ (0 °)
− (180 °)	+ (0 °)	− (180 °)	+ (0 °)
− (180 °)	− (180 °)	+ (0 °)	+ (0 °)

**Table 2:** The table lists the changes of the structure factor phases when the unit cell origin is shifted from the cell corner to another symmetry center in a two-dimensional centrosymmetric unit cell. Note that only the structure factors where the *hk* all even, never show a phase flipping when the origin is shifted. Due to this invariance these structure factors will always lead to an ambiguity between two origin positions when combined with any other possibilities in the other columns with *hk* odd/even, even/odd or odd/odd

Centers of symmetry at	*h* even *k* even	*h* odd *k* even	*h* even *k* odd	*h* odd *k* odd
*x* = 0, *y* = 0	+ 0 °	+ 0 °	+ 0 °	+ 0 °
*x* = 1/2, *y* = 0	+ 0 °	+ 180 °	+ 0 °	+ 180 °
*x* = 0, *y* = 1/2	+ 0 °	+ 0 °	+ 180 °	+ 180 °
*x* = 1/2, *y* = 1/2	+ 0 °	+ 180 °	+ 180 °	+ 0 °

## References

[bb1] Berry, J. F. & Guzei, I. A. (2026). *Acta Cryst.* E**82**, 107–120. 10.1107/S2056989025010527 PMC12810306 41551565

[bb2] Buerger, M. J. (1970). *Contemporary Crystallography*. New York: McGraw-Hill Book Company.

[bb3] Burla, M. C., Caliandro, R., Carrozzini, B., Cascarano, G. L., Cuocci, C., Giacovazzo, C., Mallamo, M., Mazzone, A. & Polidori, G. (2015). *J. Appl. Cryst.* **48**, 306–309.

[bb4] Cochran, W. (1952). *Acta Cryst.* **5**, 65–67.

[bb5] Cochran, W. & Woolfson, M. M. (1954). *Acta Cryst.* **7**, 450–451.

[bb6] Cochran, W. & Woolfson, M. M. (1955). *Acta Cryst.* **8**, 1–12.

[bb7] Cranswick, L. M. D. (2008). *Acta Cryst.* A**64**, 65–87. 10.1107/S0108767307051355 18156674

[bb9] Germain, G., Main, P. & Woolfson, M. M. (1970). *Acta Cryst.* B**26**, 274–285.

[bb10] Germain, G., Main, P. & Woolfson, M. M. (1971). *Acta Cryst.* A**27**, 368–376.

[bb11] Germain, G. & Woolfson, M. M. (1968). *Acta Cryst.* B**24**, 91–96.

[bb12] Giacovazzo, C. (2010). *Direct Methods. * In *International Tables for Crystallography* Vol. B, ch. 2.2, 215–243. https://doi.org/10.1107/97809553602060000764.

[bb13] Glusker, J. P. & Trueblood, K. N. (2010). *Crystal Structure Analysis - A Primer,* 3rd ed. Oxford University Press.

[bb14] Hauptman, H. A. (1991). *Rep. Prog. Phys.* **54**, 1427–1454.

[bb15] Hauptman, H. & Karle, J. (1952). *Acta Cryst.* **5**, 48–59.

[bb16] Hauptman, H. & Karle, J. (1954). *Acta Cryst.* **7**, 369–374.

[bb17] Hauptman, H. & Karle, J. (1957). *Acta Cryst.* **10**, 267–270.

[bb18] Hubig, S. M., Lindeman, S. V. & Kochi, J. K. (2001). *CSD Communication* (CCDC 138870, refcode HMBENZ05). CCDC, Cambridge, England. https://doi.org/10.5517/cc4nhpy, https://pubchem.ncbi.nlm.nih.gov/compound/6908.

[bb19] Irmer, E. (2025). *J. Appl. Cryst.* **58**, 1802–1809. 10.1107/S1600576725007459 PMC12502877 41064427

[bb20] Ito, S., White, F. J., Okunishi, E., Aoyama, Y., Yamano, A., Sato, H., Ferrara, J. D., Jasnowski, M. & Meyer, M. (2021). *CrystEngComm* **23**, 8622–8630.

[bb21] Karle, I. L. & Karle, J. (1963). *Acta Cryst.* **16**, 969–975.

[bb22] Karle, I. L. & Karle, J. (1964). *Acta Cryst.* **17**, 835–841.

[bb23] Karle, J. (1973). *Direct Methods for Structure Determination: Origin Specification, Normalized Structure Factors, Formulas, and the Symbolic Addition Procedure for Phase Determination.* In *International Tables for X-ray Crystallography: Revised and Supplementary Tables to Volumes II and III (Volume 4)* edited by J. A. Ibers and W. C. Hamilton, sec. 6. Birmingham: Kynoch Press.

[bb24] Karle, J. (1985). *Nobel Prize Lecture* pp. 233 https://www.nobelprize.org/prizes/chemistry/1985/karle/lecture/.

[bb25] Karle, J. & Hauptman, H. (1950). *Acta Cryst.* **3**, 181–187.

[bb26] Karle, J. & Hauptman, H. (1957). *Acta Cryst.* **10**, 515–524.

[bb27] Karle, J. & Karle, I. L. (1966). *Acta Cryst.* **21**, 849–859.

[bb28] Ladd, M. & Palmer, R. (2013). *Structure Determination by X-ray Crystallography. Analysis by X-rays and Neutrons* 5th ed. Dordrecht: Springer Science+Business Media. https://doi.org/10.1007/978-1-4614-3954-7

[bb29] Light, M. E. (2004). *Automating the Single Crystal X-ray Diffraction Experiment.* European Crystallography Meeting - ECM 22, Budapest, Hungary, 26–31 Aug 2004. https://eprints.soton.ac.uk/id/eprint/9107.

[bb30] Lonsdale, K. (1928). *Nature* **122**, 810.

[bb31] Lonsdale, K. (1929). *Proc. R. Soc. London. Ser. A* **123**, 494–515.

[bb32] Massa, L. (2018). *Isabella Karle: Crystallographer Par Excellence.* In *The Posthumous Nobel Prize in Chemistry* Vol. 2, *Ladies in Waiting for the Nobel Prize* pp. 283–294. https://doi.org/10.1021/bk-2018-1311.ch012.

[bb33] Massa, W. (2004). Crystal Structure Determination, 2nd ed. Berlin: Springer-Verlag. https://doi.org/10.1007/978-3-662-06431-3.

[bb60] Momma, K. & Izumi, F. (2011). *J. Appl. Cryst.* **44**, 1272–1276.

[bb34] Rigaku Corporation (2016). *Rigaku J.* **32**, 31–34.

[bb35] Sayre, D. (1952). *Acta Cryst.* **5**, 60–65.

[bb36] Schechter, B. (1985). *Phys. Today* **38**, 20–21.

[bb37] Schenk, H. (1971*a*). *Acta Cryst.* B**27**, 2037–2039.

[bb38] Schenk, H. (1971*b*). *Acta Cryst.* B**27**, 2039–2040.

[bb39] Schenk, H. (1971*c*). *Acta Cryst.* B**27**, 2040–2042.

[bb40] Schenk, H. (1984). *An Introduction to Direct Methods. The Most Important Phase Relationships and their Application in Solving the Phase Problem.* IUCr Teaching Pamphlet No. 17, International Union of Crystallography. University College Cardiff Press. https://www.iucr.org/education/pamphlets/17.

[bb41] Schlick, T. (2021). *Isabella L. Karle: A Crystallography Pioneer.**DNA Cell Biol*. **40**, 843–847. https://doi.org/10.1089/dna.2021.0372 10.1089/dna.2021.0372 PMC8309433 34129390

[bb42] Sheldrick, G. M. (2008). *Acta Cryst.* A**64**, 112–122. 10.1107/S0108767307043930 18156677

[bb43] Stout, G. H. & Jensen, L. H. (1989). *X-ray Structure Determination. A Practical Guide* 2nd ed. New York: John Wiley & Sons.

[bb44] Weirich, T. E. (2001). *Acta Cryst.* A**57**, 183–191. 10.1107/s0108767300014070 11223505

[bb45] Weirich, T. E. (2003). *Electron diffraction structure analysis: structural research with low-quality diffraction data**Z Kristallogr. Cryst. Mater.* **218**, 269–278. https://doi.org/10.1524/zkri.218.4.269.20744.

[bb46] Weirich, T. E. (2026*a*). *Acta Cryst.* E**82**, 235–243. 10.1107/S2056989026000745 PMC12874239 41657498

[bb47] Weirich, T. E. (2026*b*). *EasyDISI: An ImageJ-Based Educational Framework for Simulation and Visualizing Two-Dimensional Diffraction.**Acta Cryst.* E**82** (Submitted).

[bb48] Weirich, T. E., Zou, X. D., Ramlau, R., Simon, A., Cascarano, G. L., Giacovazzo, C. & Hovmöller, S. (2000). *Acta Cryst.* A**56**, 29–35. 10.1107/s0108767399009605 10874414

[bb49] Wheeler, K. A. (2026). *Acta Cryst.* E**82**, 313–319. 10.1107/S2056989026000939 PMC12961664 41799048

[bb50] Woolfson, M. M. (1954*a*). *Acta Cryst.* **7**, 65–67.

[bb51] Woolfson, M. M. (1954*b*). *Acta Cryst.* **7**, 61–64.

[bb52] Woolfson, M. M. (1971). *Rep. Prog. Phys.* **34**, 369–434.

[bb53] Woolfson, M. M. (1983). *Phys. Bull.* **34**, 330–333.

[bb54] Woolfson, M. M. (1987). *Acta Cryst.* A**43**, 593–612.

[bb55] Woolfson, M. M. (1997). *An Introduction to X-ray Crystallography* 2nd ed. Cambridge University Press.

[bb56] Woolfson, M. M. (2014). *Phys. Scr.* **89**, 108001.

[bb57] Zachariasen, W. H. (1952). *Acta Cryst.* **5**, 68–73.

[bb58] Zheng, S.-L., Litak, N. P., Campbell, M. G., Handford, R. C., Dogutan, D. K., Carsch, K. M. & Betley, T. A. (2025). *J. Appl. Cryst.* **58**, 269–275.

[bb59] Zou, X., Hovmöller, S. & Oleynikov, P. (2011). *Electron Crystallography. Electron Microscopy and Electron Diffraction.* Oxford University Press.

