# Supplementary material for: Revisiting symbolic addition: a step-by-step introduction to manual direct methods
Source: Acta Crystallogr E Crystallogr Commun. 2026 Apr 10;82(Pt 5):534–43. doi: 10.1107/S2056989026003300 (PMC13148211; doi:10.1107/S2056989026003300)
Supplement: Supplementary file 1 [file e-82-00534-sup2.docx]

**Appendix A**

**Table A1** Σ_2_ listing of normalised structure factors *E*(*hk*) for the hypothetical structure of example 1 ordered by *hk* parity. Initially unknown phase values 𝛼(*hk*) are replaced by letters. The even-odd and odd-even normalised structure factors marked by a star (*) were both assigned a phase value of zero, which fixes the origin of the unit cell at x=0 and y=0 (see Table 2).

| **Frequency**  **in triplets** | ***h*** | ***k*** | ***E*(*hk*)** | **𝛼(*hk*)** |
| --- | --- | --- | --- | --- |
| *h* even*, k* even | | | | |
| 1 | 0 | -4 | 1.34 | *A* |
| 1 | 0 | 4 | 1.34 | *A* |
| 1 | 4 | 0 | 1.32 | *B* |
| 1 | -4 | 0 | 1.32 | *B* |
| *h* even*, k* odd | | | | |
| 3 | -2 | 1 | 1.83 | *C** |
| 3 | 2 | -1 | 1.83 | *C** |
| 1 | 2 | 3 | 1.5 | *D* |
| 1 | -2 | -3 | 1.5 | *D* |
| *h* odd*, k* even | | | | |
| 3 | -1 | -2 | 1.83 | *E** |
| 3 | 1 | 2 | 1.83 | *E** |
| 1 | -3 | 2 | 1.5 | *F* |
| 1 | 3 | -2 | 1.5 | *F* |
| *h* odd*, k* odd | | | | |
| 1 | 3 | 1 | 1.67 | *G* |
| 1 | -3 | -1 | 1.67 | *G* |
| 1 | -1 | 3 | 1.66 | *H* |
| 1 | 1 | -3 | 1.66 | *H* |

**Table A2** Reduced list of triplets in algebraic form derived from Table A.1. The characters labelled with a star (*) refer to the origin fixing structure factors with *C** = *E** = 0°.

| *G* | *+* | *E** | *+* | *C** | = | 0 |
| --- | --- | --- | --- | --- | --- | --- |
| *C** | *+* | *H* | *+* | *E** | = | 0 |
| *D* | *+* | *A* | *+* | *C** | = | 0 |
| *B* | *+* | *E** | *+* | *F* | = | 0 |

**Table A3** Permutation of phases for resolving the ambiguity of the unknown phases *A* and *B* for the structure in Example 1. Since the number of unknown phases is two, four (2^2^) Fourier maps must be calculated with the permuted phases, of which one represents the correct solution.

| Trial | $\alpha_{A}$ | $\alpha_{B}$ |
| --- | --- | --- |
| S1 | 0° | 0° |
| S2 | 0° | 180° |
| S3 | 180° | 0° |
| S4 | 180° | 180° |

**Table A4** The four different sets of phases that are needed for resolving the ambiguity of the unknown phases *A* and *B* for the structure in example 1 (Numbers in the last five columns are in degrees). Each trial set of phases S1 to S4 was used to calculate a Fourier map representing a potential solution (see Figure A1). The last column contains the phase values obtained by calculations based on the model in Figure 3. Comparison of the phase values shows that the correct solution corresponds to phase set S4.

| ***h*** | ***k*** |  | **S1**  *A*=0, *B*=0 | **S2**  *A*=0, *B*=180 | **S3**  *A*=180, *B*=0 | **S4**  *A*=180, *B*=180 | **calculated**  **from model** |
| --- | --- | --- | --- | --- | --- | --- | --- |
| 0 | -4 | *A* | 0 | 0 | 180 | 180 | 180 |
| 0 | 4 | *A* | 0 | 0 | 180 | 180 | 180 |
| 4 | 0 | *B* | 0 | 180 | 0 | 180 | 180 |
| -4 | 0 | *B* | 0 | 180 | 0 | 180 | 180 |
| -2 | 1 | 0 | 0 | 0 | 0 | 0 | 0 |
| 2 | -1 | 0 | 0 | 0 | 0 | 0 | 0 |
| 2 | 3 | *A* | 0 | 0 | 180 | 180 | 180 |
| -2 | -3 | *A* | 0 | 0 | 180 | 180 | 180 |
| -1 | -2 | 0 | 0 | 0 | 0 | 0 | 0 |
| 1 | 2 | 0 | 0 | 0 | 0 | 0 | 0 |
| -3 | 2 | *B* | 0 | 180 | 0 | 180 | 180 |
| 3 | -2 | *B* | 0 | 180 | 0 | 180 | 180 |
| 3 | 1 | 0 | 0 | 0 | 0 | 0 | 0 |
| -3 | -1 | 0 | 0 | 0 | 0 | 0 | 0 |
| -1 | 3 | 0 | 0 | 0 | 0 | 0 | 0 |
| 1 | -3 | 0 | 0 | 0 | 0 | 0 | 0 |

**Table A5** Positional accuracy of the atom peaks in Fourier map S4 (see Figure A1), obtained from 16 normalised structure factors, compared to the underlying model in Figure 3.

| Model Example 1 | | Fourier map S4 | | Difference Model – S4 | | Difference in Å |
| --- | --- | --- | --- | --- | --- | --- |
| *x* | *y* | *x* | *y* | $\Delta x$ | $\Delta y$ |  |
| 0.40812 | 0.84175 | 0.39647 | 0.84619 | 0.01165 | -0.00444 | 0.039 |
| 0.15931 | 0.40838 | 0.15249 | 0.39911 | 0.00682 | 0.00927 | 0.036 |
| 0.84069 | 0.59162 | 0.84487 | 0.60221 | -0.00418 | -0.01059 | 0.035 |
| 0.59188 | 0.15825 | 0.59957 | 0.15645 | -0.00769 | 0.00180 | 0.024 |
