# Supplementary material for: Revisiting symbolic addition: a step-by-step introduction to manual direct methods
Source: Acta Crystallogr E Crystallogr Commun. 2026 Apr 10;82(Pt 5):534–43. doi: 10.1107/S2056989026003300 (PMC13148211; doi:10.1107/S2056989026003300)
Supplement: Supplementary file 3 [file e-82-00534-sup4.docx]

**Appendix C.**

**Table C1** Σ_2_ list of normalised structure factors $E(hk)$ for the projected structure of hexamethylbenzene ordered by *hk* parity. Initially unknown phase values $\alpha(hk)$ are replaced by letters (Note that the characters “I” and “O” were skipped to avoid misinterpretation with numbers). The even-odd and odd-even normalised structure factors marked by a star (*) are used for fixing the origin according to the published structure by Hubig and coworkers. (Hubig *et al.* 2001).

| **Frequency in triplets** | ***h*** | ***k*** | ***E(hk)*** | ***𝛼(hk)*** |
| --- | --- | --- | --- | --- |
| *h even, k even* | | | | |
| 12 | -6 | 4 | 1.65 | *A* |
| 12 | 6 | -4 | 1.65 | *A* |
| 12 | -4 | -2 | 1.59 | *B* |
| 12 | 4 | 2 | 1.59 | *B* |
| 12 | -2 | 6 | 1.44 | *C* |
| 12 | 2 | -6 | 1.44 | *C* |
| 12 | 0 | -4 | 1.4 | *D* |
| 12 | 0 | 4 | 1.4 | *D* |
| *h even, k odd* | | | | |
| 36 | -2 | 7 | 2.85 | *E* |
| 36 | 2 | -7 | 2.85 | *E* |
| 42 | -4 | -3 | 2.67 | *F** |
| 42 | 4 | 3 | 2.67 | *F** |
| 24 | -2 | -9 | 2.66 | *G* |
| 24 | 2 | 9 | 2.66 | *G* |
| 6 | -4 | 1 | 1.49 | *H* |
| 6 | 4 | -1 | 1.49 | *H* |
| 12 | 8 | -1 | 1.35 | *J* |
| 12 | -8 | 1 | 1.35 | *J* |
| 6 | -6 | 7 | 1.23 | *K* |
| 6 | 6 | -7 | 1.23 | *K* |
| 6 | 8 | -5 | 1.17 | *L* |
| 6 | -8 | 5 | 1.17 | *L* |
| 6 | 0 | -1 | 1.08 | *M* |
| 6 | 0 | 1 | 1.08 | *M* |
| *h odd, k even* | | | | |
| 36 | -7 | 4 | 2.86 | *N* |
| 36 | 7 | -4 | 2.86 | *N* |
| 42 | -5 | -2 | 2.61 | *P** |
| 42 | 5 | 2 | 2.61 | *P** |
| 30 | -5 | -6 | 2.13 | *Q* |
| 30 | 5 | 6 | 2.13 | *Q* |
| 6 | -3 | 8 | 1.72 | *R* |
| 6 | 3 | -8 | 1.72 | *R* |
| 12 | -3 | 4 | 1.51 | *S* |
| 12 | 3 | -4 | 1.51 | *S* |
| *h odd, k odd* | | | | |
| 30 | -3 | 7 | 2.55 | *T* |
| 30 | 3 | -7 | 2.55 | *T* |
| 30 | -7 | 5 | 2.49 | *U* |
| 30 | 7 | -5 | 2.49 | *U* |
| 24 | 7 | -1 | 2.04 | *V* |
| 24 | -7 | 1 | 2.04 | *V* |
| 36 | -1 | -3 | 1.83 | *W* |
| 36 | 1 | 3 | 1.83 | *W* |
| 6 | -5 | -3 | 1.52 | *X* |
| 6 | 5 | 3 | 1.52 | *X* |
| 6 | -3 | 3 | 1.1 | *Y* |
| 6 | 3 | -3 | 1.1 | *Y* |
| 6 | -1 | 1 | 1.09 | *Z* |
| 6 | 1 | -1 | 1.09 | *Z* |

**Table C2** Reduced list of triplets in algebraic form derived from Table C.1. The characters labelled with a star (*) refer to the origin fixing structure factors with assigned values *F** = 180° and *P** = 180 °, respectively.

*F* + P* + Z* = 0

*F* + N + T* = 0

*A + E + F** = 0

*C + F* + G* = 0

*F* + Q + W* = 0

*F* + R + U* = 0

*F* + S + V* = 0

*D + P* + Q* = 0

*C + N + P** = 0

*E + P* + U* = 0

*G + P* + T* = 0

*H + P* + W* = 0

*L + P* + T* = 0

*A + V + W* = 0

*B + E + G* = 0

*B + T + U* = 0

*D + U + V* = 0

*E + N + X* = 0

*E + Q + V* = 0

*E + S + W* = 0

*G + Q + Y* = 0

*J + N + W* = 0

*J + Q + T* = 0

*K + N + W* = 0

*M + N + U* = 0

**Table C3** Permutation of phases for resolving the ambiguity of the unknown phases *A*, *C*, *D*, and *S* for the structure of hexamethylbenzene.

| Trial | $\alpha_{A}$ | $\alpha_{C}$ | $\alpha_{D}$ | $\alpha_{S}$ |
| --- | --- | --- | --- | --- |
| S1 | 0° | 0° | 0° | 0° |
| S2 | 0° | 0° | 0° | 180° |
| S3 | 0° | 0° | 180° | 0° |
| S4 | 0° | 180° | 0° | 0° |
| S5 | 180° | 0° | 0° | 0° |
| S6 | 0° | 0° | 180° | 180° |
| S7 | 180° | 180° | 0° | 0° |
| S8 | 180° | 0° | 180° | 0° |
| S9 | 0° | 180° | 0° | 180° |
| S10 | 180° | 0° | 0° | 180° |
| S11 | 0° | 180° | 180° | 0° |
| S12 | 0° | 180° | 180° | 180° |
| S13 | 180° | 0° | 180° | 180° |
| S14 | 180° | 180° | 0° | 180° |
| S15 | 180° | 180° | 180° | 0° |
| S16 | 180° | 180° | 180° | 180° |

**Table C4** The 16 different sets of phases that are required for resolving the ambiguity of the unknown phases *A, C, D*, and *S* for the structure of hexamethlybenzene. Each trial set of phases S1 to S16 was used to calculate a Fourier map representing a potential solution (see Figure C1). The last column contains the phase values obtained by calculations based on the model in Figure 6. Comparison of the phase values shows that the correct solution is phase set S3.

| ***h*** | ***k*** |  | **S1**  *A*=0 *C*=0 D=0 S=0 | **S2**  *A*=0 *C*=0 D=0 S=180 | **S3**  *A*=0 *C*=0 D=180 S=0 | **S4**  *A*=0 *C*=180 D=0 S=0 | **S5**  *A*=180 *C*=0 D=0 S=0 | **S6**  *A*=0 *C*=0 D=180 S=180 | **S7**  *A*=180 *C*=180 D=0 S=0 | **S8**  *A*=180 *C*=0 D=180 S=0 | **calculated from model** |
| --- | --- | --- | --- | --- | --- | --- | --- | --- | --- | --- | --- |
| -6 | 4 | A | 0 | 0 | 0 | 0 | 180 | 0 | 180 | 180 | 0 |
| 6 | -4 | A | 0 | 0 | 0 | 0 | 180 | 0 | 180 | 180 | 0 |
| -4 | -2 | A + C | 0 | 0 | 0 | 180 | 180 | 0 | 0 | 180 | 0 |
| 4 | 2 | A + C | 0 | 0 | 0 | 180 | 180 | 0 | 0 | 180 | 0 |
| -2 | 6 | C | 0 | 0 | 0 | 180 | 0 | 0 | 180 | 0 | 0 |
| 2 | -6 | C | 0 | 0 | 0 | 180 | 0 | 0 | 180 | 0 | 0 |
| 0 | -4 | D | 0 | 0 | 180 | 0 | 0 | 180 | 0 | 180 | 180 |
| 0 | 4 | D | 0 | 0 | 180 | 0 | 0 | 180 | 0 | 180 | 180 |
| -2 | 7 | 180 - A | 180 | 180 | 180 | 180 | 0 | 180 | 0 | 0 | 180 |
| 2 | -7 | 180 - A | 180 | 180 | 180 | 180 | 0 | 180 | 0 | 0 | 180 |
| -4 | -3 | 180 | 180 | 180 | 180 | 180 | 180 | 180 | 180 | 180 | 180 |
| 4 | 3 | 180 | 180 | 180 | 180 | 180 | 180 | 180 | 180 | 180 | 180 |
| -2 | -9 | 180 - C | 180 | 180 | 180 | 0 | 180 | 180 | 0 | 180 | 180 |
| 2 | 9 | 180 - C | 180 | 180 | 180 | 0 | 180 | 180 | 0 | 180 | 180 |
| -4 | 1 | 180 - D | 180 | 180 | 0 | 180 | 180 | 0 | 180 | 0 | 0 |
| 4 | -1 | 180 - D | 180 | 180 | 0 | 180 | 180 | 0 | 180 | 0 | 0 |
| 8 | -1 | 180 + D - C | 180 | 180 | 0 | 0 | 180 | 0 | 0 | 0 | 0 |
| -8 | 1 | 180 + D - C | 180 | 180 | 0 | 0 | 180 | 0 | 0 | 0 | 0 |
| -6 | 7 | 180 + C - D | 180 | 180 | 0 | 0 | 180 | 0 | 0 | 0 | 0 |
| 6 | -7 | 180 + C - D | 180 | 180 | 0 | 0 | 180 | 0 | 0 | 0 | 0 |
| 8 | -5 | 180 - C | 180 | 180 | 180 | 0 | 180 | 180 | 0 | 180 | 180 |
| -8 | 5 | 180 - C | 180 | 180 | 180 | 0 | 180 | 180 | 0 | 180 | 180 |
| 0 | -1 | 180 + C - A | 180 | 180 | 180 | 0 | 0 | 180 | 180 | 0 | 180 |
| 0 | 1 | 180 + C - A | 180 | 180 | 180 | 0 | 0 | 180 | 180 | 0 | 180 |
| -7 | 4 | 180 - C | 180 | 180 | 180 | 0 | 180 | 180 | 0 | 180 | 180 |
| 7 | -4 | 180 - C | 180 | 180 | 180 | 0 | 180 | 180 | 0 | 180 | 180 |
| -5 | -2 | 180 | 180 | 180 | 180 | 180 | 180 | 180 | 180 | 180 | 180 |
| 5 | 2 | 180 | 180 | 180 | 180 | 180 | 180 | 180 | 180 | 180 | 180 |
| -5 | -6 | 180 - D | 180 | 180 | 0 | 180 | 180 | 0 | 180 | 0 | 0 |
| 5 | 6 | 180 - D | 180 | 180 | 0 | 180 | 180 | 0 | 180 | 0 | 0 |
| -3 | 8 | 180 - A | 180 | 180 | 180 | 180 | 0 | 180 | 0 | 0 | 180 |
| 3 | -8 | 180 - A | 180 | 180 | 180 | 180 | 0 | 180 | 0 | 0 | 180 |
| -3 | 4 | S | 0 | 180 | 0 | 0 | 0 | 180 | 0 | 0 | 0 |
| 3 | -4 | S | 0 | 180 | 0 | 0 | 0 | 180 | 0 | 0 | 0 |
| -3 | 7 | C | 0 | 0 | 0 | 180 | 0 | 0 | 180 | 0 | 0 |
| 3 | -7 | C | 0 | 0 | 0 | 180 | 0 | 0 | 180 | 0 | 0 |
| -7 | 5 | A | 0 | 0 | 0 | 0 | 180 | 0 | 180 | 180 | 0 |
| 7 | -5 | A | 0 | 0 | 0 | 0 | 180 | 0 | 180 | 180 | 0 |
| 7 | -1 | 180 - S | 180 | 0 | 180 | 180 | 180 | 0 | 180 | 180 | 180 |
| -7 | 1 | 180 - S | 180 | 0 | 180 | 180 | 180 | 0 | 180 | 180 | 180 |
| -1 | -3 | D | 0 | 0 | 180 | 0 | 0 | 180 | 0 | 180 | 180 |
| 1 | 3 | D | 0 | 0 | 180 | 0 | 0 | 180 | 0 | 180 | 180 |
| -5 | -3 | A + C | 0 | 0 | 0 | 180 | 180 | 0 | 0 | 180 | 0 |
| 5 | 3 | A + C | 0 | 0 | 0 | 180 | 180 | 0 | 0 | 180 | 0 |
| -3 | 3 | C + D | 0 | 0 | 180 | 180 | 0 | 180 | 180 | 180 | 180 |
| 3 | -3 | C + D | 0 | 0 | 180 | 180 | 0 | 180 | 180 | 180 | 180 |
| -1 | 1 | 0 | 0 | 0 | 0 | 0 | 0 | 0 | 0 | 0 | 0 |
| 1 | -1 | 0 | 0 | 0 | 0 | 0 | 0 | 0 | 0 | 0 | 0 |

| ***h*** | ***k*** |  | **S9**  *A*=0 *C*=180 D=0 S=180 | **S10**  *A*=180 *C*=0 D=0 S=180 | **S11**  *A*=0 *C*=180 D=180 S=0 | **S12**  *A*=0 *C*=180 D=180 S=180 | **S13**  *A*=180 *C*=0 D=180 S=180 | **S14**  *A*=180 *C*=180 D=0 S=180 | **S15**  *A*=180 *C*=180 D=180 S=0 | **S16**  *A*=180 *C*=180 D=180 S=180 | **calculated from model** |
| --- | --- | --- | --- | --- | --- | --- | --- | --- | --- | --- | --- |
| -6 | 4 | A | 0 | 180 | 0 | 0 | 180 | 180 | 180 | 180 | 0 |
| 6 | -4 | A | 0 | 180 | 0 | 0 | 180 | 180 | 180 | 180 | 0 |
| -4 | -2 | A + C | 180 | 180 | 180 | 180 | 180 | 0 | 0 | 0 | 0 |
| 4 | 2 | A + C | 180 | 180 | 180 | 180 | 180 | 0 | 0 | 0 | 0 |
| -2 | 6 | C | 180 | 0 | 180 | 180 | 0 | 180 | 180 | 180 | 0 |
| 2 | -6 | C | 180 | 0 | 180 | 180 | 0 | 180 | 180 | 180 | 0 |
| 0 | -4 | D | 0 | 0 | 180 | 180 | 180 | 0 | 180 | 180 | 180 |
| 0 | 4 | D | 0 | 0 | 180 | 180 | 180 | 0 | 180 | 180 | 180 |
| -2 | 7 | 180 - A | 180 | 0 | 180 | 180 | 0 | 0 | 0 | 0 | 180 |
| 2 | -7 | 180 - A | 180 | 0 | 180 | 180 | 0 | 0 | 0 | 0 | 180 |
| -4 | -3 | 180 | 180 | 180 | 180 | 180 | 180 | 180 | 180 | 180 | 180 |
| 4 | 3 | 180 | 180 | 180 | 180 | 180 | 180 | 180 | 180 | 180 | 180 |
| -2 | -9 | 180 - C | 0 | 180 | 0 | 0 | 180 | 0 | 0 | 0 | 180 |
| 2 | 9 | 180 - C | 0 | 180 | 0 | 0 | 180 | 0 | 0 | 0 | 180 |
| -4 | 1 | 180 - D | 180 | 180 | 0 | 0 | 0 | 180 | 0 | 0 | 0 |
| 4 | -1 | 180 - D | 180 | 180 | 0 | 0 | 0 | 180 | 0 | 0 | 0 |
| 8 | -1 | 180 + D - C | 0 | 180 | 180 | 180 | 0 | 0 | 180 | 180 | 0 |
| -8 | 1 | 180 + D - C | 0 | 180 | 180 | 180 | 0 | 0 | 180 | 180 | 0 |
| -6 | 7 | 180 + C - D | 0 | 180 | 180 | 180 | 0 | 0 | 180 | 180 | 0 |
| 6 | -7 | 180 + C - D | 0 | 180 | 180 | 180 | 0 | 0 | 180 | 180 | 0 |
| 8 | -5 | 180 - C | 0 | 180 | 0 | 0 | 180 | 0 | 0 | 0 | 180 |
| -8 | 5 | 180 - C | 0 | 180 | 0 | 0 | 180 | 0 | 0 | 0 | 180 |
| 0 | -1 | 180 + C - A | 0 | 0 | 0 | 0 | 0 | 180 | 180 | 180 | 180 |
| 0 | 1 | 180 + C - A | 0 | 0 | 0 | 0 | 0 | 180 | 180 | 180 | 180 |
| -7 | 4 | 180 - C | 0 | 180 | 0 | 0 | 180 | 0 | 0 | 0 | 180 |
| 7 | -4 | 180 - C | 0 | 180 | 0 | 0 | 180 | 0 | 0 | 0 | 180 |
| -5 | -2 | 180 | 180 | 180 | 180 | 180 | 180 | 180 | 180 | 180 | 180 |
| 5 | 2 | 180 | 180 | 180 | 180 | 180 | 180 | 180 | 180 | 180 | 180 |
| -5 | -6 | 180 - D | 180 | 180 | 0 | 0 | 0 | 180 | 0 | 0 | 0 |
| 5 | 6 | 180 - D | 180 | 180 | 0 | 0 | 0 | 180 | 0 | 0 | 0 |
| -3 | 8 | 180 - A | 180 | 0 | 180 | 180 | 0 | 0 | 0 | 0 | 180 |
| 3 | -8 | 180 - A | 180 | 0 | 180 | 180 | 0 | 0 | 0 | 0 | 180 |
| -3 | 4 | S | 180 | 180 | 0 | 180 | 180 | 180 | 0 | 180 | 0 |
| 3 | -4 | S | 180 | 180 | 0 | 180 | 180 | 180 | 0 | 180 | 0 |
| -3 | 7 | C | 180 | 0 | 180 | 180 | 0 | 180 | 180 | 180 | 0 |
| 3 | -7 | C | 180 | 0 | 180 | 180 | 0 | 180 | 180 | 180 | 0 |
| -7 | 5 | A | 0 | 180 | 0 | 0 | 180 | 180 | 180 | 180 | 0 |
| 7 | -5 | A | 0 | 180 | 0 | 0 | 180 | 180 | 180 | 180 | 0 |
| 7 | -1 | 180 - S | 0 | 0 | 180 | 0 | 0 | 0 | 180 | 0 | 180 |
| -7 | 1 | 180 - S | 0 | 0 | 180 | 0 | 0 | 0 | 180 | 0 | 180 |
| -1 | -3 | D | 0 | 0 | 180 | 180 | 180 | 0 | 180 | 180 | 180 |
| 1 | 3 | D | 0 | 0 | 180 | 180 | 180 | 0 | 180 | 180 | 180 |
| -5 | -3 | A + C | 180 | 180 | 180 | 180 | 180 | 0 | 0 | 0 | 0 |
| 5 | 3 | A + C | 180 | 180 | 180 | 180 | 180 | 0 | 0 | 0 | 0 |
| -3 | 3 | C + D | 180 | 0 | 0 | 0 | 180 | 180 | 0 | 0 | 180 |
| 3 | -3 | C + D | 180 | 0 | 0 | 0 | 180 | 180 | 0 | 0 | 180 |
| -1 | 1 | 0 | 0 | 0 | 0 | 0 | 0 | 0 | 0 | 0 | 0 |
| 1 | -1 | 0 | 0 | 0 | 0 | 0 | 0 | 0 | 0 | 0 | 0 |

**Figure C1** Fourier maps of the 16 potential solutions for the two-dimensional model structure of hexamethylbenzene. The maps were calculated with the $E(hk)$ values and phases listed in table C3. Comparison with the structural model in Figure 6 shows that Fourier map S3 is the correct solution.


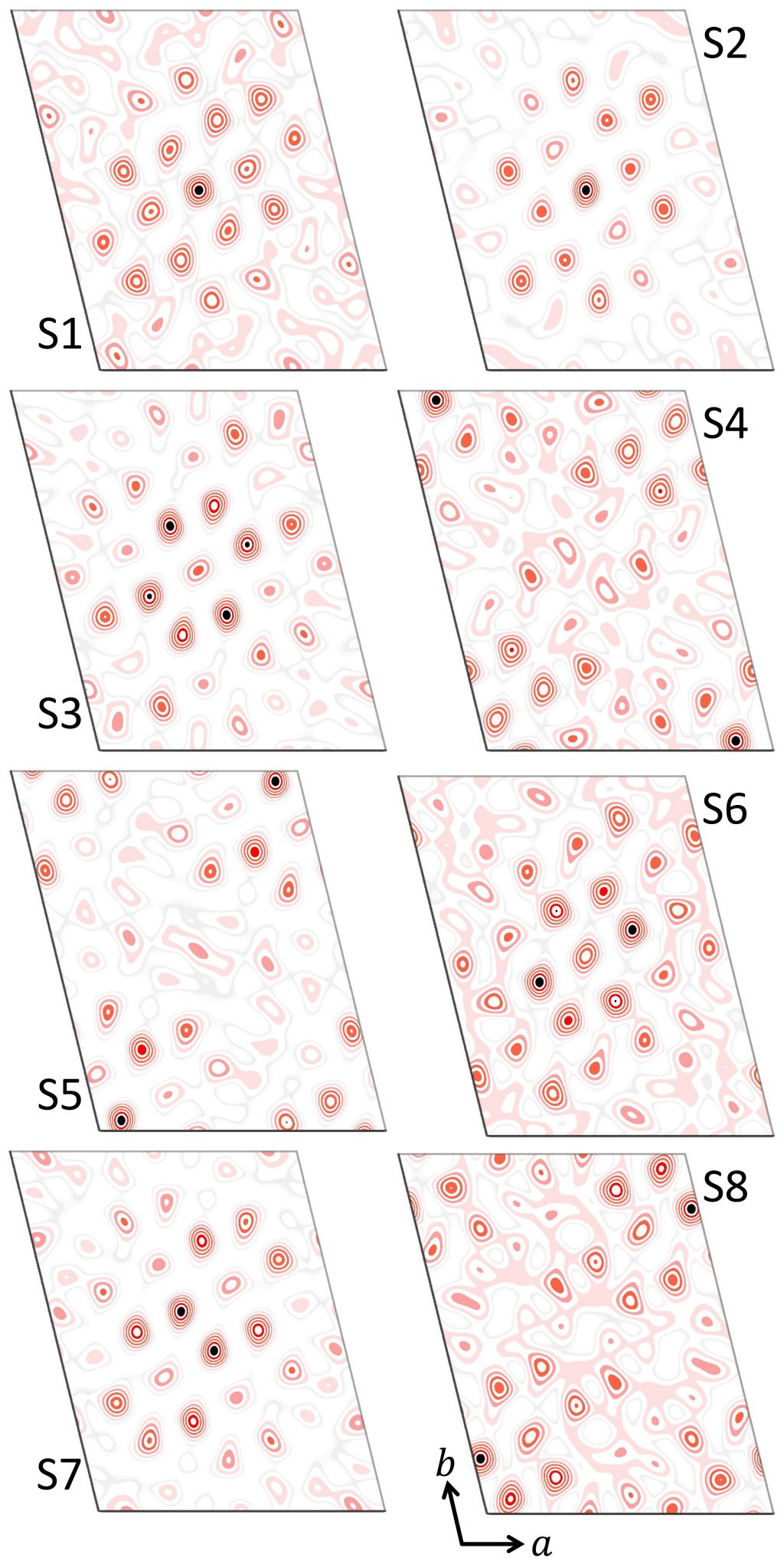


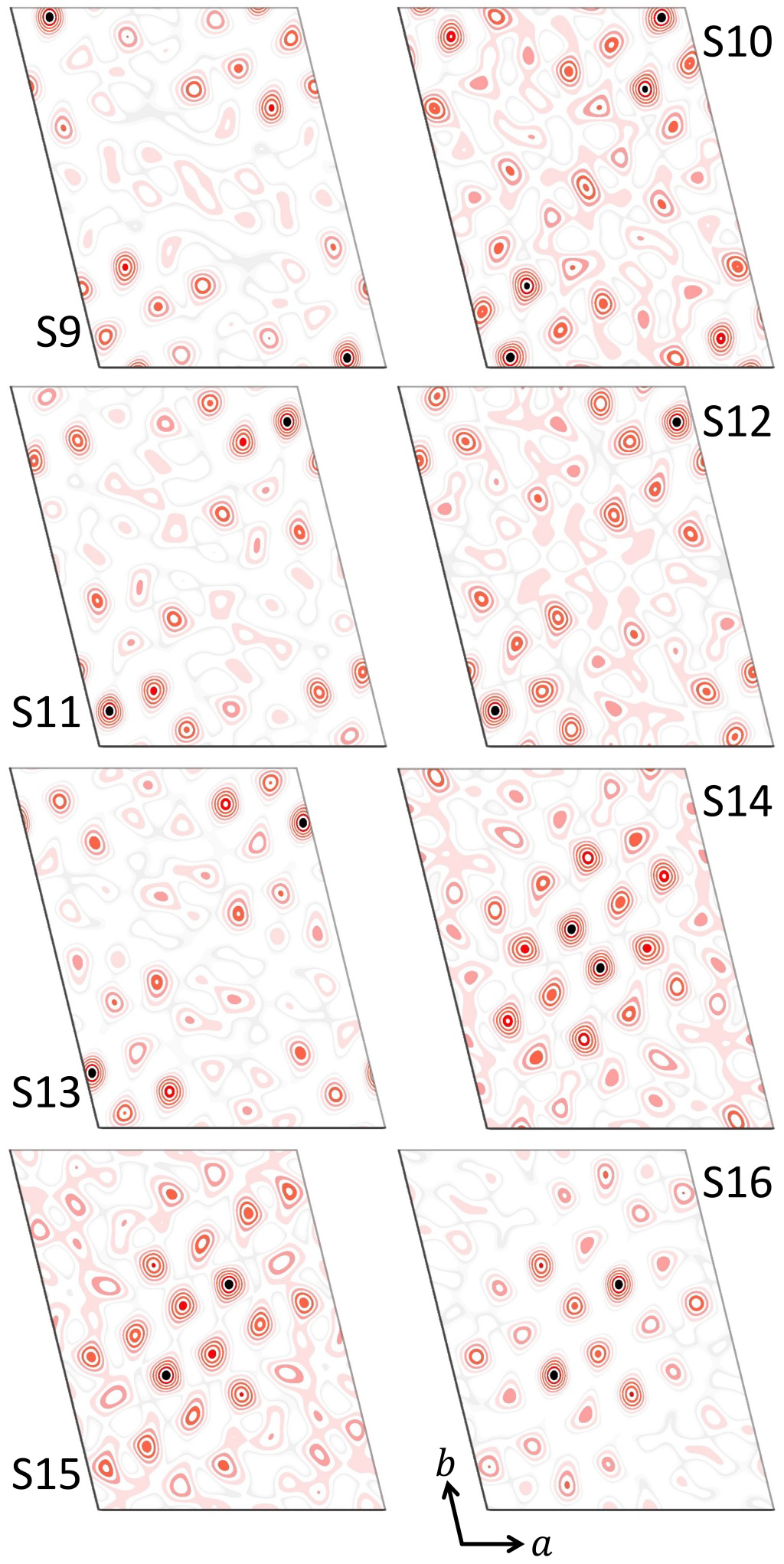


**Table C5** Positional accuracy of the carbon atom peaks in Fourier map S3 (see Figure C1), obtained from 48 normalised structure factors, compared with the projected model in Figure 6 obtained from single crystal X-ray diffraction (XRD).

| XRD single crystal | | Fourier map S3 | | Difference Model – S3 | | Difference in Å |
| --- | --- | --- | --- | --- | --- | --- |
| *x* | *y* | *x* | *y* | $\Delta x$ | $\Delta y$ |  |
| 0.38488 | 0.31920 | 0.39193 | 0.31980 | -0.00705 | -0.00060 | 0.044 |
| 0.61512 | 0.68080 | 0.60938 | 0.67679 | 0.00575 | 0.00401 | 0.048 |
| 0.57005 | 0.38614 | 0.55862 | 0.37363 | 0.01143 | 0.01251 | 0.123 |
| 0.42995 | 0.61386 | 0.44271 | 0.62493 | -0.01276 | -0.01107 | 0.119 |
| 0.68300 | 0.56753 | 0.69271 | 0.57113 | -0.00971 | -0.00360 | 0.067 |
| 0.31700 | 0.43247 | 0.30864 | 0.42934 | 0.00836 | 0.00313 | 0.058 |
| 0.25416 | 0.12605 | 0.25524 | 0.11833 | -0.00108 | 0.00772 | 0.062 |
| 0.74584 | 0.87395 | 0.74479 | 0.87724 | 0.00105 | -0.00329 | 0.027 |
| 0.65172 | 0.26423 | 0.64714 | 0.26553 | 0.00458 | -0.00129 | 0.030 |
| 0.34828 | 0.73577 | 0.35156 | 0.73299 | -0.00328 | 0.00278 | 0.030 |
| 0.87237 | 0.63866 | 0.86589 | 0.62908 | 0.00648 | 0.00958 | 0.087 |
| 0.12763 | 0.36134 | 0.13802 | 0.36967 | -0.01039 | -0.00833 | 0.093 |
