# Supplementary figures and images for: Revisiting symbolic addition: a step-by-step introduction to manual direct methods (part 1 of 2)
Source: Acta Crystallogr E Crystallogr Commun. 2026 Apr 10;82(Pt 5):534–43. doi: 10.1107/S2056989026003300 (PMC13148211; doi:10.1107/S2056989026003300)

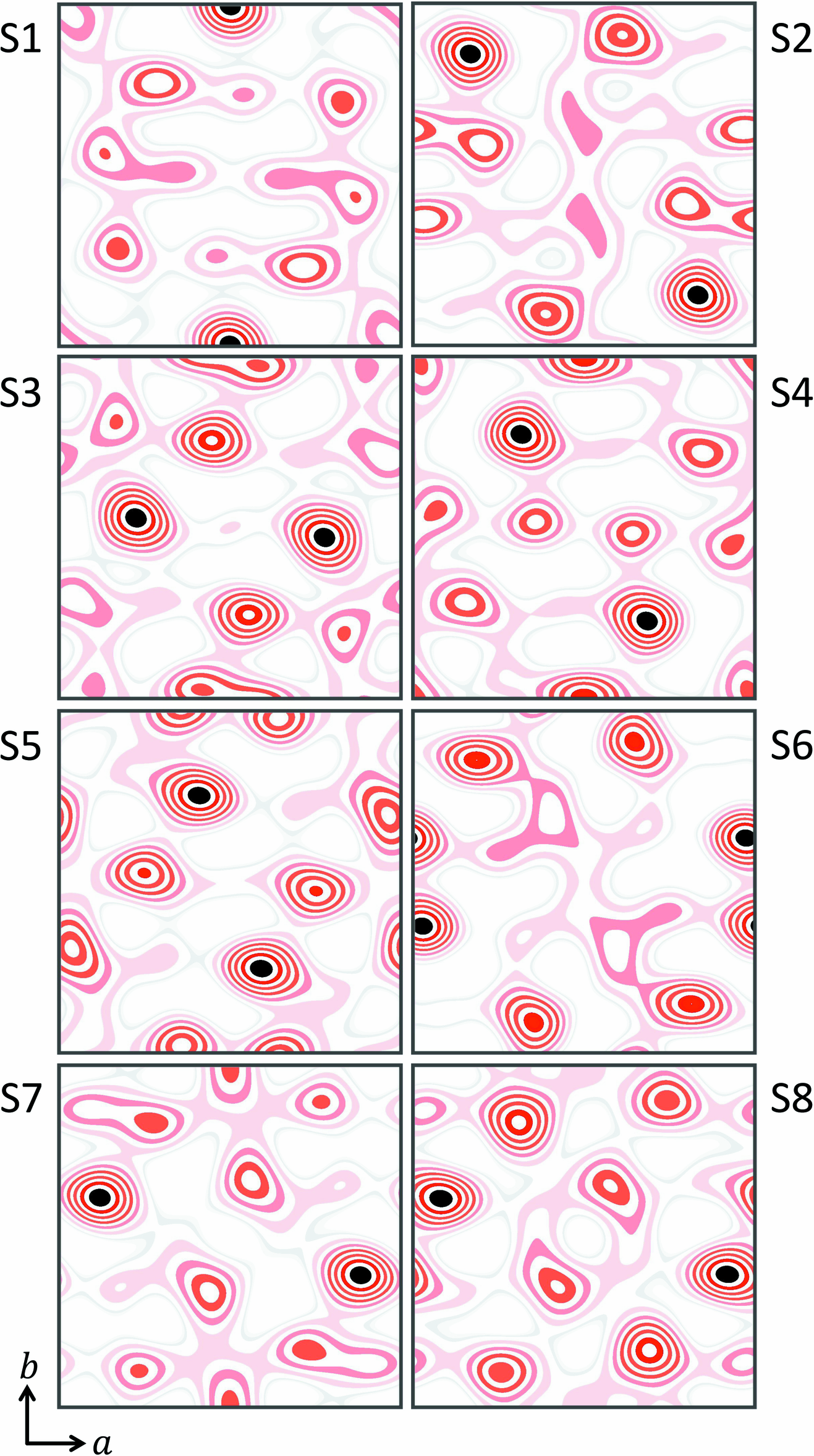

Supplement: Supplementary file 4 [file e-82-00534-figA1sup5.tif]

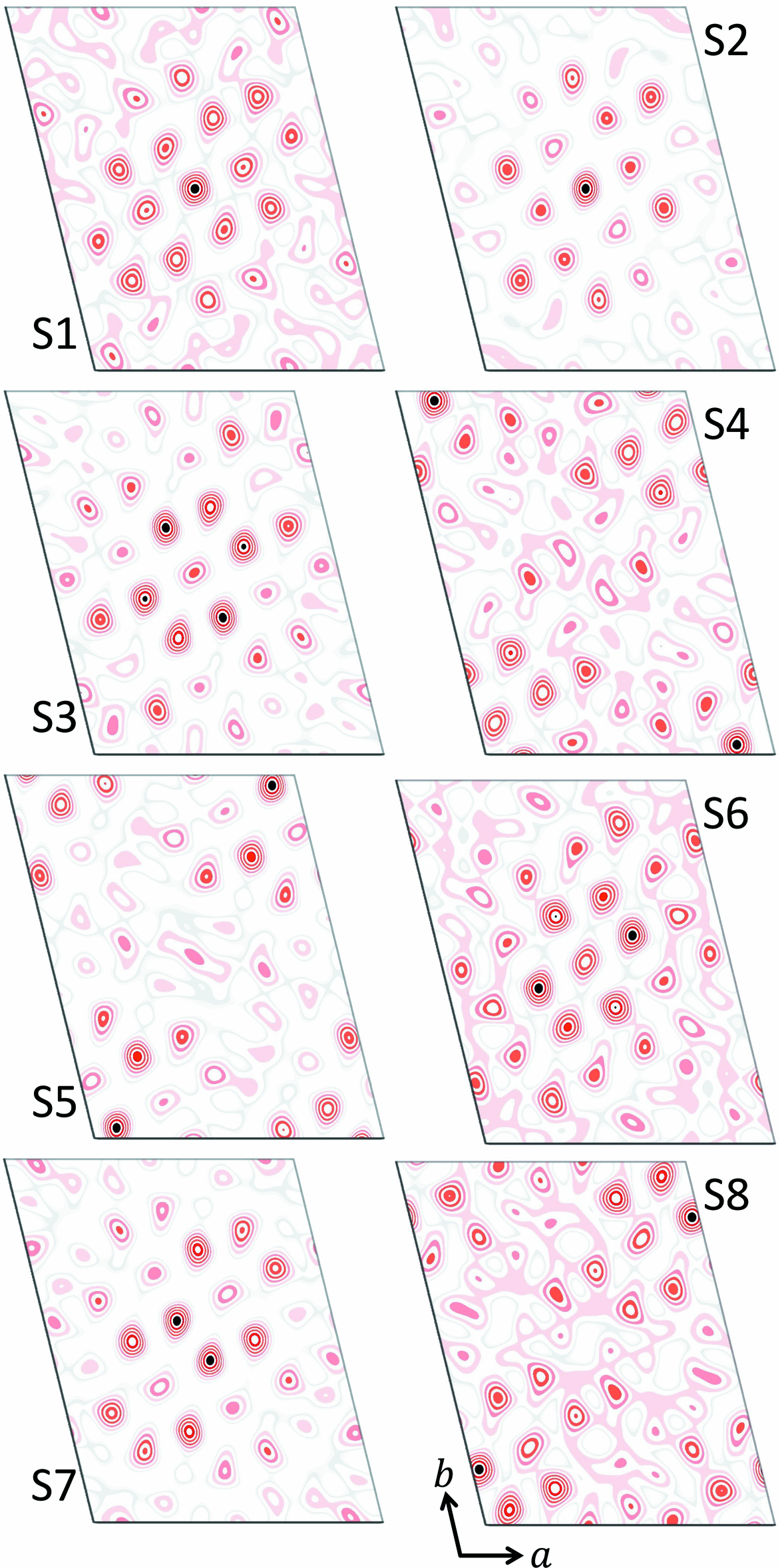

Supplement: Supplementary file 5 [file e-82-00534-figC1asup6.tif]

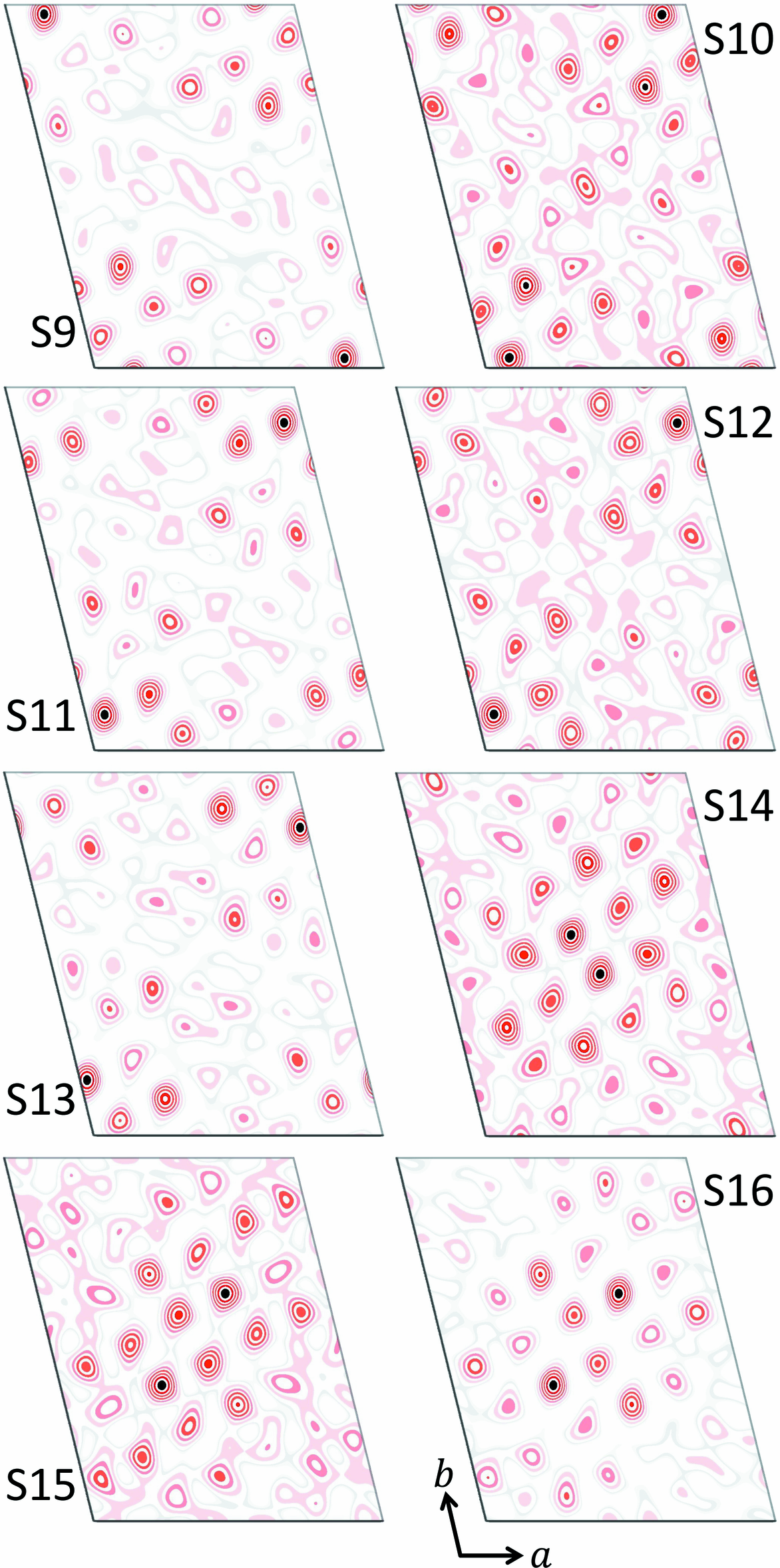

Supplement: Supplementary file 6 [file e-82-00534-figC1bsup7.tif]

## Slide 1
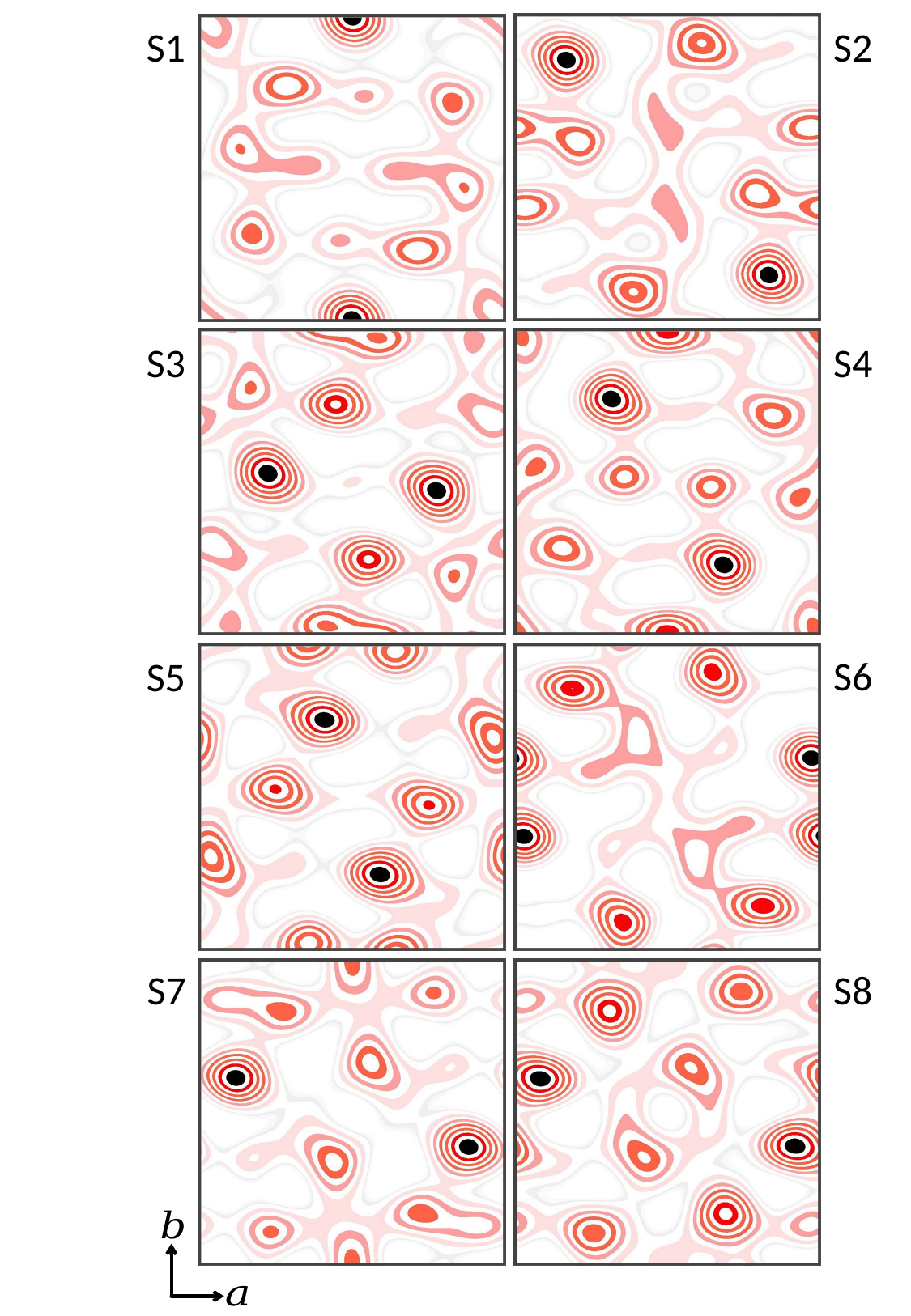

S1
S2
S4
S3
S6
S5
S7
S8

Supplement: Supplementary file 7 [file e-82-00534-sup8.zip › oi2035_SupportingMaterial/Example2/Example2_Fou_maps.pptx]

S1

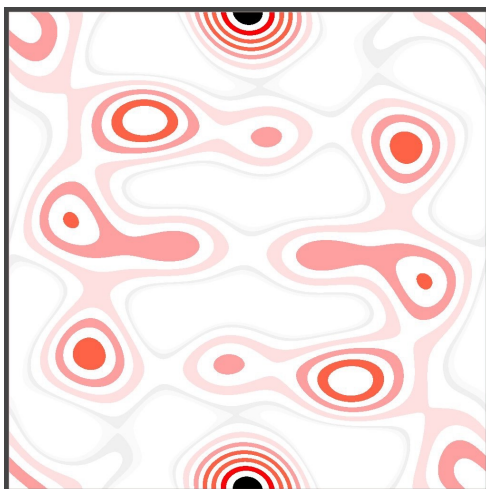

S2

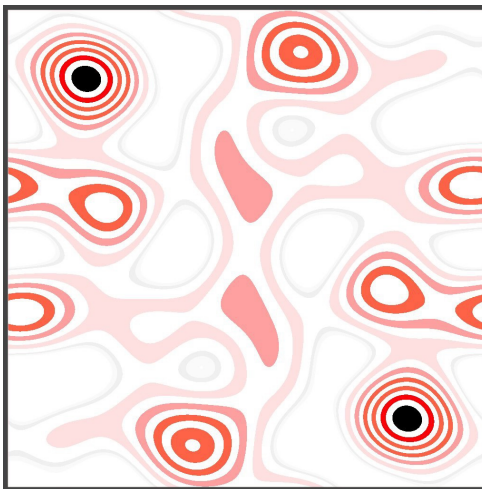

S3

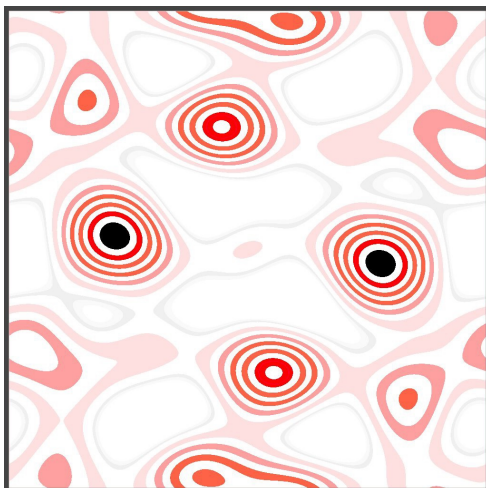

S4

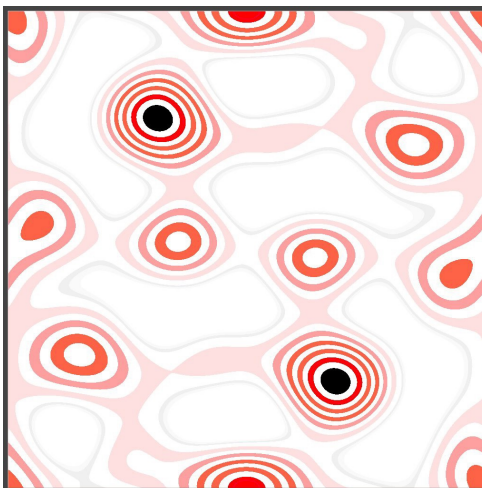

S5

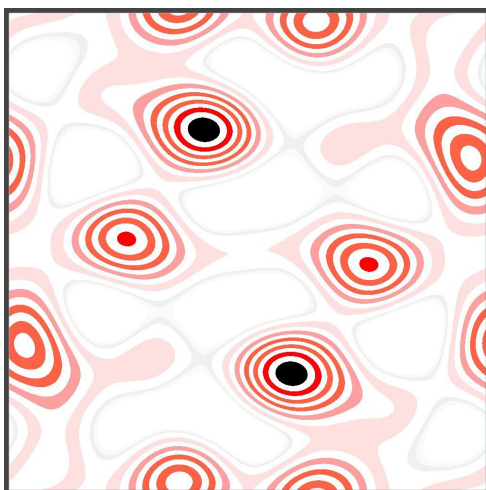

S6

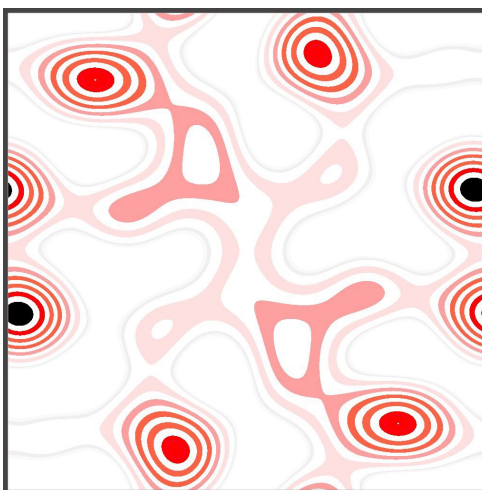

S7

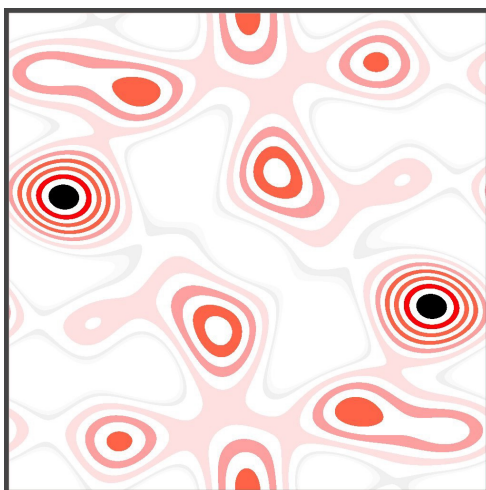

S8

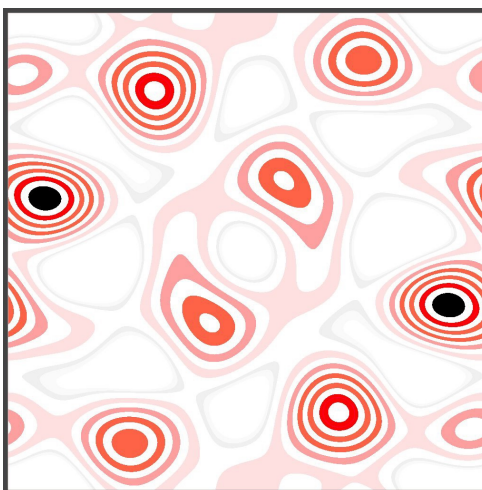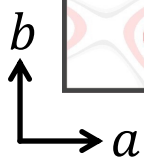

Supplement: Supplementary file 7 [file e-82-00534-sup8.zip › oi2035_SupportingMaterial/Example2/Example2_Fou_maps.pdf]

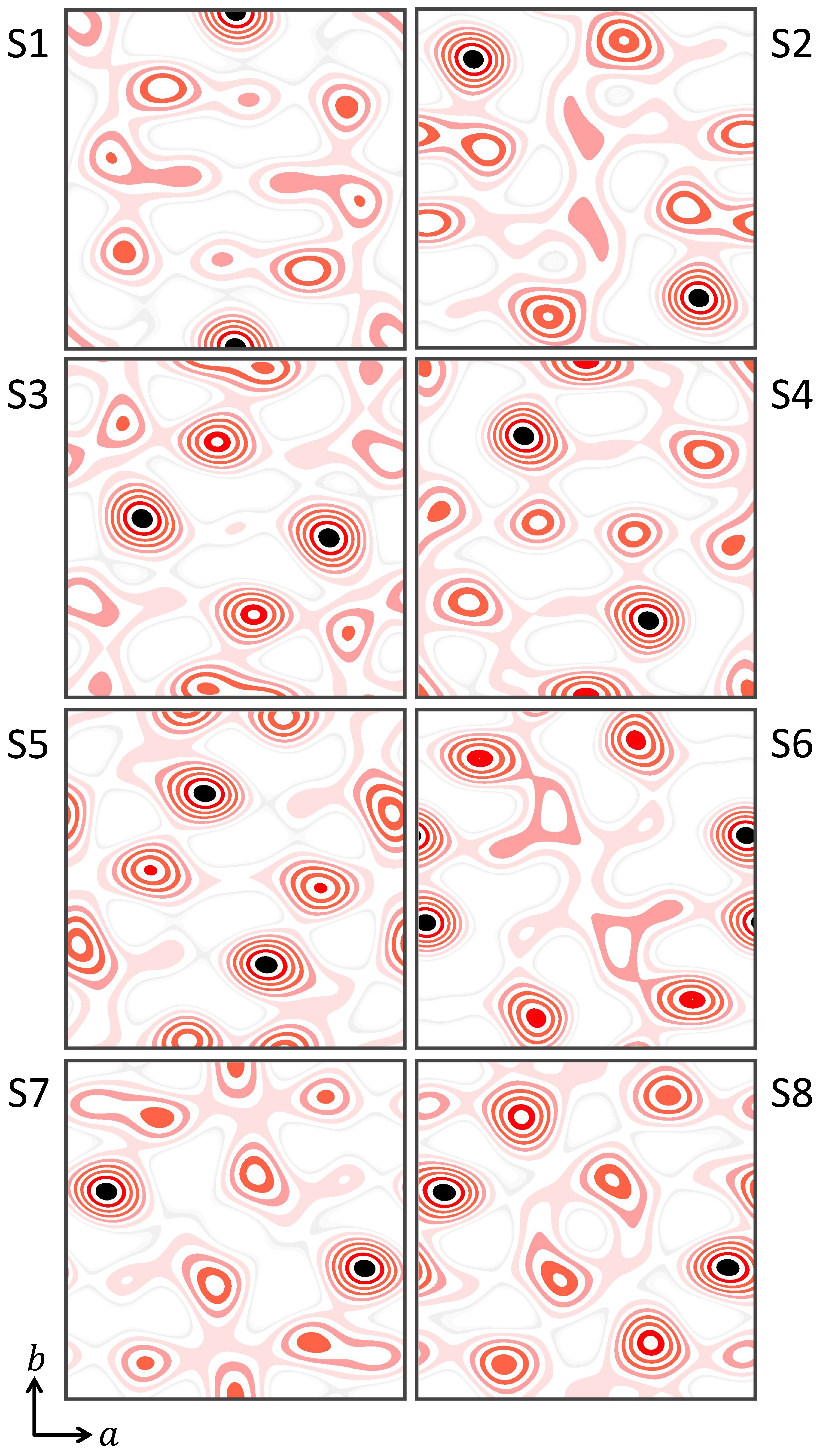

Supplement: Supplementary file 7 [file e-82-00534-sup8.zip › oi2035_SupportingMaterial/Example2/Example2_Fou_maps.jpg]

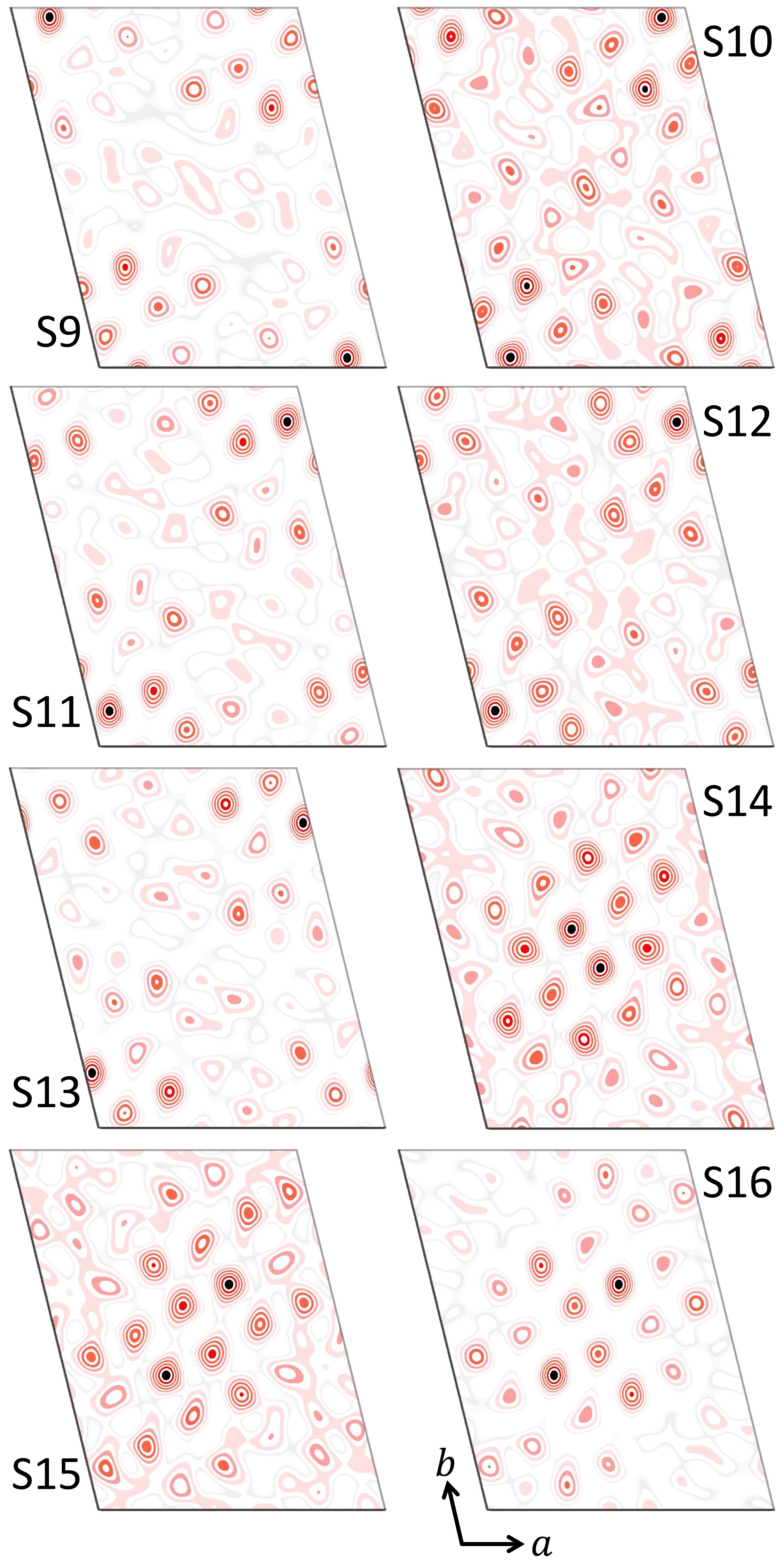

Supplement: Supplementary file 7 [file e-82-00534-sup8.zip › oi2035_SupportingMaterial/Example3/Example3_Fou_maps2.jpg]

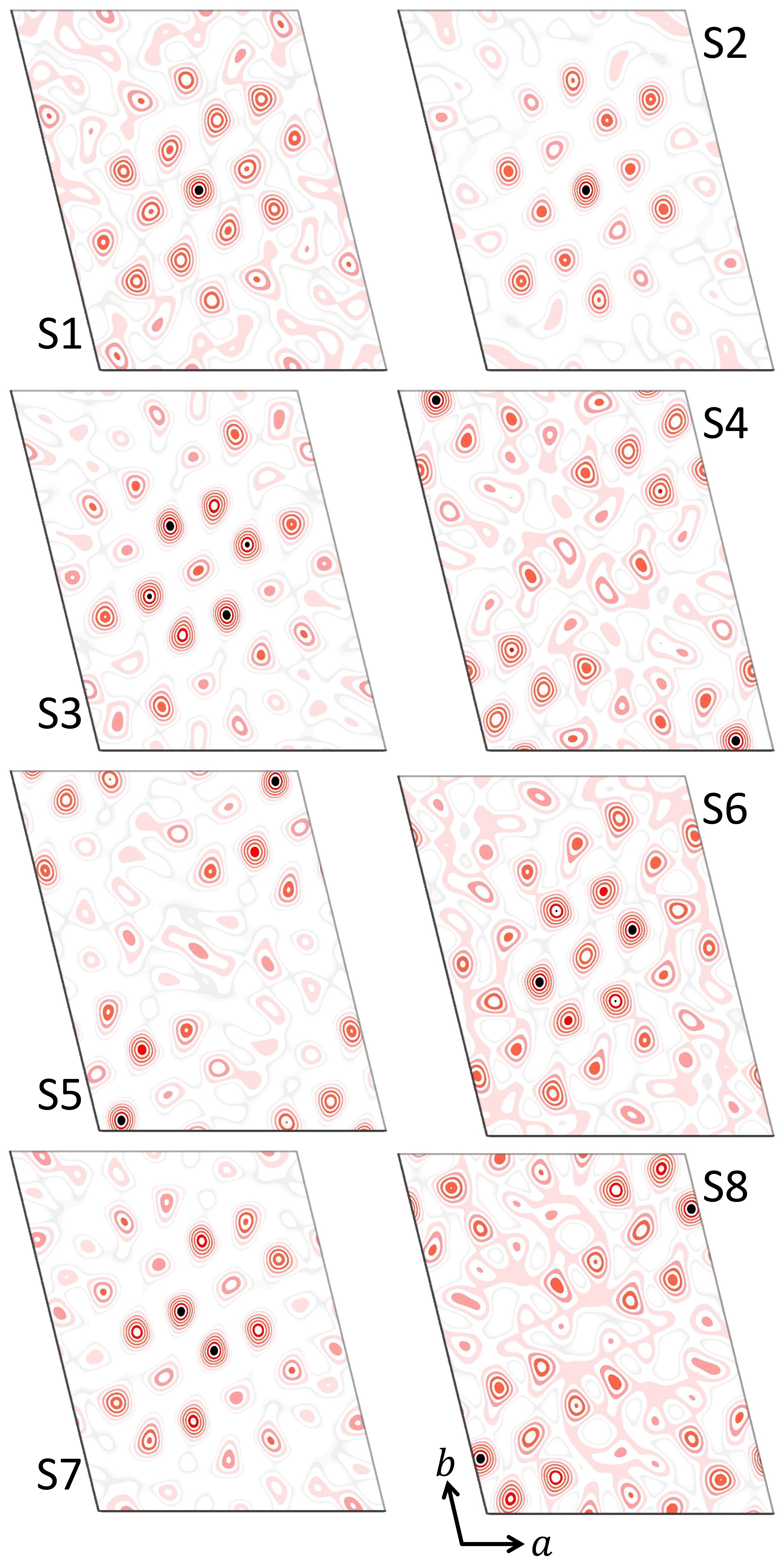

Supplement: Supplementary file 7 [file e-82-00534-sup8.zip › oi2035_SupportingMaterial/Example3/Example3_Fou_maps1.jpg]

## Slide 1
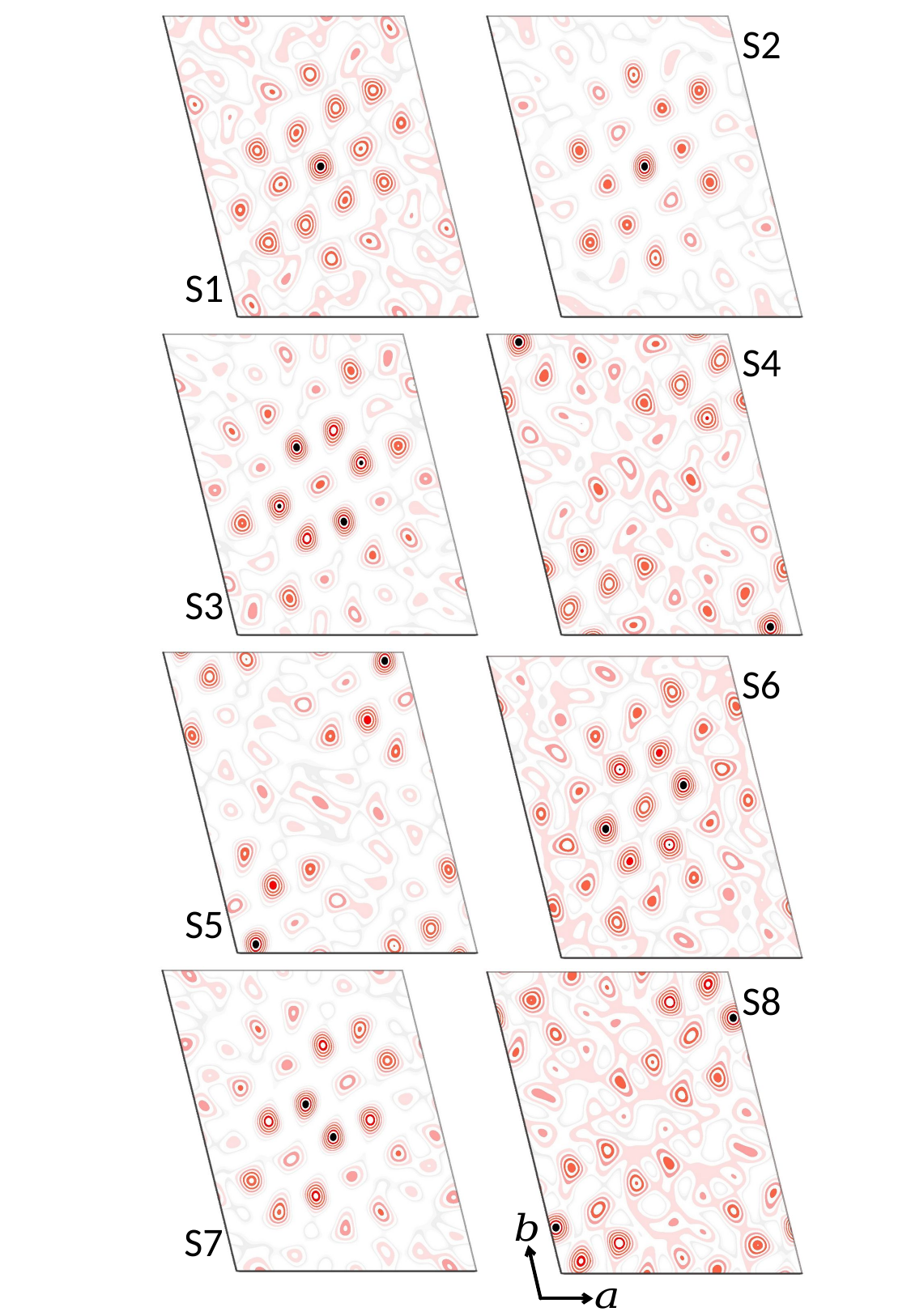

S2
S1
S4
S3
S6
S5
S8
S7

## Slide 2
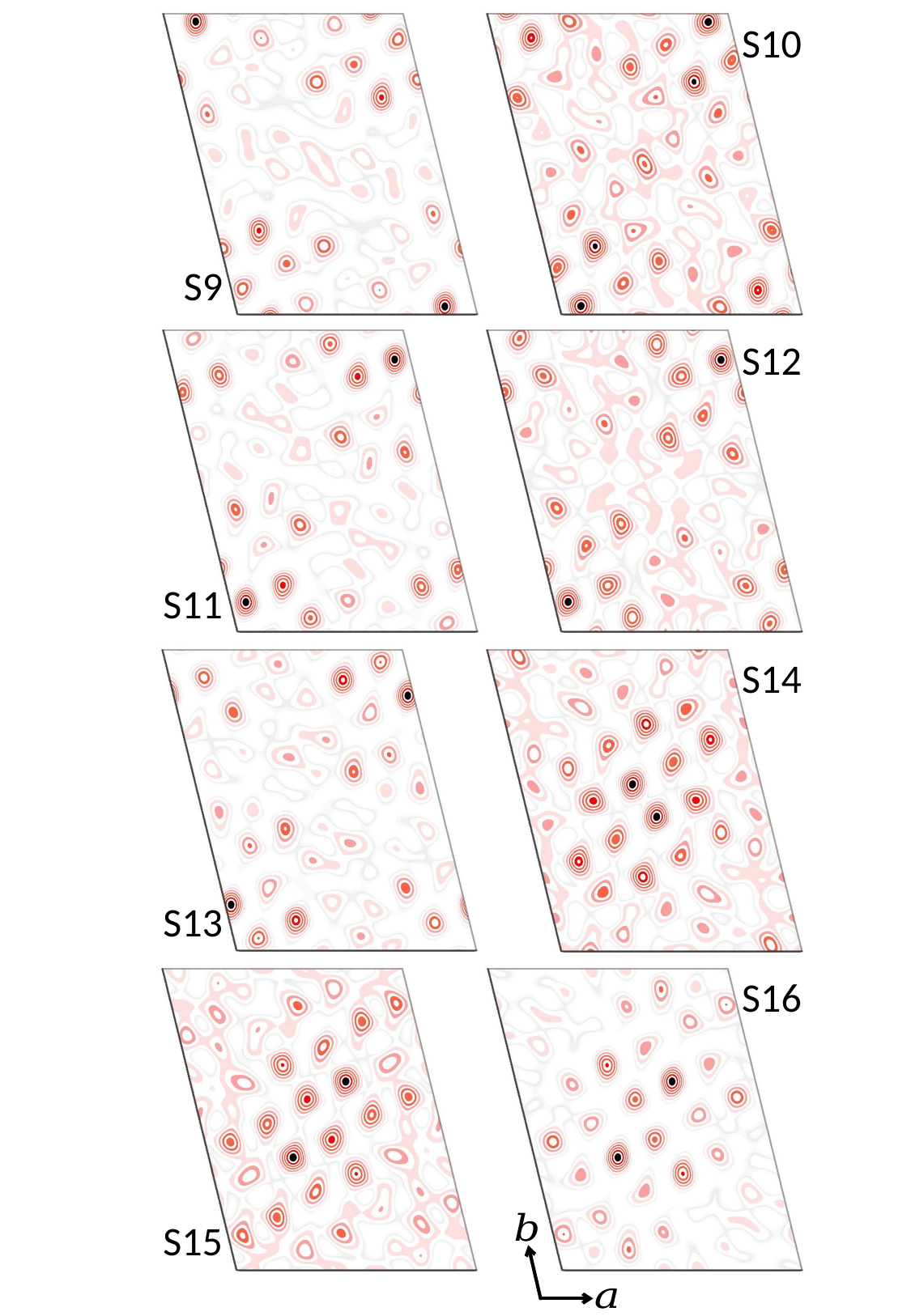

S10
S9
S12
S11
S14
S13
S16
S15

Supplement: Supplementary file 7 [file e-82-00534-sup8.zip › oi2035_SupportingMaterial/Example3/Example3_Fou_maps.pptx]

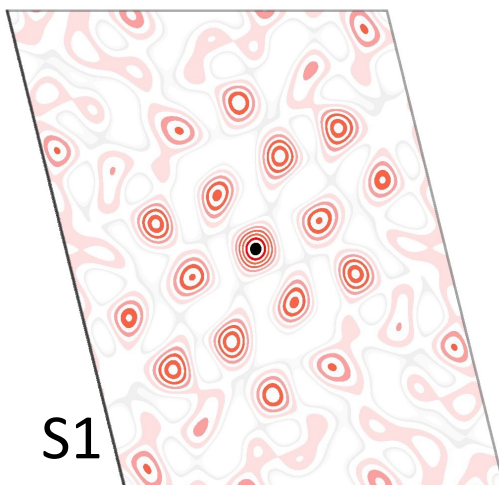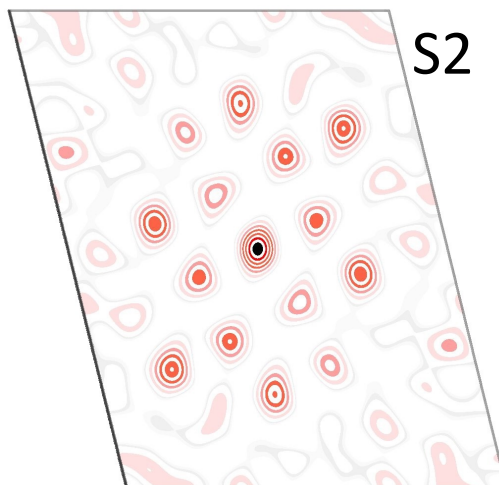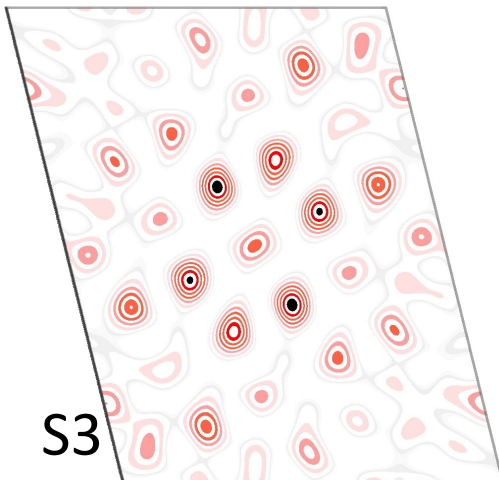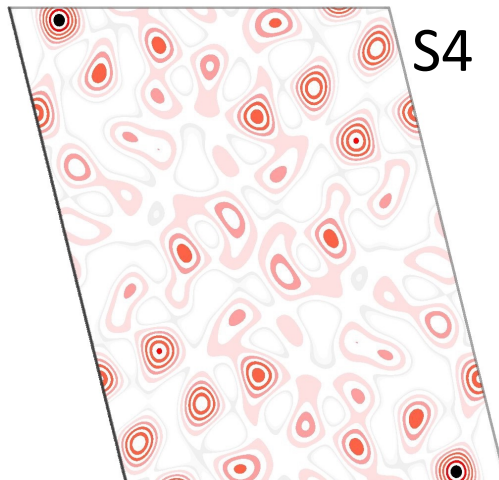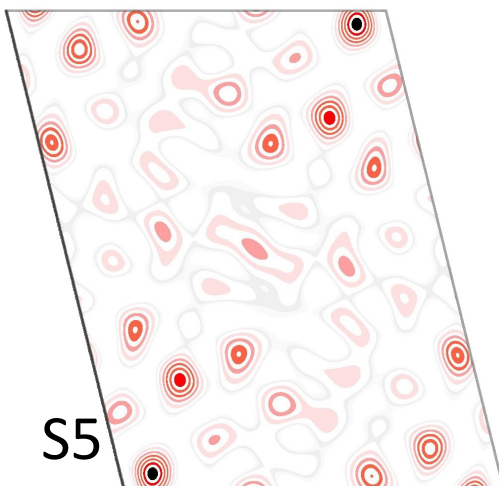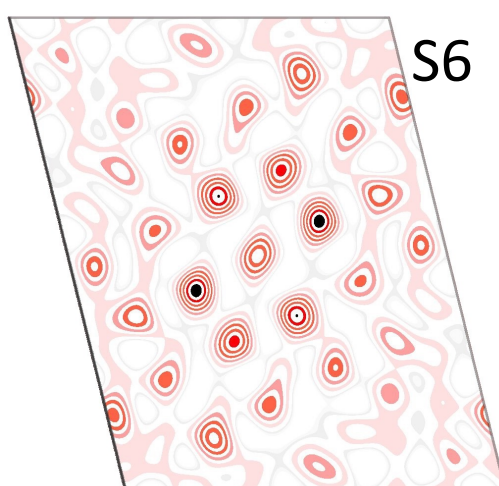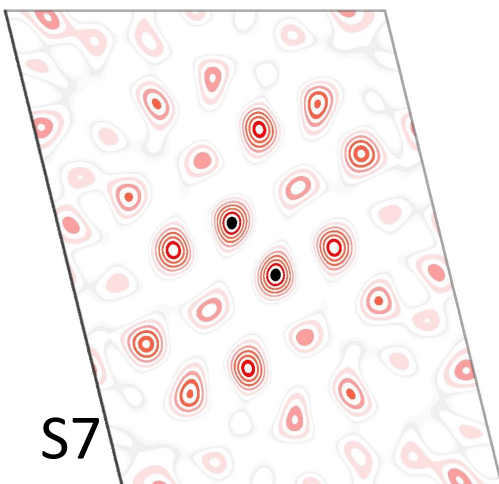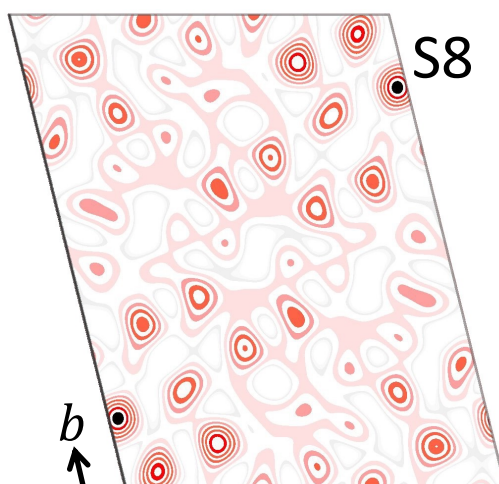

$b$   
 $a$

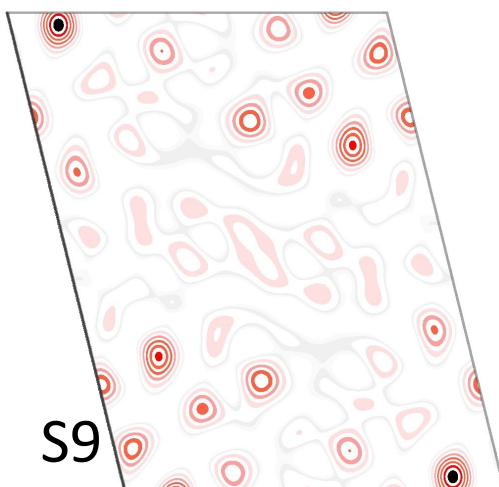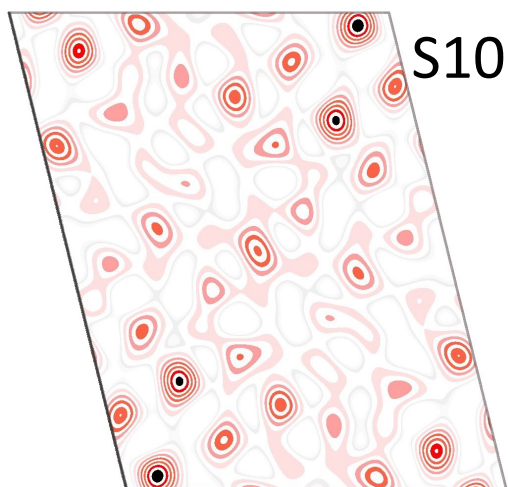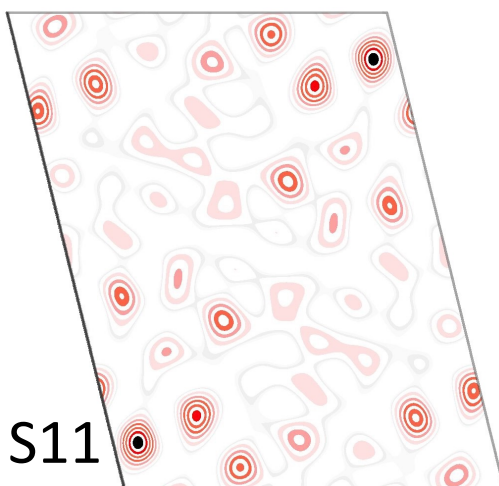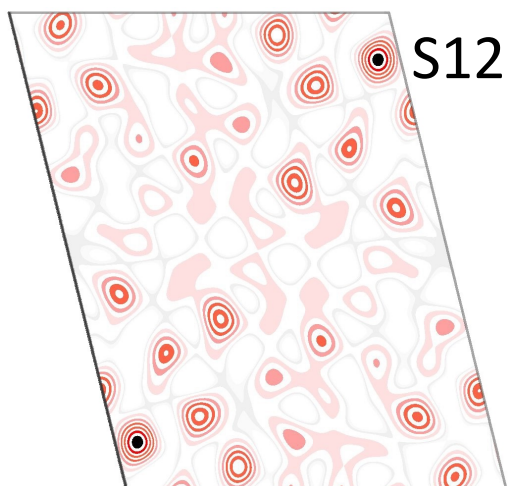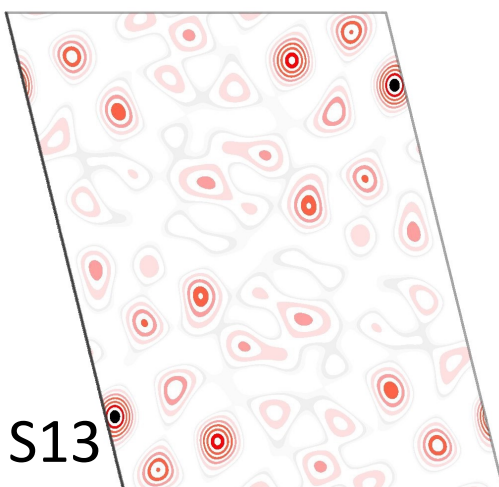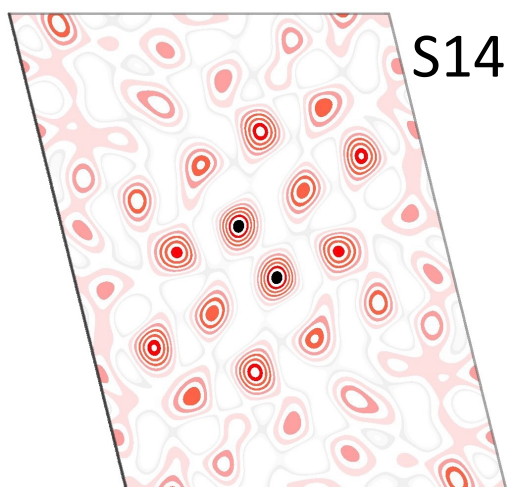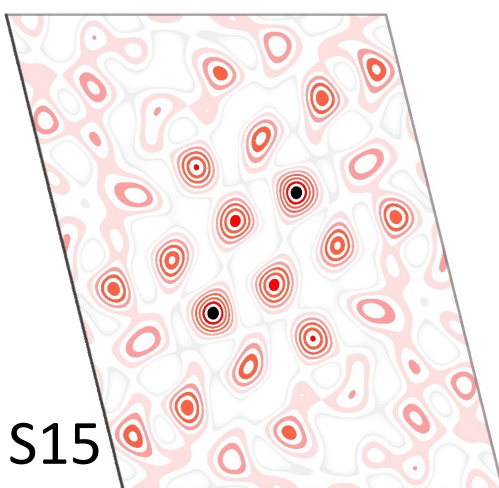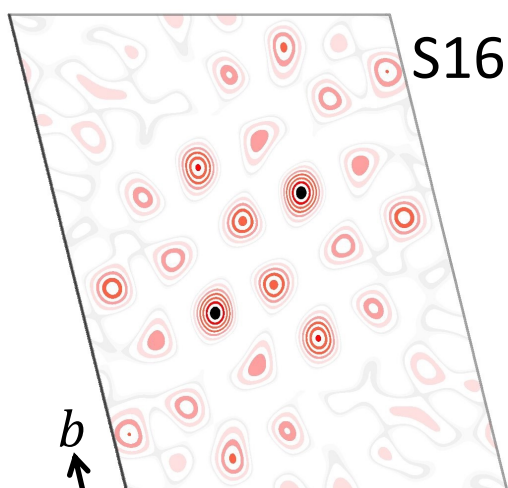

$b$   
 $a$

Supplement: Supplementary file 7 [file e-82-00534-sup8.zip › oi2035_SupportingMaterial/Example3/Example3_Fou_maps.pdf]

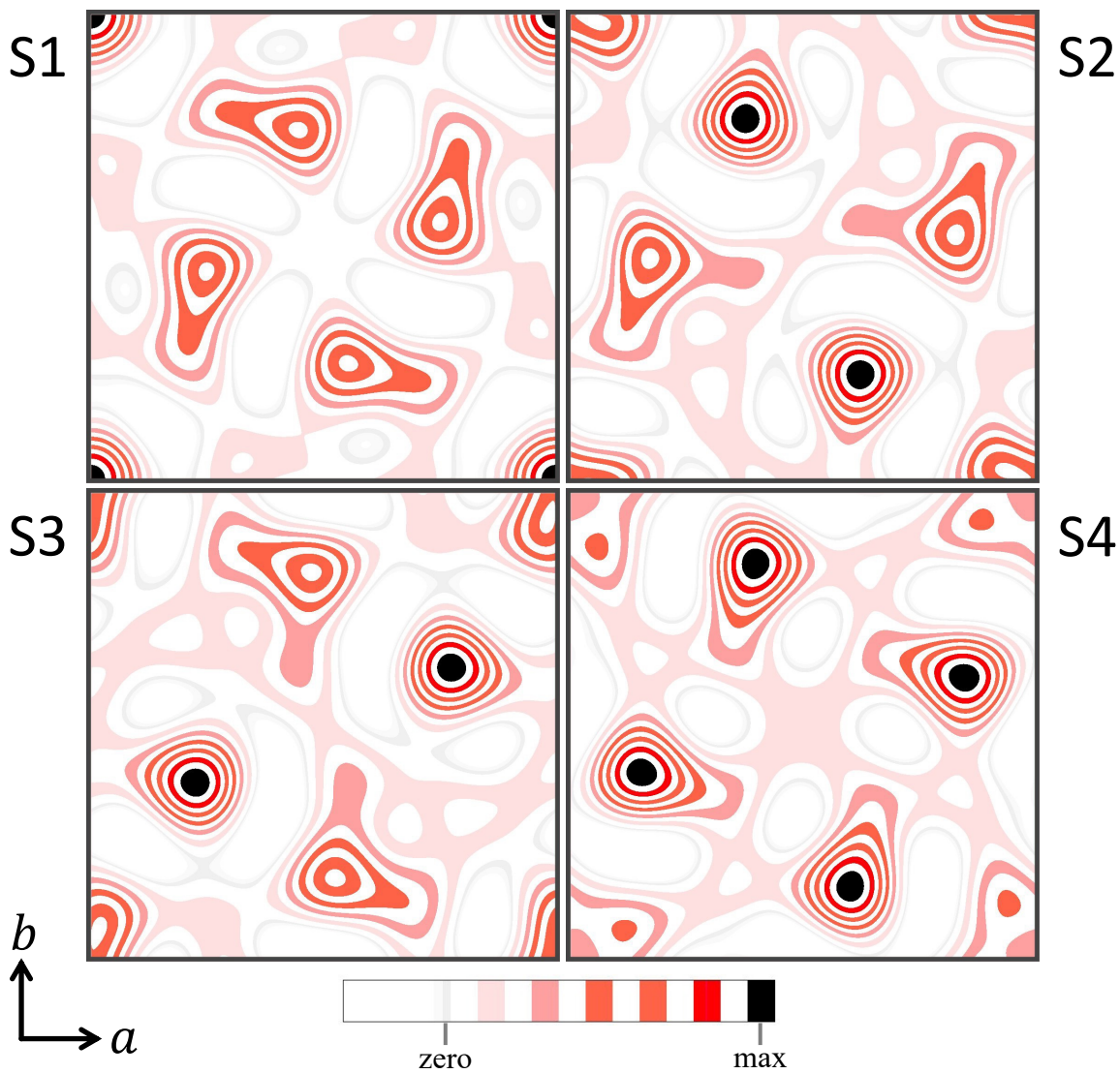

Supplement: Supplementary file 7 [file e-82-00534-sup8.zip › oi2035_SupportingMaterial/Example1/Example1_Fou_maps.pdf]

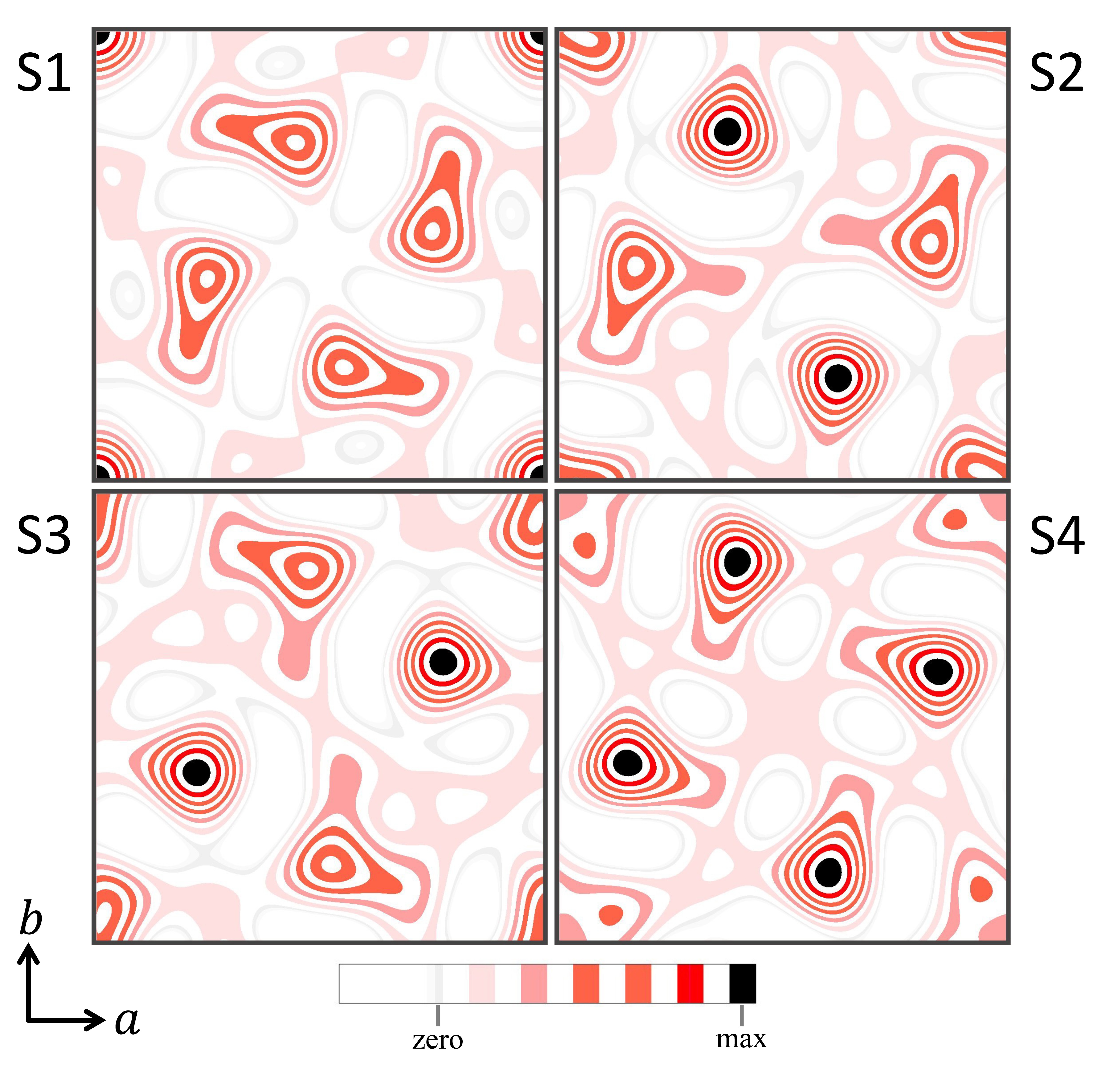

Supplement: Supplementary file 7 [file e-82-00534-sup8.zip › oi2035_SupportingMaterial/Example1/Example1_Fou_maps.jpg]

## Slide 1
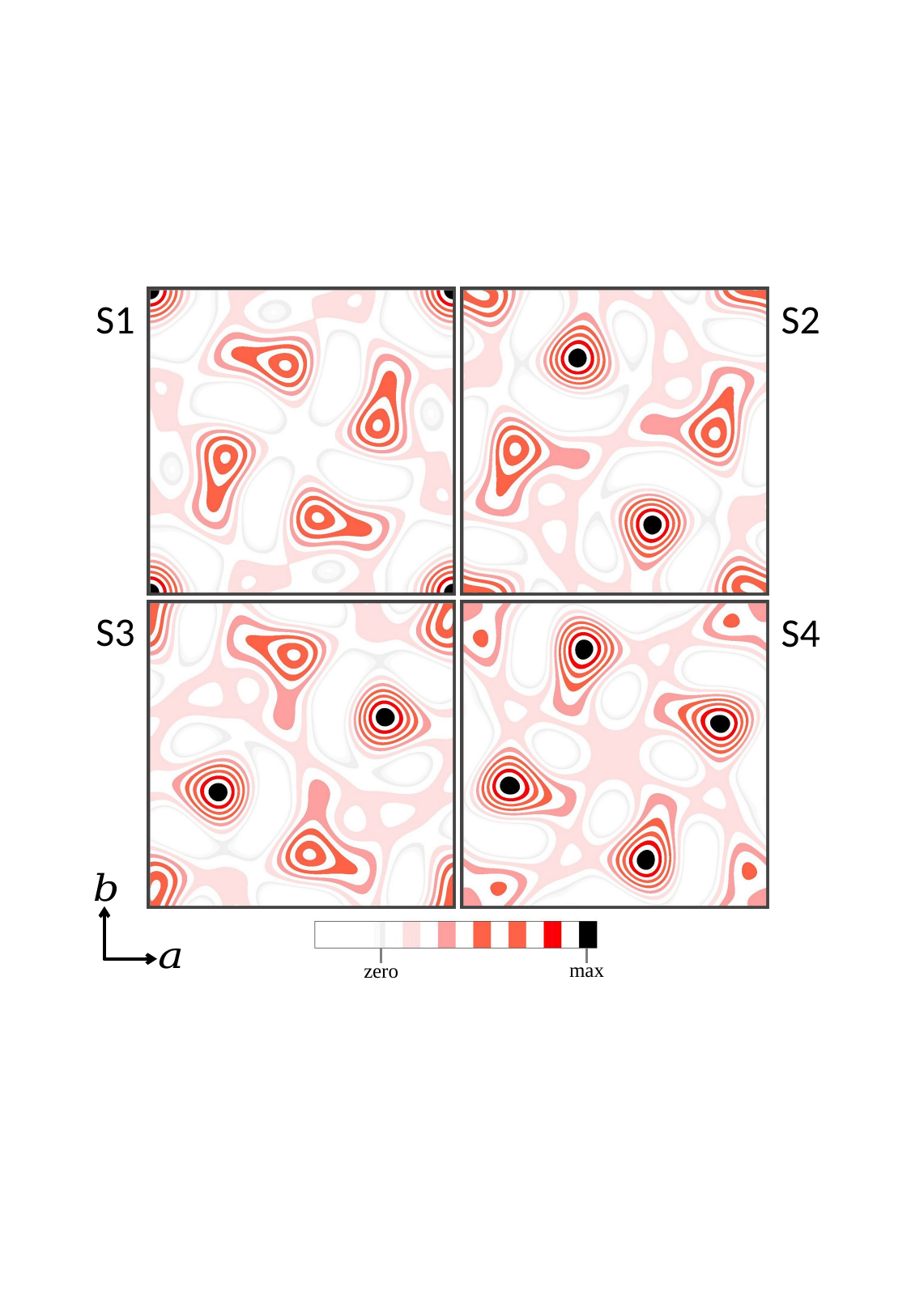

S2
S1
S3
S4
max
zero

Supplement: Supplementary file 7 [file e-82-00534-sup8.zip › oi2035_SupportingMaterial/Example1/Example1_Fou_maps.pptx]

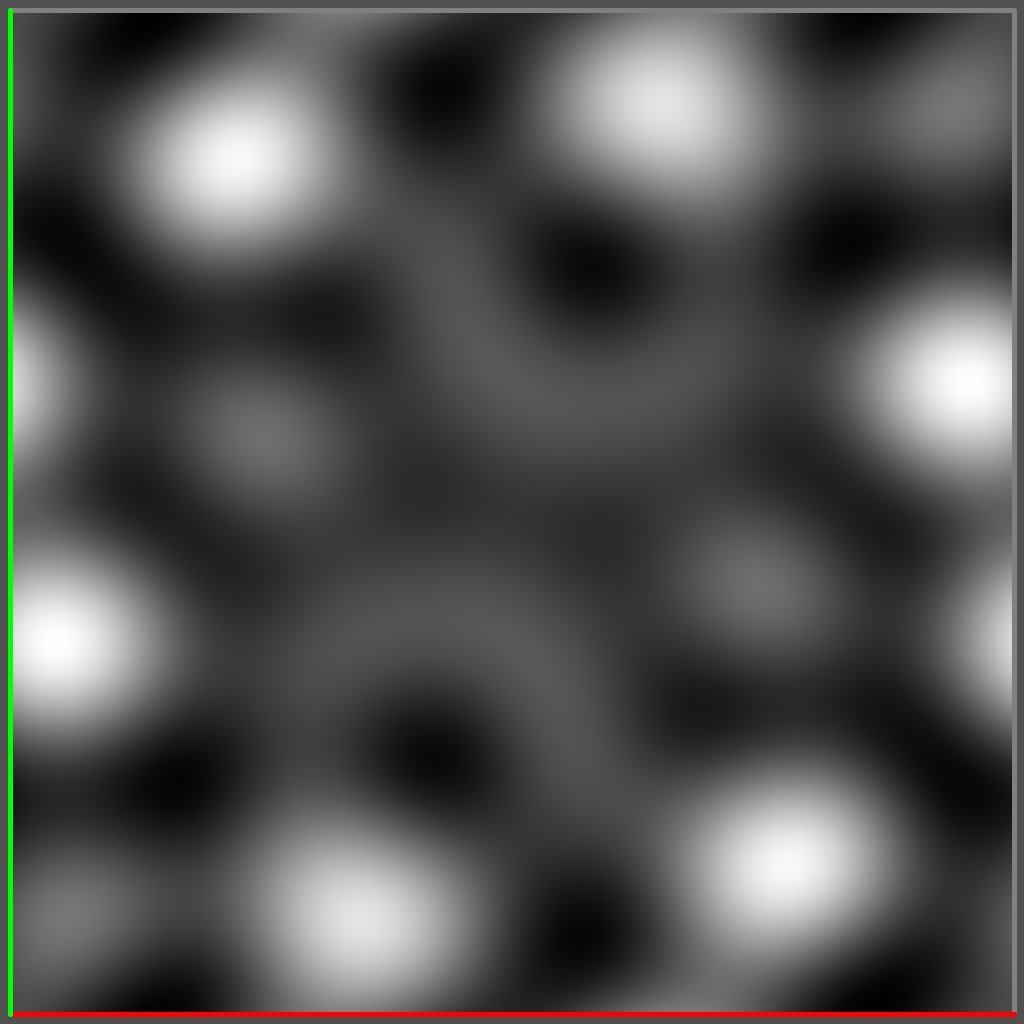

Supplement: Supplementary file 7 [file e-82-00534-sup8.zip › oi2035_SupportingMaterial/Example2/Example2 DISI Kernel calc/Model-2_FOU Fourier-Map_N60_RGB_1024pix.jpg]

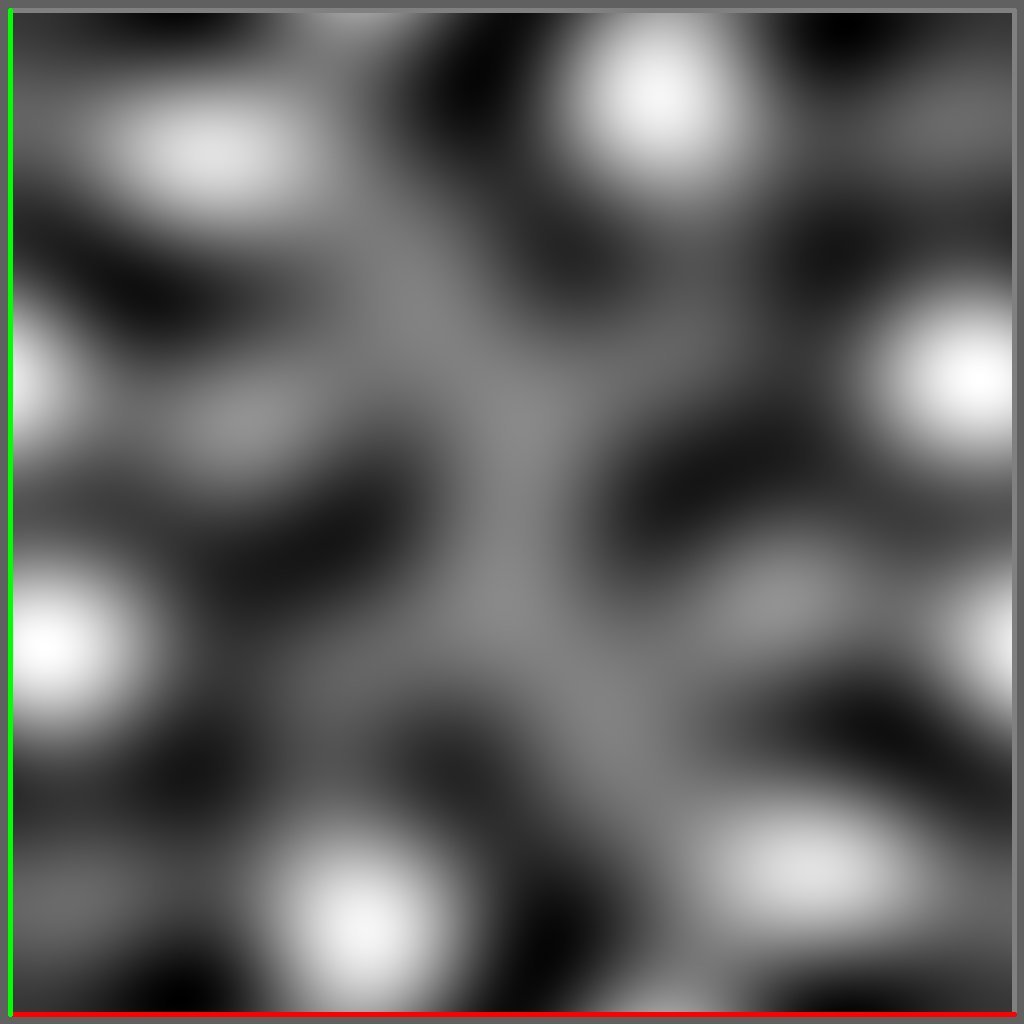

Supplement: Supplementary file 7 [file e-82-00534-sup8.zip › oi2035_SupportingMaterial/Example2/Example2 DISI Kernel calc/Model-2_EFOU Fourier-Map_N24_RGB_1024pix.jpg]

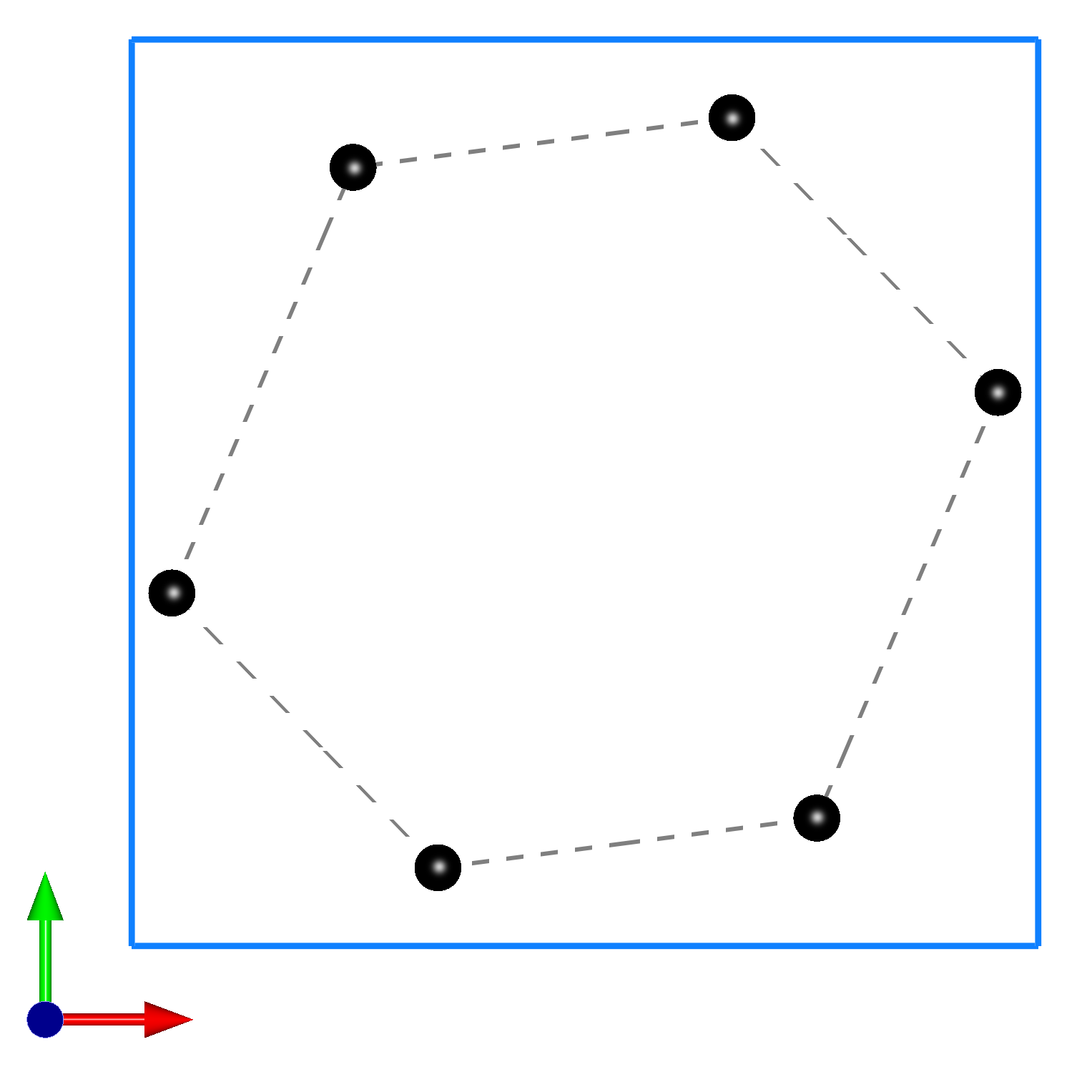

Supplement: Supplementary file 7 [file e-82-00534-sup8.zip › oi2035_SupportingMaterial/Example2/Example2 DISI Kernel calc/Model-2_CIF.png]

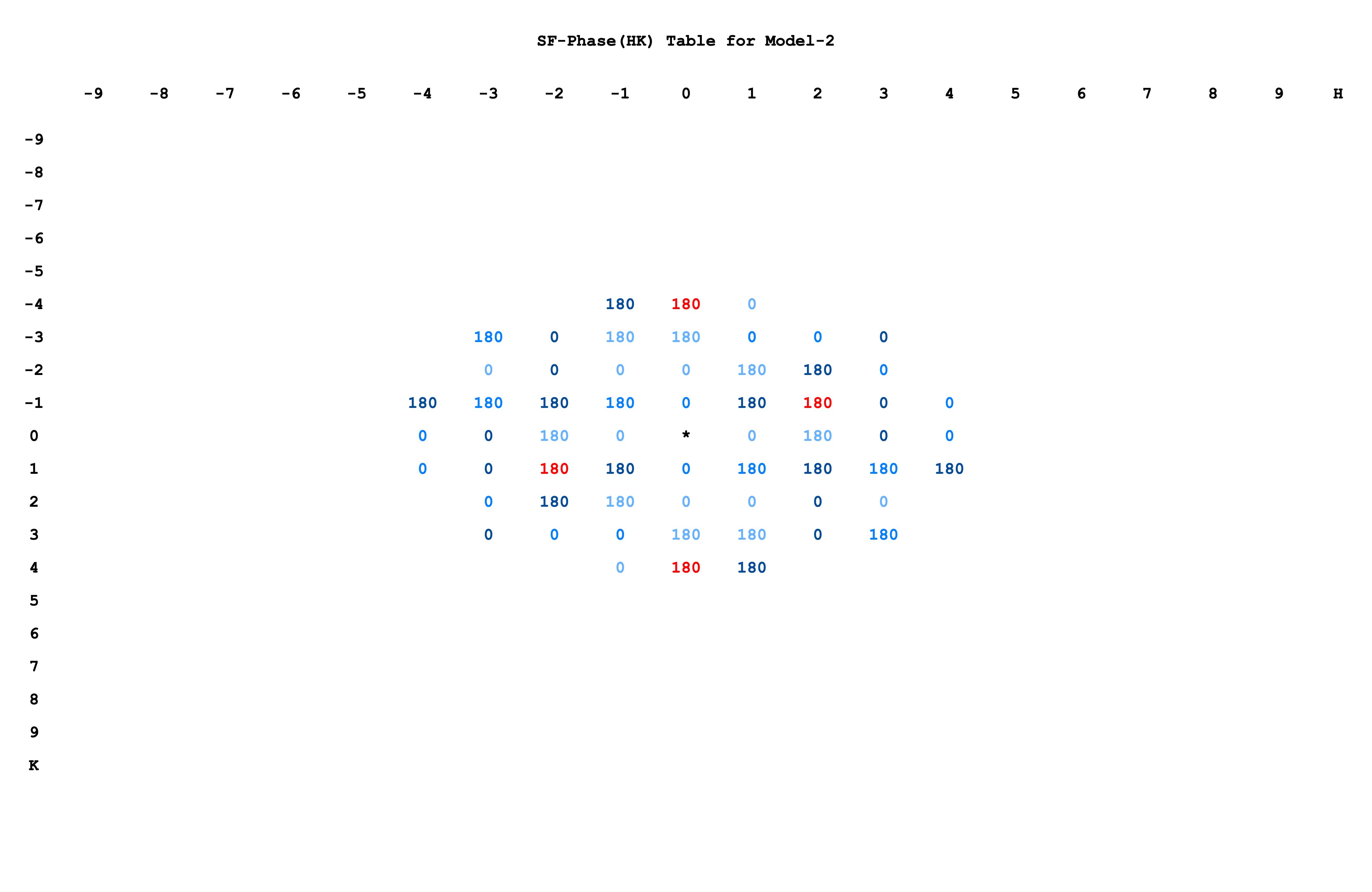

Supplement: Supplementary file 7 [file e-82-00534-sup8.zip › oi2035_SupportingMaterial/Example2/Example2 DISI Kernel calc/Model-2_SF-Phase(HK)-Table.jpg]

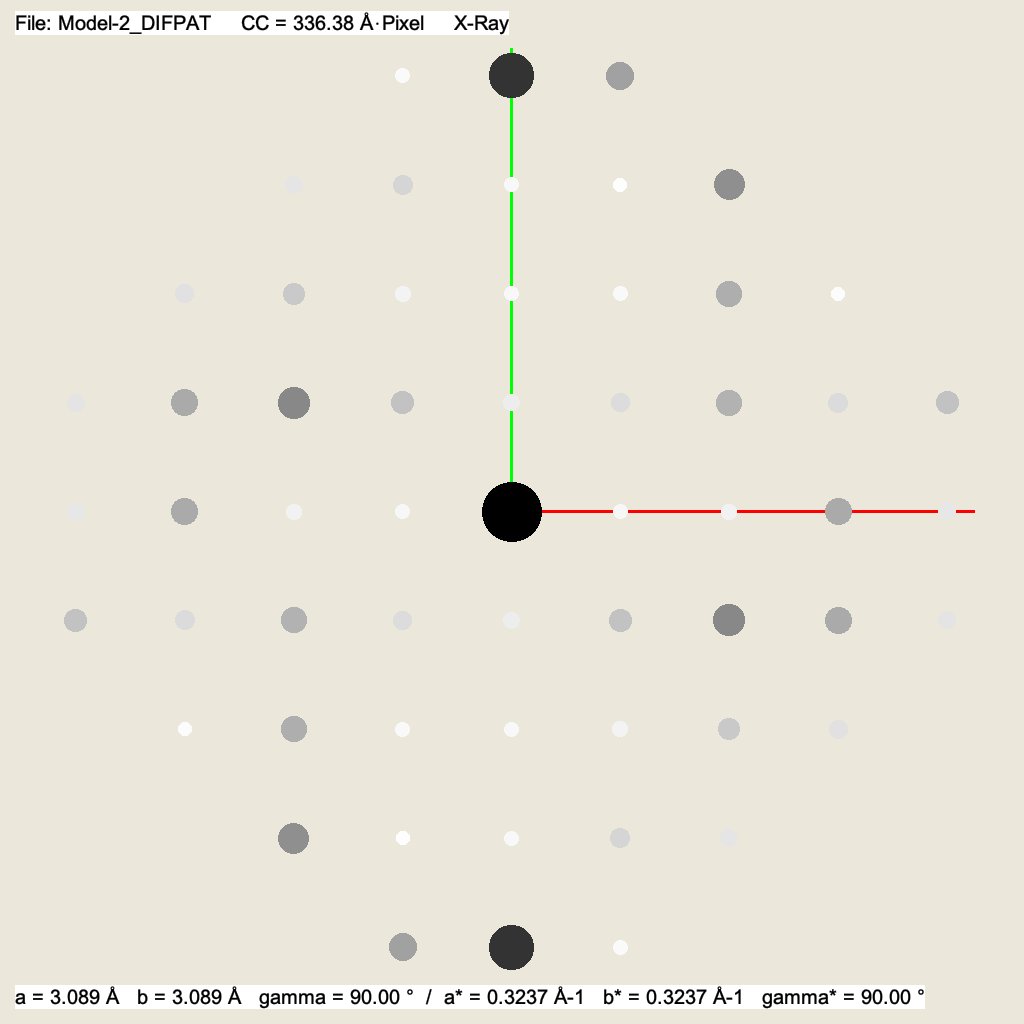

Supplement: Supplementary file 7 [file e-82-00534-sup8.zip › oi2035_SupportingMaterial/Example2/Example2 DISI Kernel calc/Model-2_DIFPAT_CC=336A╠èΓïàPix_Plotsize1024Pix.jpg]

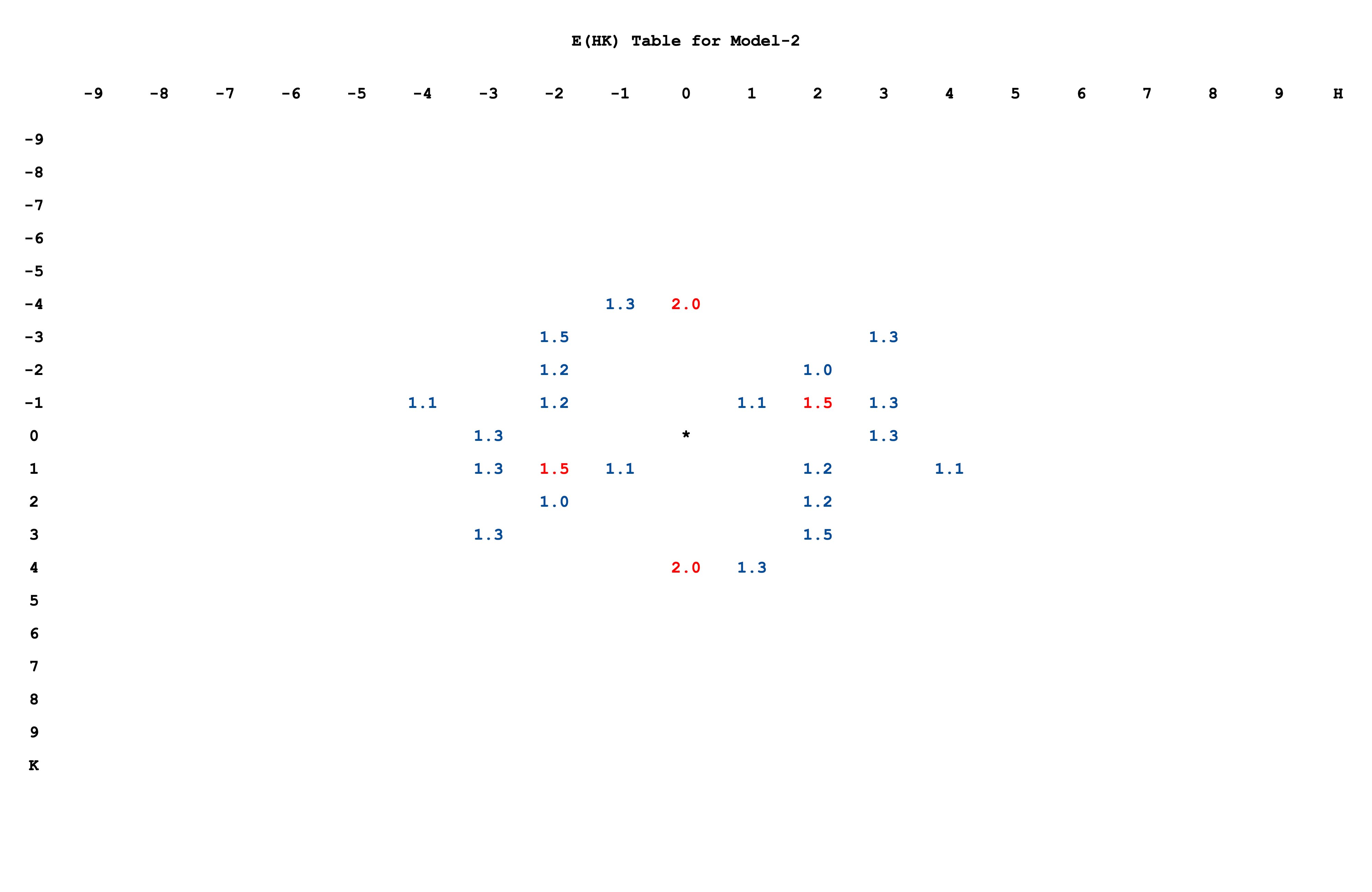

Supplement: Supplementary file 7 [file e-82-00534-sup8.zip › oi2035_SupportingMaterial/Example2/Example2 DISI Kernel calc/Model-2_E(HK)-Table.jpg]

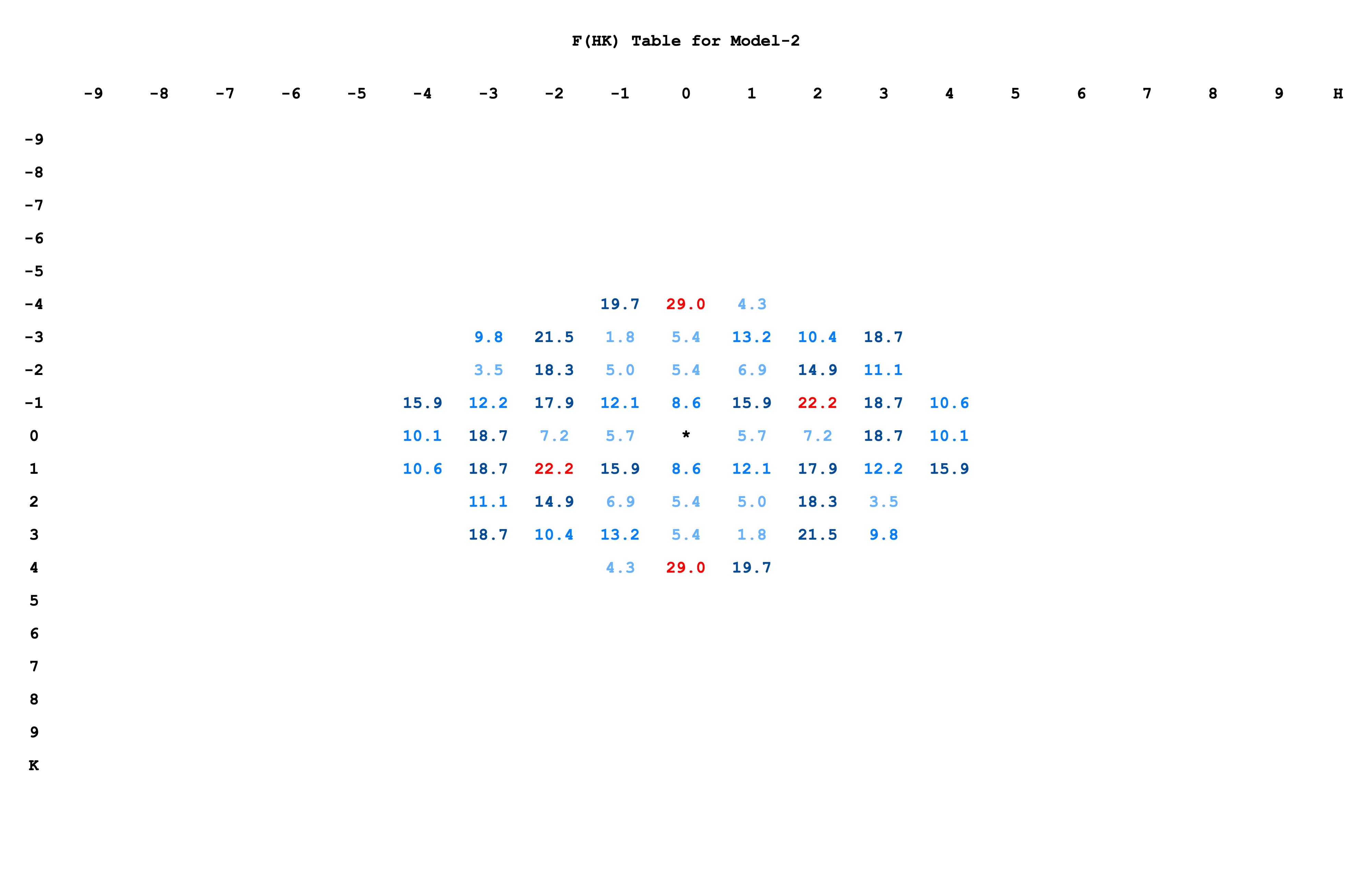

Supplement: Supplementary file 7 [file e-82-00534-sup8.zip › oi2035_SupportingMaterial/Example2/Example2 DISI Kernel calc/Model-2_F(HK)-Table.jpg]

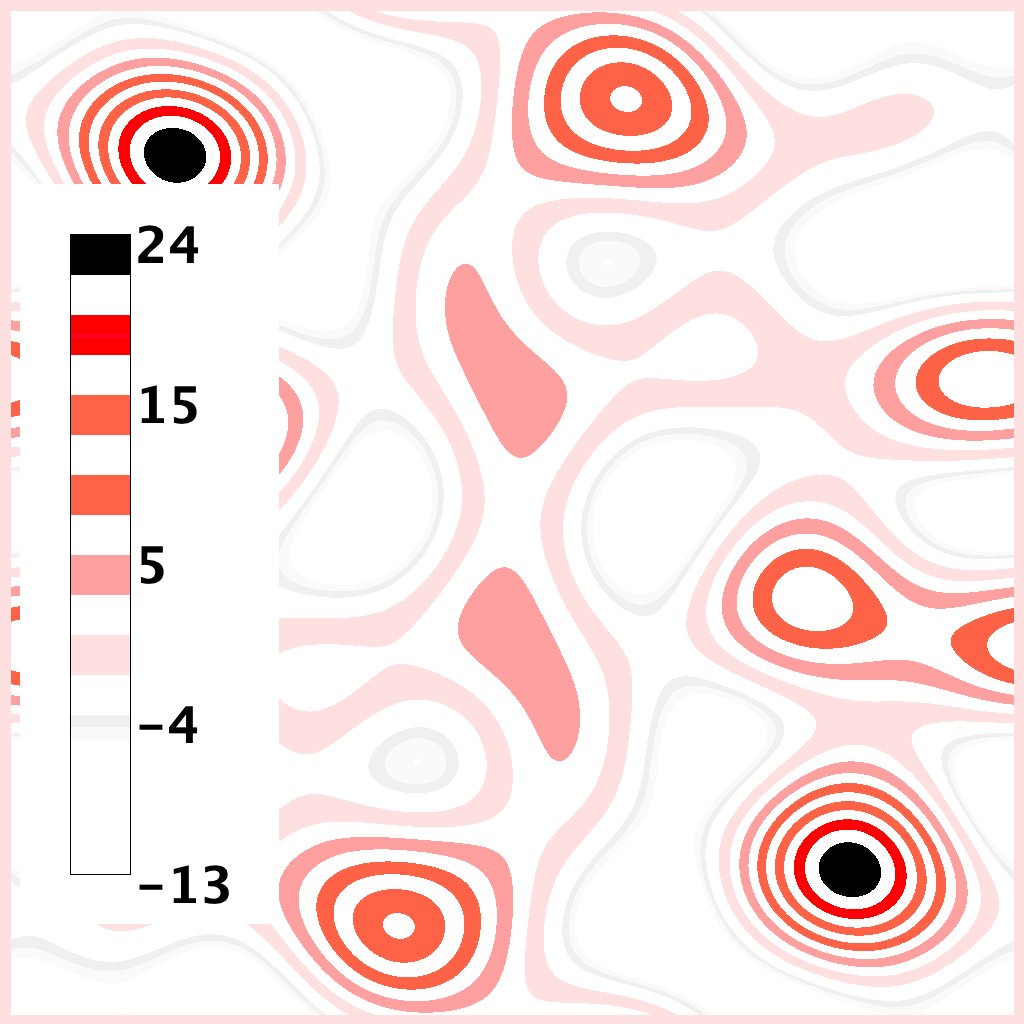

Supplement: Supplementary file 7 [file e-82-00534-sup8.zip › oi2035_SupportingMaterial/Example2/Example2 FOU Maps/Solution2 A=180 B=0 F=0 Fourier-Map_N22_32bit_gray_1024pix_ramp.jpg]

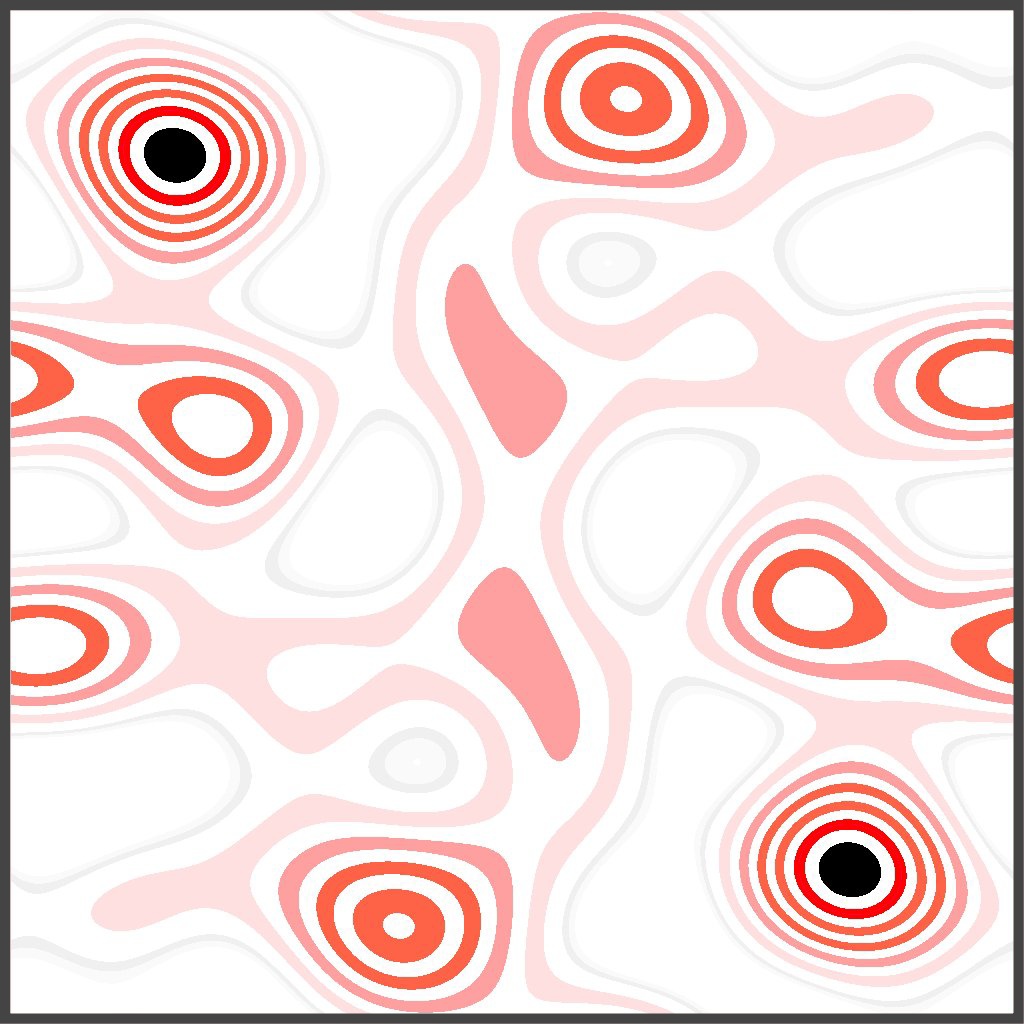

Supplement: Supplementary file 7 [file e-82-00534-sup8.zip › oi2035_SupportingMaterial/Example2/Example2 FOU Maps/Frame_Solution2 A=180 B=0 F=0 Fourier-Map_N22_32bit_gray_1024pix_LUT3.jpg]

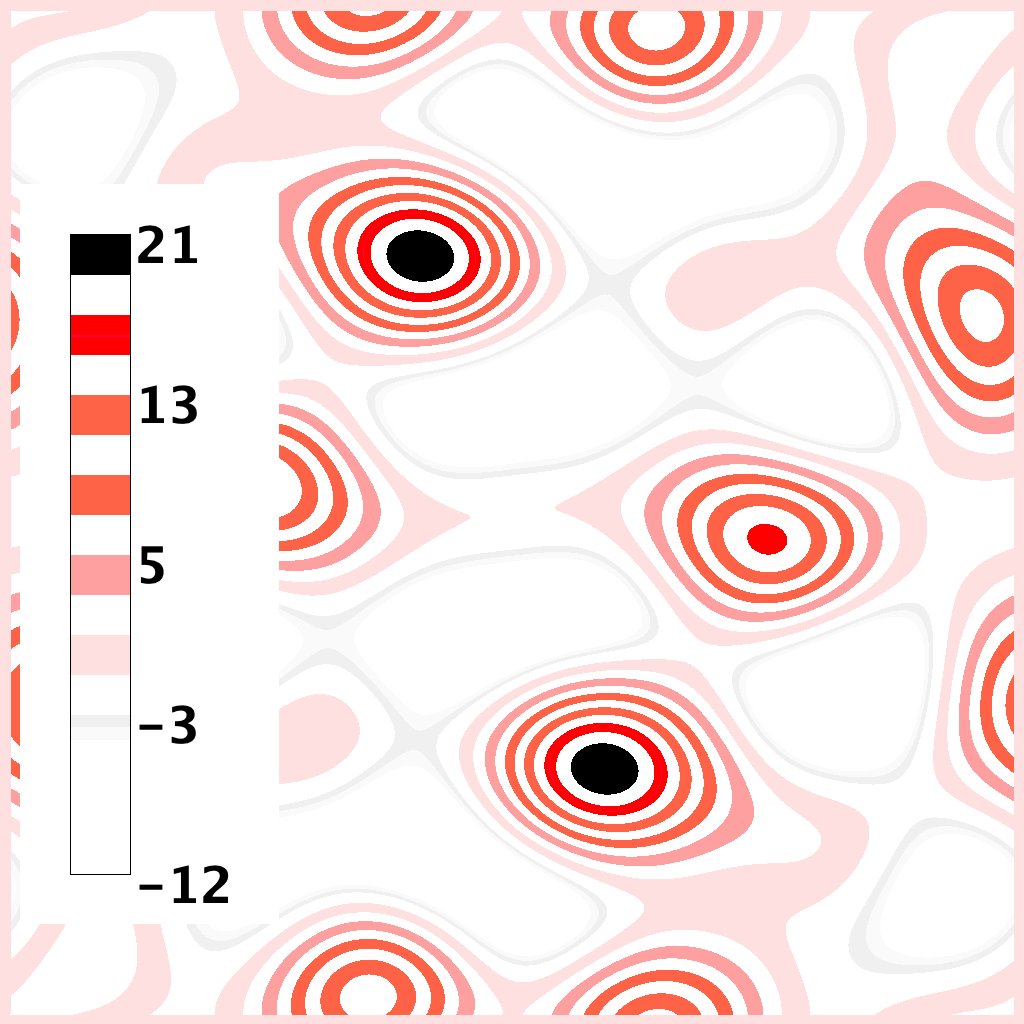

Supplement: Supplementary file 7 [file e-82-00534-sup8.zip › oi2035_SupportingMaterial/Example2/Example2 FOU Maps/Solution5 A=0 B=180 F=180 Fourier-Map_N22_32bit_gray_1024pix_ramp.jpg]

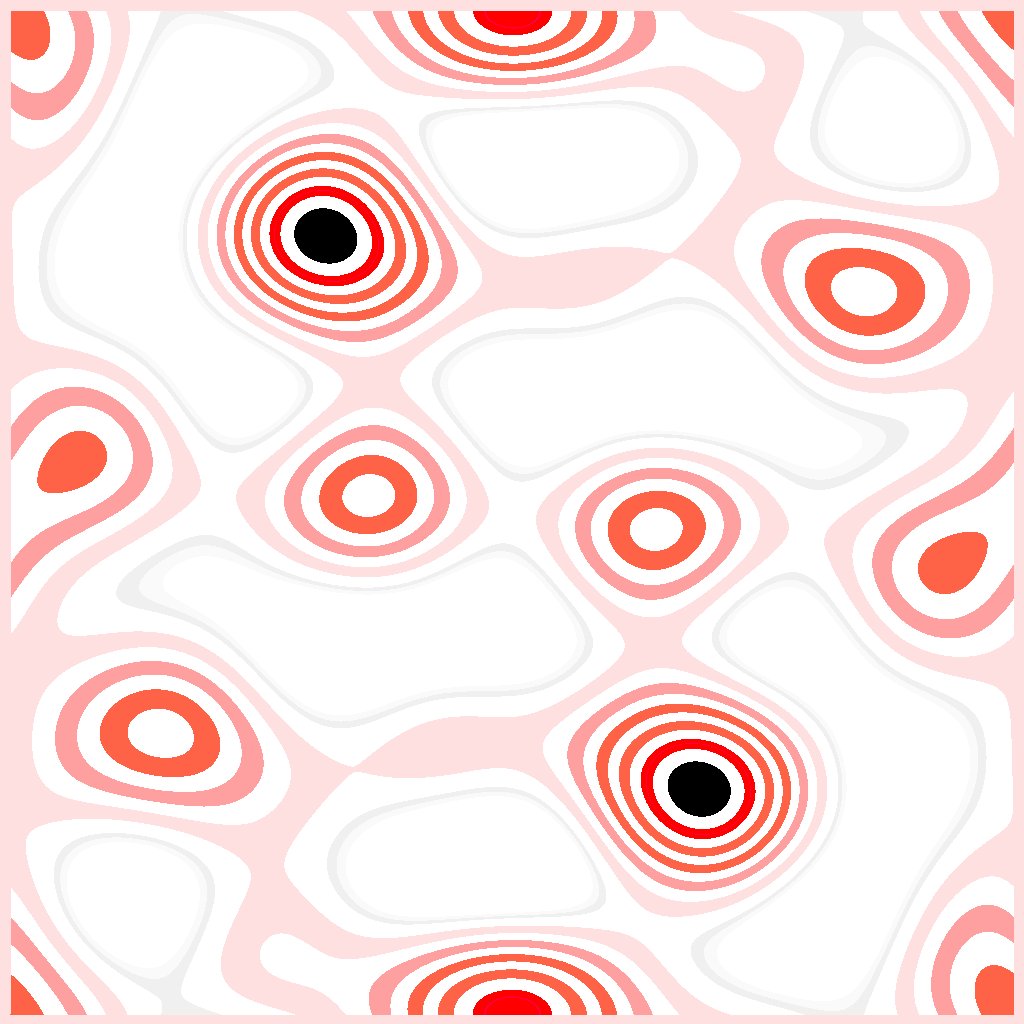

Supplement: Supplementary file 7 [file e-82-00534-sup8.zip › oi2035_SupportingMaterial/Example2/Example2 FOU Maps/Solution4 A=0 B=0 F=180 Fourier-Map_N22_32bit_gray_1024pix_LUT3.jpg]

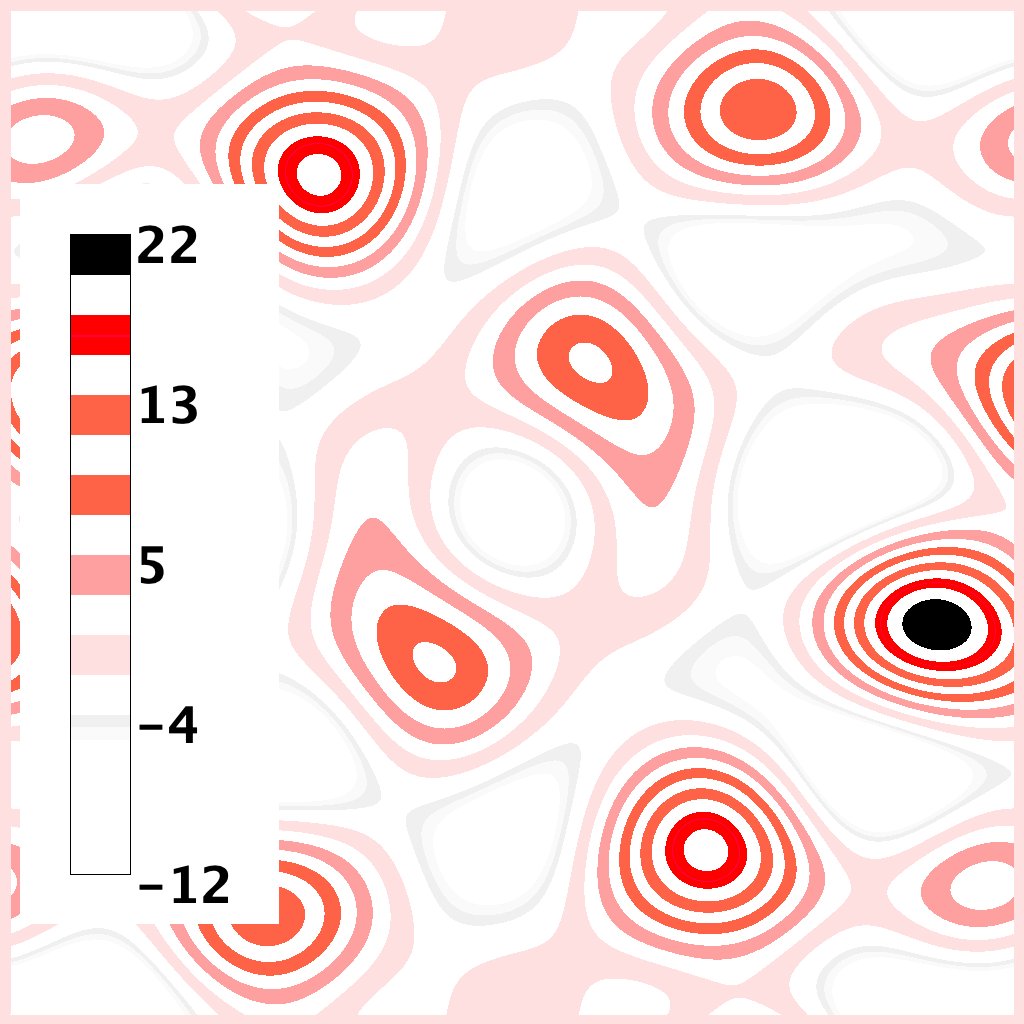

Supplement: Supplementary file 7 [file e-82-00534-sup8.zip › oi2035_SupportingMaterial/Example2/Example2 FOU Maps/Solution8 A=180 B=180 F=180 Fourier-Map_N22_32bit_gray_1024pix_ramp.jpg]

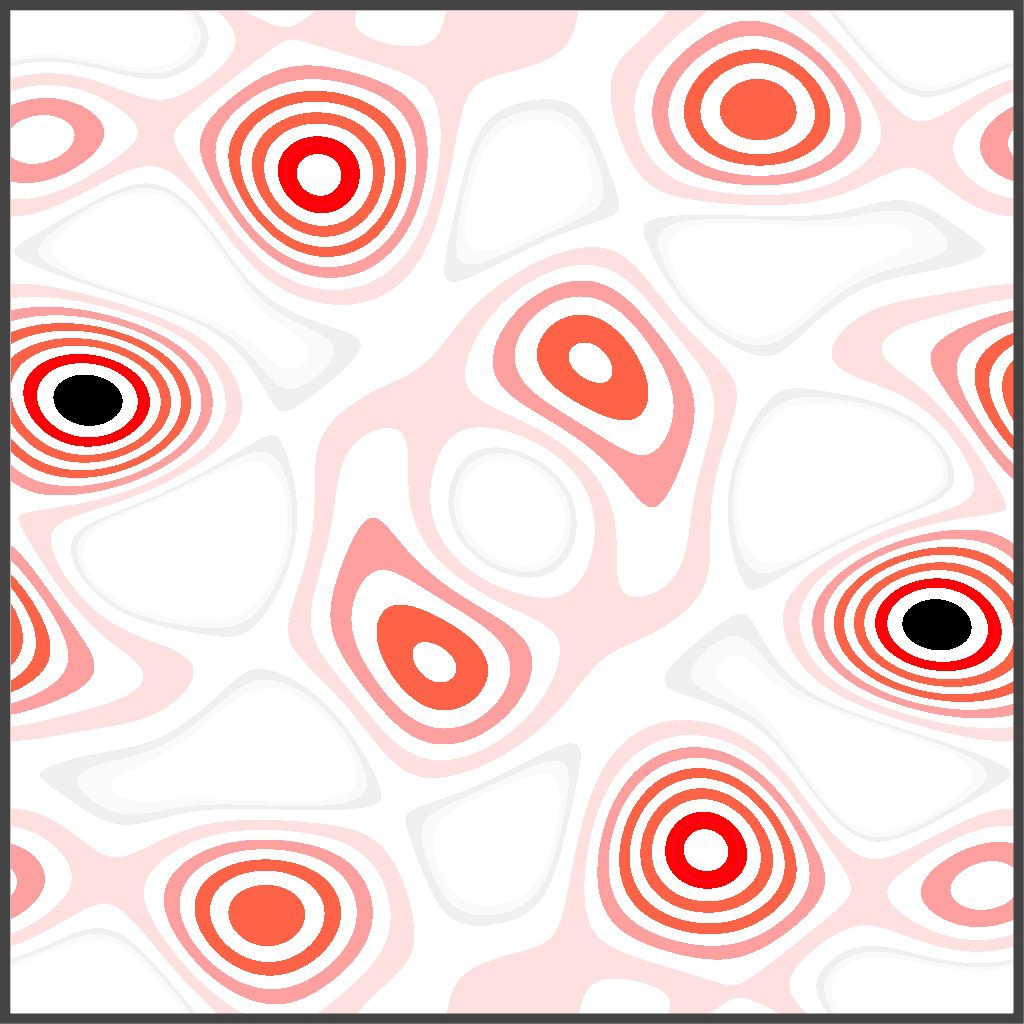

Supplement: Supplementary file 7 [file e-82-00534-sup8.zip › oi2035_SupportingMaterial/Example2/Example2 FOU Maps/Frame_Solution8 A=180 B=180 F=180 Fourier-Map_N22_32bit_gray_1024pix_LUT3.jpg]

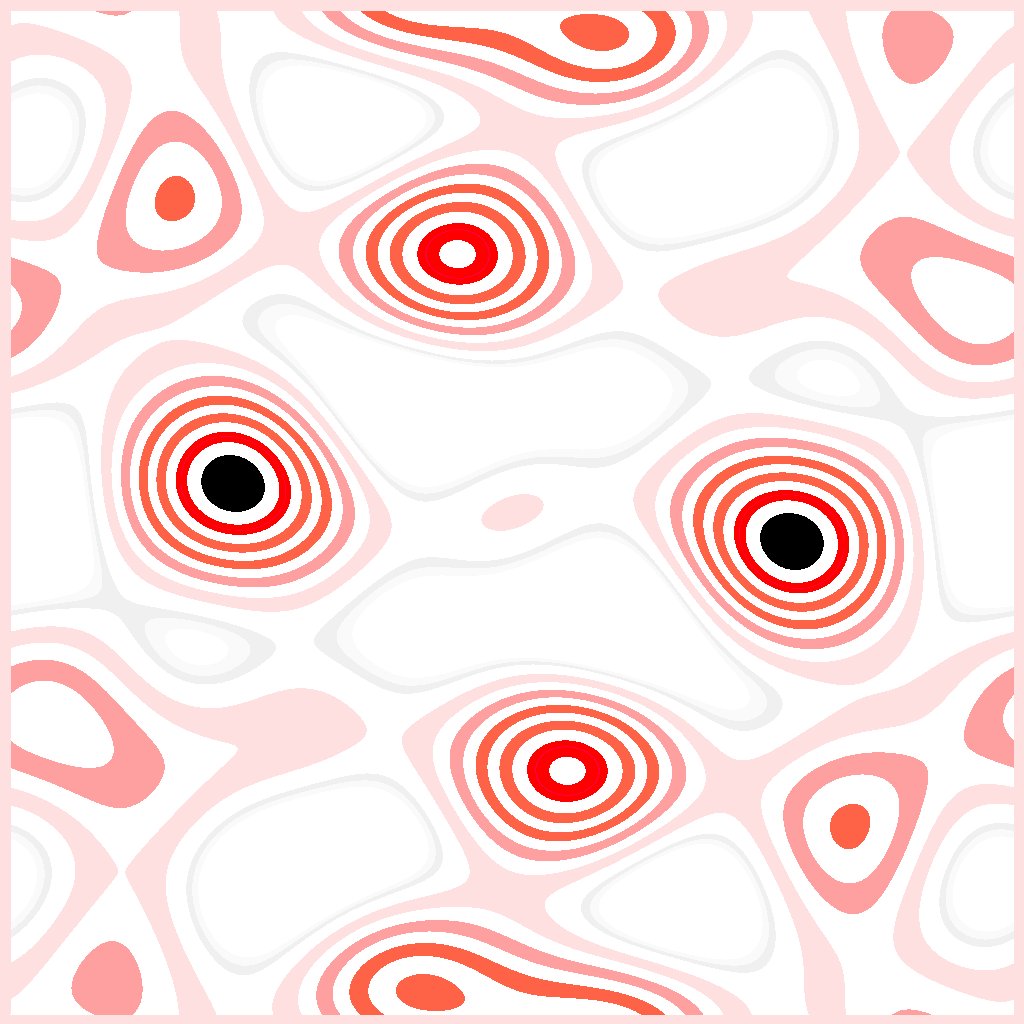

Supplement: Supplementary file 7 [file e-82-00534-sup8.zip › oi2035_SupportingMaterial/Example2/Example2 FOU Maps/Solution3 A=0 B=180 F=0 Fourier-Map_N22_32bit_gray_1024pix_LUT3.jpg]

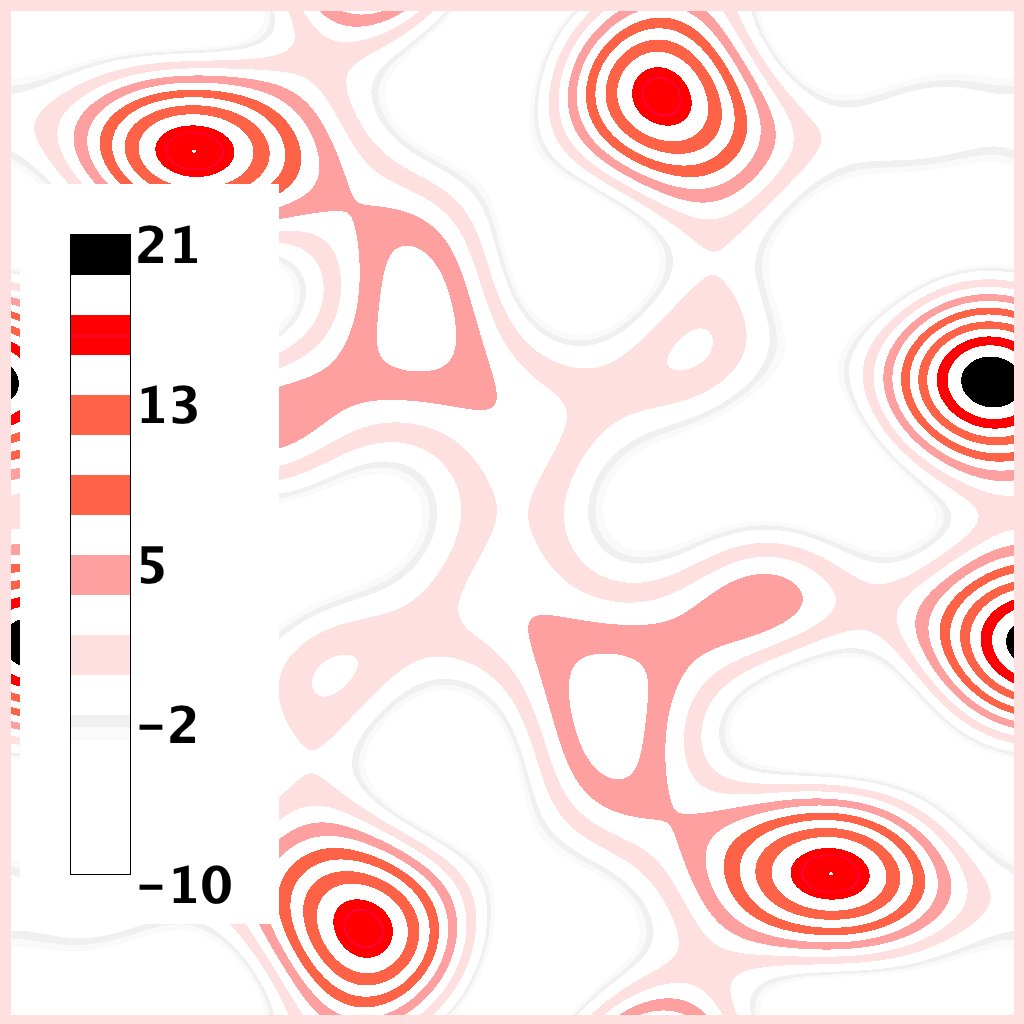

Supplement: Supplementary file 7 [file e-82-00534-sup8.zip › oi2035_SupportingMaterial/Example2/Example2 FOU Maps/Solution6 A=180 B=0 F=180 Fourier-Map_N22_32bit_gray_1024pix_ramp.jpg]

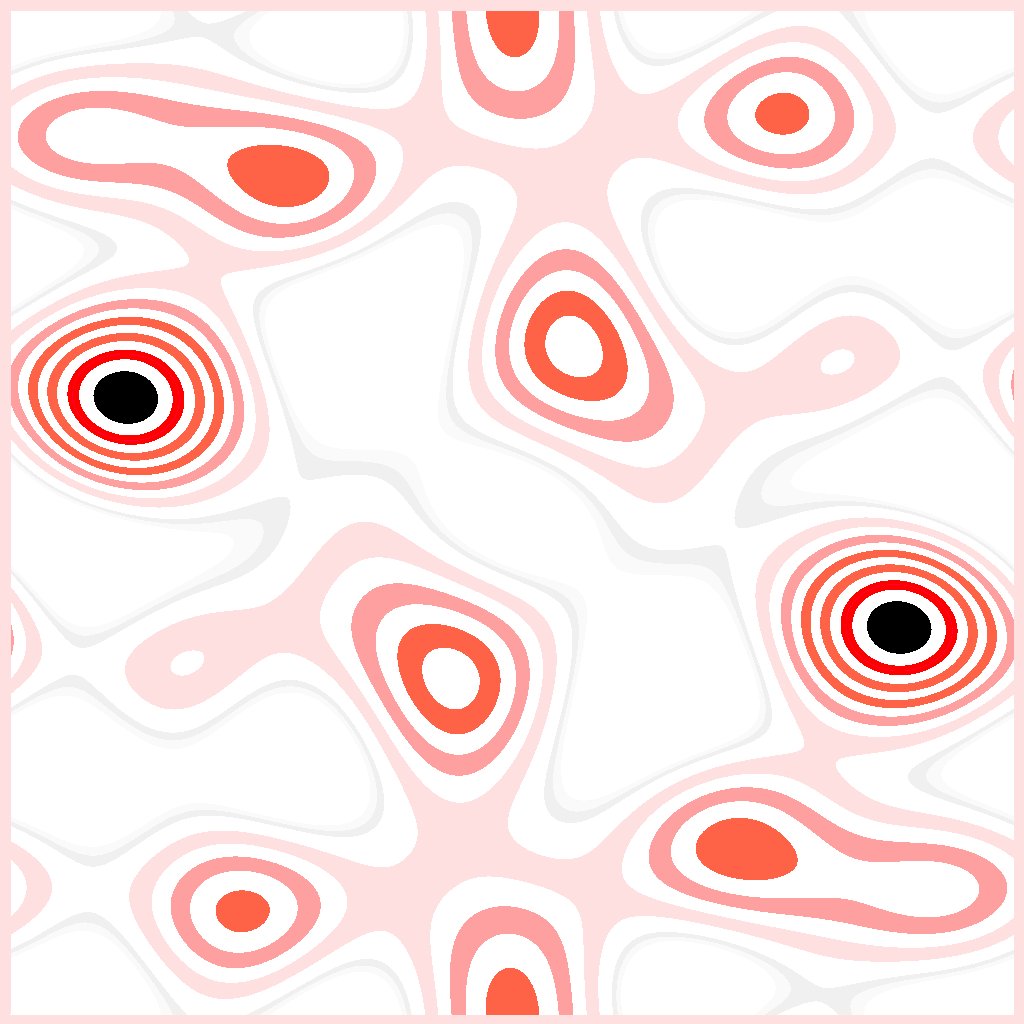

Supplement: Supplementary file 7 [file e-82-00534-sup8.zip › oi2035_SupportingMaterial/Example2/Example2 FOU Maps/Solution7 A=180 B=180 F=0 Fourier-Map_N22_32bit_gray_1024pix_LUT3.jpg]

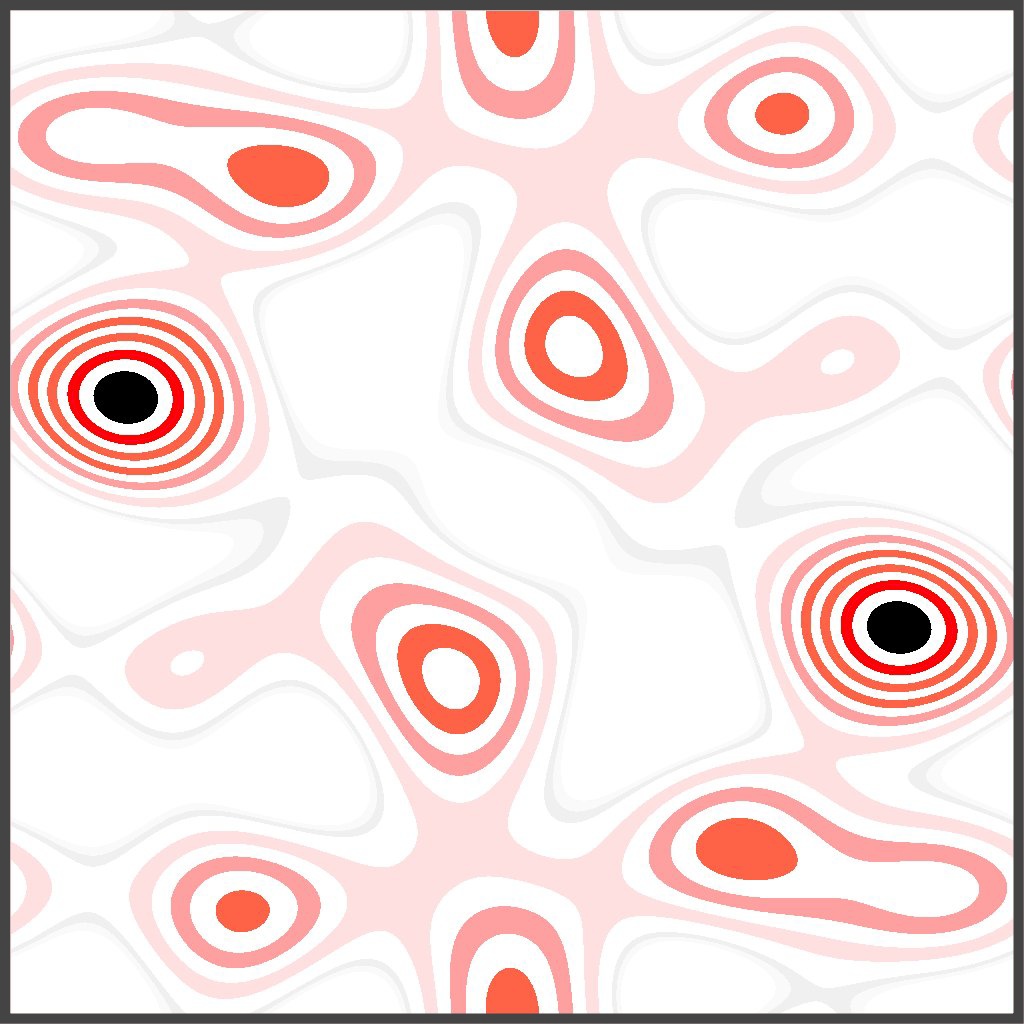

Supplement: Supplementary file 7 [file e-82-00534-sup8.zip › oi2035_SupportingMaterial/Example2/Example2 FOU Maps/Frame_Solution7 A=180 B=180 F=0 Fourier-Map_N22_32bit_gray_1024pix_LUT3.jpg]

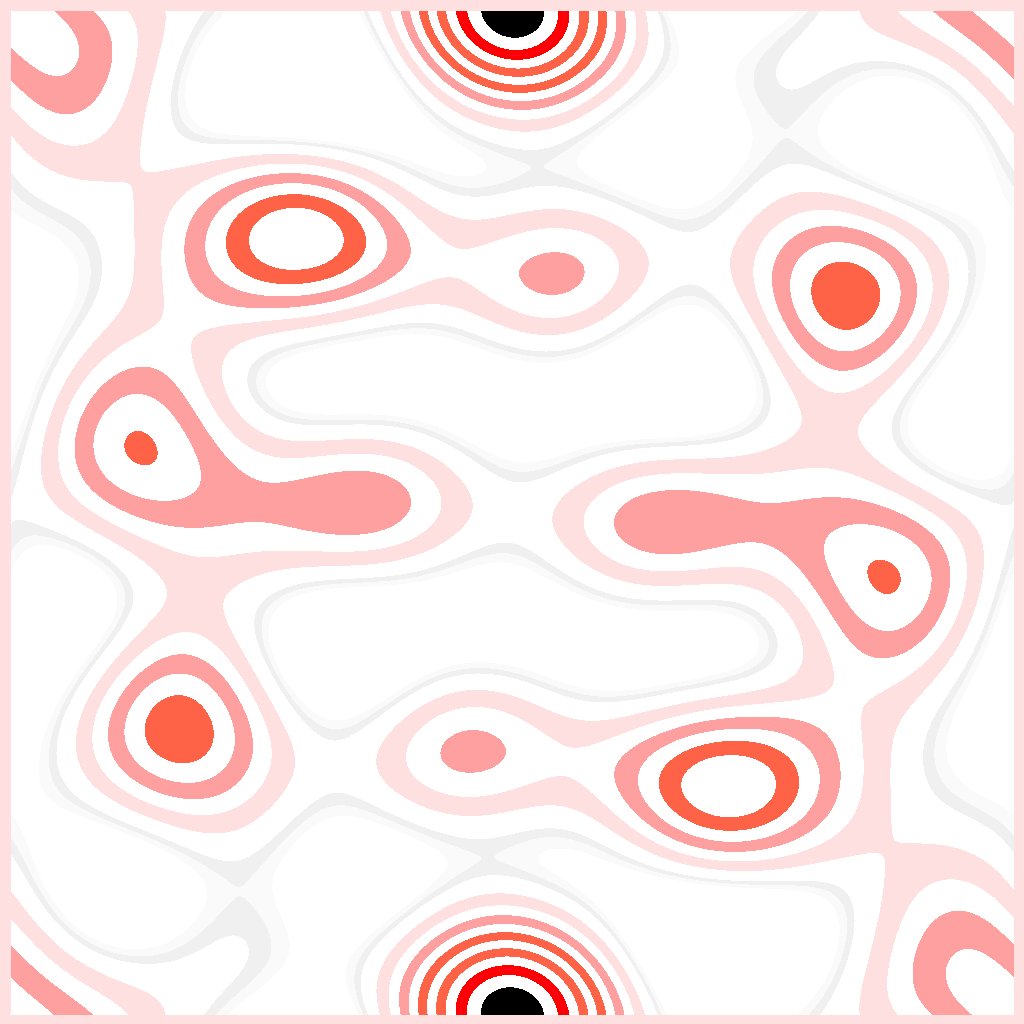

Supplement: Supplementary file 7 [file e-82-00534-sup8.zip › oi2035_SupportingMaterial/Example2/Example2 FOU Maps/Solution1 A=0 B=0 F=0 Fourier-Map_N22_32bit_gray_1024pix_LUT1.jpg]

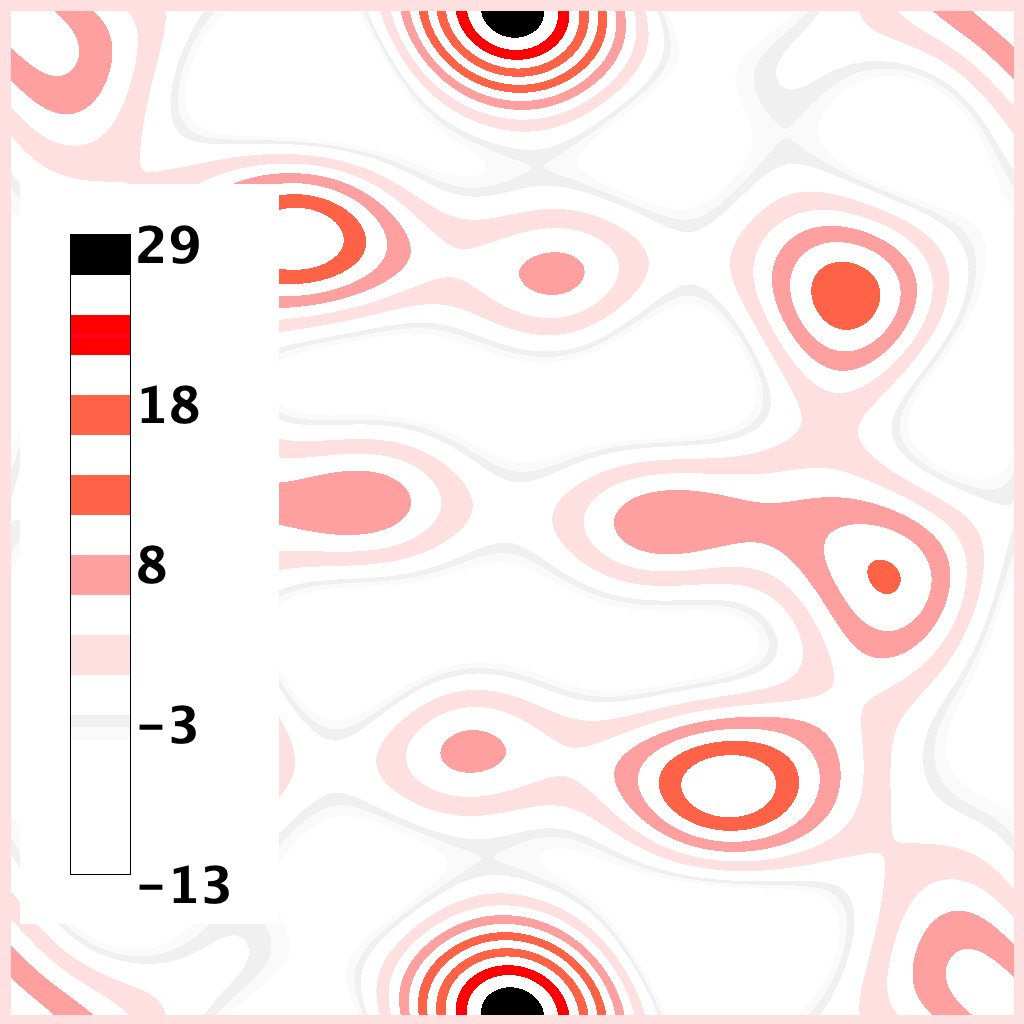

Supplement: Supplementary file 7 [file e-82-00534-sup8.zip › oi2035_SupportingMaterial/Example2/Example2 FOU Maps/Solution1 A=0 B=0 F=0 Fourier-Map_N22_32bit_gray_1024pix_ramp.jpg]

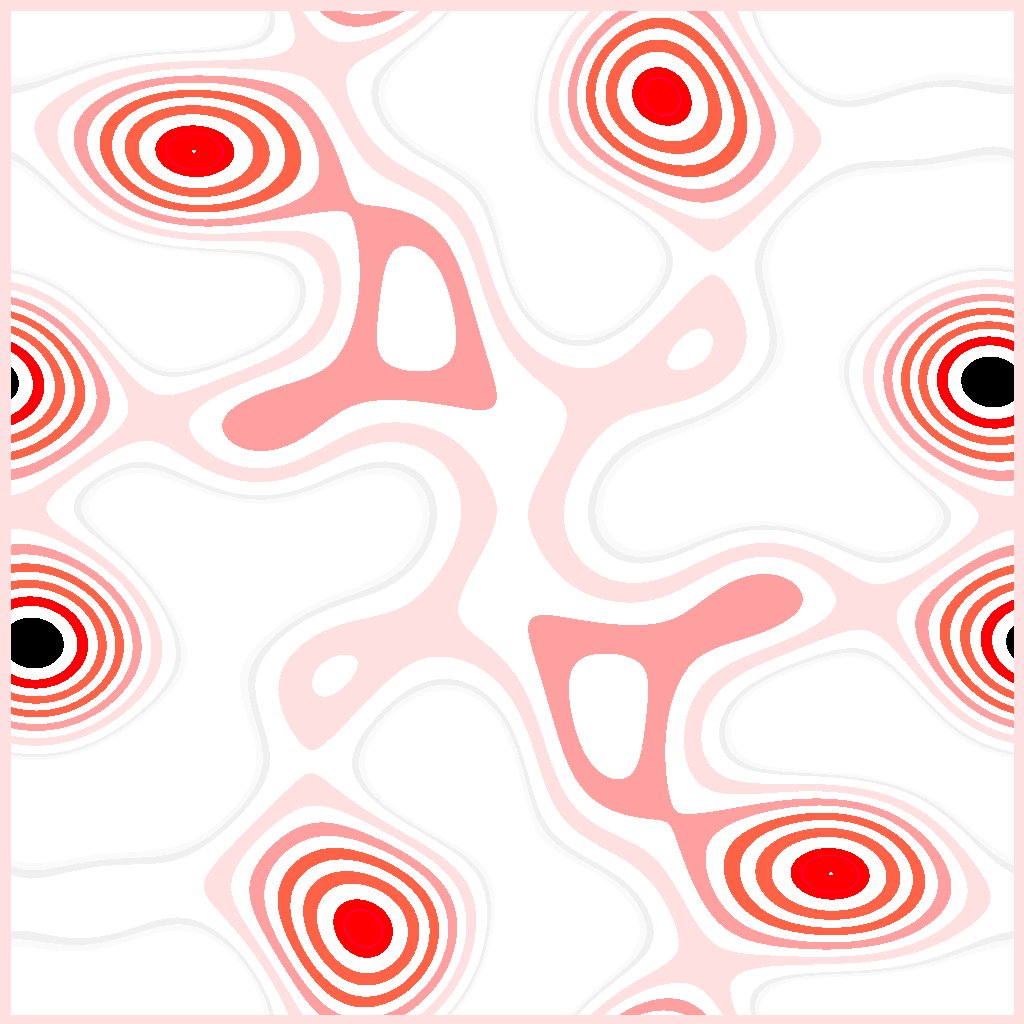

Supplement: Supplementary file 7 [file e-82-00534-sup8.zip › oi2035_SupportingMaterial/Example2/Example2 FOU Maps/Solution6 A=180 B=0 F=180 Fourier-Map_N22_32bit_gray_1024pix_LUT3.jpg]

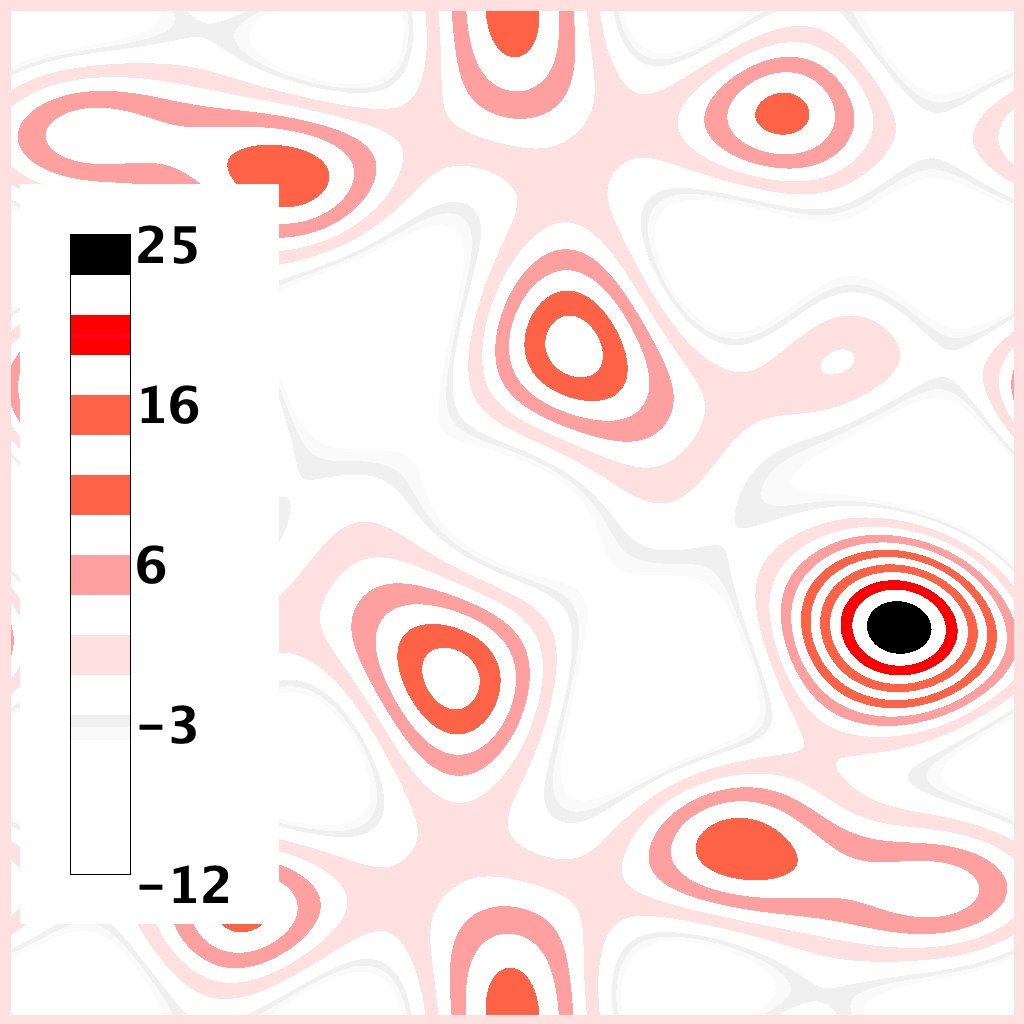

Supplement: Supplementary file 7 [file e-82-00534-sup8.zip › oi2035_SupportingMaterial/Example2/Example2 FOU Maps/Solution7 A=180 B=180 F=0 Fourier-Map_N22_32bit_gray_1024pix_ramp.jpg]

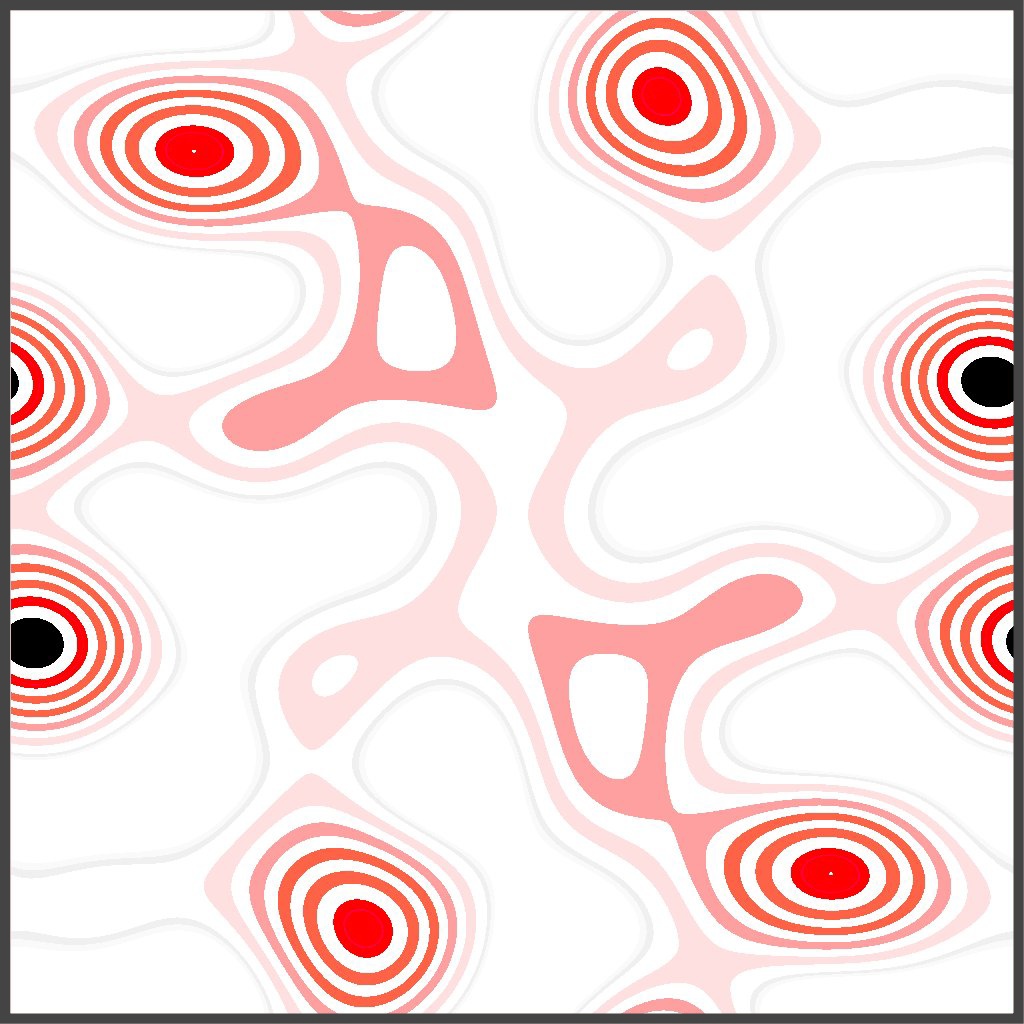

Supplement: Supplementary file 7 [file e-82-00534-sup8.zip › oi2035_SupportingMaterial/Example2/Example2 FOU Maps/Frame_Solution6 A=180 B=0 F=180 Fourier-Map_N22_32bit_gray_1024pix_LUT3.jpg]

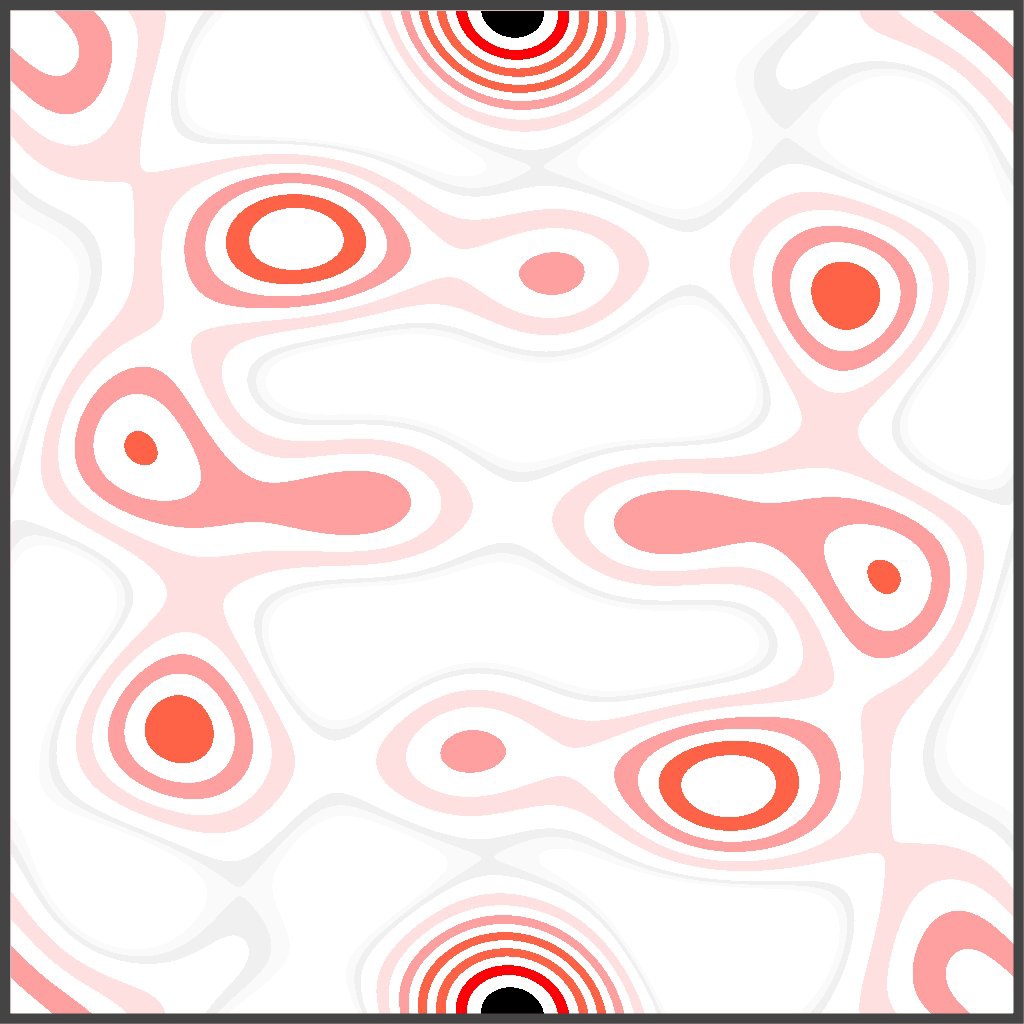

Supplement: Supplementary file 7 [file e-82-00534-sup8.zip › oi2035_SupportingMaterial/Example2/Example2 FOU Maps/Frame_Solution1 A=0 B=0 F=0 Fourier-Map_N22_32bit_gray_1024pix_LUT1.jpg]

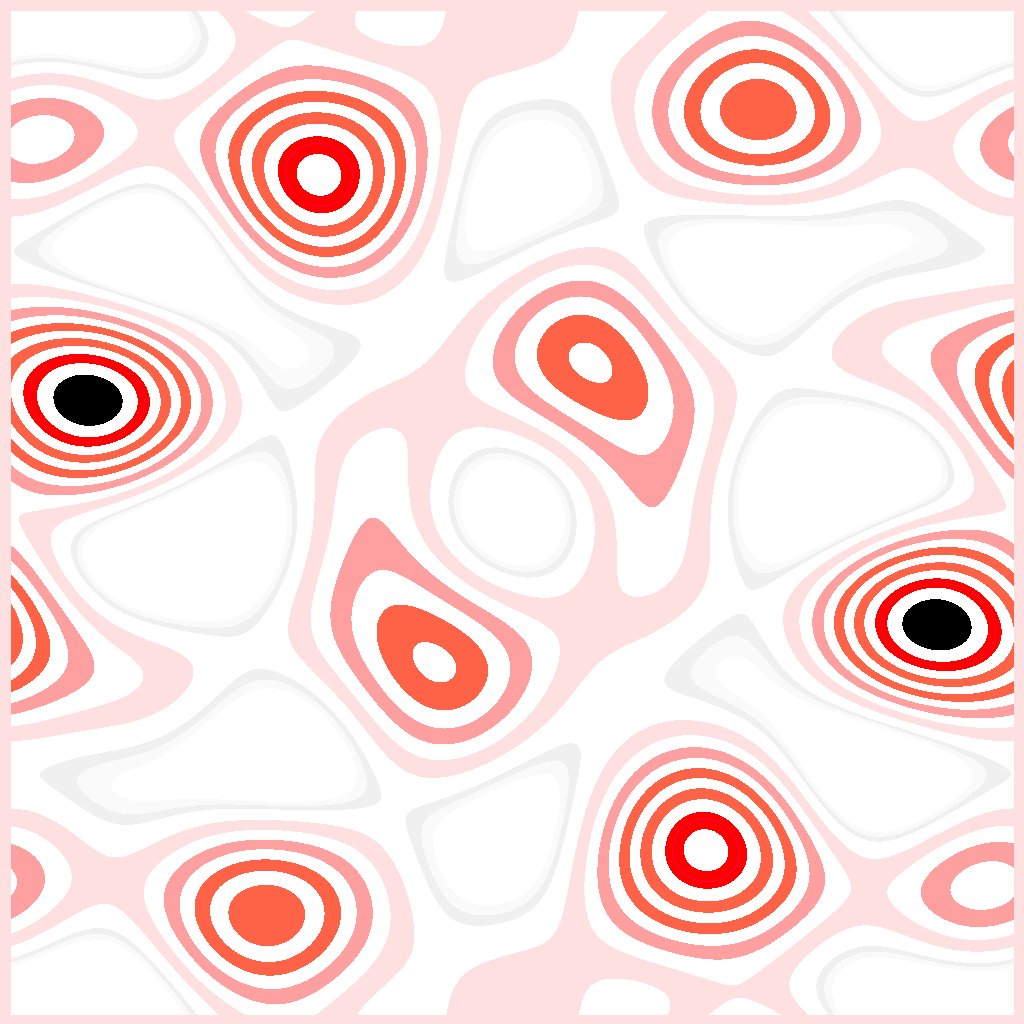

Supplement: Supplementary file 7 [file e-82-00534-sup8.zip › oi2035_SupportingMaterial/Example2/Example2 FOU Maps/Solution8 A=180 B=180 F=180 Fourier-Map_N22_32bit_gray_1024pix_LUT3.jpg]

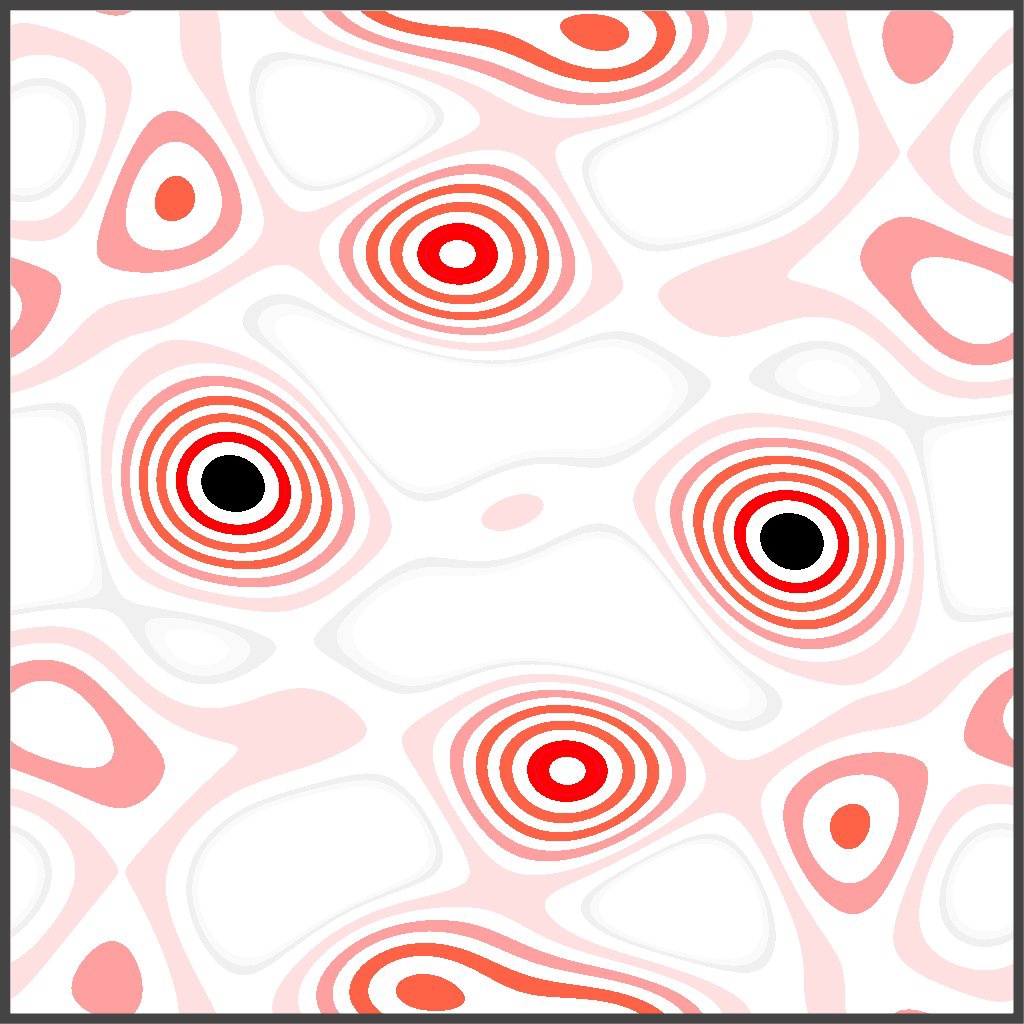

Supplement: Supplementary file 7 [file e-82-00534-sup8.zip › oi2035_SupportingMaterial/Example2/Example2 FOU Maps/Frame_Solution3 A=0 B=180 F=0 Fourier-Map_N22_32bit_gray_1024pix_LUT3.jpg]

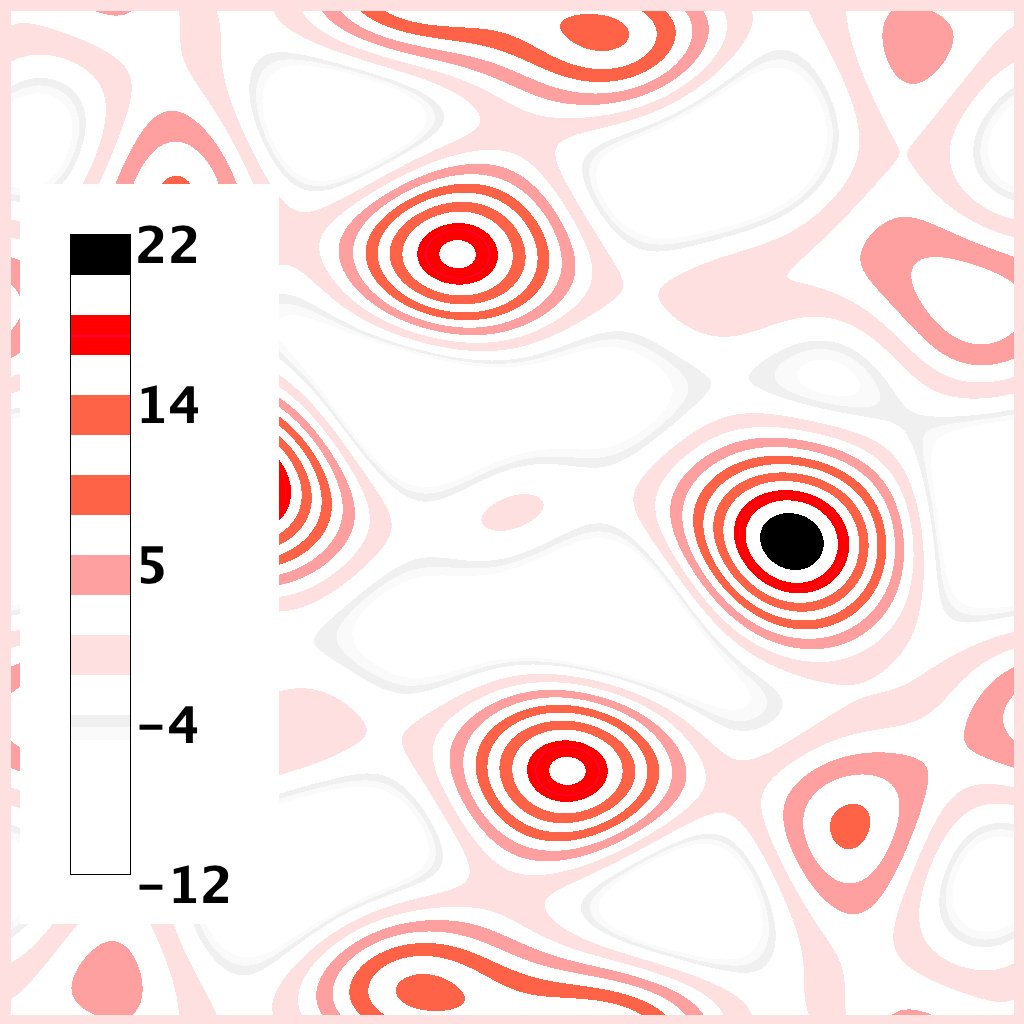

Supplement: Supplementary file 7 [file e-82-00534-sup8.zip › oi2035_SupportingMaterial/Example2/Example2 FOU Maps/Solution3 A=0 B=180 F=0 Fourier-Map_N22_32bit_gray_1024pix_ramp.jpg]

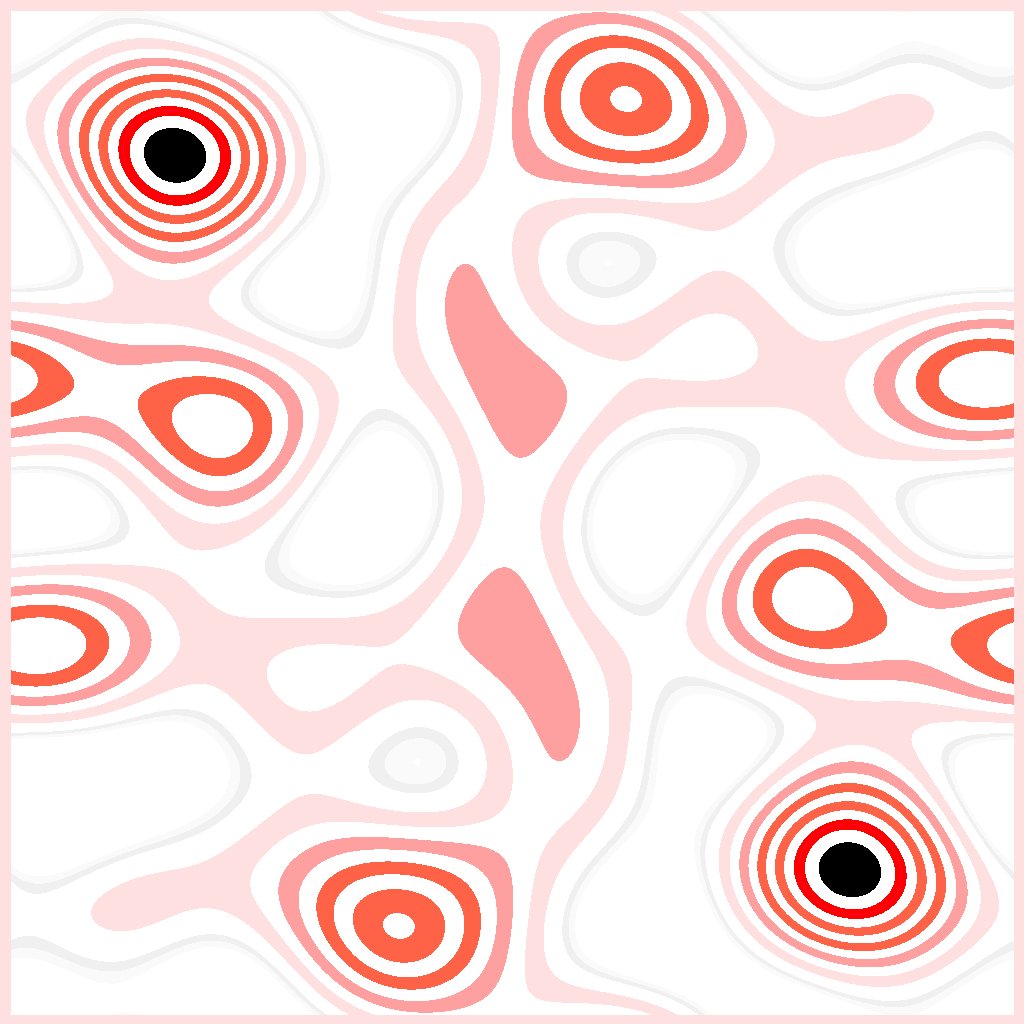

Supplement: Supplementary file 7 [file e-82-00534-sup8.zip › oi2035_SupportingMaterial/Example2/Example2 FOU Maps/Solution2 A=180 B=0 F=0 Fourier-Map_N22_32bit_gray_1024pix_LUT3.jpg]

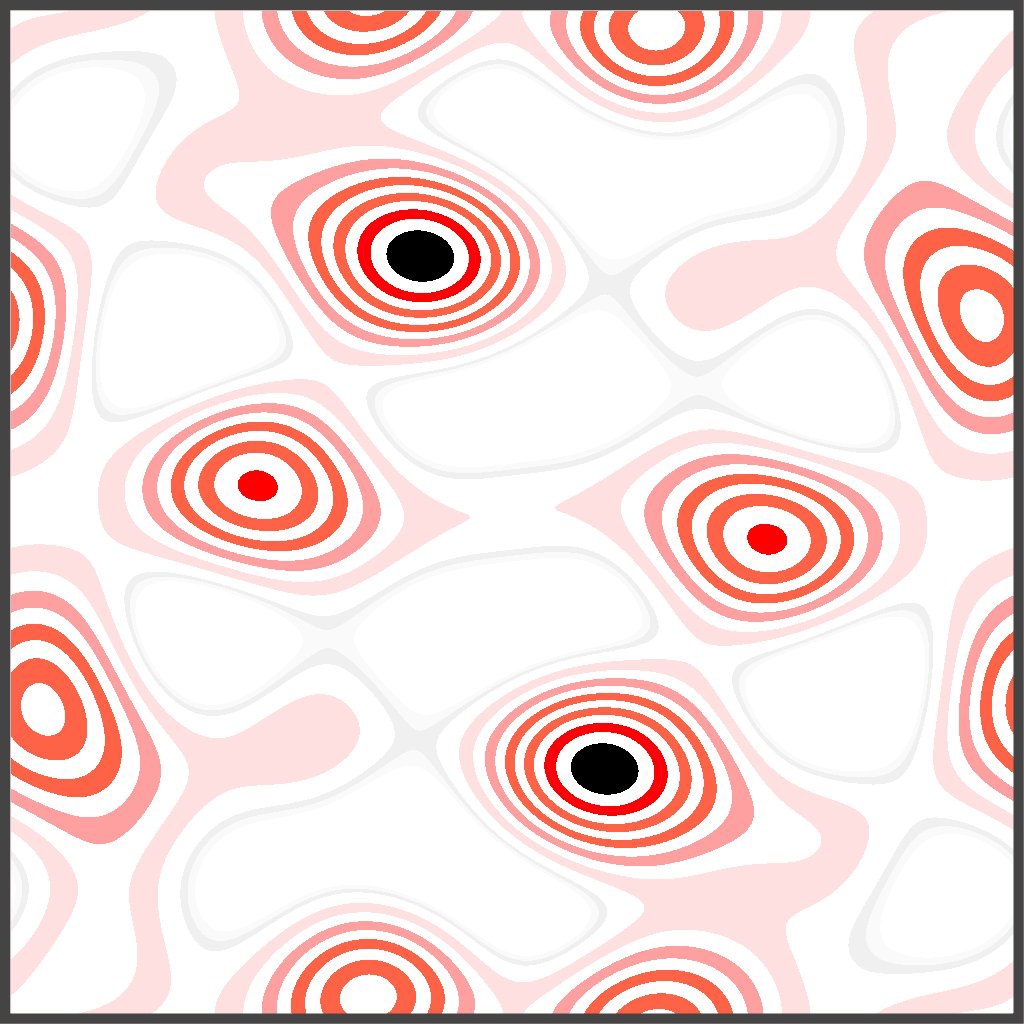

Supplement: Supplementary file 7 [file e-82-00534-sup8.zip › oi2035_SupportingMaterial/Example2/Example2 FOU Maps/Frame_Solution5 A=0 B=180 F=180 Fourier-Map_N22_32bit_gray_1024pix_LUT3.jpg]

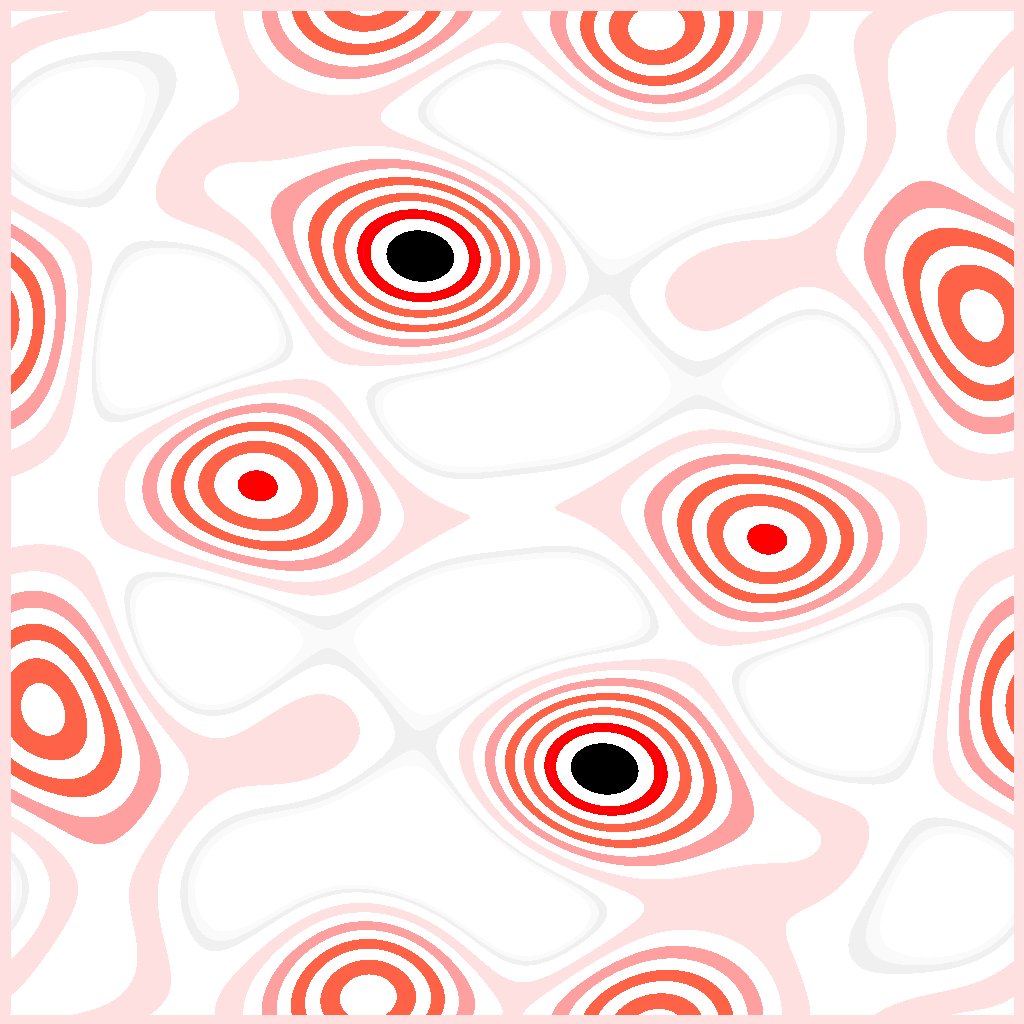

Supplement: Supplementary file 7 [file e-82-00534-sup8.zip › oi2035_SupportingMaterial/Example2/Example2 FOU Maps/Solution5 A=0 B=180 F=180 Fourier-Map_N22_32bit_gray_1024pix_LUT3.jpg]

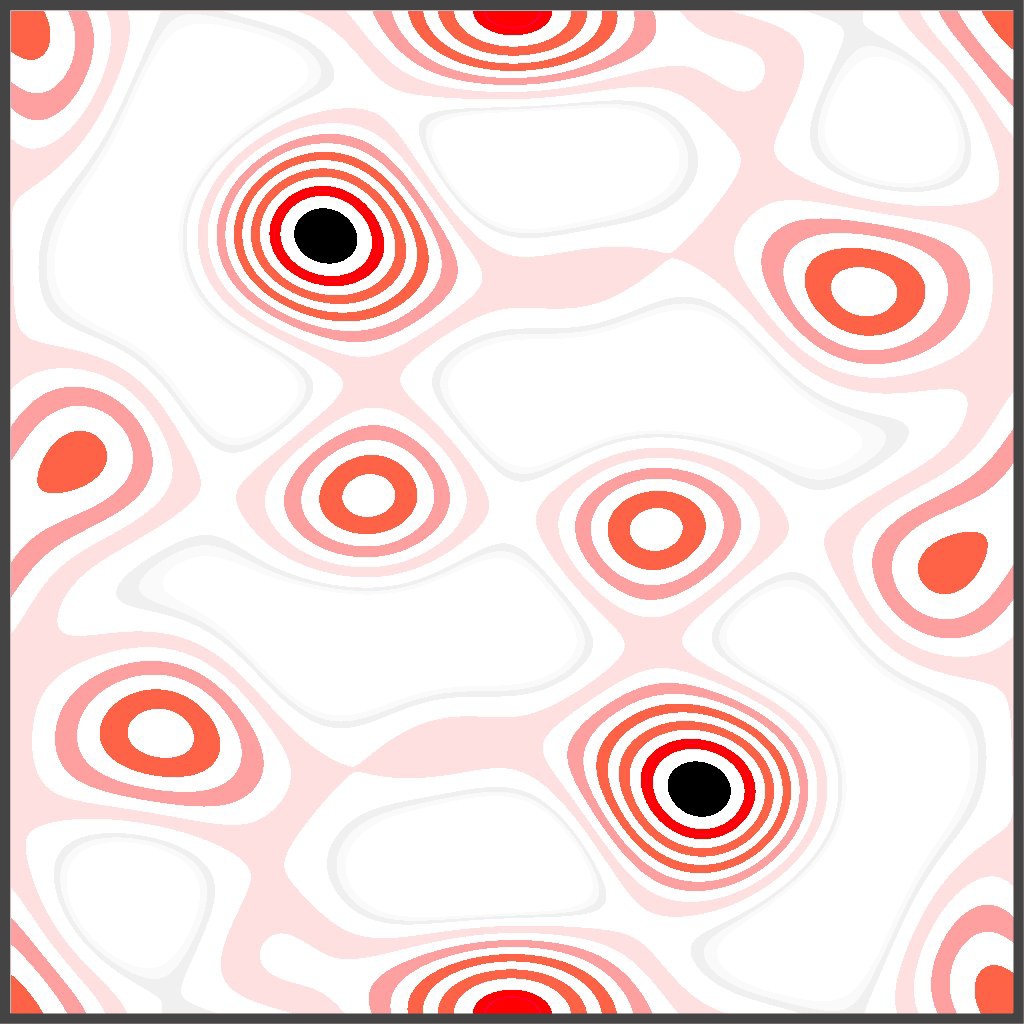

Supplement: Supplementary file 7 [file e-82-00534-sup8.zip › oi2035_SupportingMaterial/Example2/Example2 FOU Maps/Frame_Solution4 A=0 B=0 F=180 Fourier-Map_N22_32bit_gray_1024pix_LUT3.jpg]

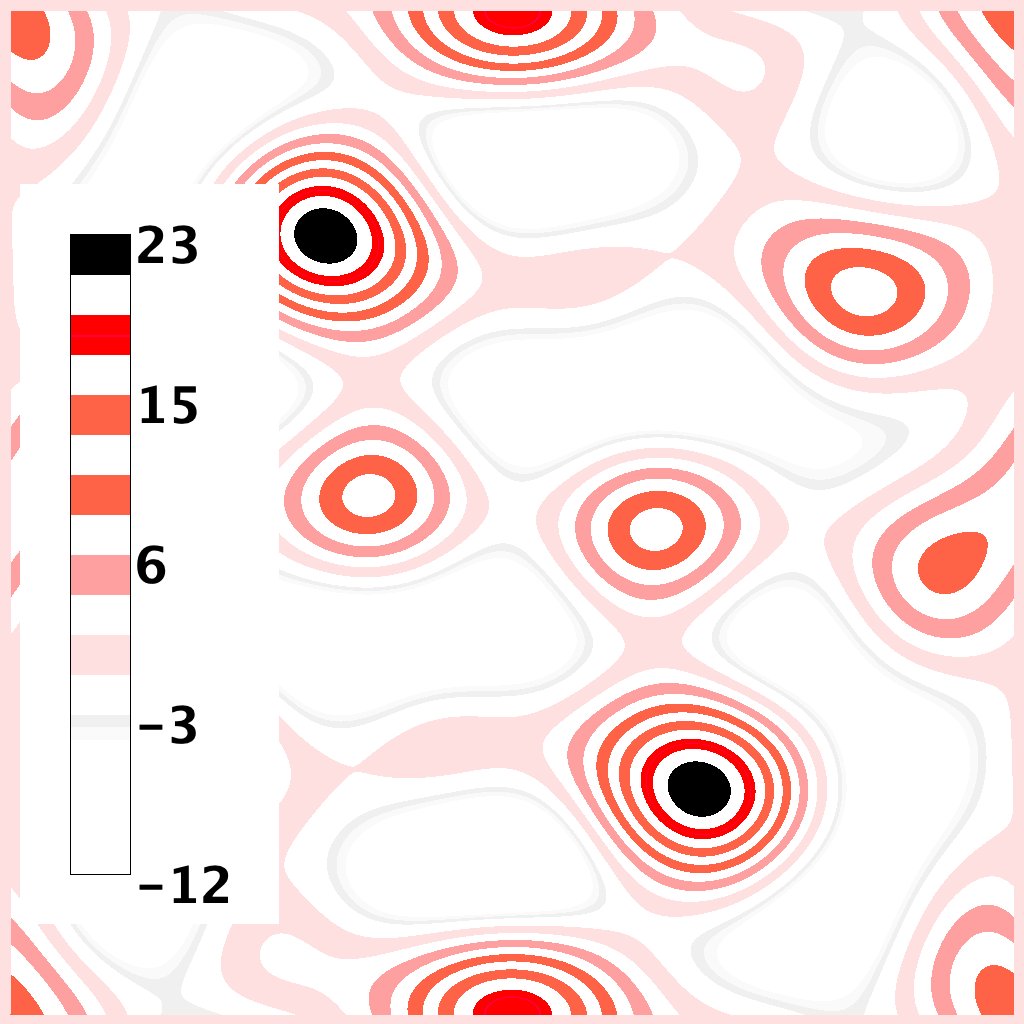

Supplement: Supplementary file 7 [file e-82-00534-sup8.zip › oi2035_SupportingMaterial/Example2/Example2 FOU Maps/Solution4 A=0 B=0 F=180 Fourier-Map_N22_32bit_gray_1024pix_ramp.jpg]

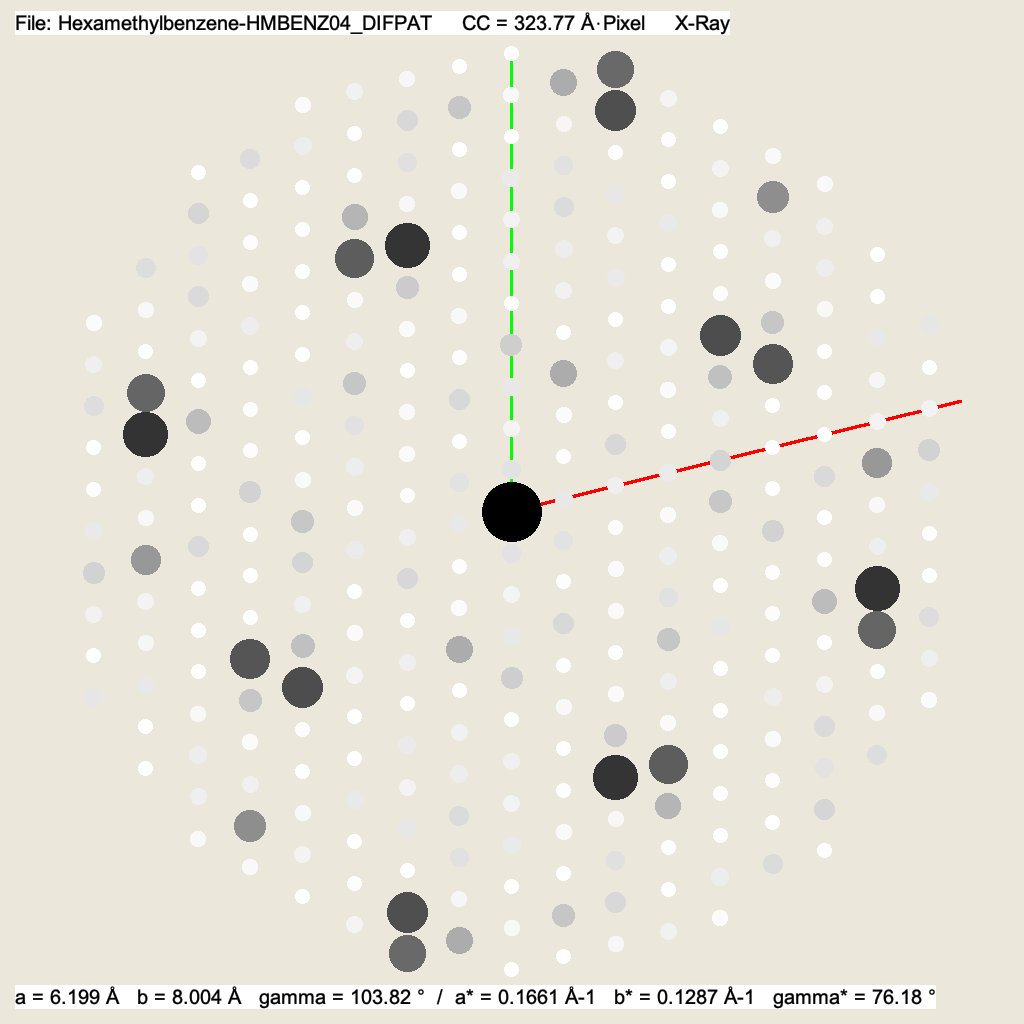

Supplement: Supplementary file 7 [file e-82-00534-sup8.zip › oi2035_SupportingMaterial/Example3/Example3 DISI-Kernel/Hexamethylbenzene-HMBENZ04_DIFPAT_CC=324A╠èΓïàPix_Plotsize1024Pix.jpg]

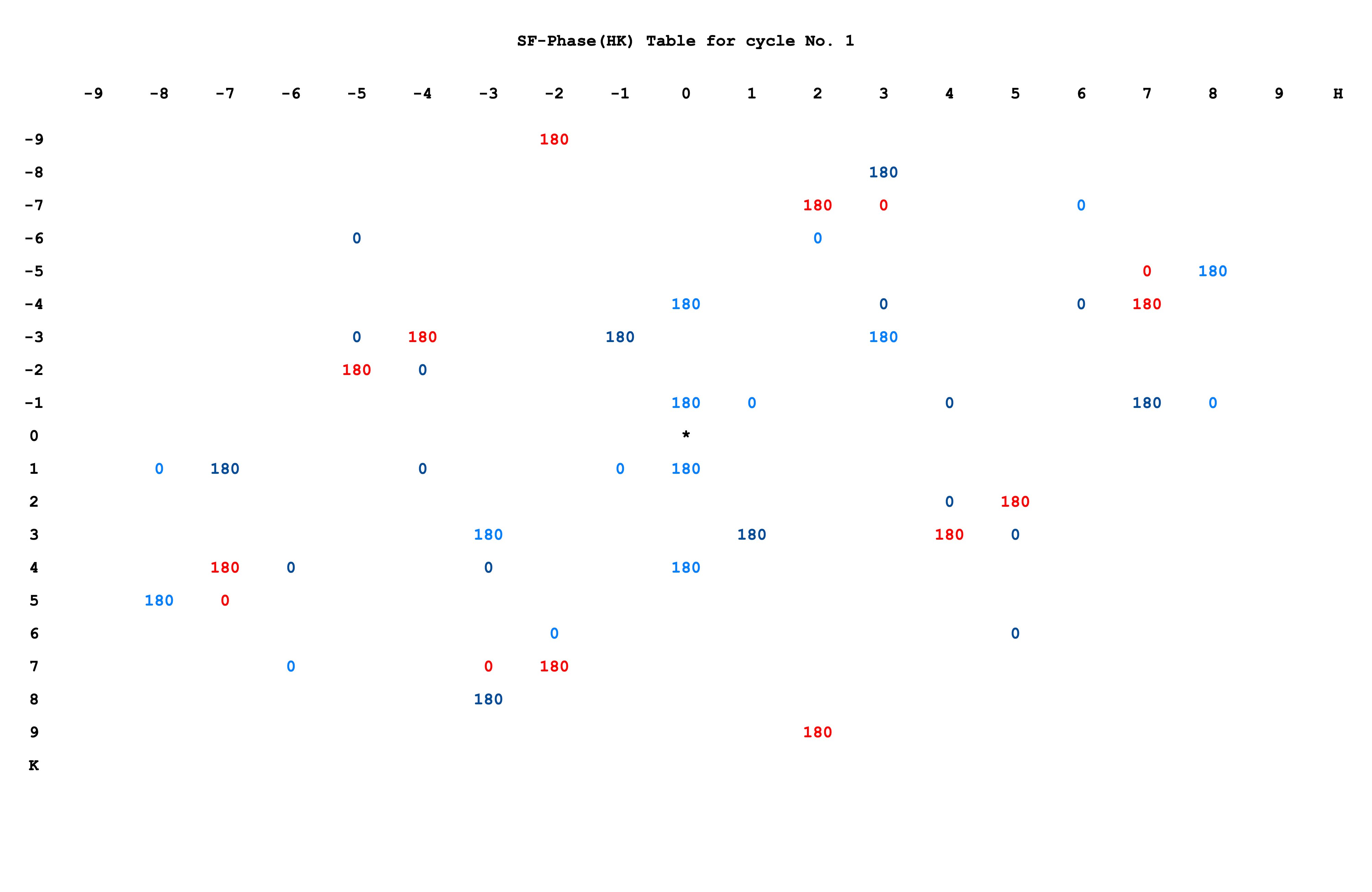

Supplement: Supplementary file 7 [file e-82-00534-sup8.zip › oi2035_SupportingMaterial/Example3/Example3 DISI-Kernel/1_SymbAddPhasing.jpg]

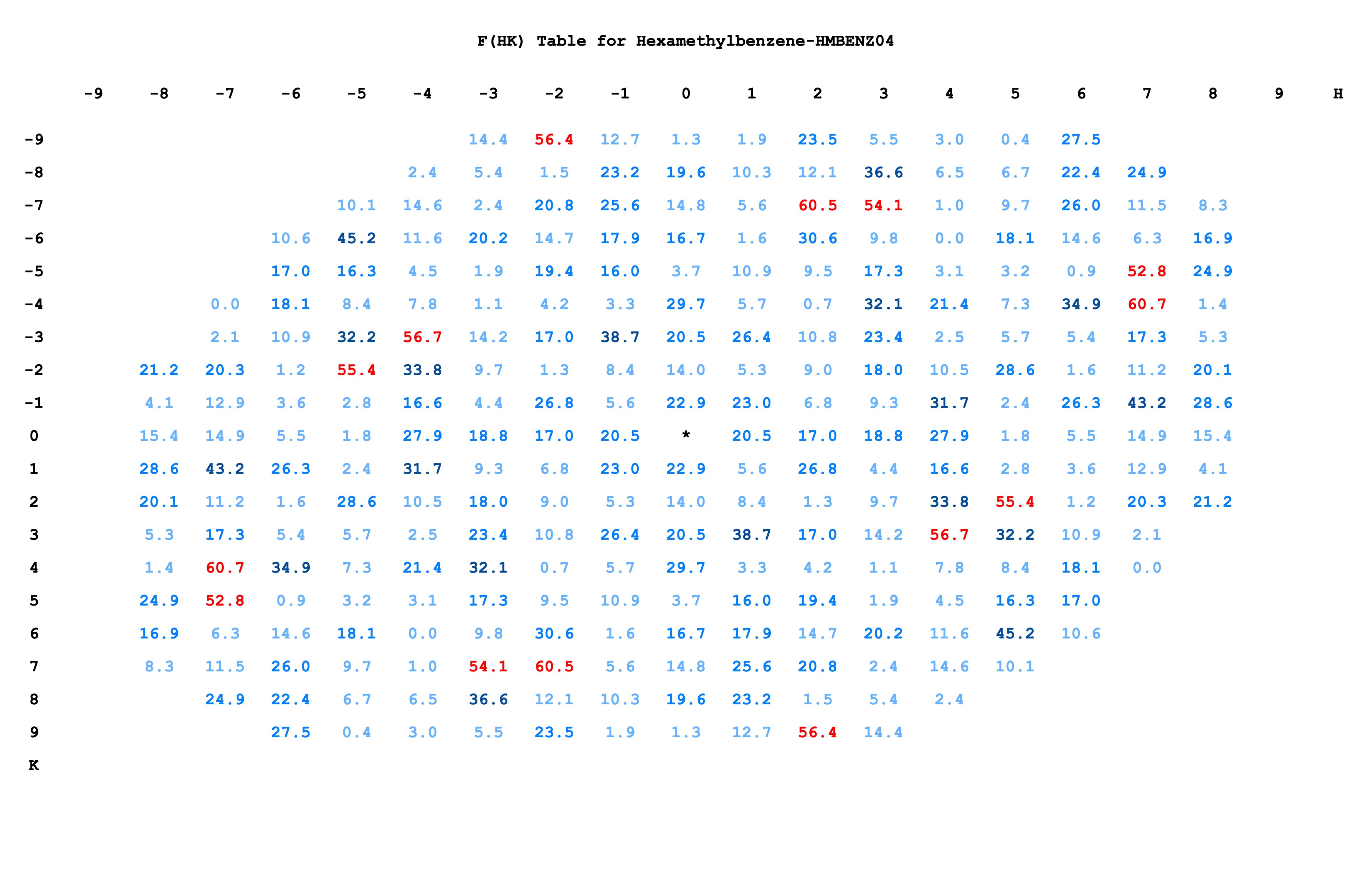

Supplement: Supplementary file 7 [file e-82-00534-sup8.zip › oi2035_SupportingMaterial/Example3/Example3 DISI-Kernel/Hexamethylbenzene-HMBENZ04_F(HK)-Table.jpg]

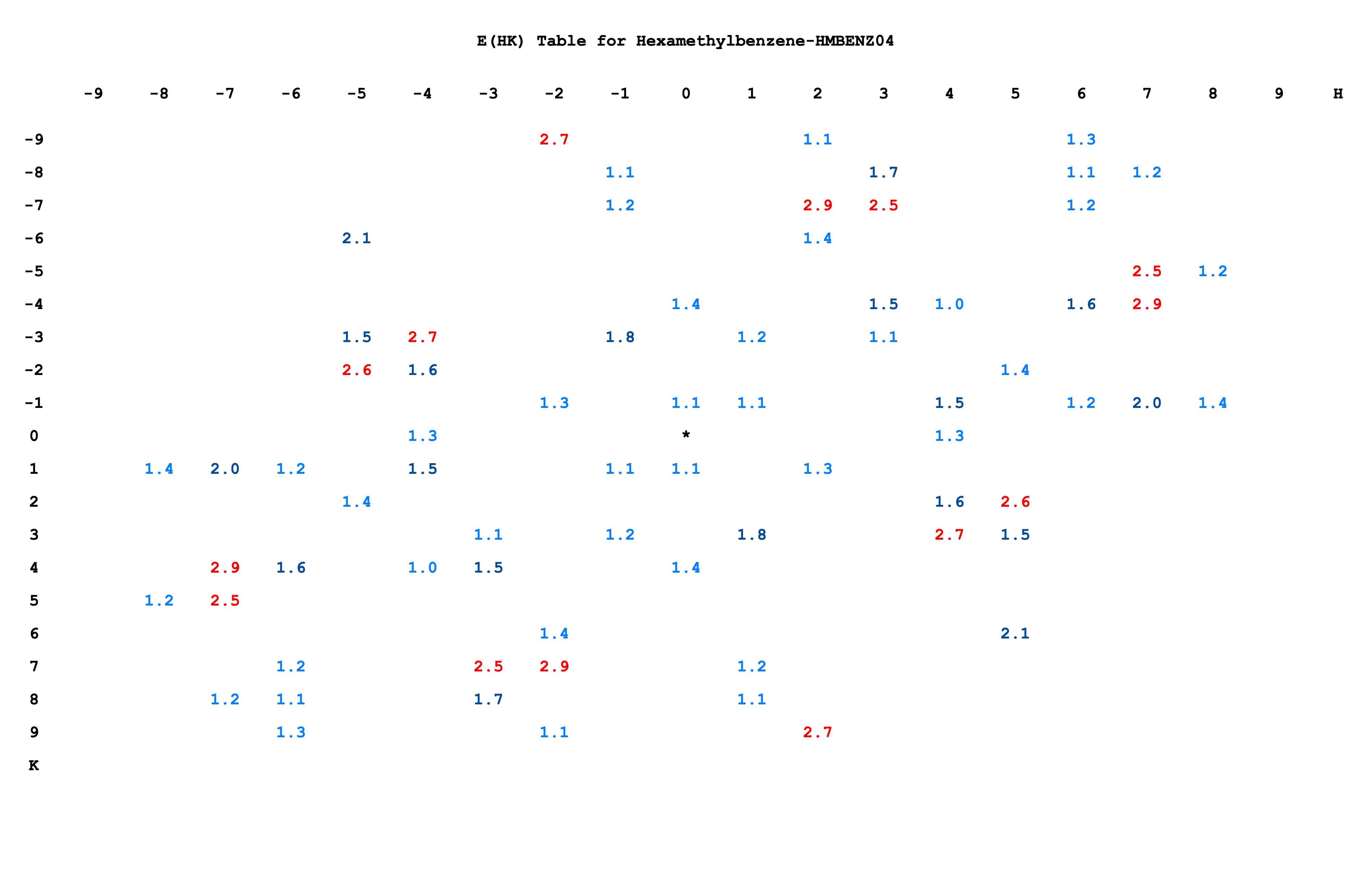

Supplement: Supplementary file 7 [file e-82-00534-sup8.zip › oi2035_SupportingMaterial/Example3/Example3 DISI-Kernel/Hexamethylbenzene-HMBENZ04_E(HK)-Table.jpg]

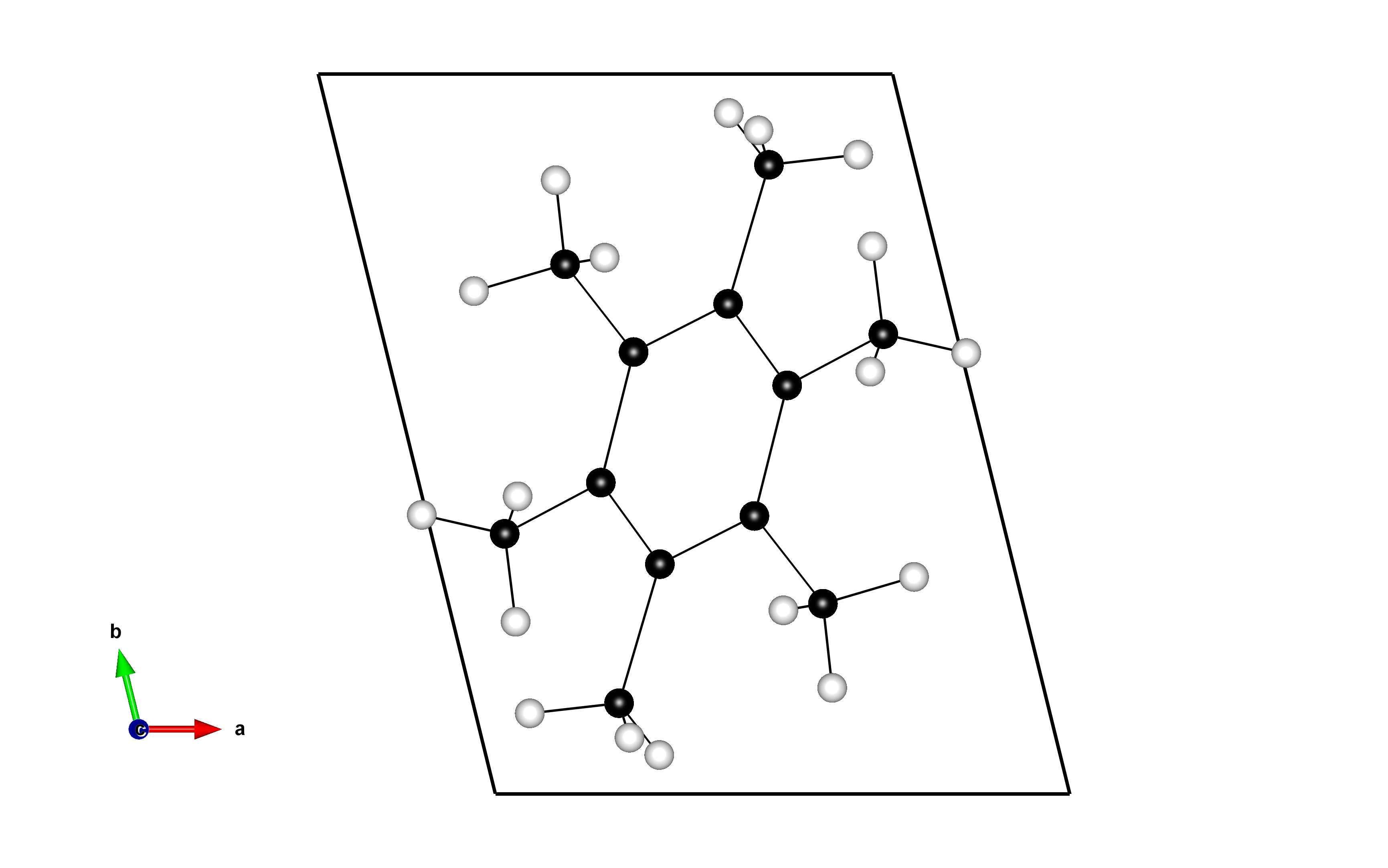

Supplement: Supplementary file 7 [file e-82-00534-sup8.zip › oi2035_SupportingMaterial/Example3/Example3 DISI-Kernel/Hexamethylbenzene-HMBENZ04_CIF model.png]

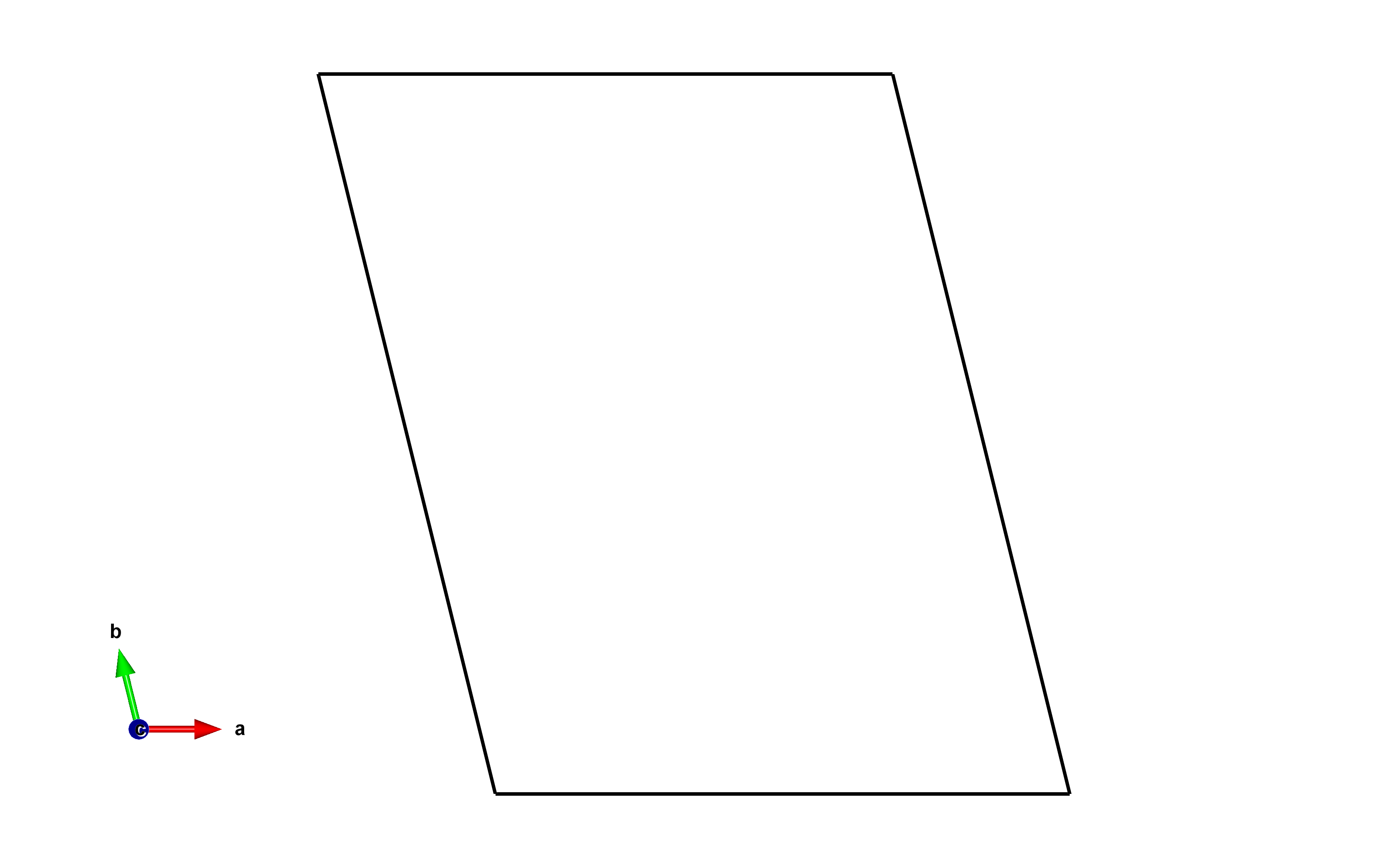

Supplement: Supplementary file 7 [file e-82-00534-sup8.zip › oi2035_SupportingMaterial/Example3/Example3 DISI-Kernel/Hexamethylbenzene-HMBENZ04_CIF unit cell.png]

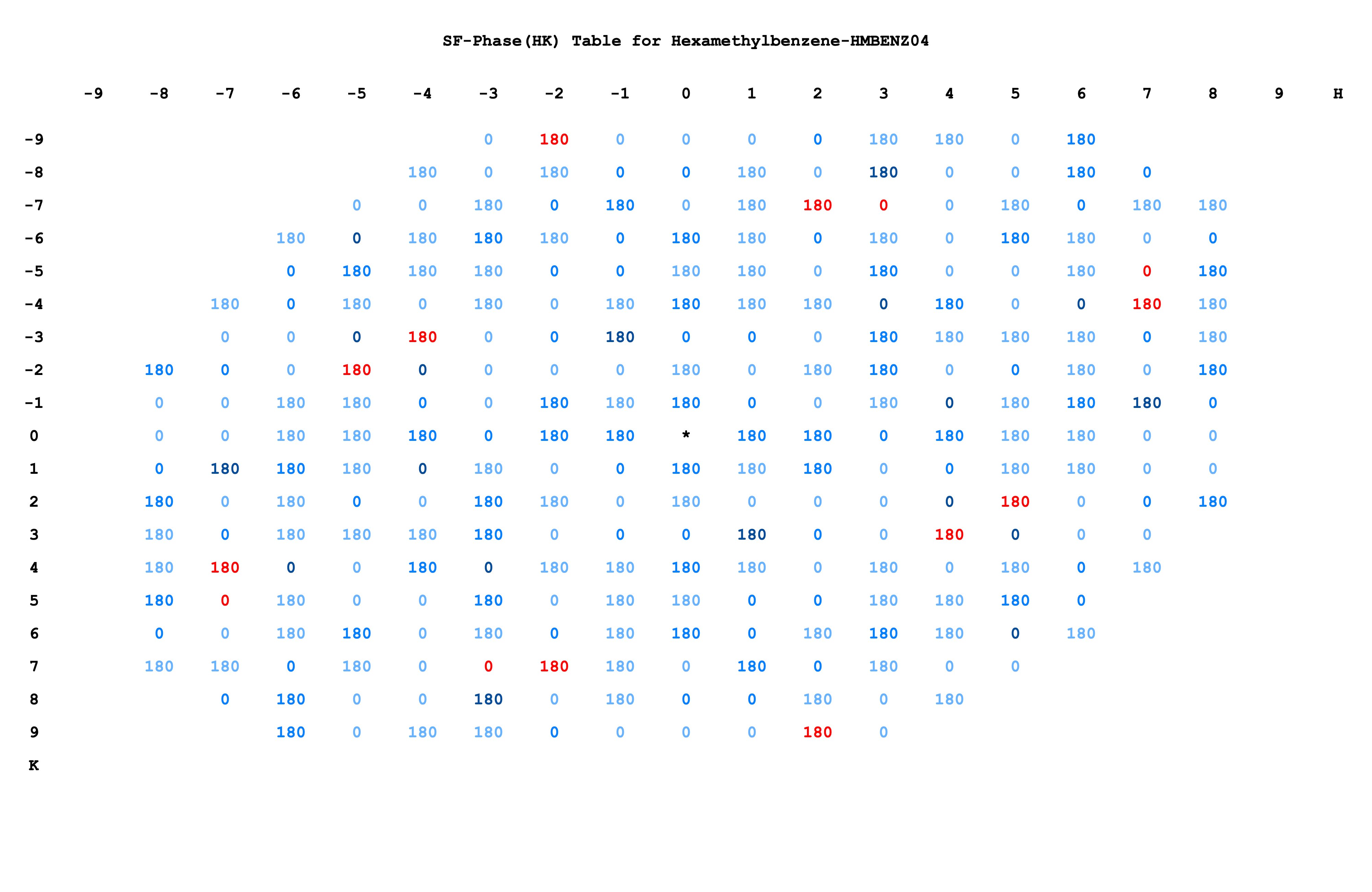

Supplement: Supplementary file 7 [file e-82-00534-sup8.zip › oi2035_SupportingMaterial/Example3/Example3 DISI-Kernel/Hexamethylbenzene-HMBENZ04_SF-Phase(HK)-Table.jpg]

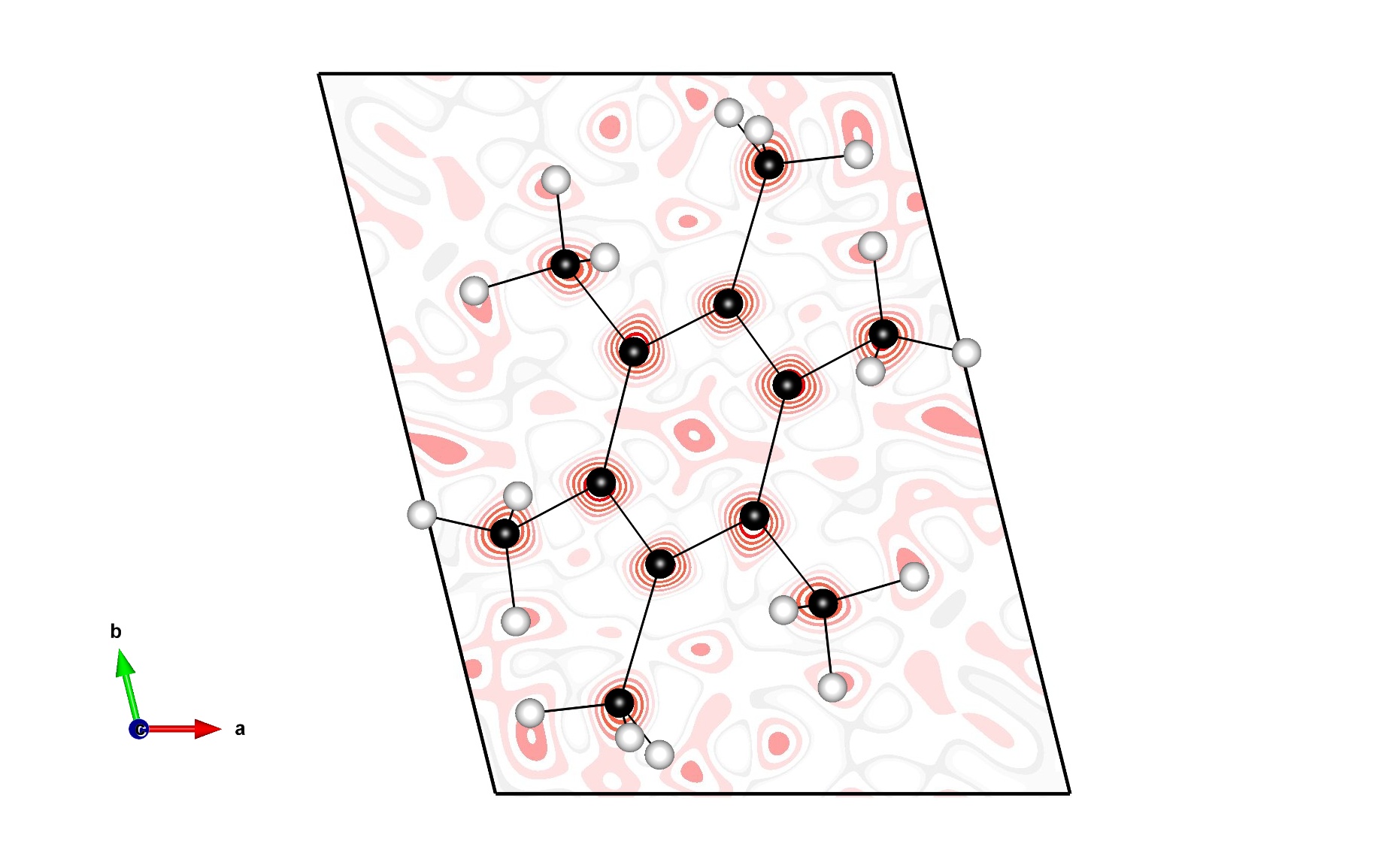

Supplement: Supplementary file 7 [file e-82-00534-sup8.zip › oi2035_SupportingMaterial/Example3/Example3 Model/Hexamethylbenzene-HMBENZ04_EFOU Fourier-Map_N82_32bit_gray_1024pix_LUT3_overlay.jpg]

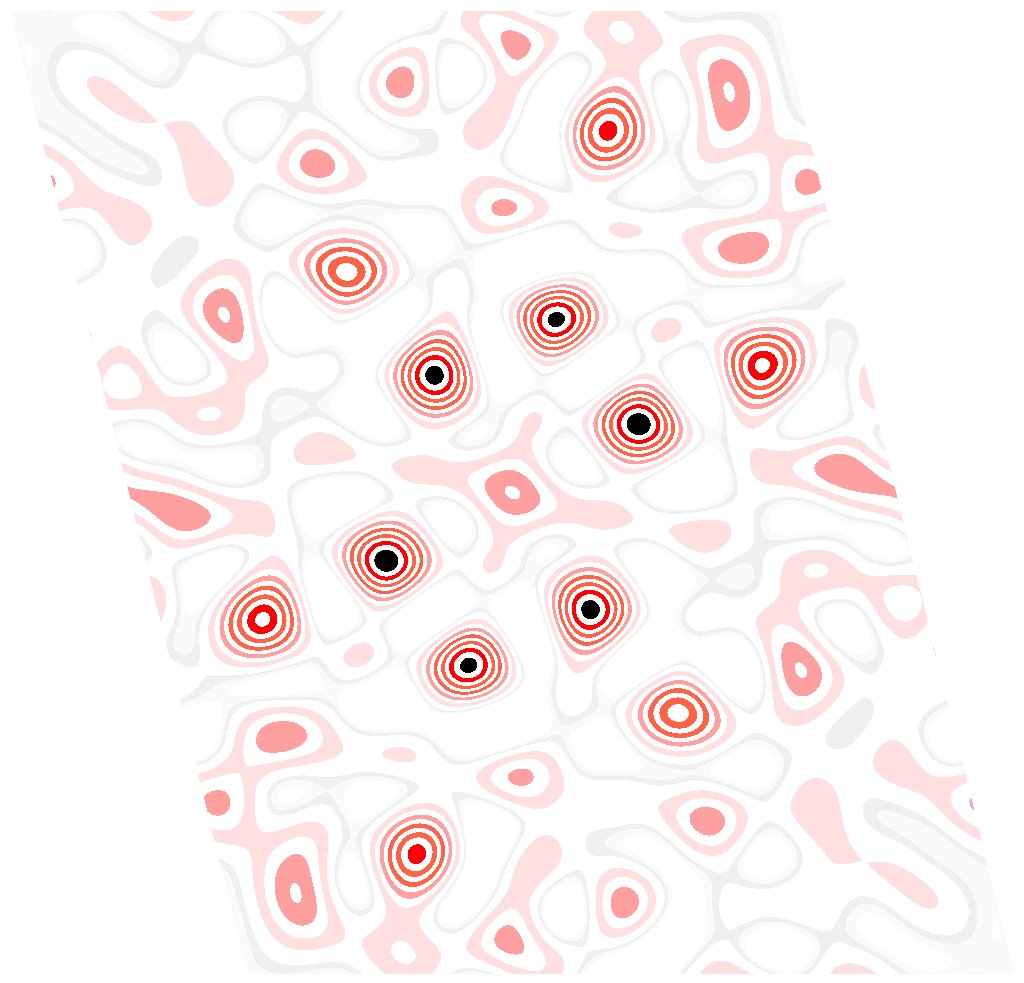

Supplement: Supplementary file 7 [file e-82-00534-sup8.zip › oi2035_SupportingMaterial/Example3/Example3 Model/Hexamethylbenzene-HMBENZ04_EFOU Fourier-Map_N82_32bit_gray_1024pix_LUT3.jpg]

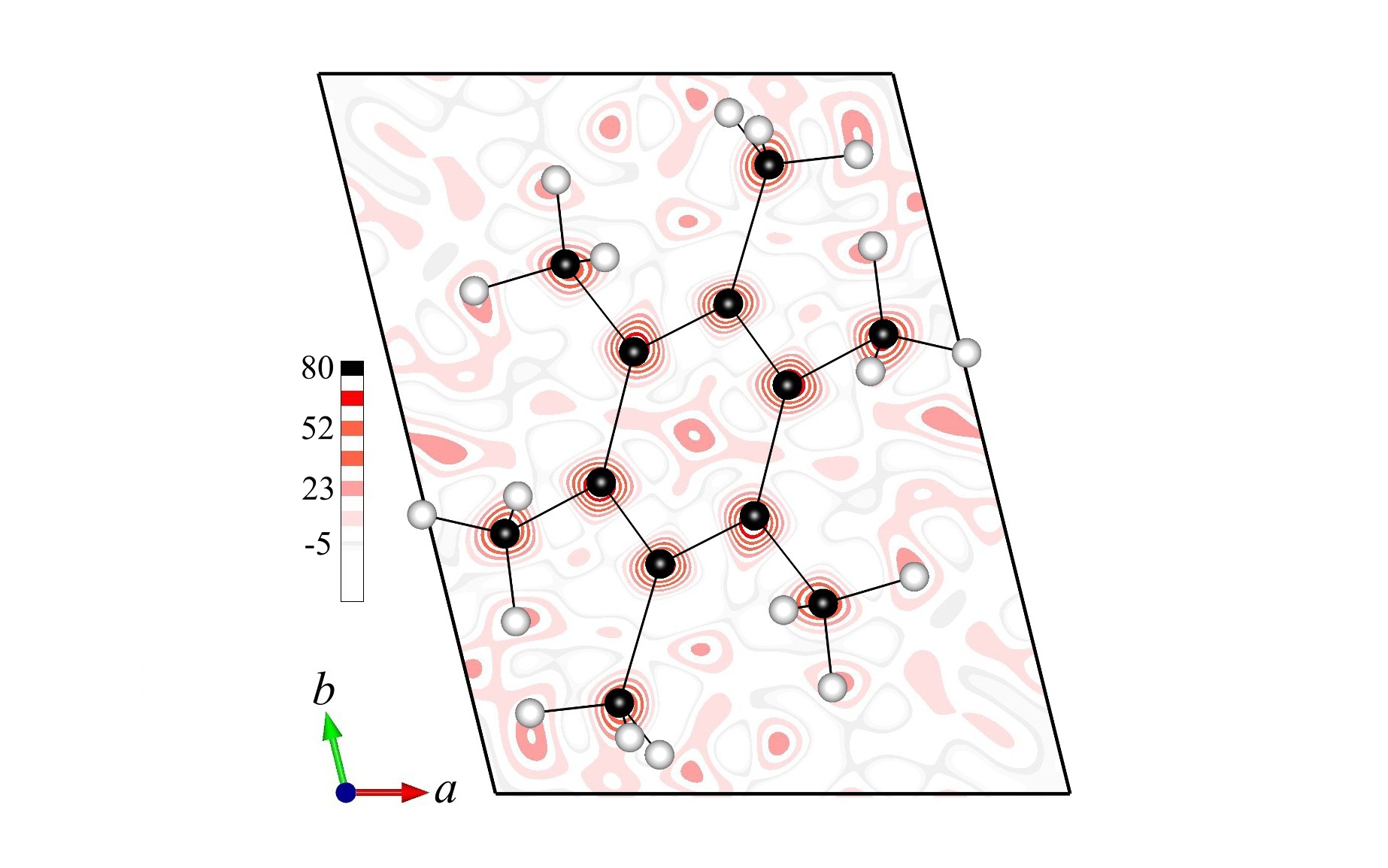

Supplement: Supplementary file 7 [file e-82-00534-sup8.zip › oi2035_SupportingMaterial/Example3/Example3 Model/Hexamethylbenzene-HMBENZ04_EFOU Fourier-Map_axes_Intrange.jpg]

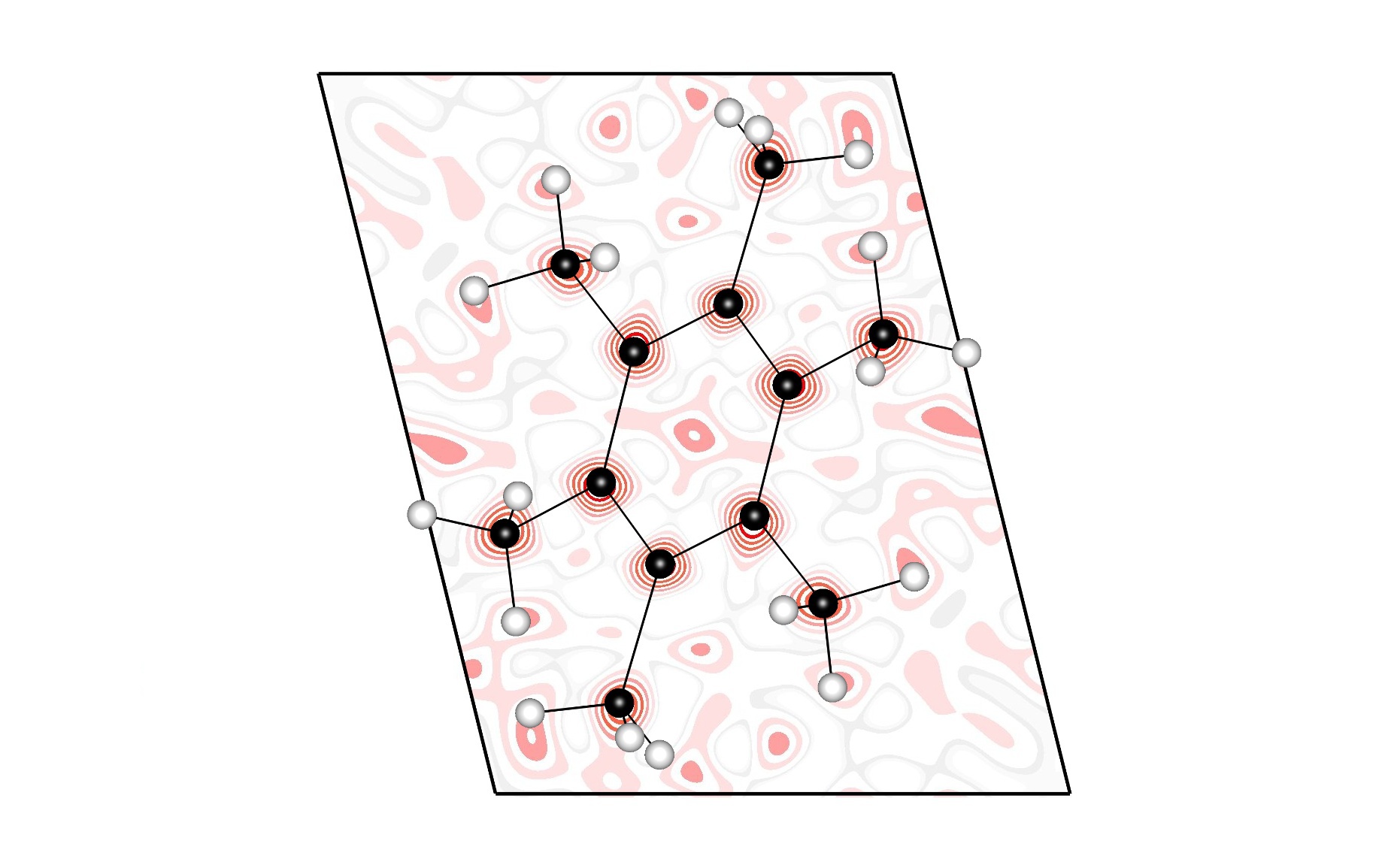

Supplement: Supplementary file 7 [file e-82-00534-sup8.zip › oi2035_SupportingMaterial/Example3/Example3 Model/Hexamethylbenzene-HMBENZ04_EFOU Fourier-Map_N82_32bit_gray_1024pix_LUT3_overlay_clean.jpg]

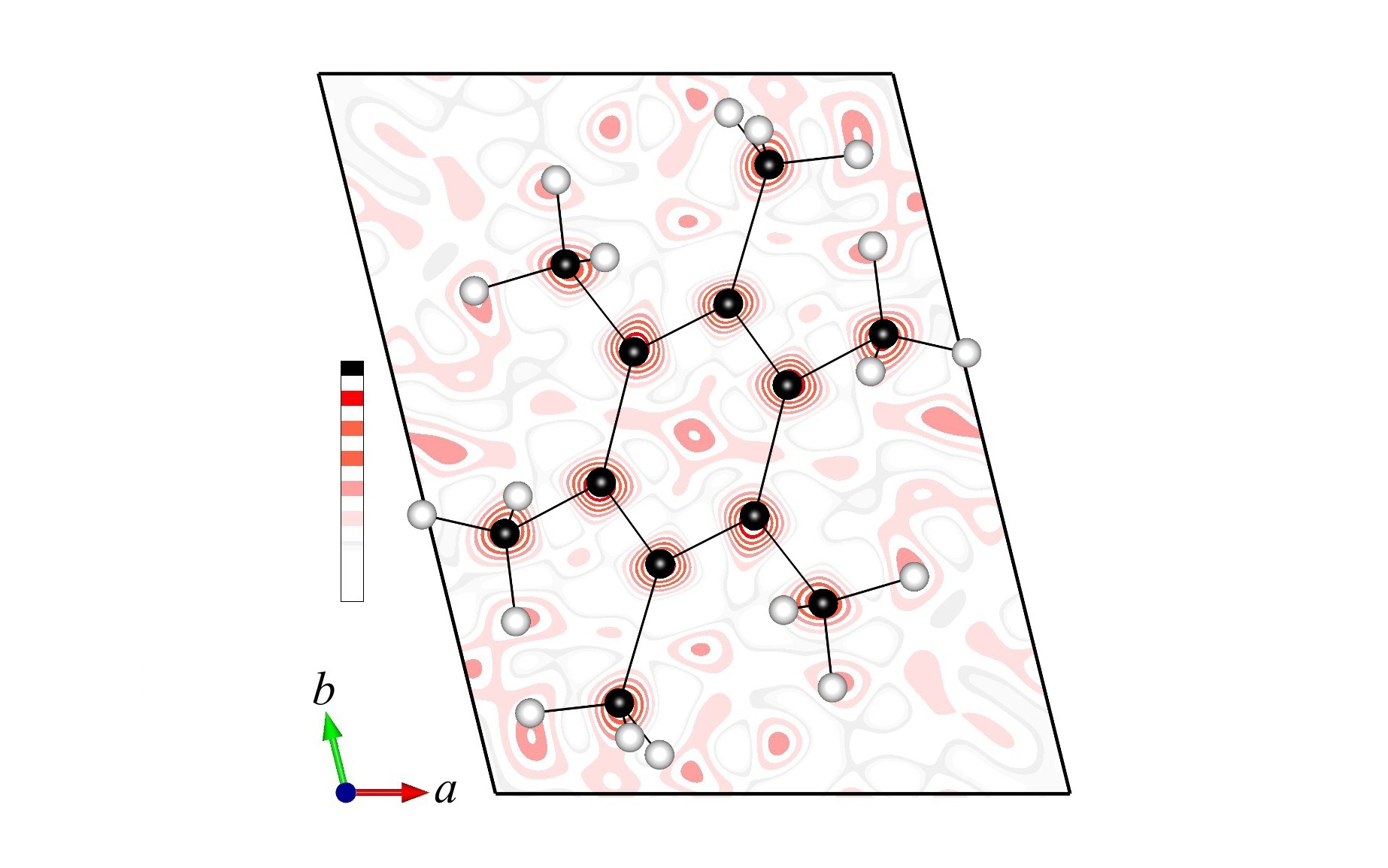

Supplement: Supplementary file 7 [file e-82-00534-sup8.zip › oi2035_SupportingMaterial/Example3/Example3 Model/Hexamethylbenzene-HMBENZ04_EFOU Fourier-Map_axes.jpg]

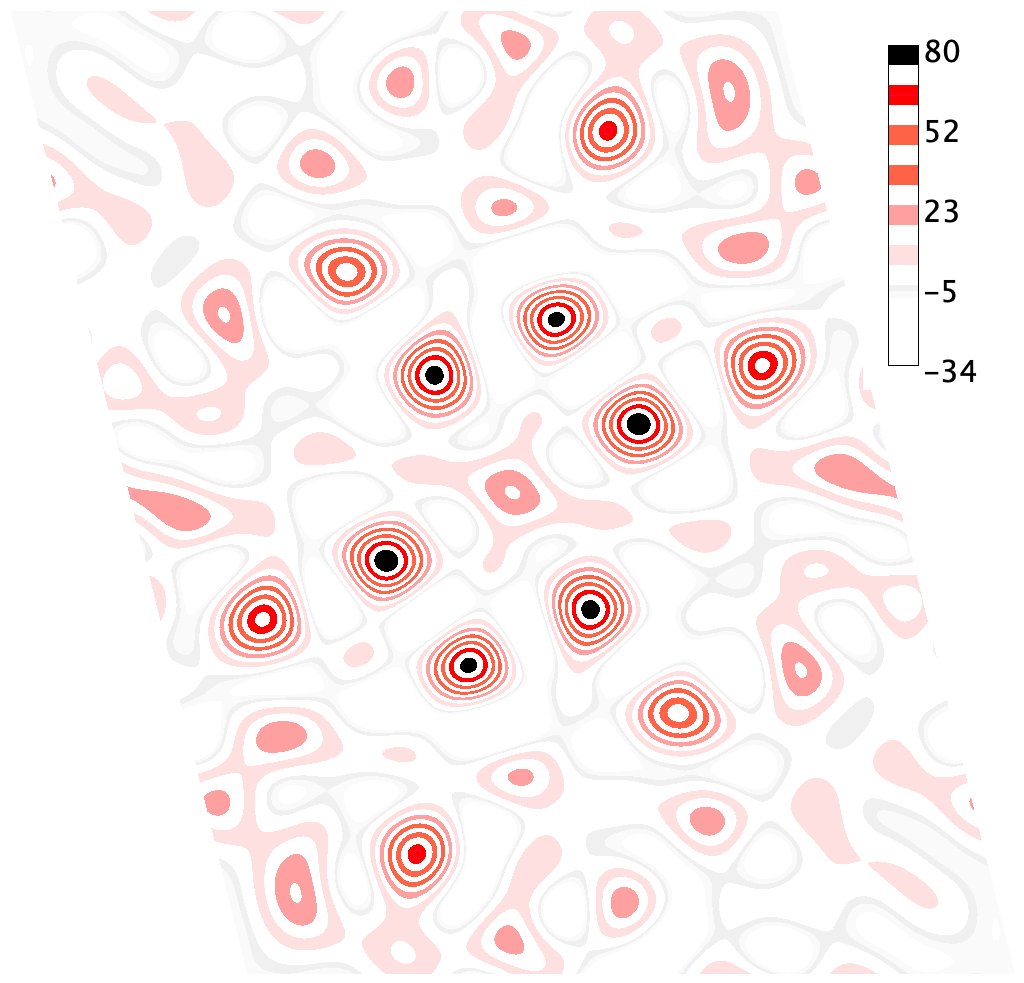

Supplement: Supplementary file 7 [file e-82-00534-sup8.zip › oi2035_SupportingMaterial/Example3/Example3 Model/Hexamethylbenzene-HMBENZ04_EFOU Fourier-Map_N82_32bit_gray_1024pix_ramp.jpg]

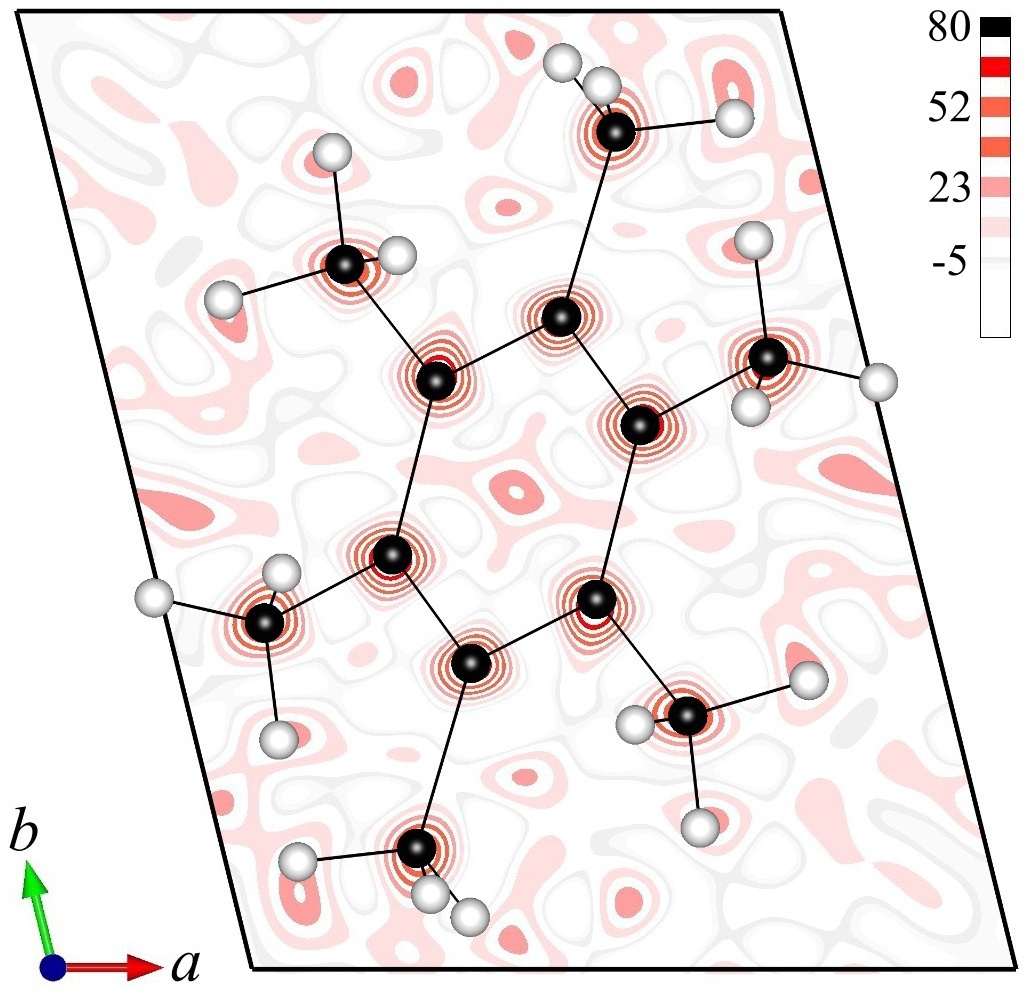

Supplement: Supplementary file 7 [file e-82-00534-sup8.zip › oi2035_SupportingMaterial/Example3/Example3 Model/Hexamethylbenzene-HMBENZ04_EFOU Fourier-Map_model.jpg]

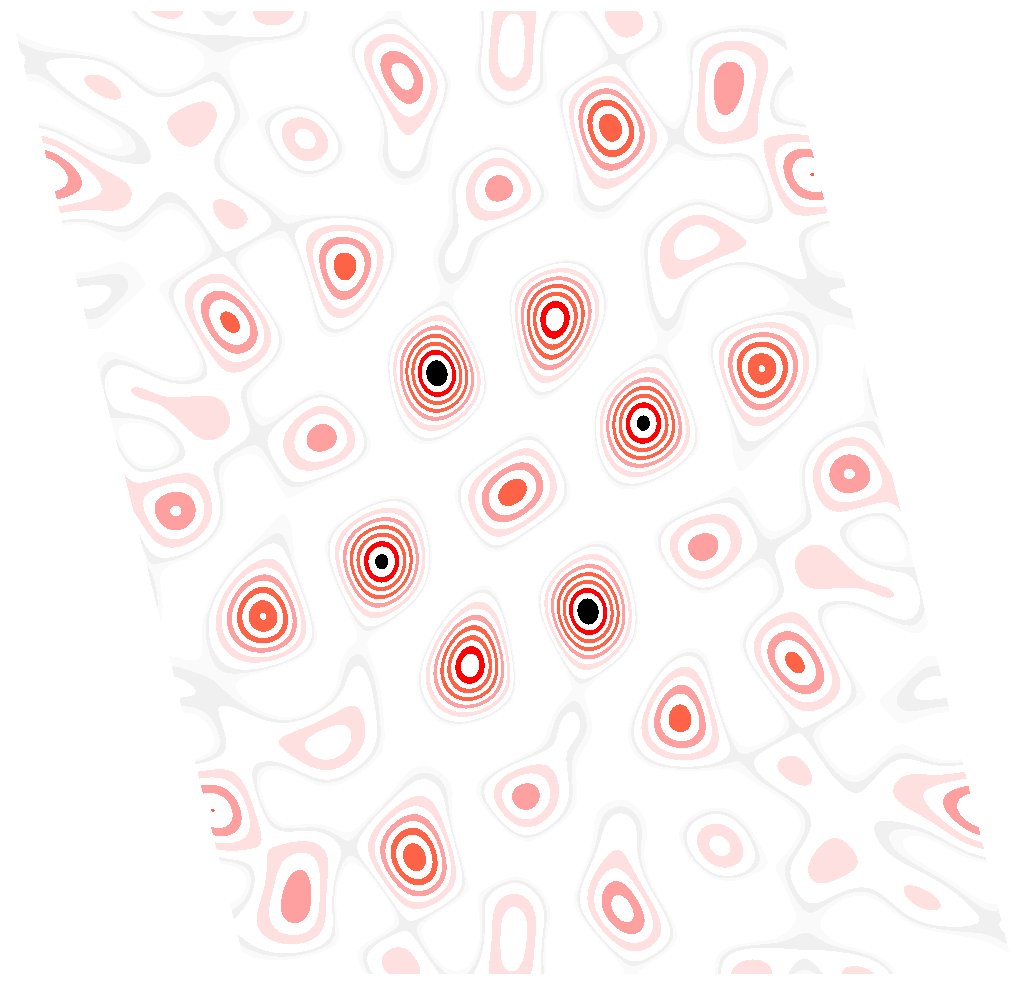

Supplement: Supplementary file 7 [file e-82-00534-sup8.zip › oi2035_SupportingMaterial/Example3/Example3 FOU Maps/S3 A=0 C=0 D=180 S=0 EFOU Fourier-Map_N48_32bit_gray_1024pix_LUT3.jpg]

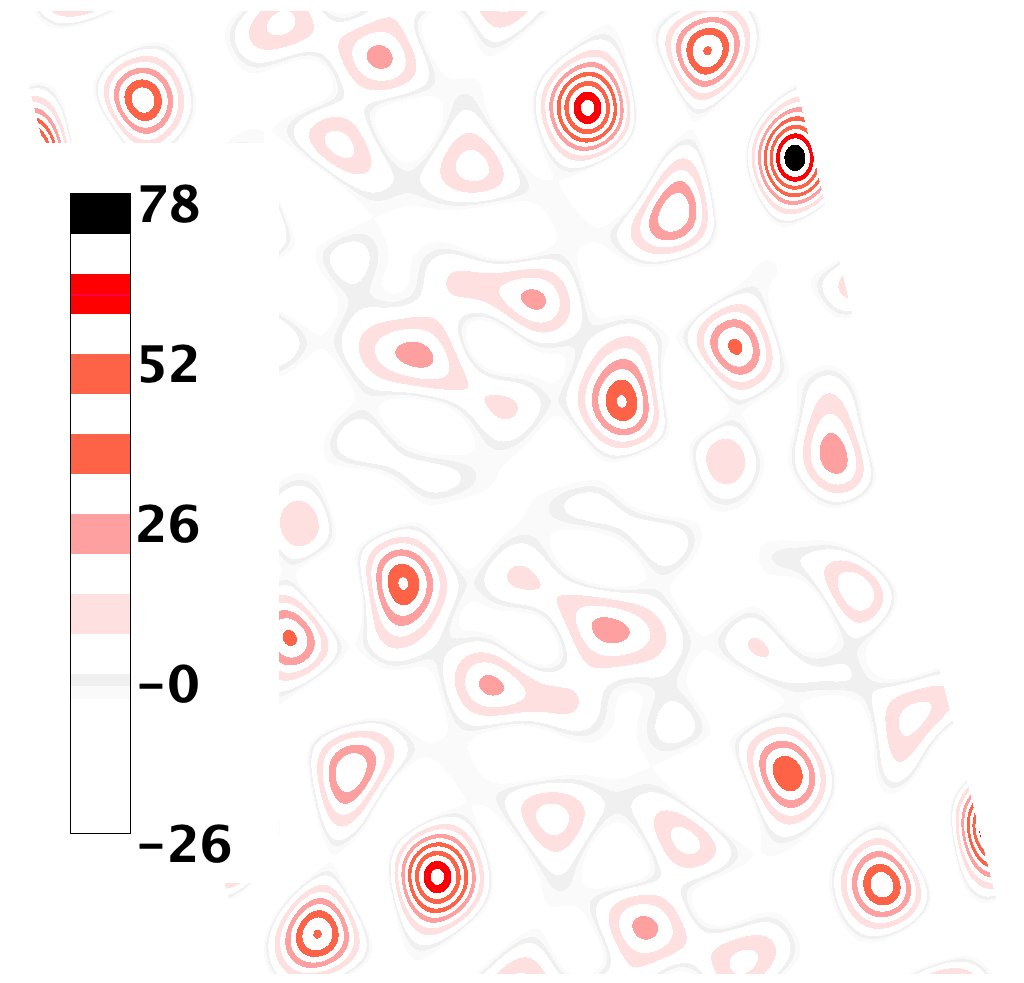

Supplement: Supplementary file 7 [file e-82-00534-sup8.zip › oi2035_SupportingMaterial/Example3/Example3 FOU Maps/S13 A=180 C=0 D=180 S=180 EFOU Fourier-Map_N48_32bit_gray_1024pix_ramp.jpg]

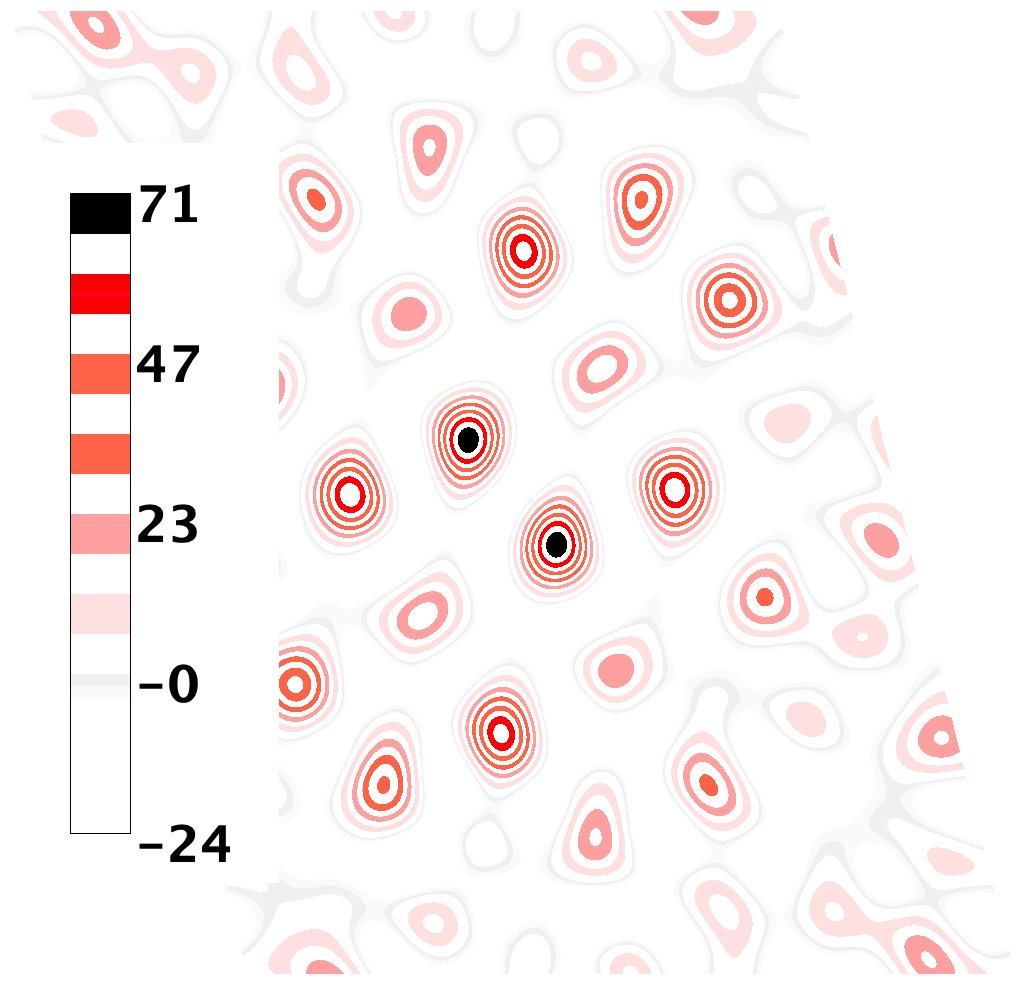

Supplement: Supplementary file 7 [file e-82-00534-sup8.zip › oi2035_SupportingMaterial/Example3/Example3 FOU Maps/S7 A=180 C=180 D=0 S=0 EFOU Fourier-Map_N48_32bit_gray_1024pix_ramp.jpg]

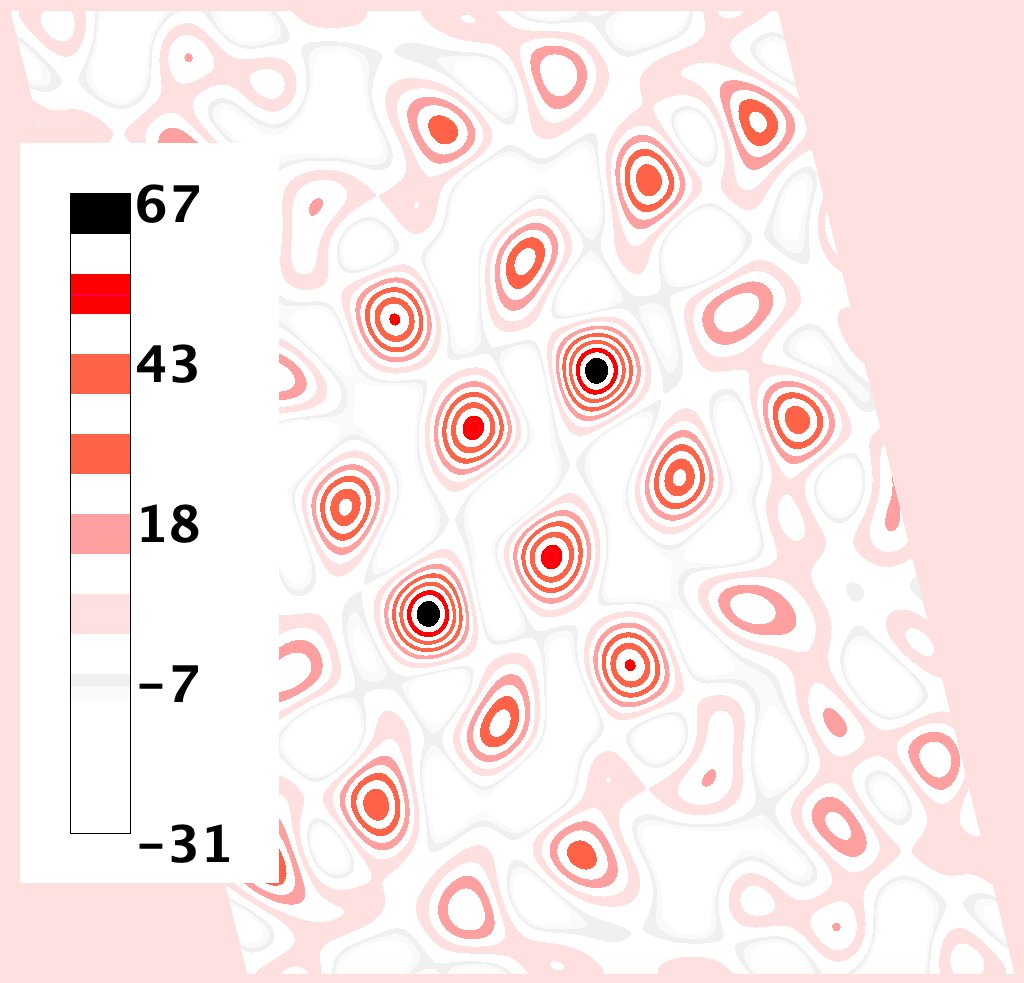

Supplement: Supplementary file 7 [file e-82-00534-sup8.zip › oi2035_SupportingMaterial/Example3/Example3 FOU Maps/S15 A=180 C=180 D=180 S=0 EFOU Fourier-Map_N48_32bit_gray_1024pix_ramp.jpg]

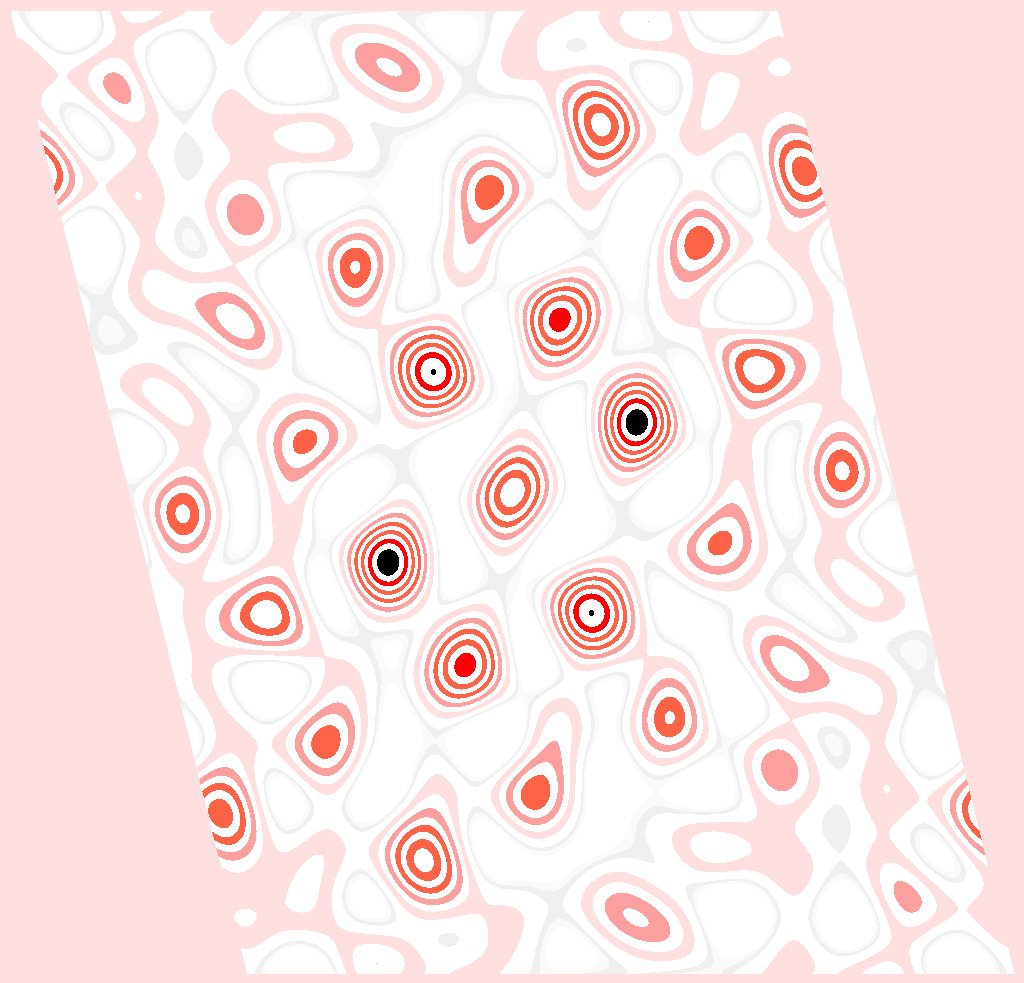

Supplement: Supplementary file 7 [file e-82-00534-sup8.zip › oi2035_SupportingMaterial/Example3/Example3 FOU Maps/S6 A=0 C=0 D=180 S=180 EFOU Fourier-Map_N48_32bit_gray_1024pix_LUT3.jpg]

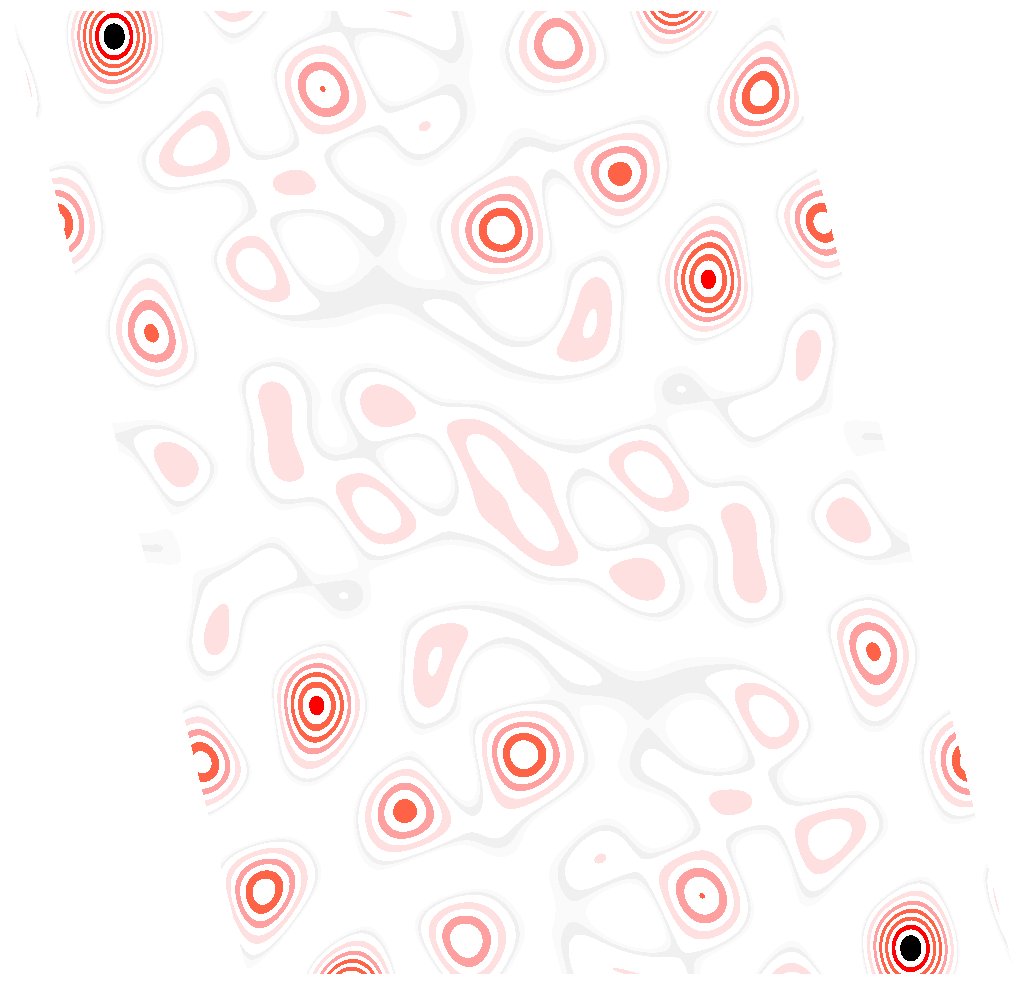

Supplement: Supplementary file 7 [file e-82-00534-sup8.zip › oi2035_SupportingMaterial/Example3/Example3 FOU Maps/S9 A=0 C=180 D=0 S=180 EFOU Fourier-Map_N48_32bit_gray_1024pix_LUT3.jpg]

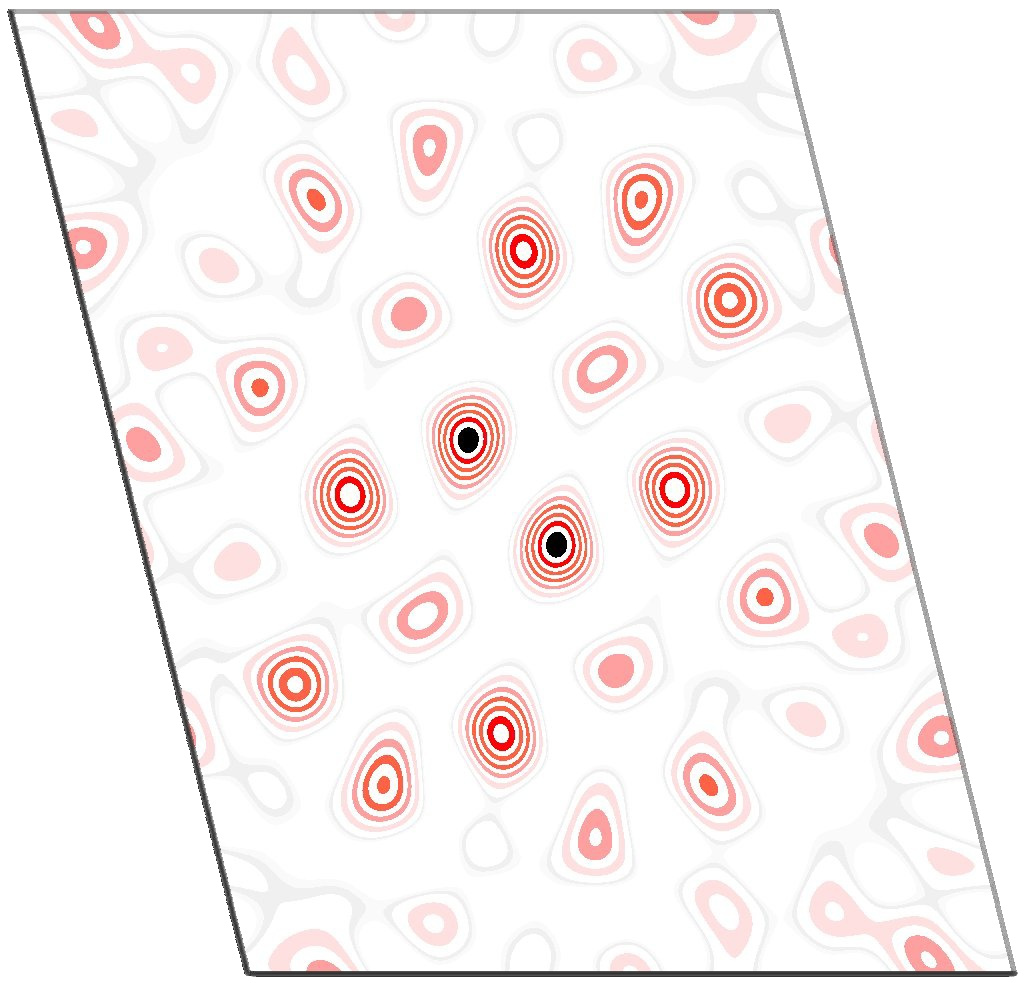

Supplement: Supplementary file 7 [file e-82-00534-sup8.zip › oi2035_SupportingMaterial/Example3/Example3 FOU Maps/Frame_S7 A=180 C=180 D=0 S=0 EFOU Fourier-Map_N48_32bit_gray_1024pix_LUT3.jpg]

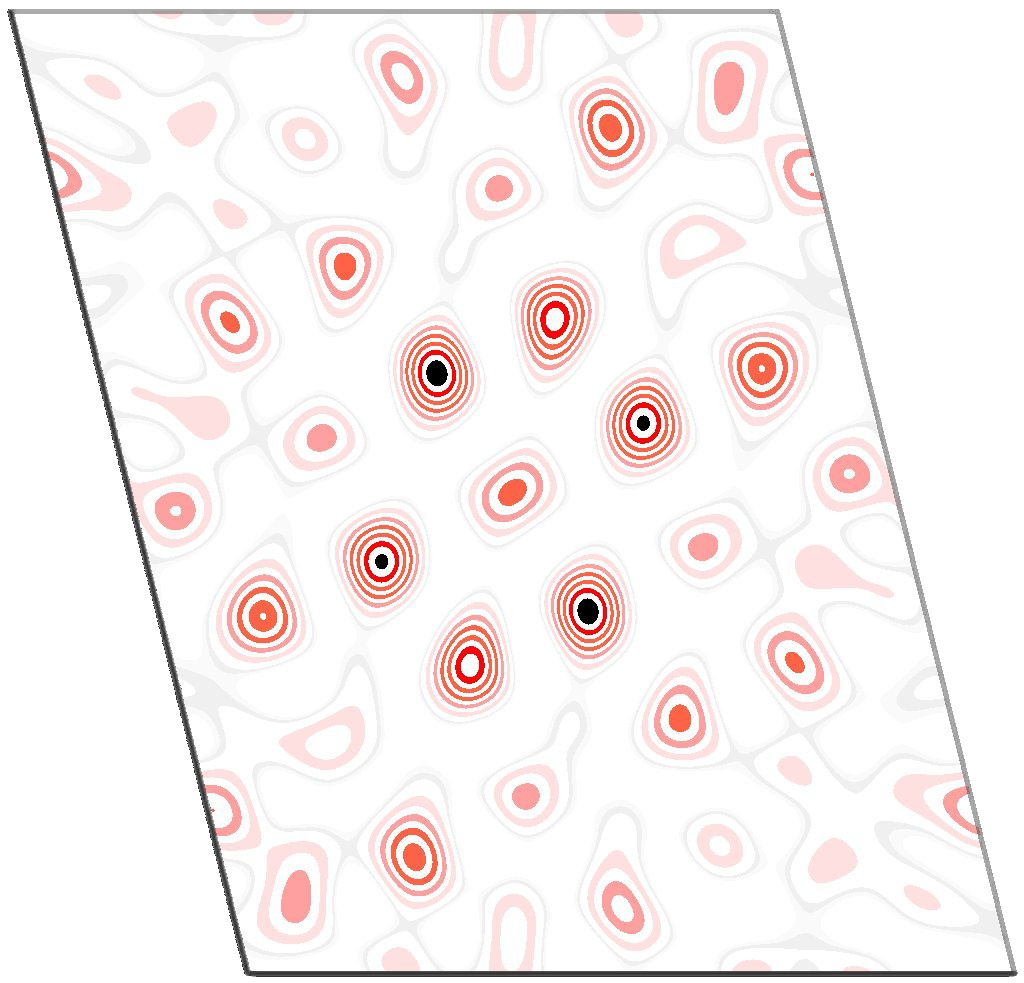

Supplement: Supplementary file 7 [file e-82-00534-sup8.zip › oi2035_SupportingMaterial/Example3/Example3 FOU Maps/Frame_S3 A=0 C=0 D=180 S=0 EFOU Fourier-Map_N48_32bit_gray_1024pix_LUT3.jpg]

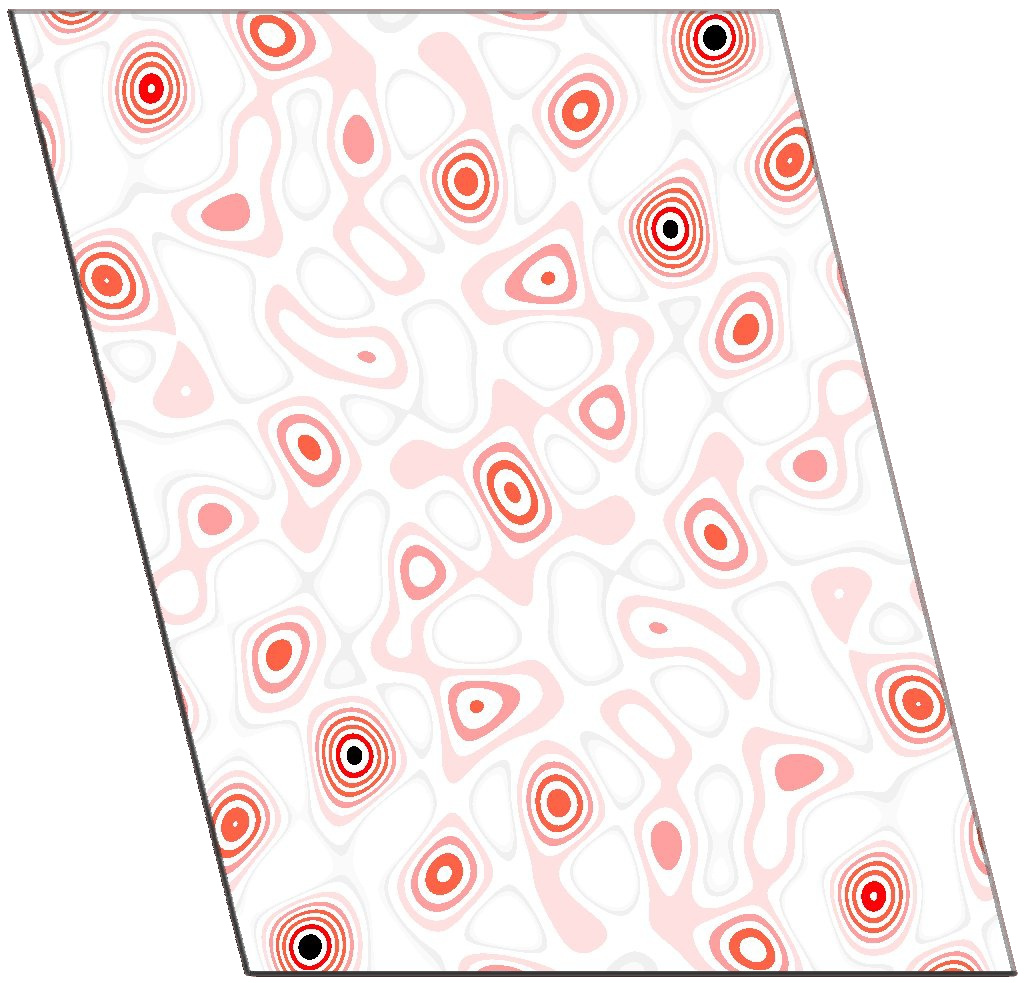

Supplement: Supplementary file 7 [file e-82-00534-sup8.zip › oi2035_SupportingMaterial/Example3/Example3 FOU Maps/Frame_S10 A=180 C=0 D=0 S=180 EFOU Fourier-Map_N48_32bit_gray_1024pix_LUT3.jpg]

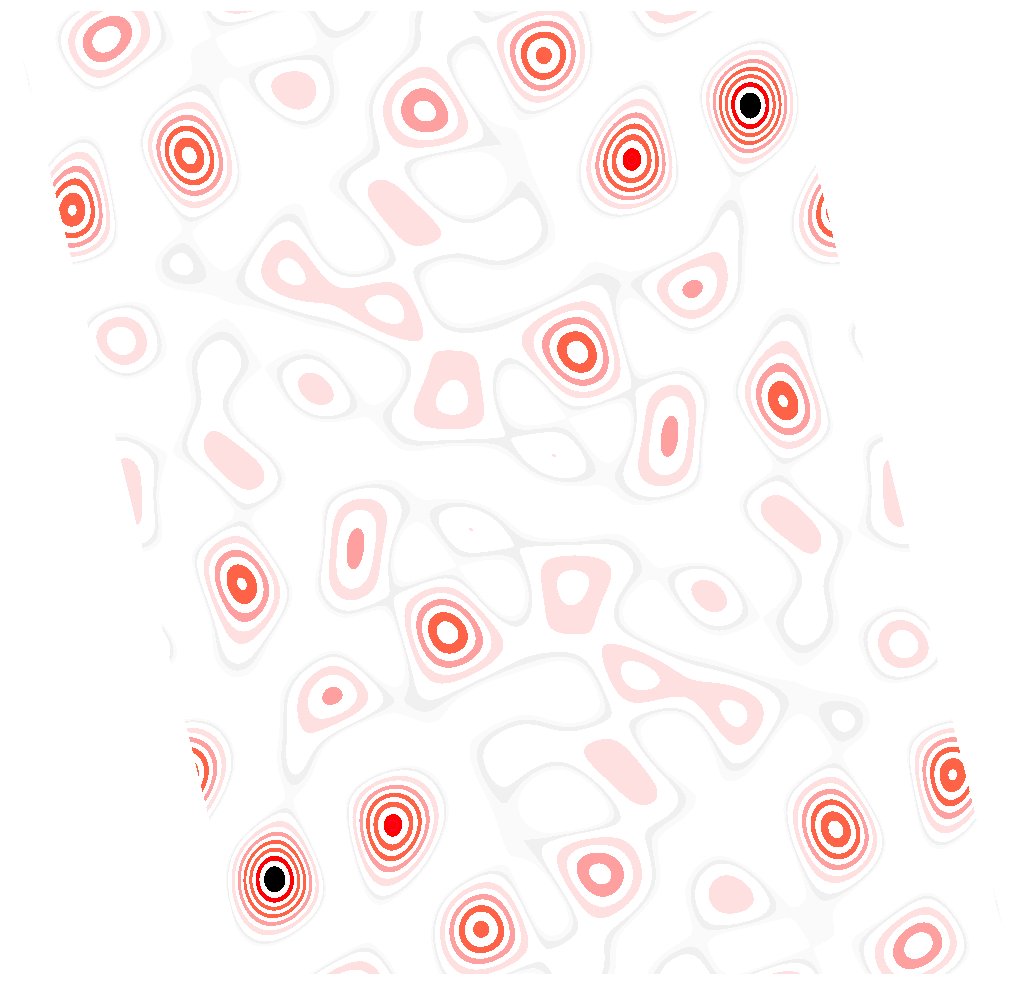

Supplement: Supplementary file 7 [file e-82-00534-sup8.zip › oi2035_SupportingMaterial/Example3/Example3 FOU Maps/S11 A=0 C=180 D=180 S=0 EFOU Fourier-Map_N48_32bit_gray_1024pix_LUT3.jpg]

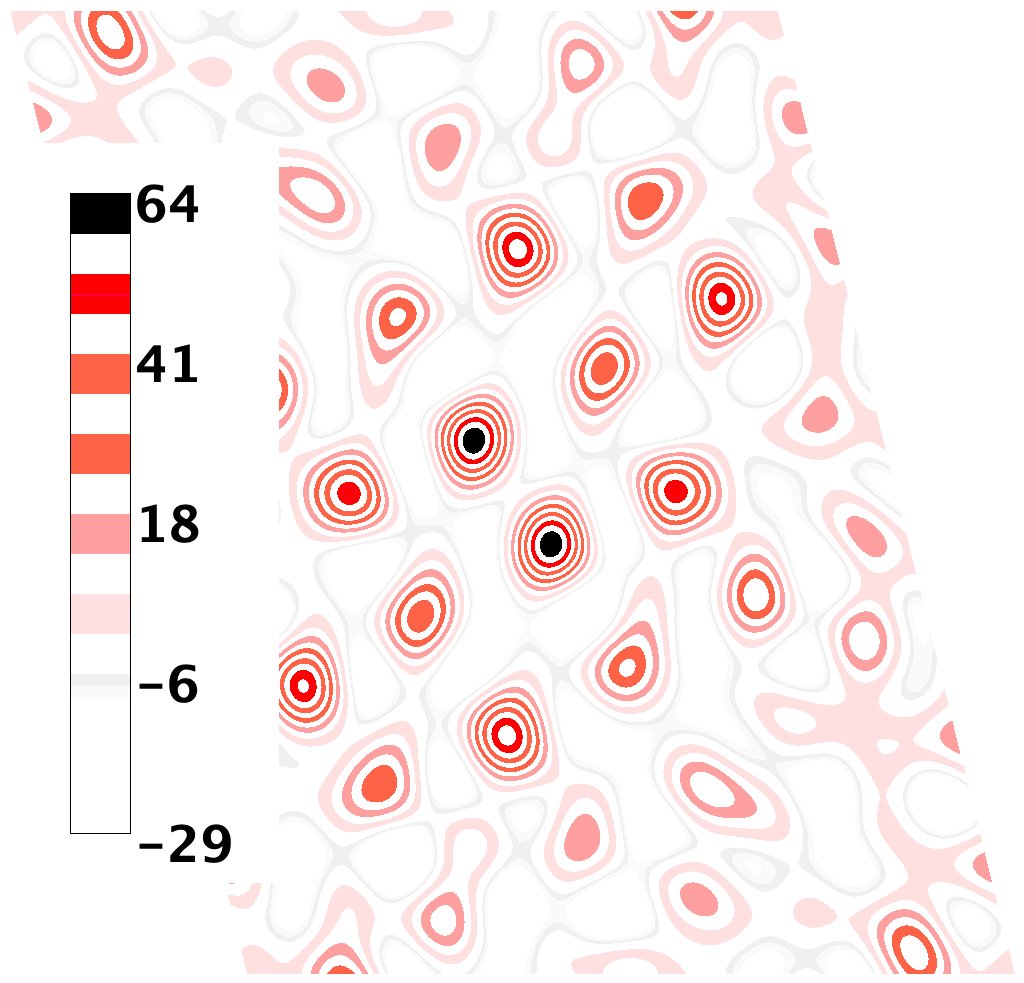

Supplement: Supplementary file 7 [file e-82-00534-sup8.zip › oi2035_SupportingMaterial/Example3/Example3 FOU Maps/S14 A=180 C=180 D=0 S=180 EFOU Fourier-Map_N48_32bit_gray_1024pix_ramp.jpg]

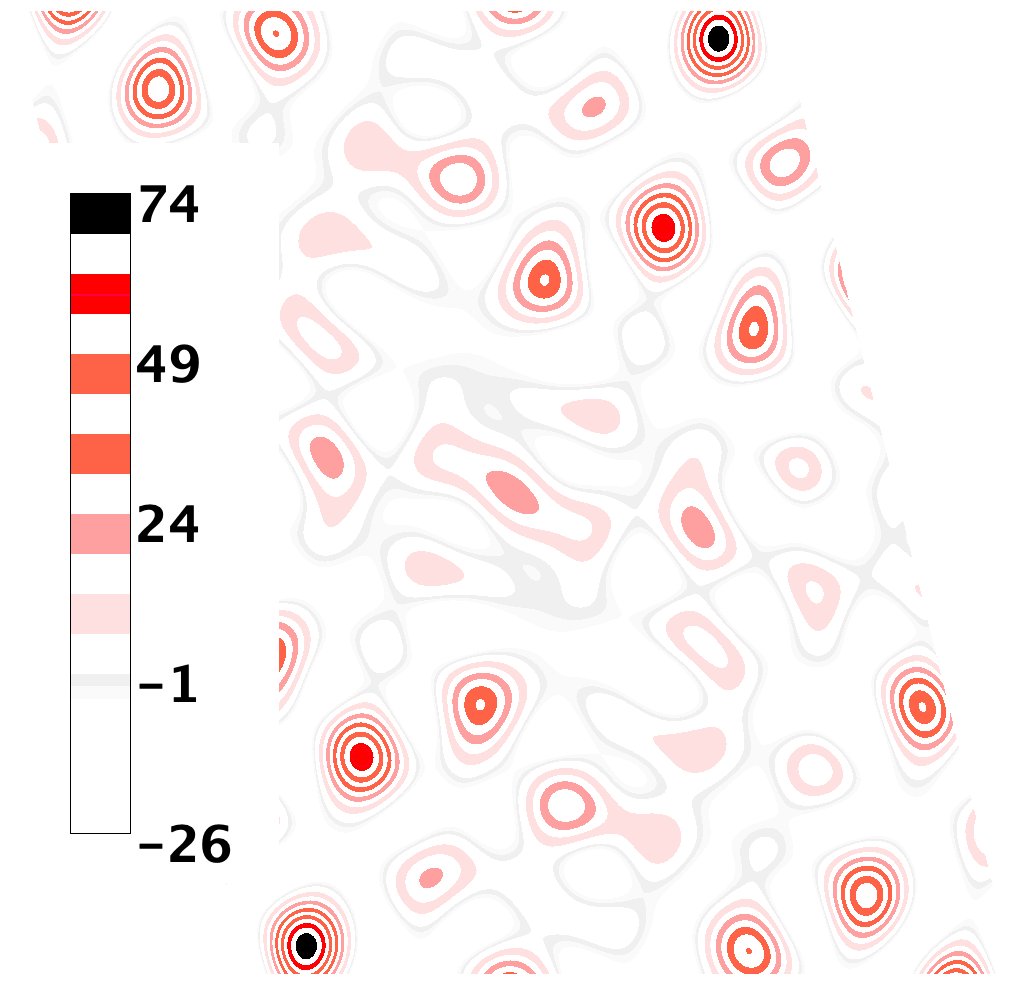

Supplement: Supplementary file 7 [file e-82-00534-sup8.zip › oi2035_SupportingMaterial/Example3/Example3 FOU Maps/S5 A=180 C=0 D=0 S=0 EFOU Fourier-Map_N48_32bit_gray_1024pix_ramp.jpg]

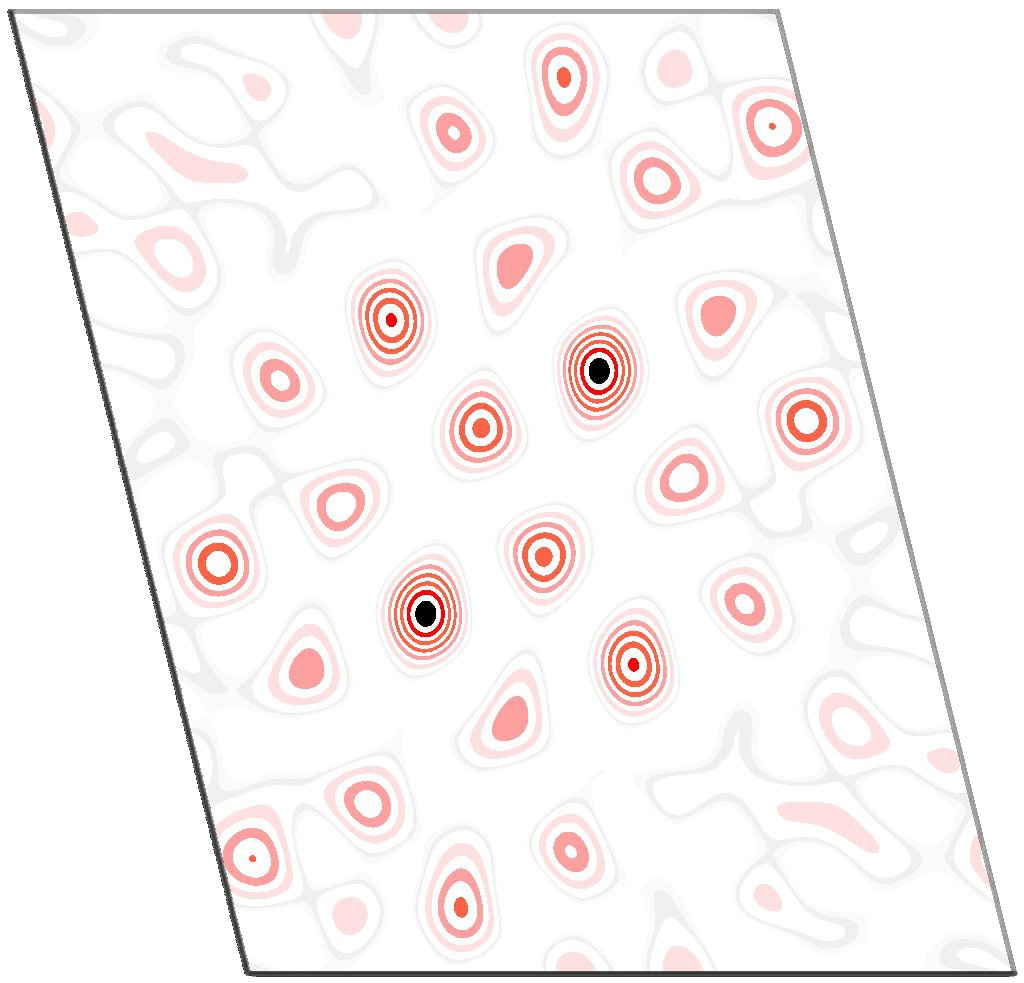

Supplement: Supplementary file 7 [file e-82-00534-sup8.zip › oi2035_SupportingMaterial/Example3/Example3 FOU Maps/Frame_S16 A=180 C=180 D=180 S=180 EFOU Fourier-Map_N48_32bit_gray_1024pix_LUT3.jpg]

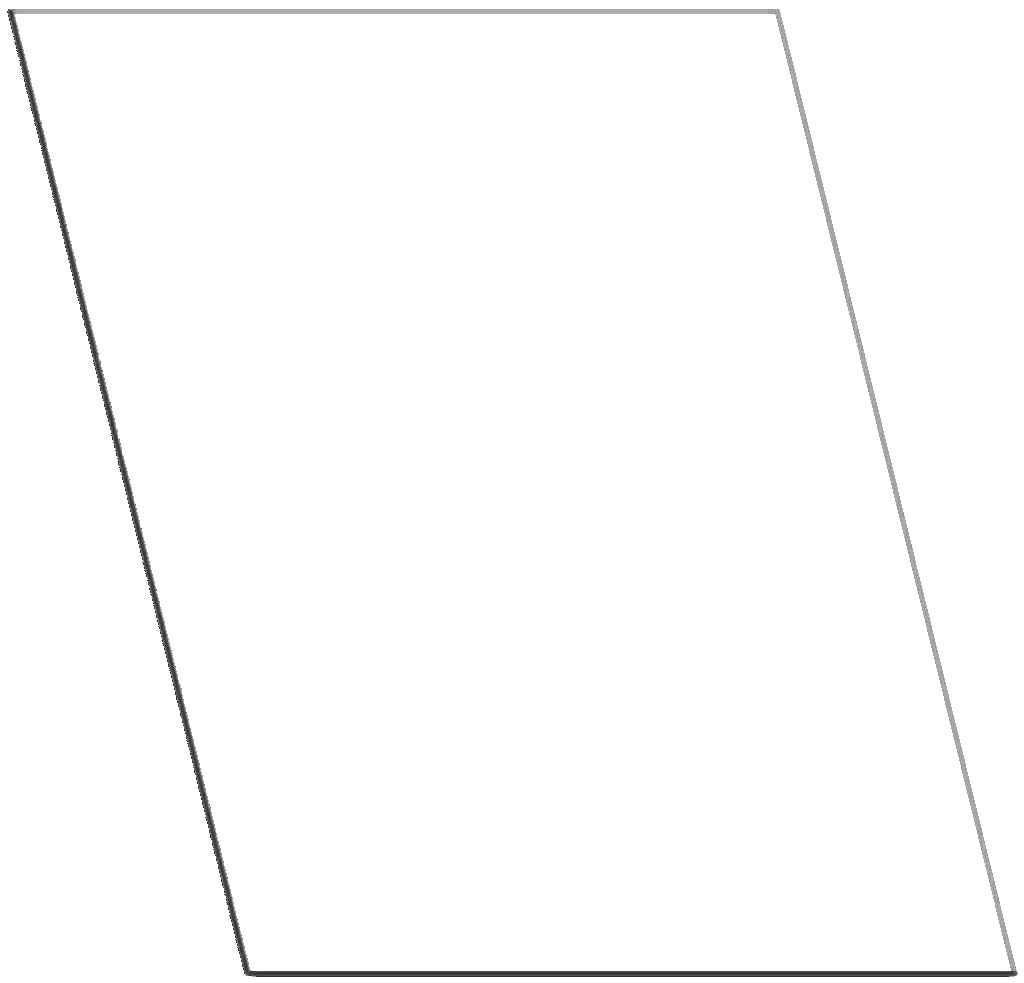

Supplement: Supplementary file 7 [file e-82-00534-sup8.zip › oi2035_SupportingMaterial/Example3/Example3 FOU Maps/unitcell dark FOU Fourier-Map_N1_RGB_1024pix.png]

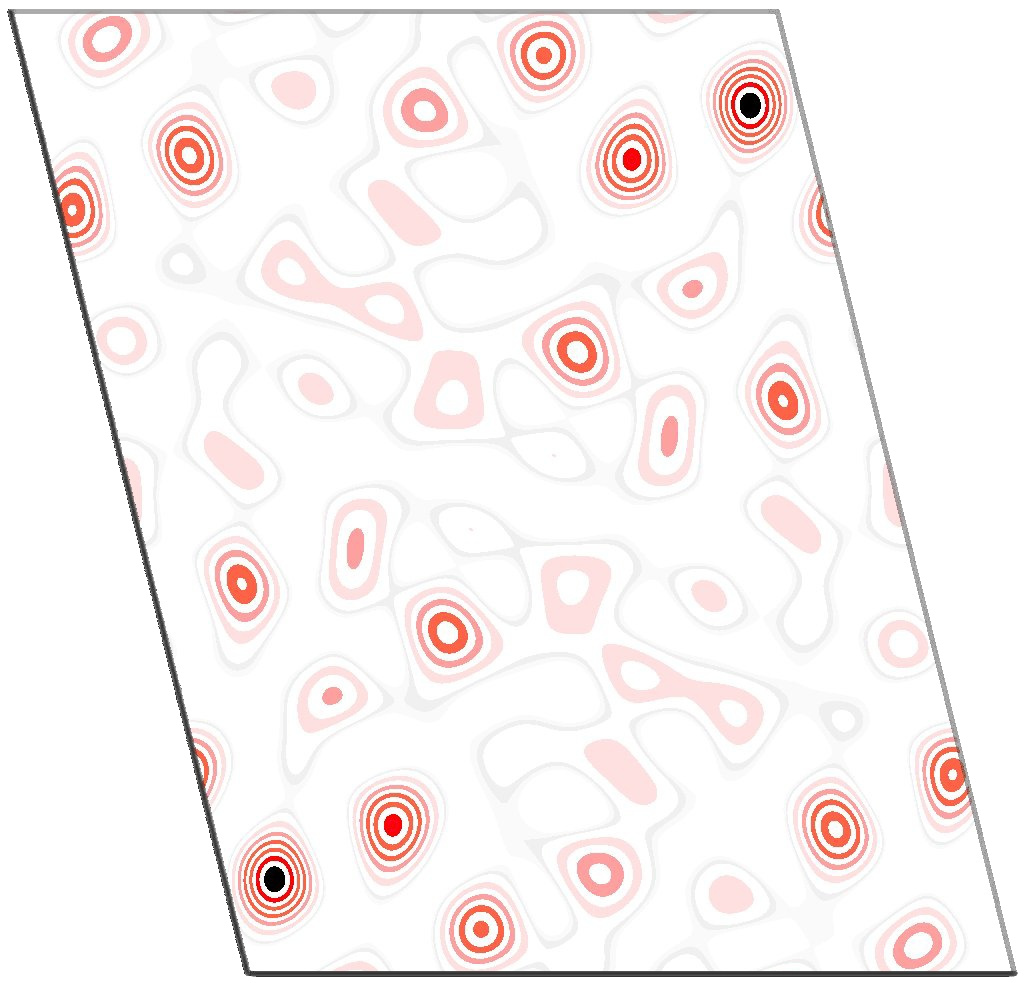

Supplement: Supplementary file 7 [file e-82-00534-sup8.zip › oi2035_SupportingMaterial/Example3/Example3 FOU Maps/Frame_S11 A=0 C=180 D=180 S=0 EFOU Fourier-Map_N48_32bit_gray_1024pix_LUT3.jpg]

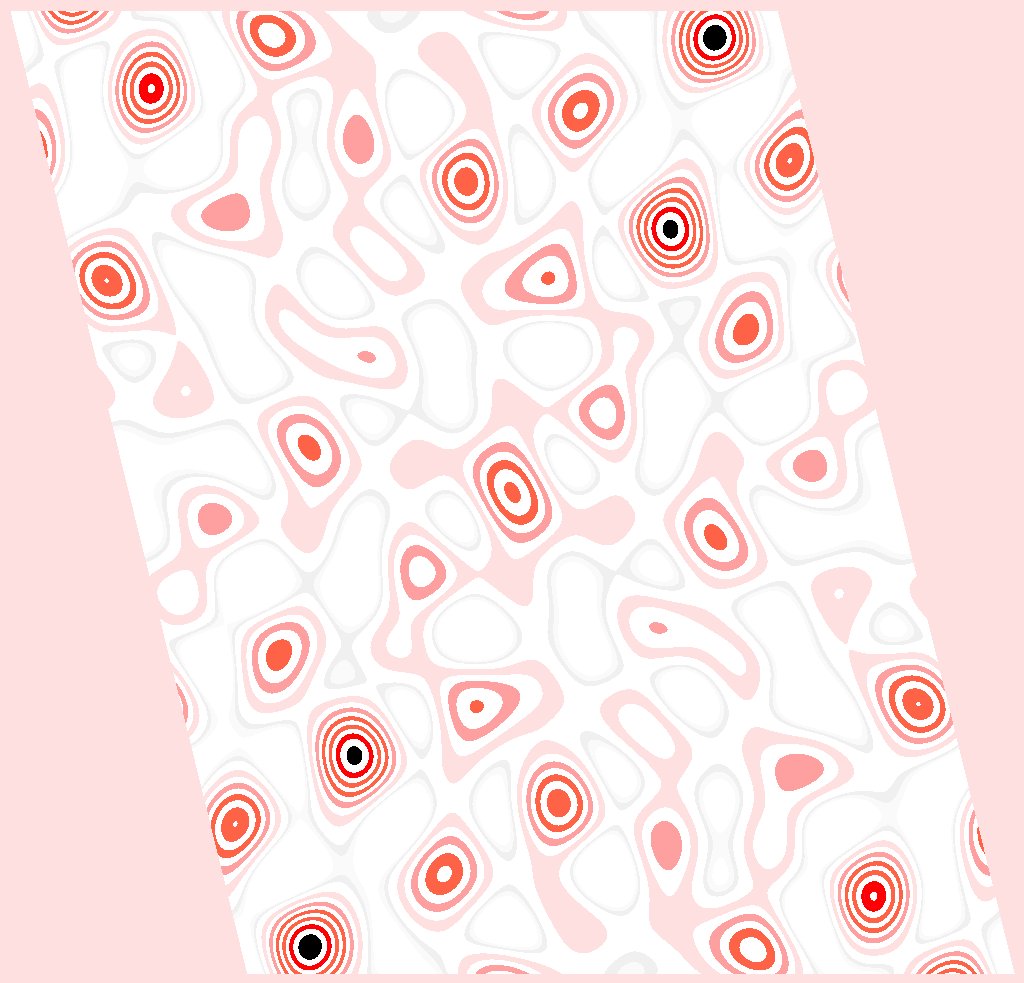

Supplement: Supplementary file 7 [file e-82-00534-sup8.zip › oi2035_SupportingMaterial/Example3/Example3 FOU Maps/S10 A=180 C=0 D=0 S=180 EFOU Fourier-Map_N48_32bit_gray_1024pix_LUT3.jpg]

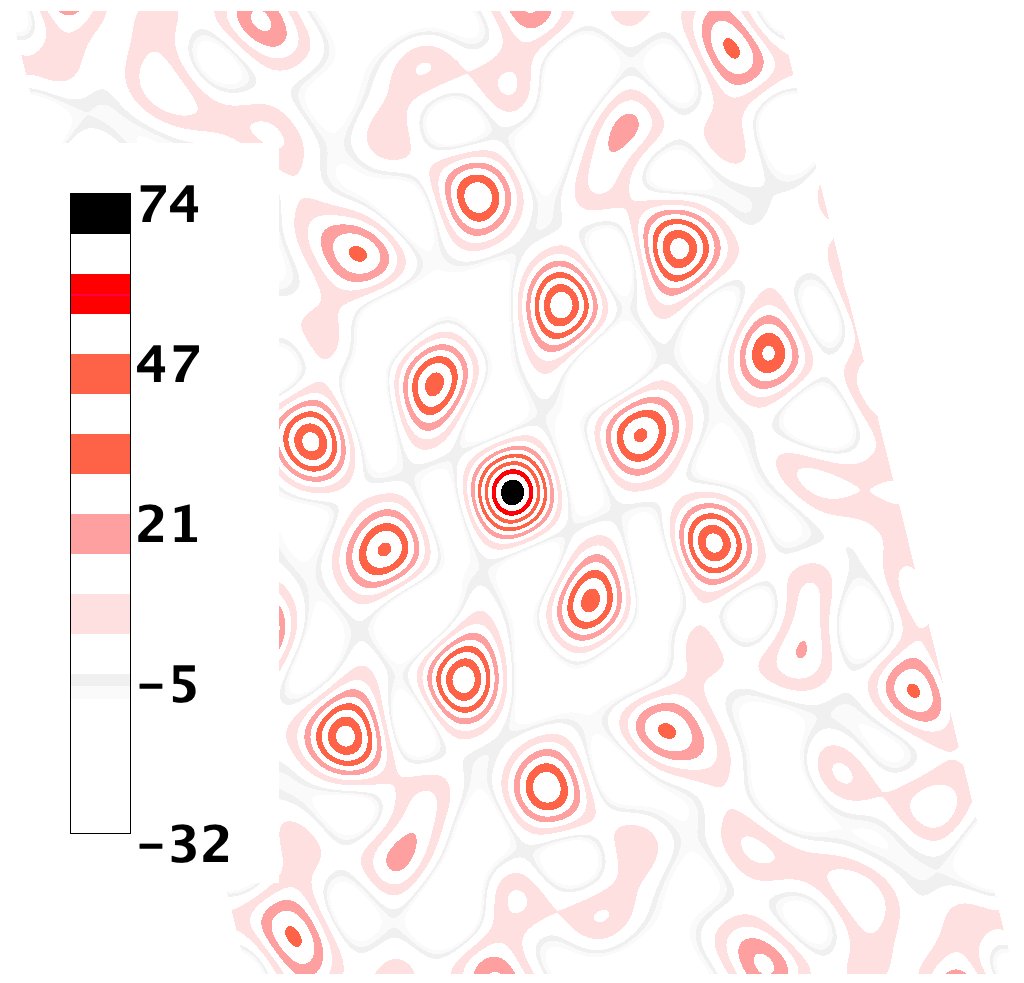

Supplement: Supplementary file 7 [file e-82-00534-sup8.zip › oi2035_SupportingMaterial/Example3/Example3 FOU Maps/S1 A=0 C=0 D=0 S=0 EFOU Fourier-Map_N48_32bit_gray_1024pix_ramp.jpg]

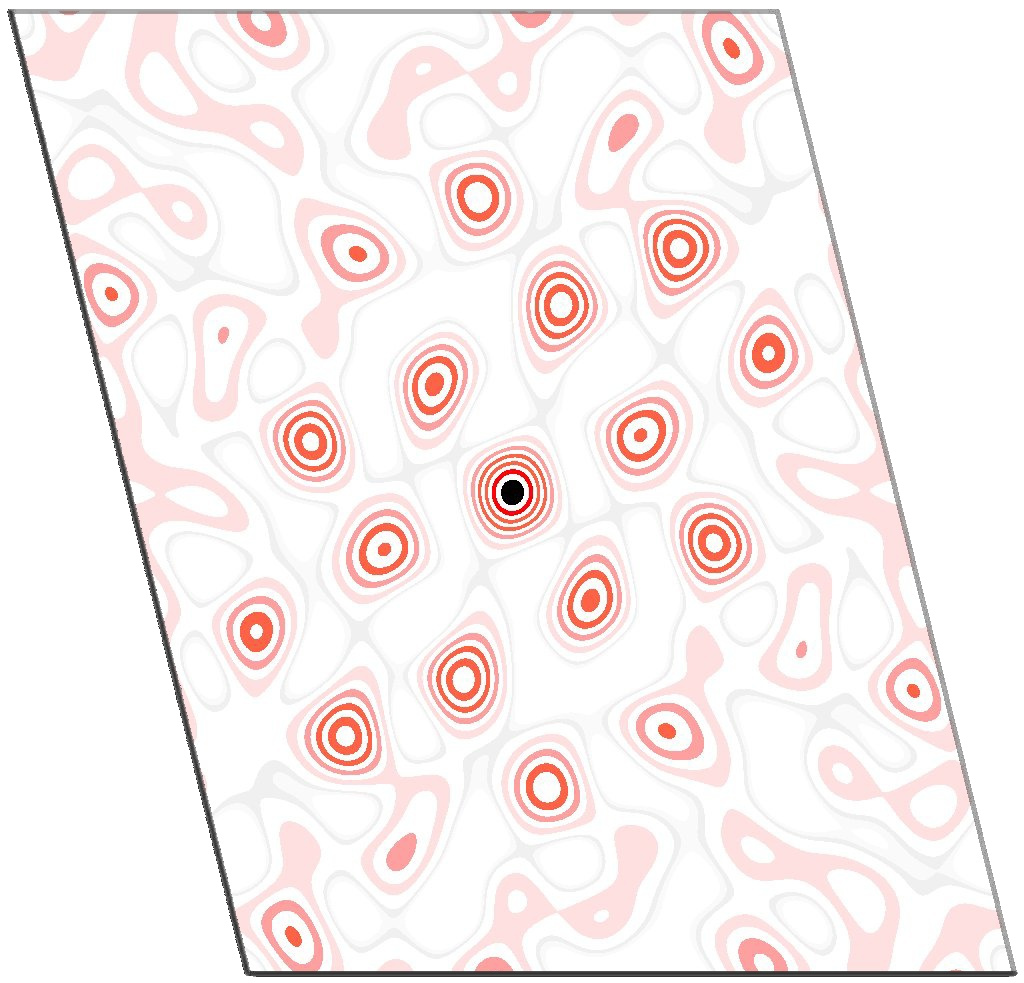

Supplement: Supplementary file 7 [file e-82-00534-sup8.zip › oi2035_SupportingMaterial/Example3/Example3 FOU Maps/Frame_S1 A=0 C=0 D=0 S=0 EFOU Fourier-Map_N48_32bit_gray_1024pix_LUT3.jpg]

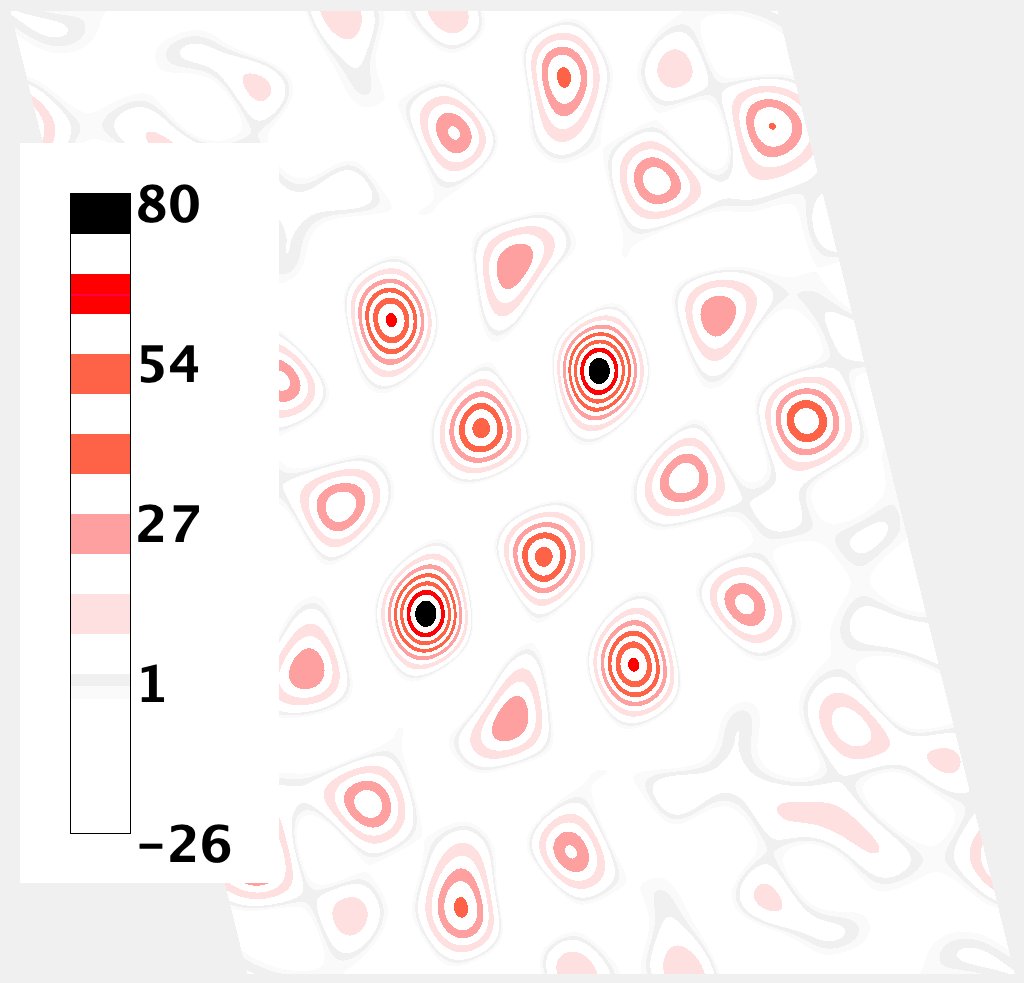

Supplement: Supplementary file 7 [file e-82-00534-sup8.zip › oi2035_SupportingMaterial/Example3/Example3 FOU Maps/S16 A=180 C=180 D=180 S=180 EFOU Fourier-Map_N48_32bit_gray_1024pix_ramp.jpg]

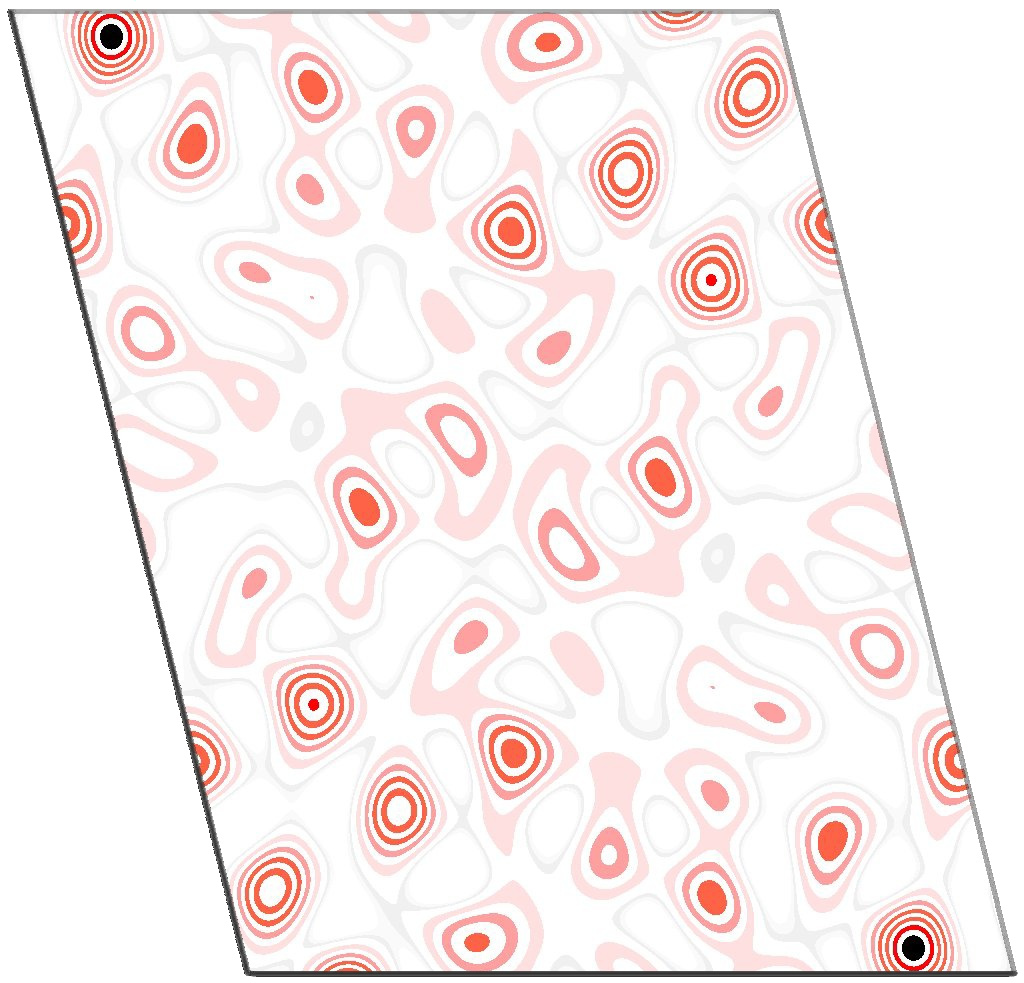

Supplement: Supplementary file 7 [file e-82-00534-sup8.zip › oi2035_SupportingMaterial/Example3/Example3 FOU Maps/Frame_S4 A=0 C=180 D=0 S=0 EFOU Fourier-Map_N48_32bit_gray_1024pix_LUT3.jpg]

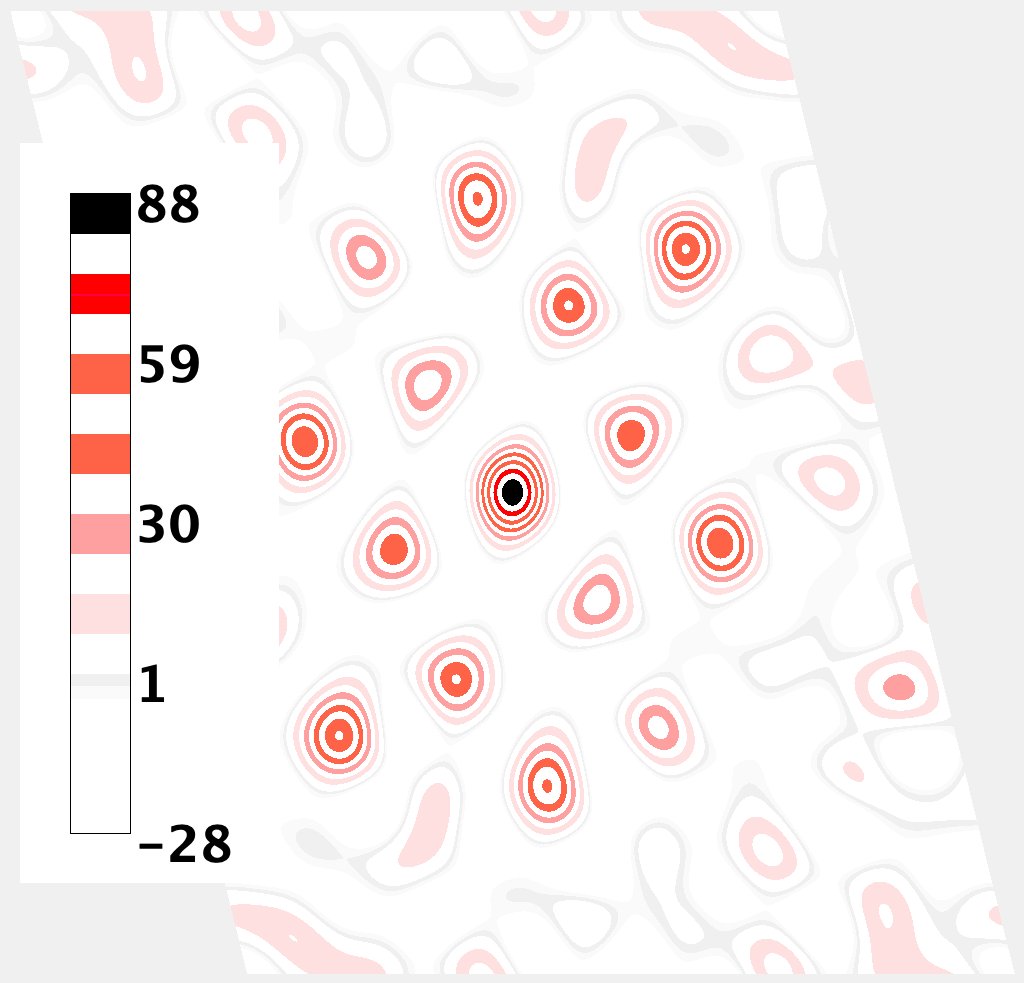

Supplement: Supplementary file 7 [file e-82-00534-sup8.zip › oi2035_SupportingMaterial/Example3/Example3 FOU Maps/S2 A=0 C=0 D=0 S=180 EFOU Fourier-Map_N48_32bit_gray_1024pix_ramp.jpg]

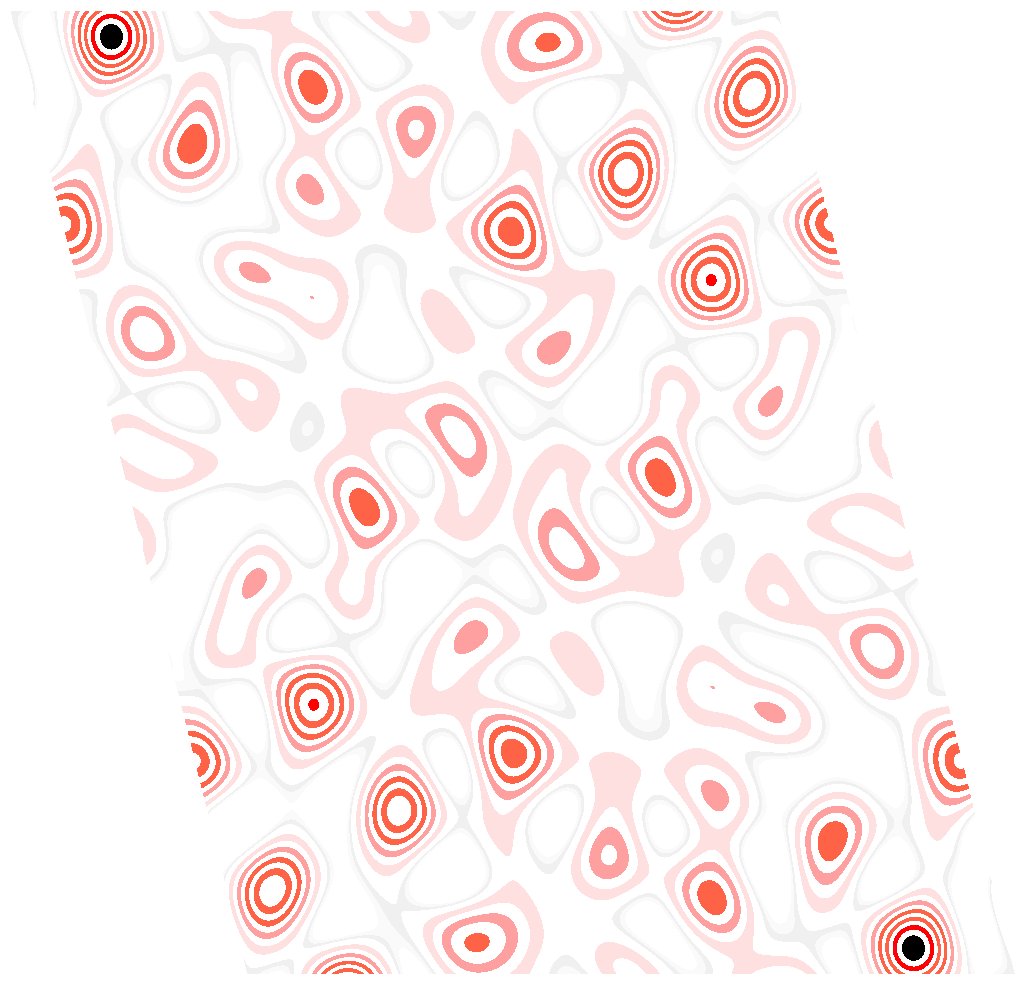

Supplement: Supplementary file 7 [file e-82-00534-sup8.zip › oi2035_SupportingMaterial/Example3/Example3 FOU Maps/S4 A=0 C=180 D=0 S=0 EFOU Fourier-Map_N48_32bit_gray_1024pix_LUT3.jpg]

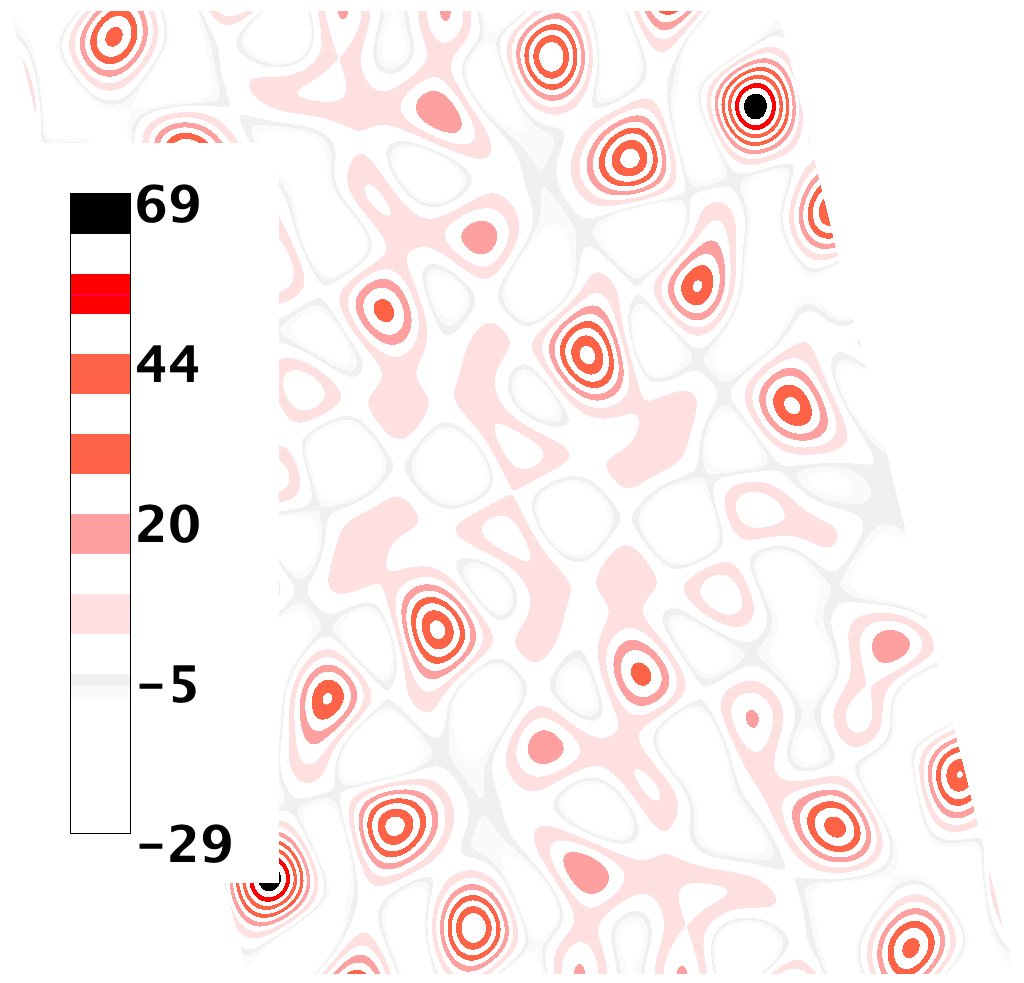

Supplement: Supplementary file 7 [file e-82-00534-sup8.zip › oi2035_SupportingMaterial/Example3/Example3 FOU Maps/S12 A=0 C=180 D=180 S=180 EFOU Fourier-Map_N48_32bit_gray_1024pix_ramp.jpg]

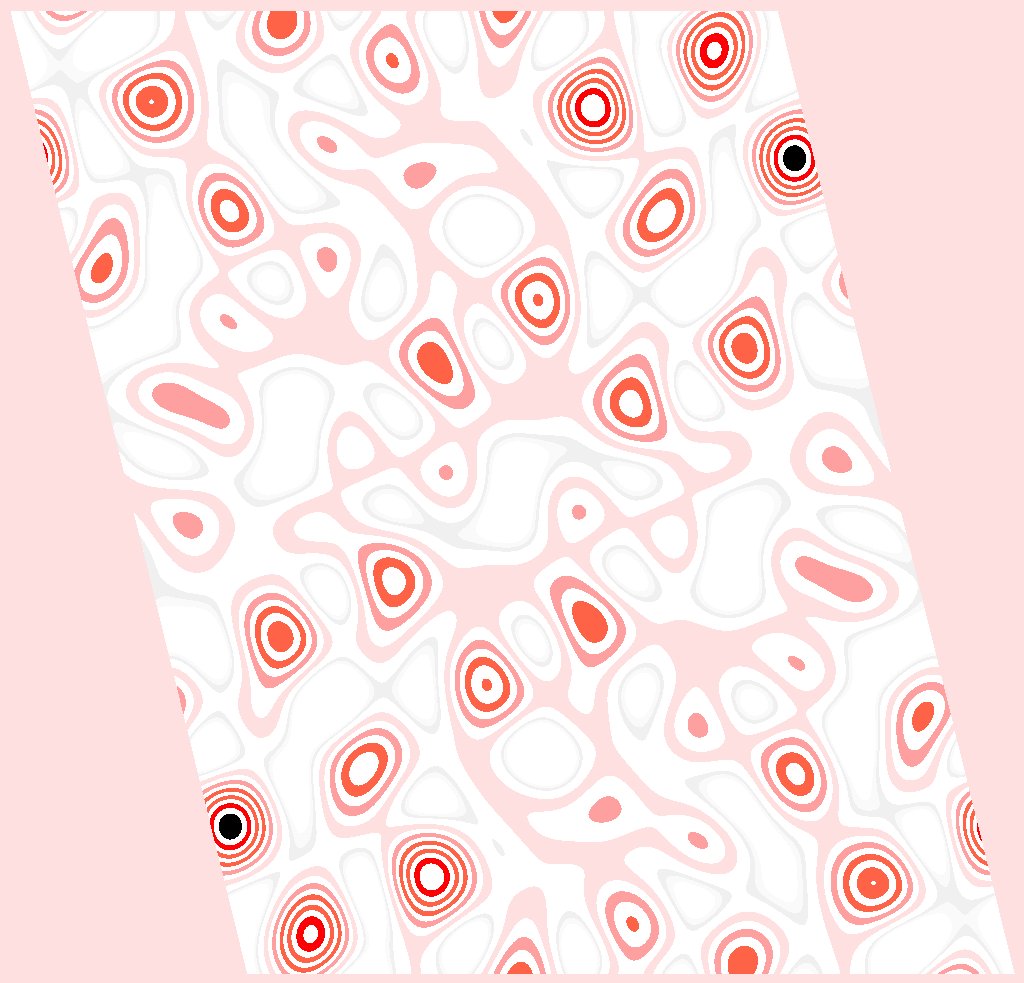

Supplement: Supplementary file 7 [file e-82-00534-sup8.zip › oi2035_SupportingMaterial/Example3/Example3 FOU Maps/S8 A=180 C=0 D=180 S=0 EFOU Fourier-Map_N48_32bit_gray_1024pix_LUT3.jpg]

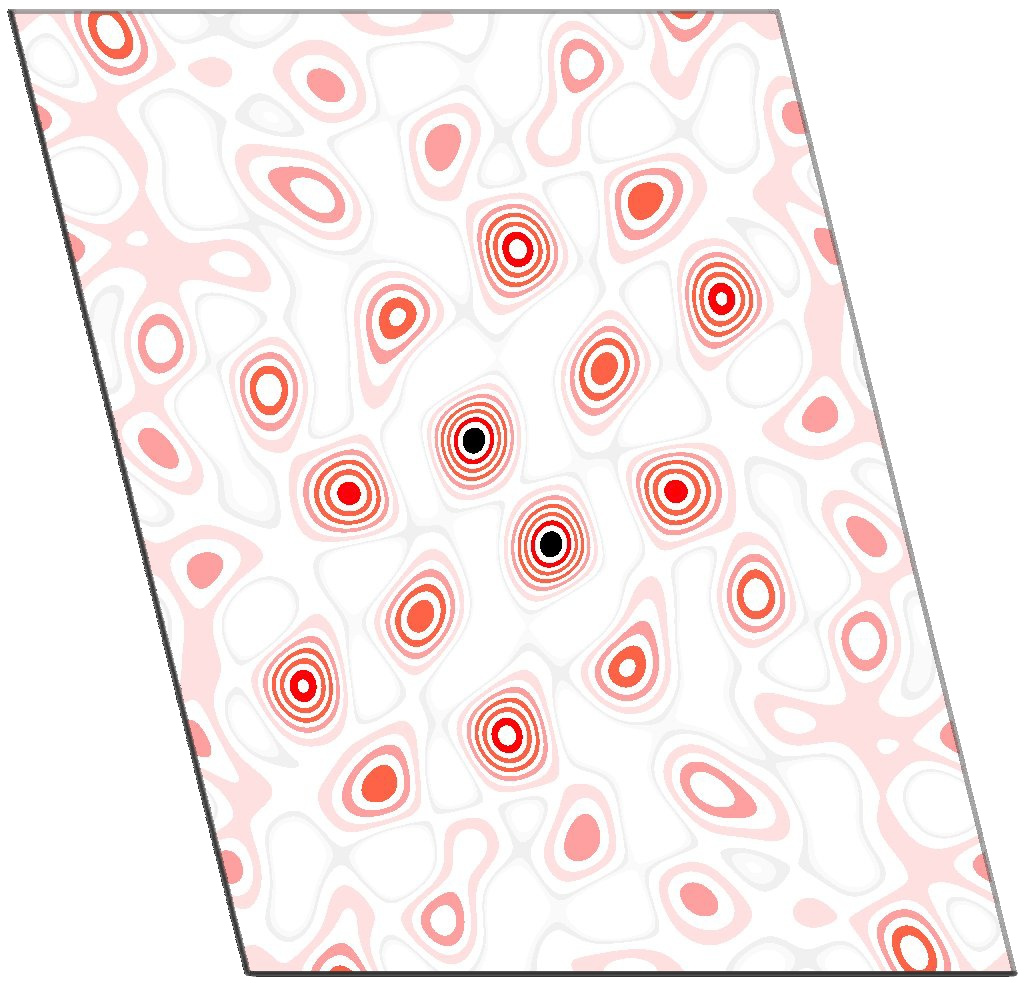

Supplement: Supplementary file 7 [file e-82-00534-sup8.zip › oi2035_SupportingMaterial/Example3/Example3 FOU Maps/Frame_S14 A=180 C=180 D=0 S=180 EFOU Fourier-Map_N48_32bit_gray_1024pix_LUT3.jpg]

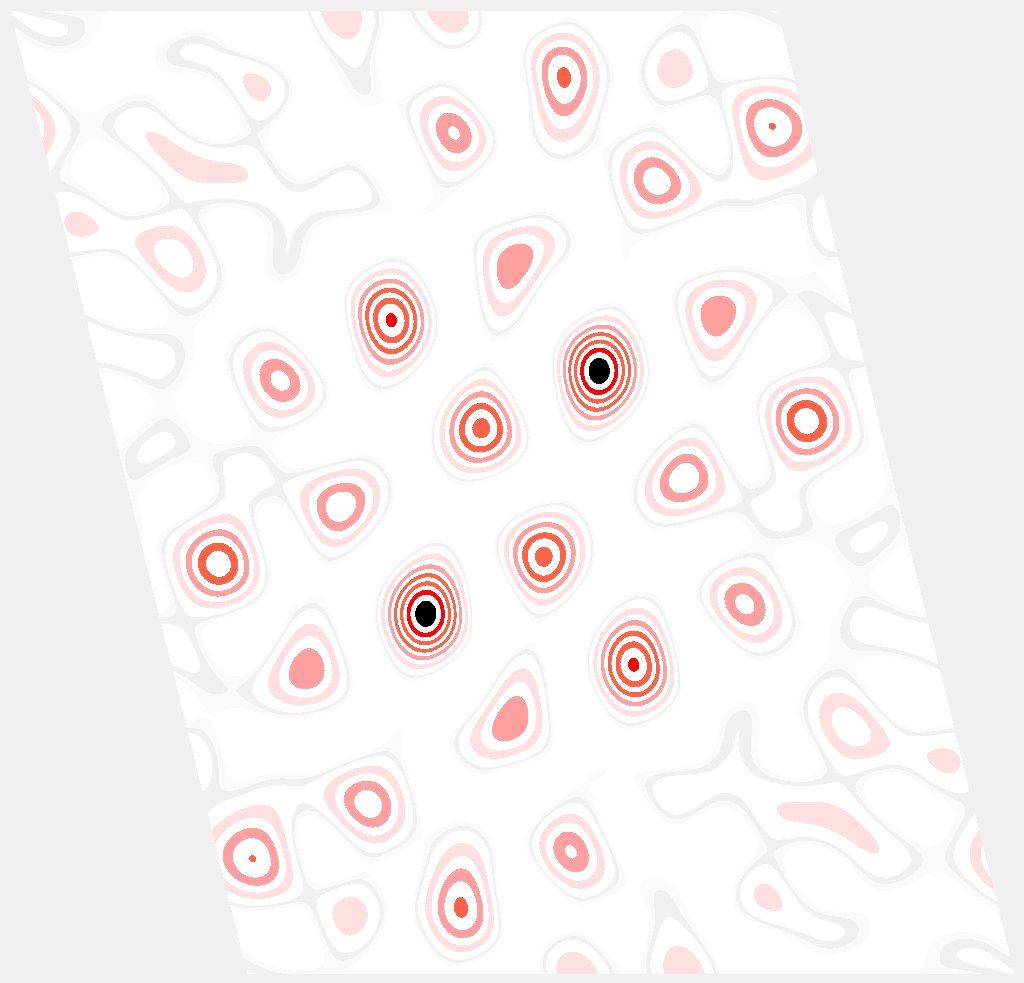

Supplement: Supplementary file 7 [file e-82-00534-sup8.zip › oi2035_SupportingMaterial/Example3/Example3 FOU Maps/S16 A=180 C=180 D=180 S=180 EFOU Fourier-Map_N48_32bit_gray_1024pix_LUT3.jpg]

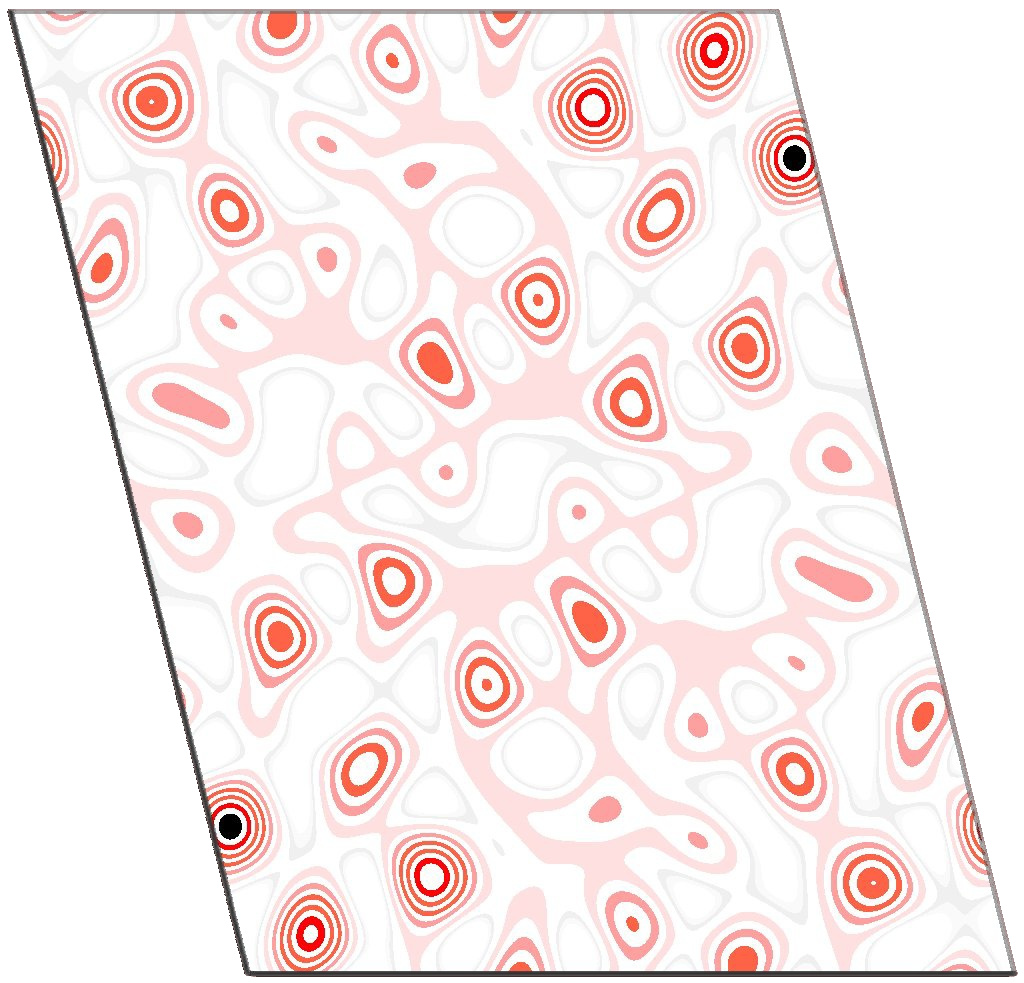

Supplement: Supplementary file 7 [file e-82-00534-sup8.zip › oi2035_SupportingMaterial/Example3/Example3 FOU Maps/Frame_S8 A=180 C=0 D=180 S=0 EFOU Fourier-Map_N48_32bit_gray_1024pix_LUT3.jpg]

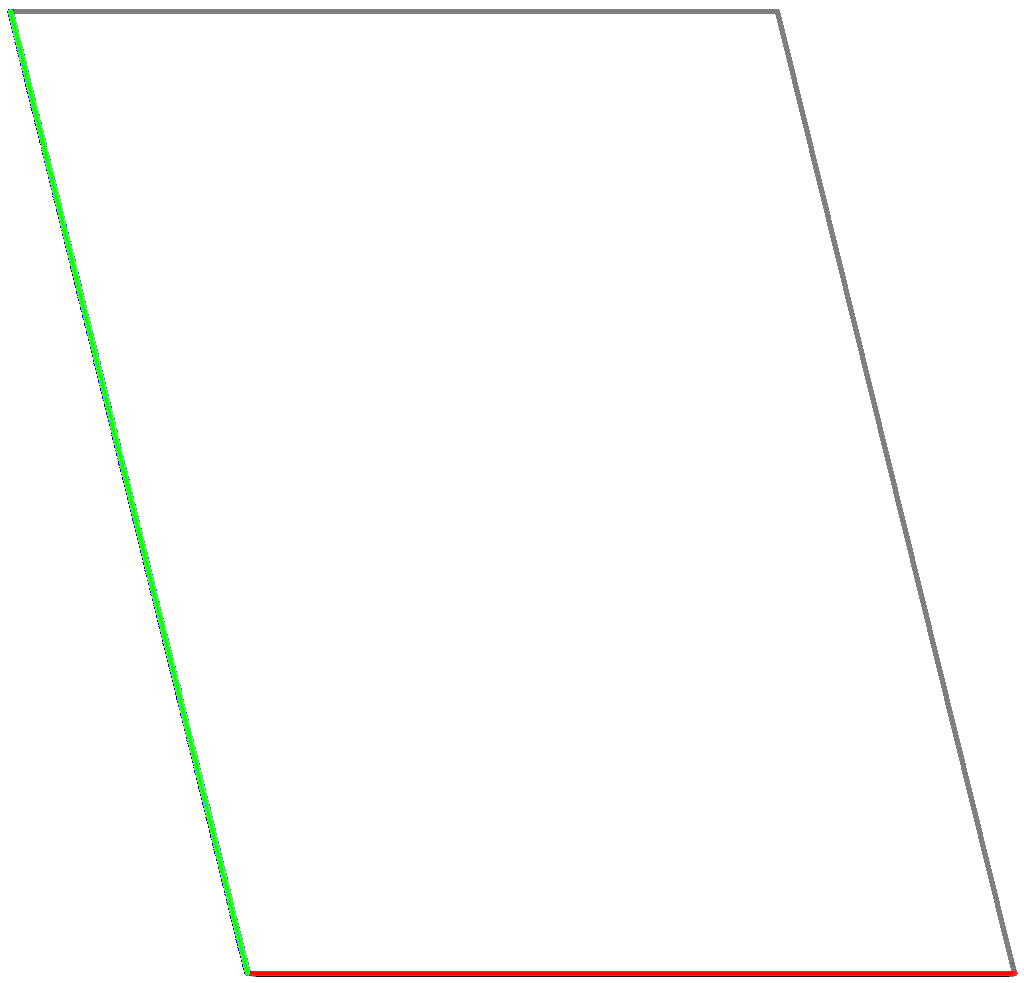

Supplement: Supplementary file 7 [file e-82-00534-sup8.zip › oi2035_SupportingMaterial/Example3/Example3 FOU Maps/unitcell FOU Fourier-Map_N1_RGB_1024pix.png]

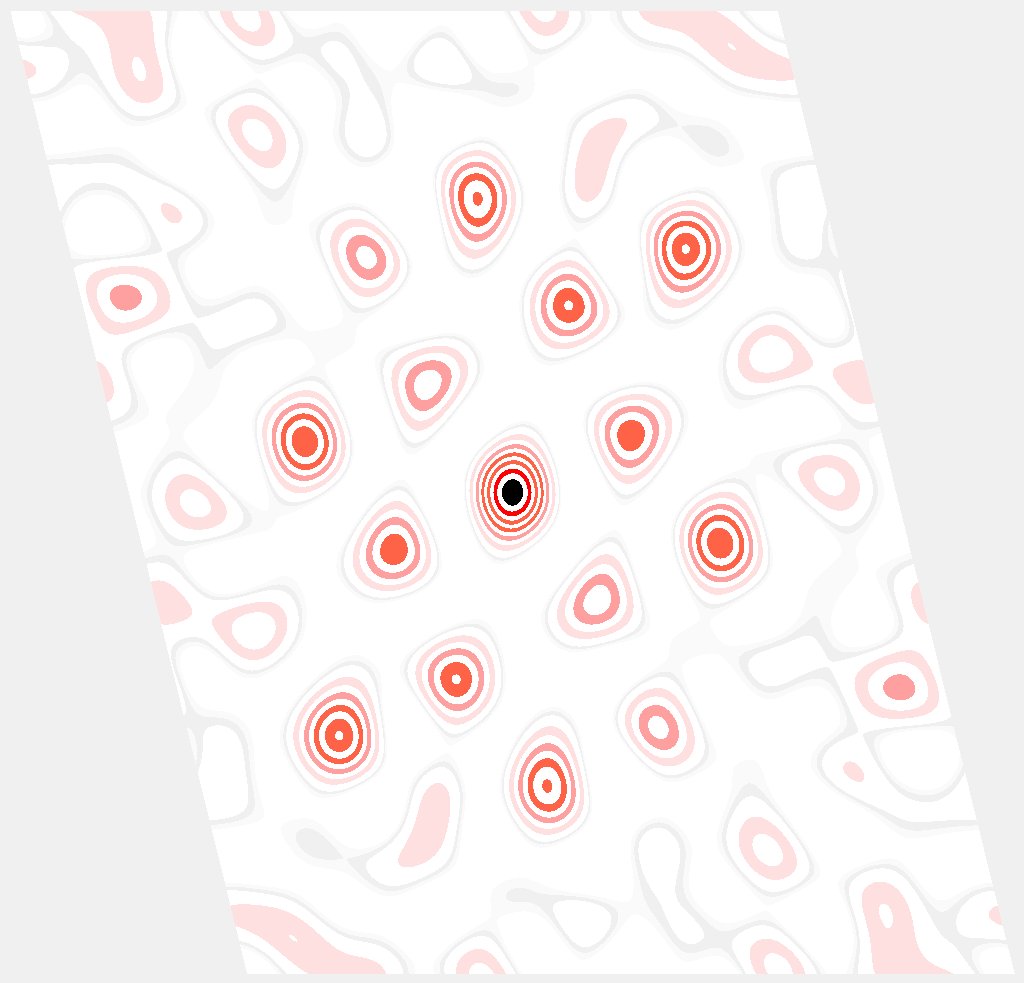

Supplement: Supplementary file 7 [file e-82-00534-sup8.zip › oi2035_SupportingMaterial/Example3/Example3 FOU Maps/S2 A=0 C=0 D=0 S=180 EFOU Fourier-Map_N48_32bit_gray_1024pix_LUT3.jpg]

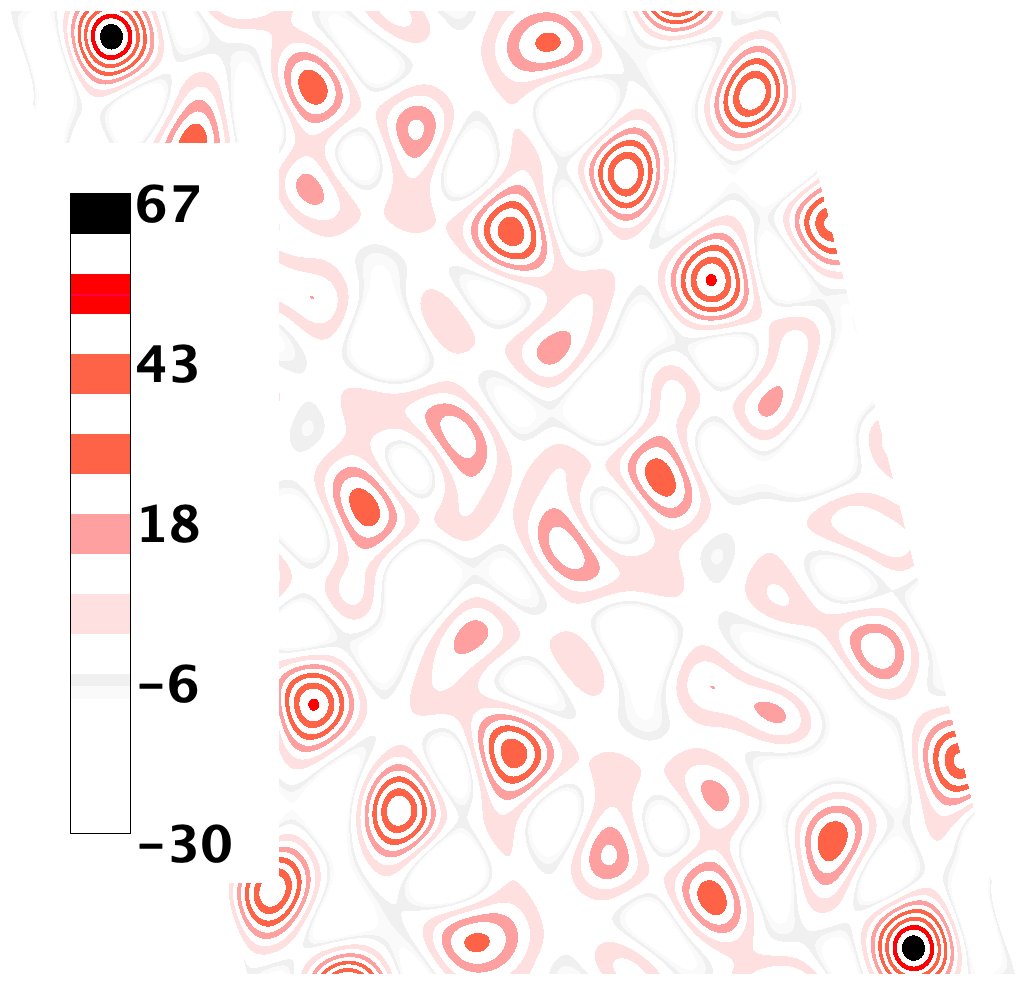

Supplement: Supplementary file 7 [file e-82-00534-sup8.zip › oi2035_SupportingMaterial/Example3/Example3 FOU Maps/S4 A=0 C=180 D=0 S=0 EFOU Fourier-Map_N48_32bit_gray_1024pix_ramp.jpg]

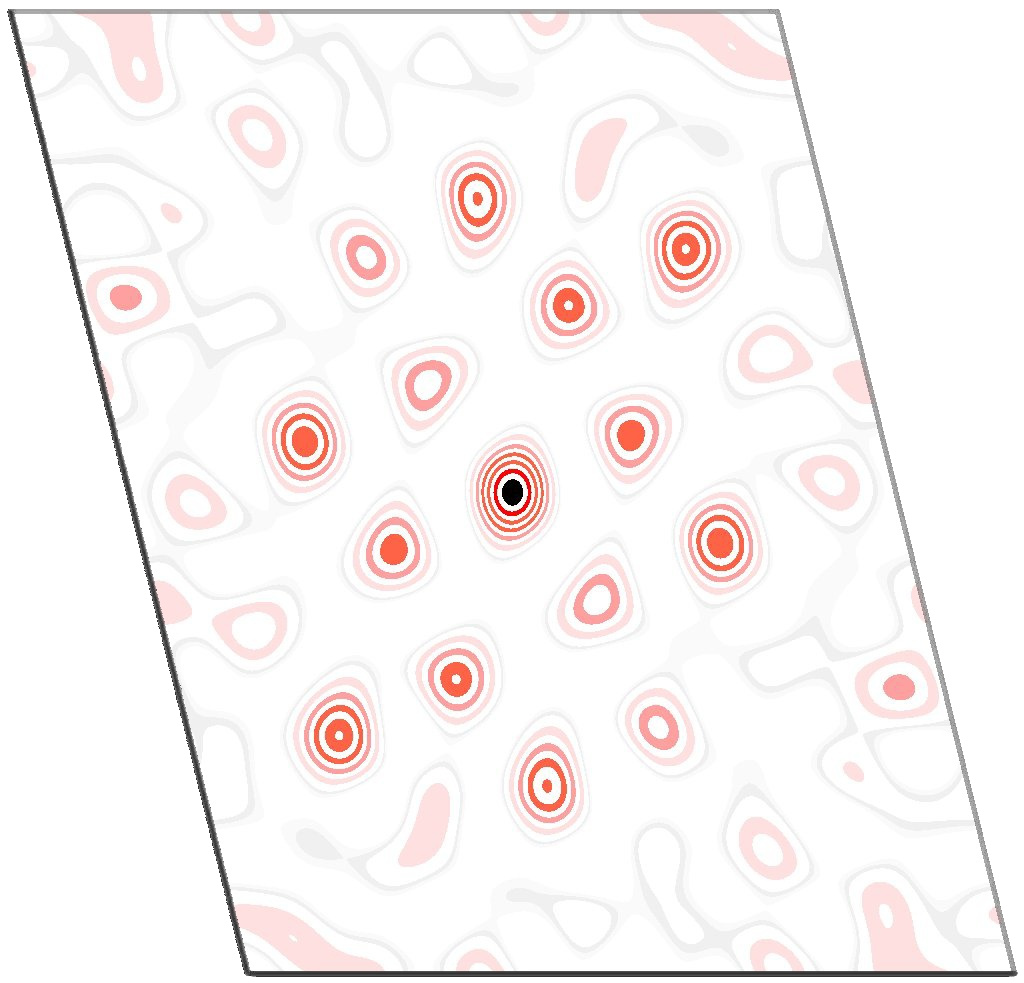

Supplement: Supplementary file 7 [file e-82-00534-sup8.zip › oi2035_SupportingMaterial/Example3/Example3 FOU Maps/Frame_S2 A=0 C=0 D=0 S=180 EFOU Fourier-Map_N48_32bit_gray_1024pix_LUT3.jpg]

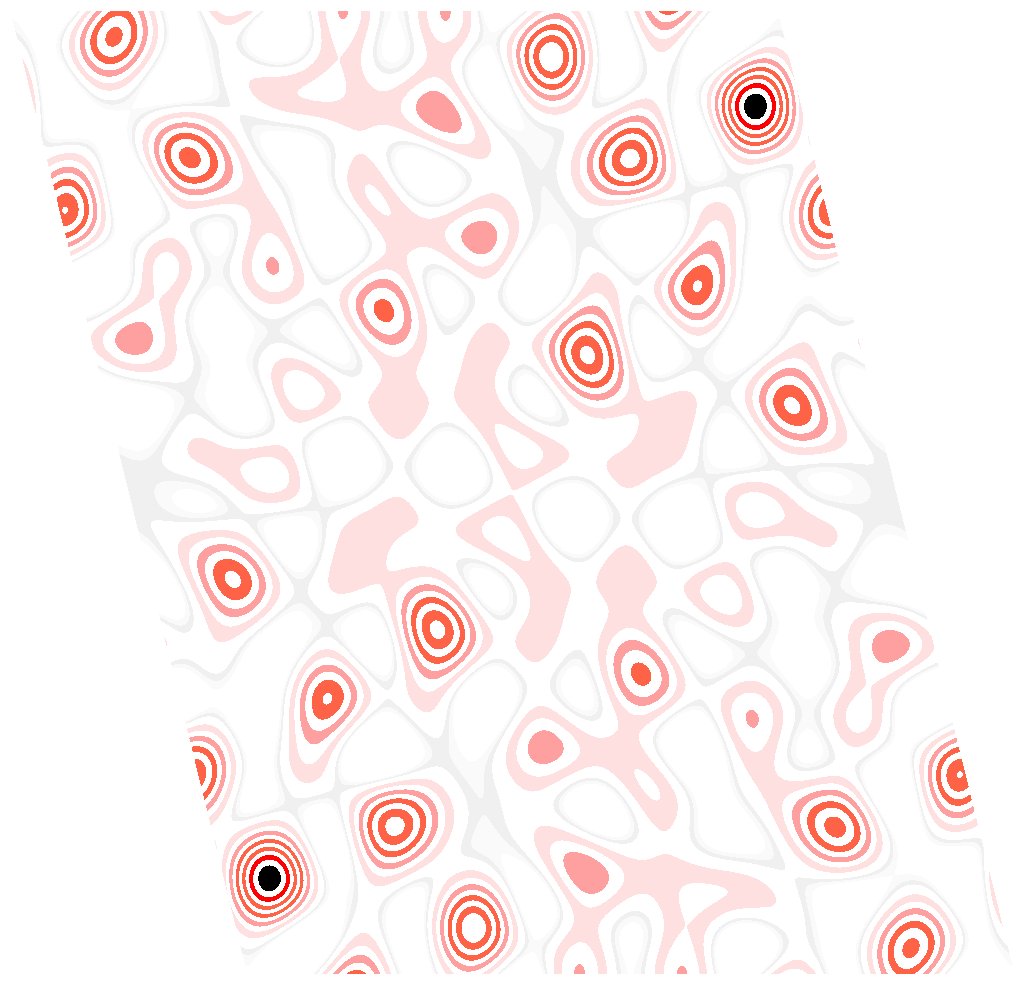

Supplement: Supplementary file 7 [file e-82-00534-sup8.zip › oi2035_SupportingMaterial/Example3/Example3 FOU Maps/S12 A=0 C=180 D=180 S=180 EFOU Fourier-Map_N48_32bit_gray_1024pix_LUT3.jpg]

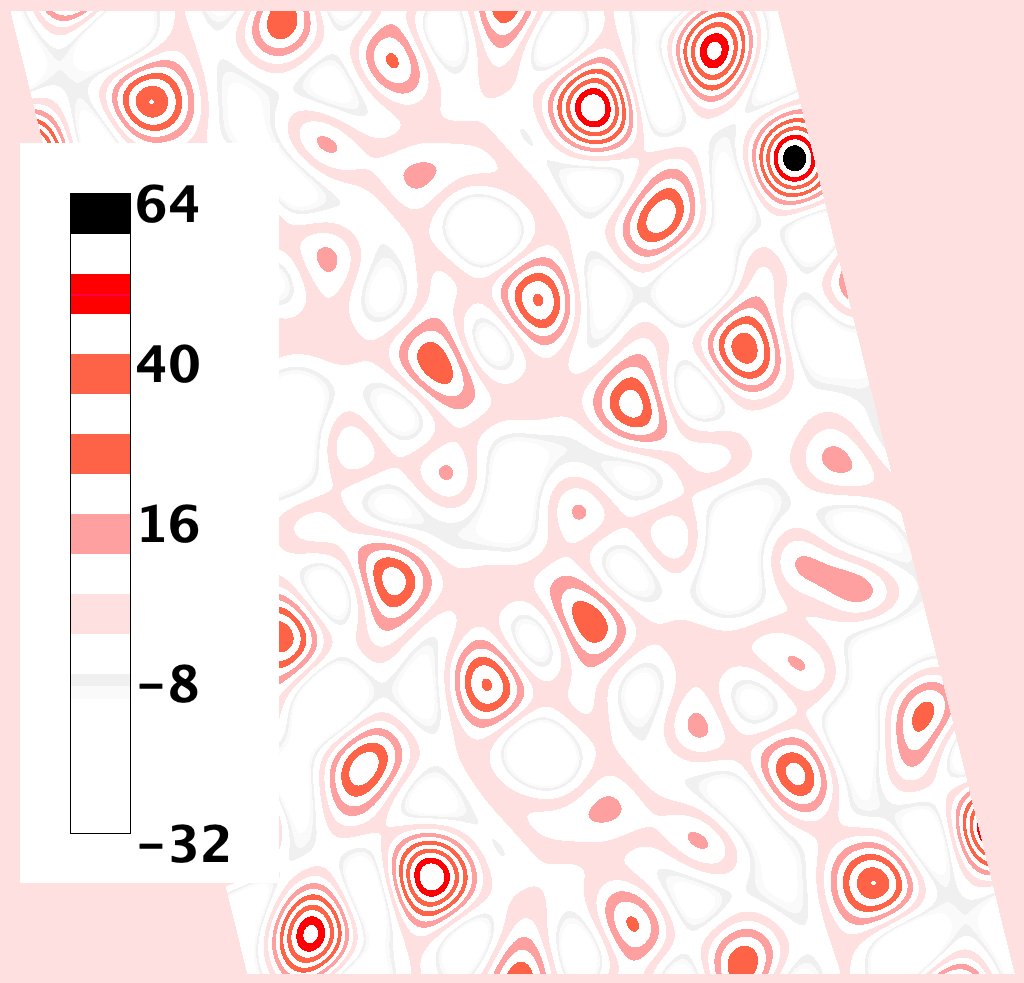

Supplement: Supplementary file 7 [file e-82-00534-sup8.zip › oi2035_SupportingMaterial/Example3/Example3 FOU Maps/S8 A=180 C=0 D=180 S=0 EFOU Fourier-Map_N48_32bit_gray_1024pix_ramp.jpg]

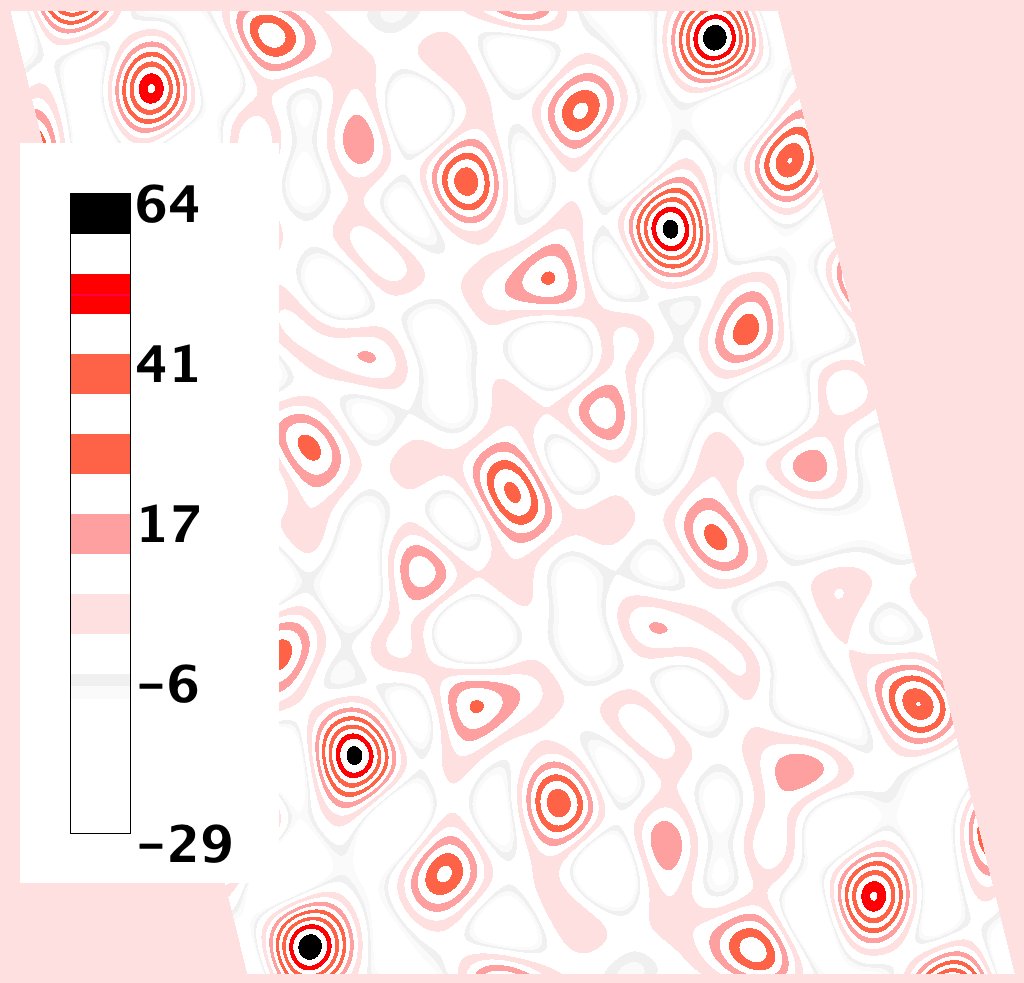

Supplement: Supplementary file 7 [file e-82-00534-sup8.zip › oi2035_SupportingMaterial/Example3/Example3 FOU Maps/S10 A=180 C=0 D=0 S=180 EFOU Fourier-Map_N48_32bit_gray_1024pix_ramp.jpg]

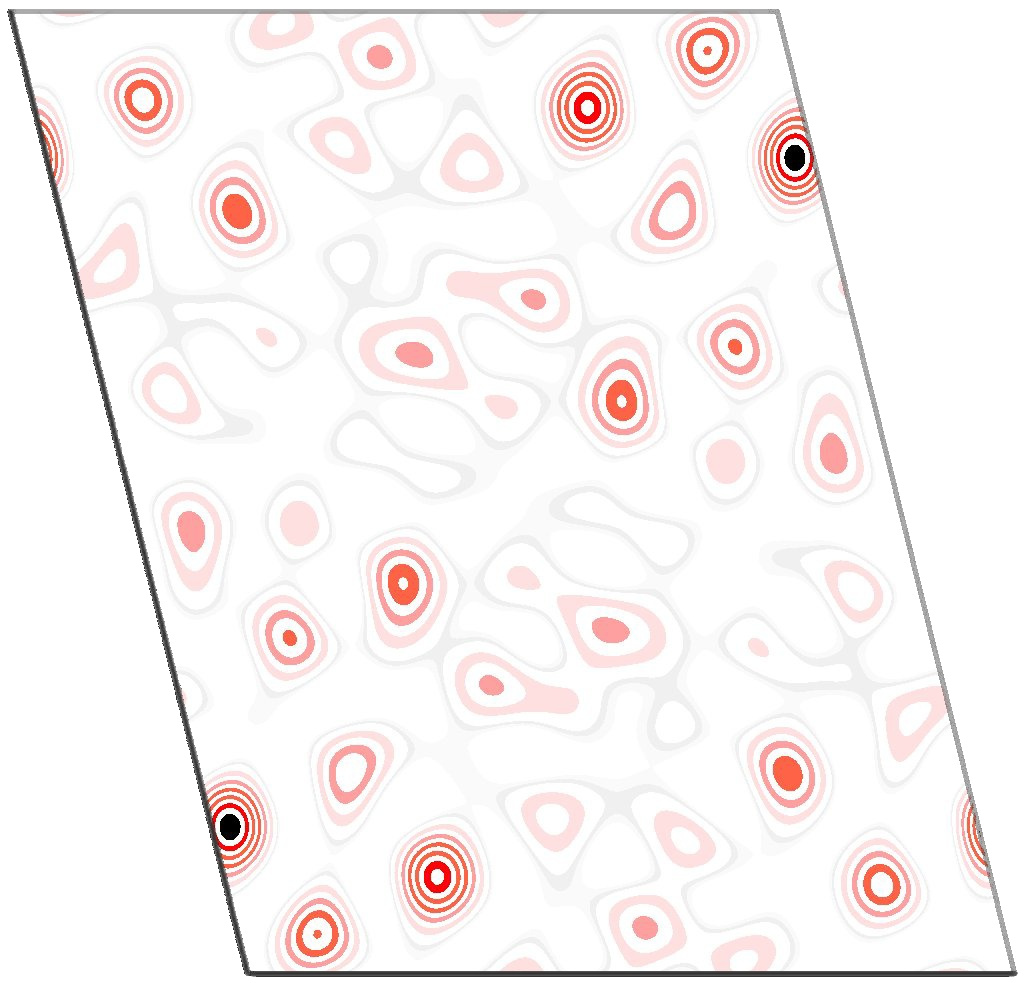

Supplement: Supplementary file 7 [file e-82-00534-sup8.zip › oi2035_SupportingMaterial/Example3/Example3 FOU Maps/Frame_S13 A=180 C=0 D=180 S=180 EFOU Fourier-Map_N48_32bit_gray_1024pix_LUT3.jpg]

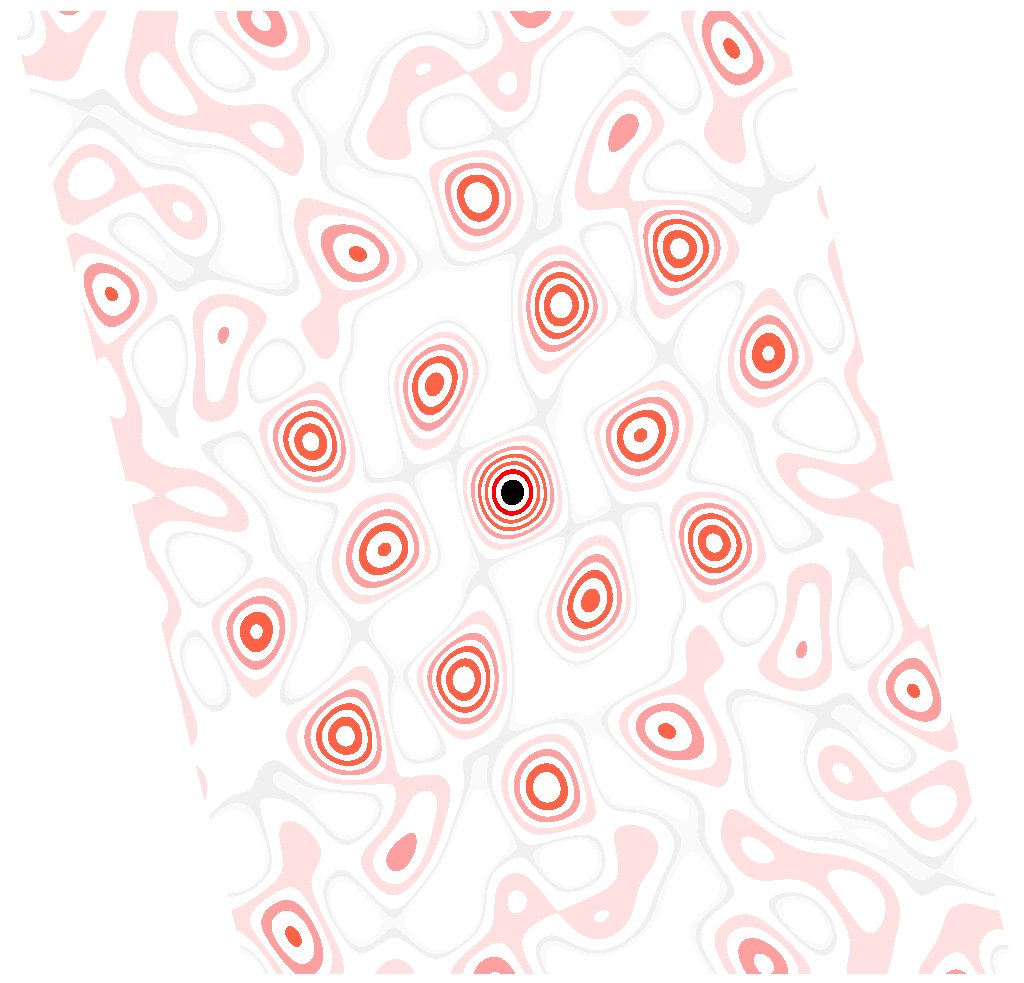

Supplement: Supplementary file 7 [file e-82-00534-sup8.zip › oi2035_SupportingMaterial/Example3/Example3 FOU Maps/S1 A=0 C=0 D=0 S=0 EFOU Fourier-Map_N48_32bit_gray_1024pix_LUT3.jpg]

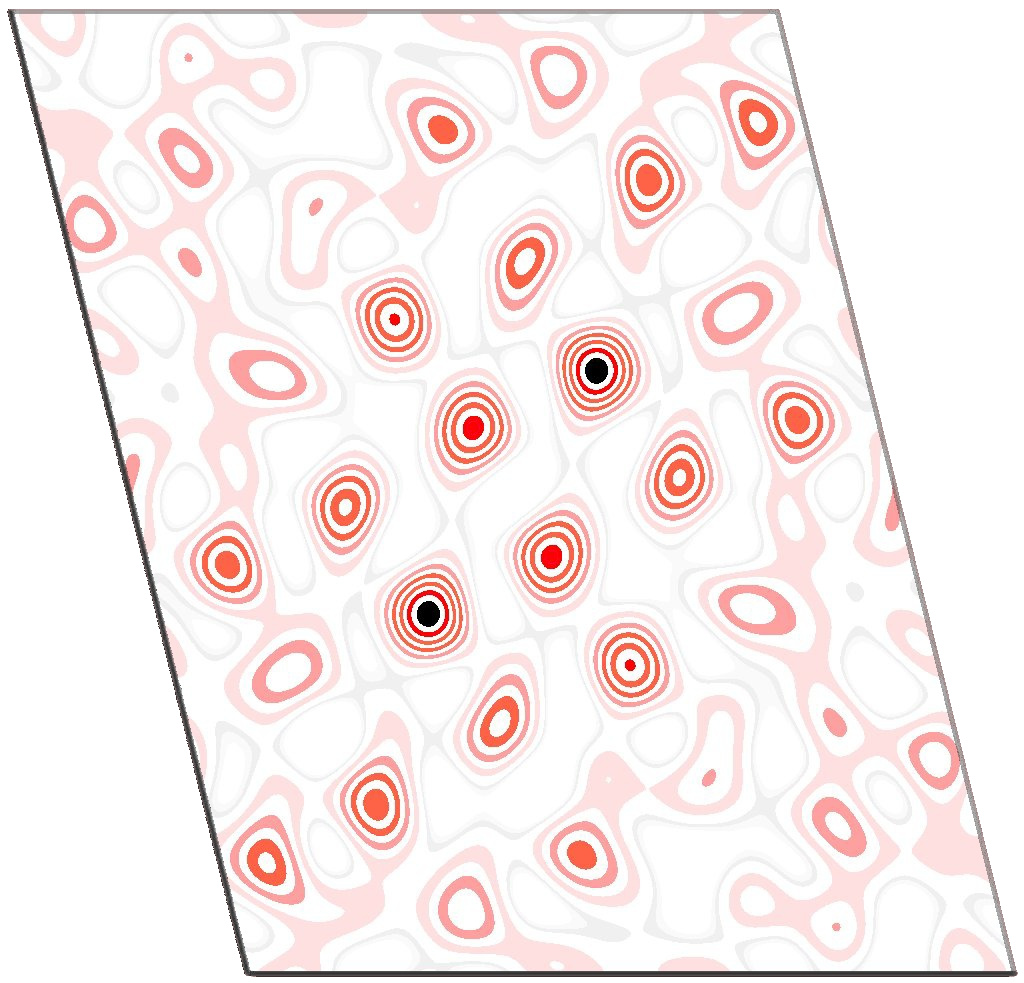

Supplement: Supplementary file 7 [file e-82-00534-sup8.zip › oi2035_SupportingMaterial/Example3/Example3 FOU Maps/Frame_S15 A=180 C=180 D=180 S=0 EFOU Fourier-Map_N48_32bit_gray_1024pix_LUT3.jpg]

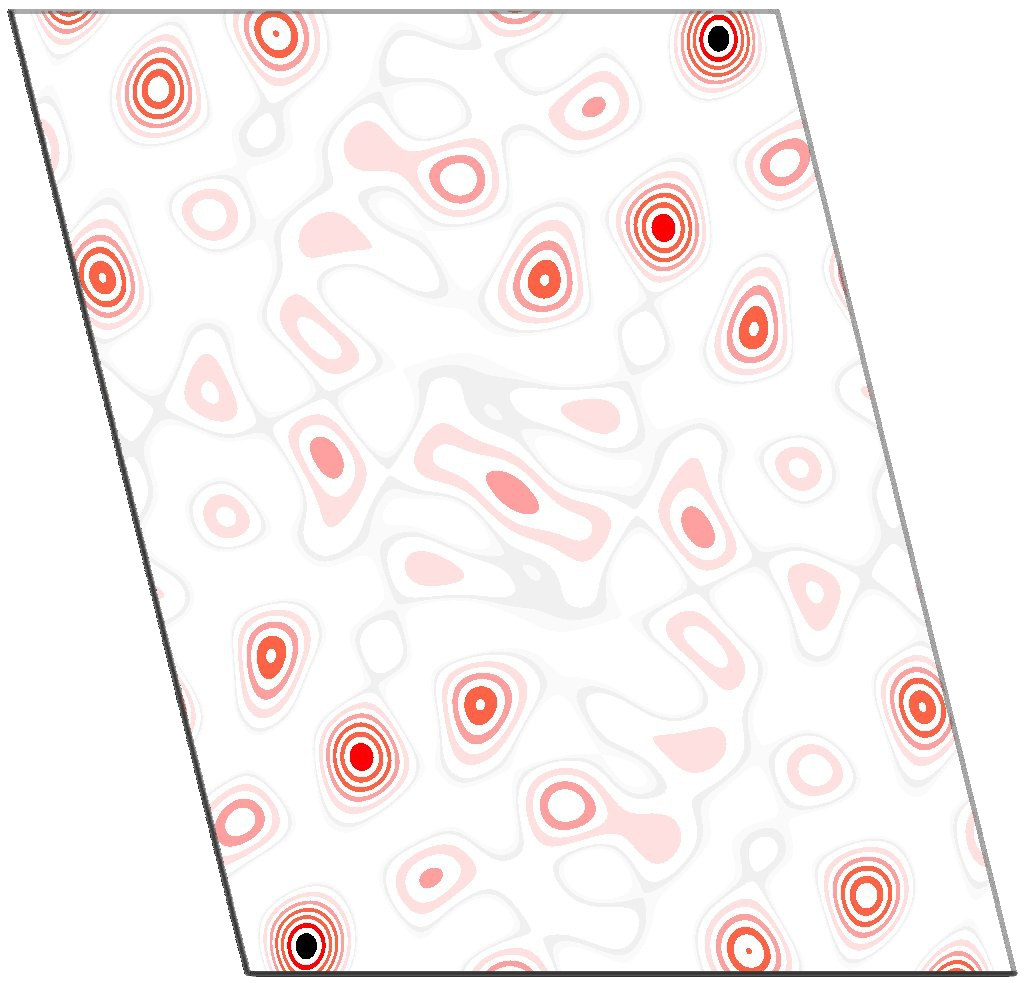

Supplement: Supplementary file 7 [file e-82-00534-sup8.zip › oi2035_SupportingMaterial/Example3/Example3 FOU Maps/Frame_S5 A=180 C=0 D=0 S=0 EFOU Fourier-Map_N48_32bit_gray_1024pix_LUT3.jpg]

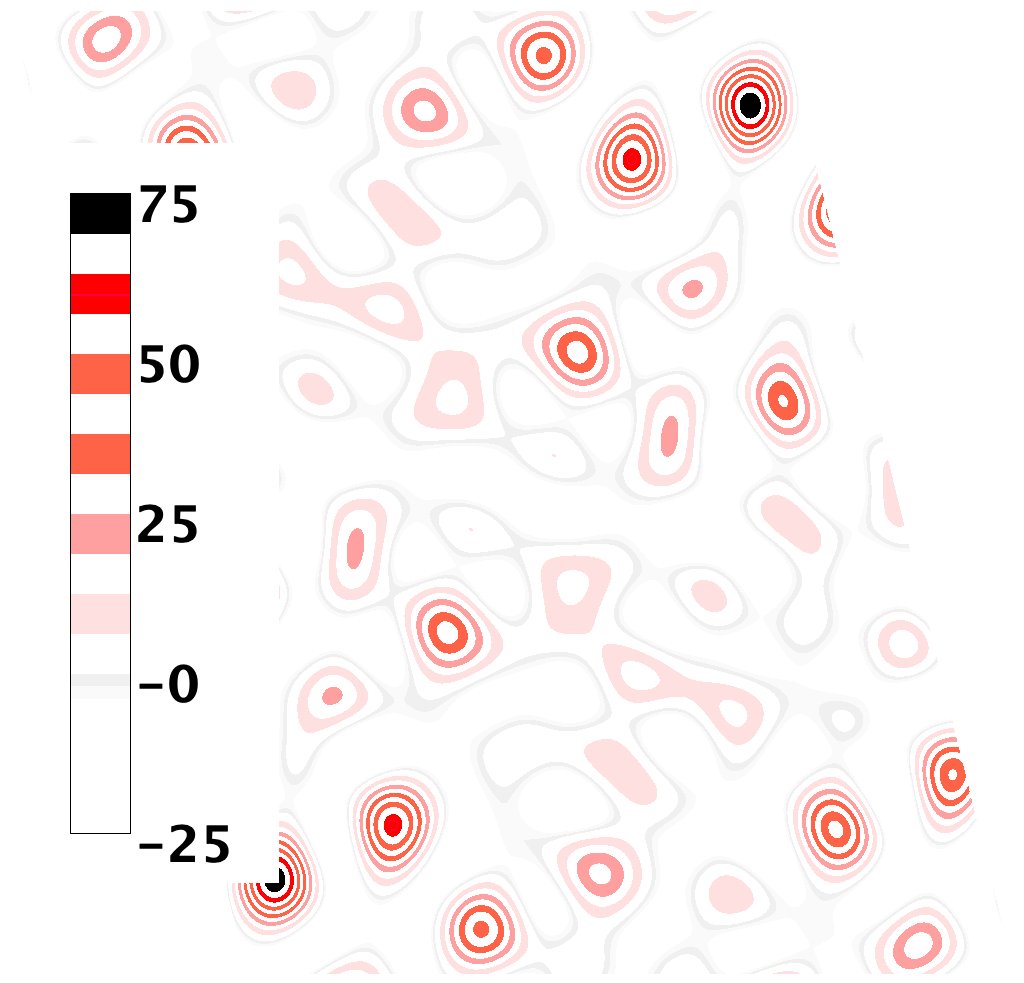

Supplement: Supplementary file 7 [file e-82-00534-sup8.zip › oi2035_SupportingMaterial/Example3/Example3 FOU Maps/S11 A=0 C=180 D=180 S=0 EFOU Fourier-Map_N48_32bit_gray_1024pix_ramp.jpg]

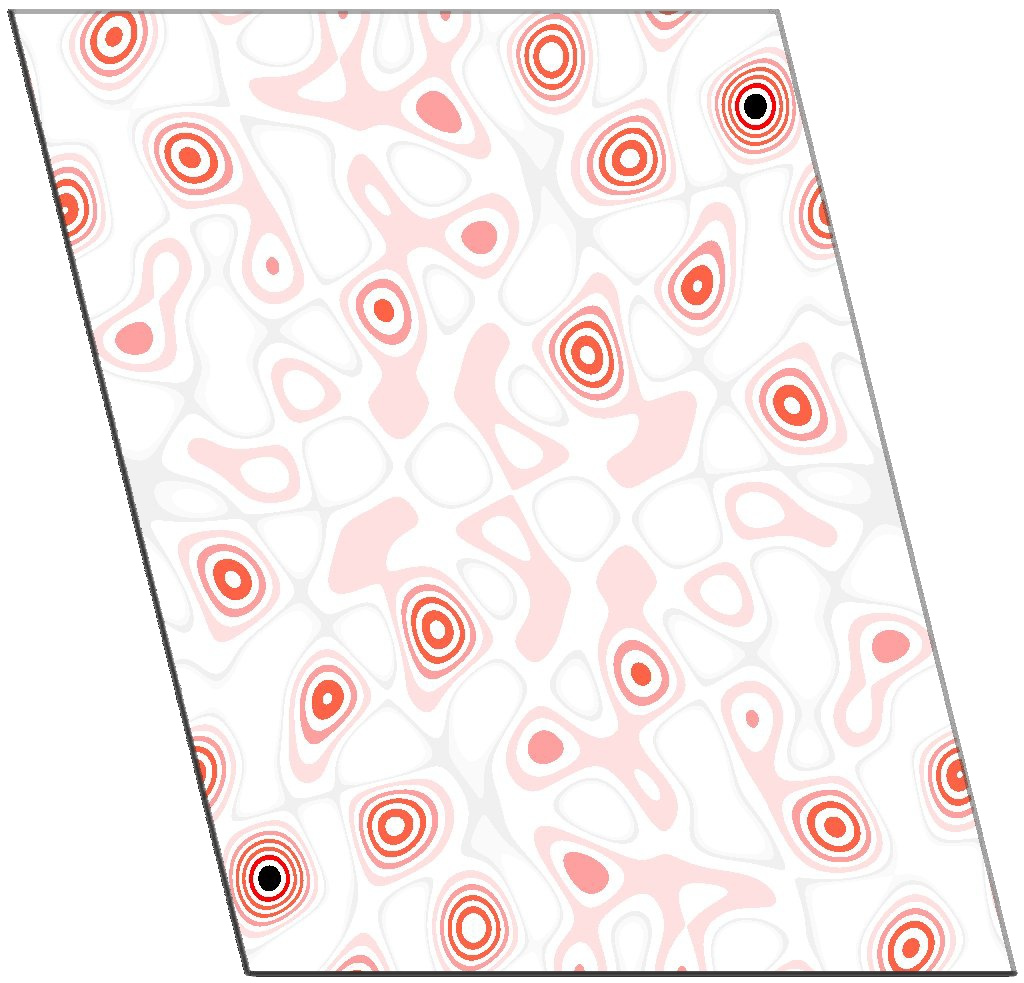

Supplement: Supplementary file 7 [file e-82-00534-sup8.zip › oi2035_SupportingMaterial/Example3/Example3 FOU Maps/Frame_S12 A=0 C=180 D=180 S=180 EFOU Fourier-Map_N48_32bit_gray_1024pix_LUT3.jpg]

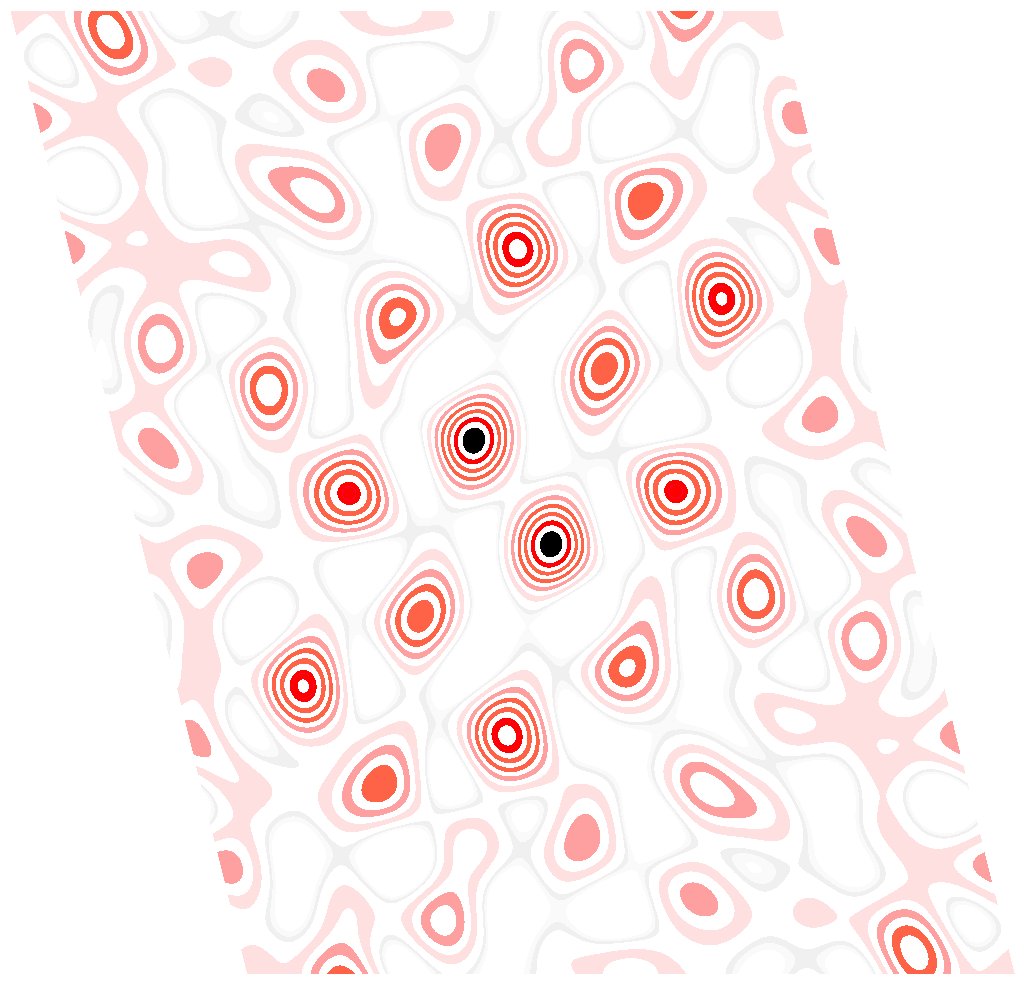

Supplement: Supplementary file 7 [file e-82-00534-sup8.zip › oi2035_SupportingMaterial/Example3/Example3 FOU Maps/S14 A=180 C=180 D=0 S=180 EFOU Fourier-Map_N48_32bit_gray_1024pix_LUT3.jpg]

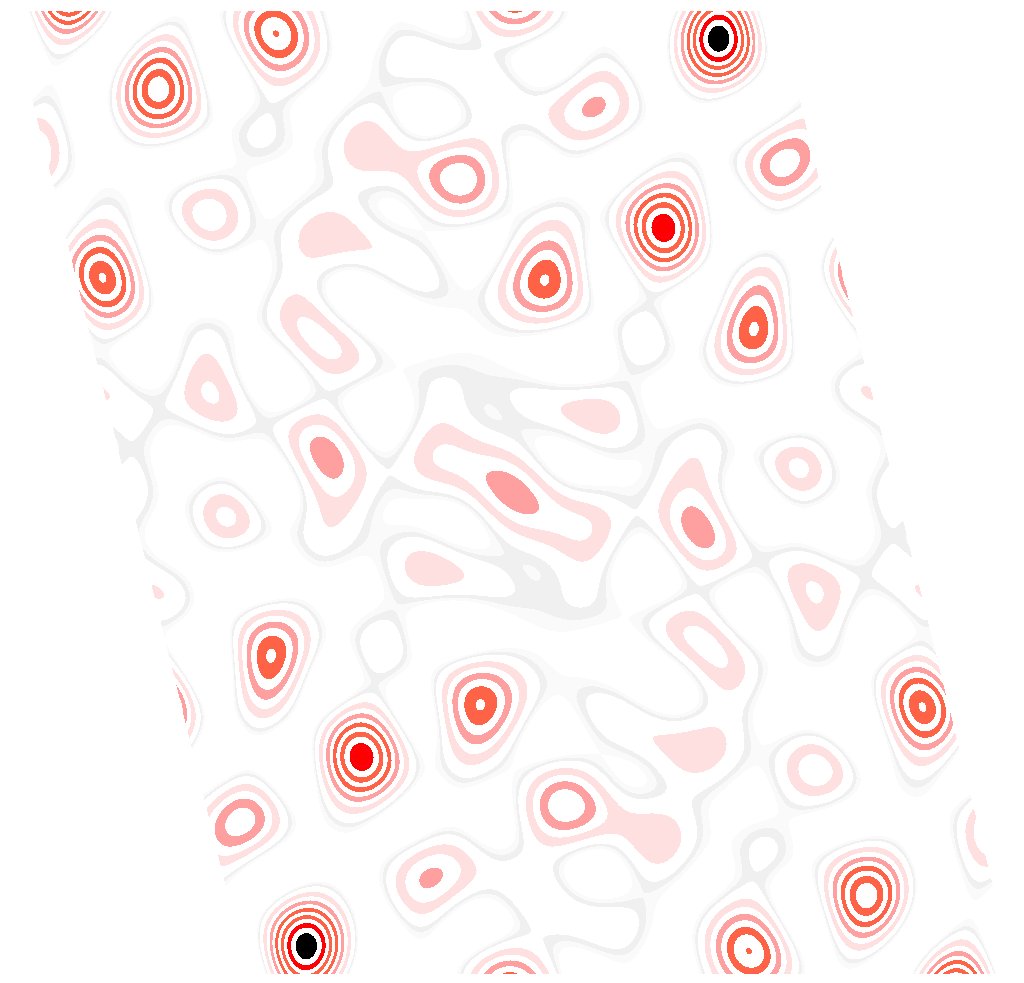

Supplement: Supplementary file 7 [file e-82-00534-sup8.zip › oi2035_SupportingMaterial/Example3/Example3 FOU Maps/S5 A=180 C=0 D=0 S=0 EFOU Fourier-Map_N48_32bit_gray_1024pix_LUT3.jpg]
