# Supplementary material for: Revisiting symbolic addition: a step-by-step introduction to manual direct methods
Source: Acta Crystallogr E Crystallogr Commun. 2026 Apr 10;82(Pt 5):534–43. doi: 10.1107/S2056989026003300 (PMC13148211; doi:10.1107/S2056989026003300)
Supplement: Supplementary file 7 [file e-82-00534-sup8.zip › oi2035_SupportingMaterial/Example2/AppendixB.pdf]

Appendix B.

Table B1 Σ_2 list of normalised structure factors *E(hk)* for the hypothetical structure of example 2 ordered by hk parity. Initially unknown phase values α(hk) are replaced by letters (Note that the letter “I” has been skipped to avoid misinterpretation). The even-odd and odd-even normalised structure factors marked by a star (*) are used for fixing the origin at x=0 and y=0 (see Table 2).

**Frequency in triplets**	***h***	***k***	***E(hk)***	***α(hk)***
h even, k even				
2	0	-4	1.97	*A*
2	0	4	1.97	*A*
2	-2	-2	1.24	*B*
2	2	2	1.24	*B*
3	-2	2	1.01	*C*
3	2	-2	1.01	*C*
h even, k odd				
2	-2	1	1.51	*D*
2	2	-1	1.51	*D*
3	-2	-3	1.46	*E**
3	2	3	1.46	*E**
1	-2	-1	1.22	*F*
1	2	1	1.22	*F*
3	4	1	1.08	*G*
3	-4	-1	1.08	*G*
h odd, k even				
2	-1	-4	1.34	*H**
2	1	4	1.34	*H**
1	3	0	1.27	*J*
1	-3	0	1.27	*J*
h odd, k odd				
2	-3	3	1.27	*K*
2	3	-3	1.27	*K*
3	-1	1	1.08	*L*
3	1	-1	1.08	*L*

Table B2 Reduced list of triplets in algebraic form derived from Table B.1. The characters labelled with a star (*) refer to the origin fixing structure factors with $E^* = 0°$ and $H^* = 180$ °

$E^* + A + D = 0$

$E^* + H^* + L = 0$

$G + H^* + K = 0$

$G + C + E^* = 0$

$J + L + F = 0$

$G + D + B = 0$

$K + L + C = 0$

$B + A + C = 0$

Table B3 Permutation of phases for resolving the ambiguity of the unknown phases *A*, *B* and *F* for the structure in Example 2.

Trial	α_A	α_B	α_F
S1	0°	0°	0°
S2	180°	0°	0°
S3	0°	180°	0°
S4	0°	0°	180°
S5	0°	180°	180°
S6	180°	0°	180°
S7	180°	180°	0°
S8	180°	180°	180°

Table B4 The eight different sets of phases that are needed for resolving the ambiguity of the unknown phases *A, B*, and *F* for the structure in example 2. Each trial set of phases S1 to S8 was used to calculate a Fourier map representing a potential solution (see Figure B1). The last column contains the phase values obtained by calculations based on the model in Figure 4. Comparison of the phase values shows that the correct solution corresponds to phase set S6.

h	***k***		**S1**<br>*A*=0<br>*B*=0<br>*F*=0	**S2**<br>*A*=180<br>*B*=0<br>*F*=0	**S3**<br>*A*=0<br>*B*=180<br>*F*=0	**S4**<br>*A*=0<br>*B*=0<br>*F*=180	**S5**<br>*A*=0<br>*B*=180<br>*F*=180	**S6**<br>*A*=180<br>*B*=0<br>*F*=180	**S7**<br>*A*=180<br>*B*=180<br>*F*=0	**S8**<br>*A*=180<br>*B*=180<br>*F*=180	**calculated from model**
0	-4	A	0	180	0	0	0	180	180	180	180
0	4	A	0	180	0	0	0	180	180	180	180
-2	-2	B	0	0	180	0	180	0	180	180	0
2	2	B	0	0	180	0	180	0	180	180	0
-2	2	-A -B	0	180	180	0	180	180	0	0	180
2	-2	-A -B	0	180	180	0	180	180	0	0	180
-2	1	A	0	180	0	0	0	180	180	180	180
2	-1	A	0	180	0	0	0	180	180	180	180
-2	-3	0	0	0	0	0	0	0	0	0	0
2	3	0	0	0	0	0	0	0	0	0	0
-2	-1	F	0	0	0	180	180	180	0	180	180
2	1	F	0	0	0	180	180	180	0	180	180
4	1	-A -B	0	180	180	0	180	180	0	0	180
-4	-1	-A -B	0	180	180	0	180	180	0	0	180
-1	-4	180	180	180	180	180	180	180	180	180	180
1	4	180	180	180	180	180	180	180	180	180	180
3	0	180 - F	180	180	180	0	0	0	180	0	0
-3	0	180 - F	180	180	180	0	0	0	180	0	0
-3	3	A+B+180	180	0	0	180	0	0	180	180	0
3	-3	A+B+180	180	0	0	180	0	0	180	180	0
-1	1	180	180	180	180	180	180	180	180	180	180
1	-1	180	180	180	180	180	180	180	180	180	180

Appendix B

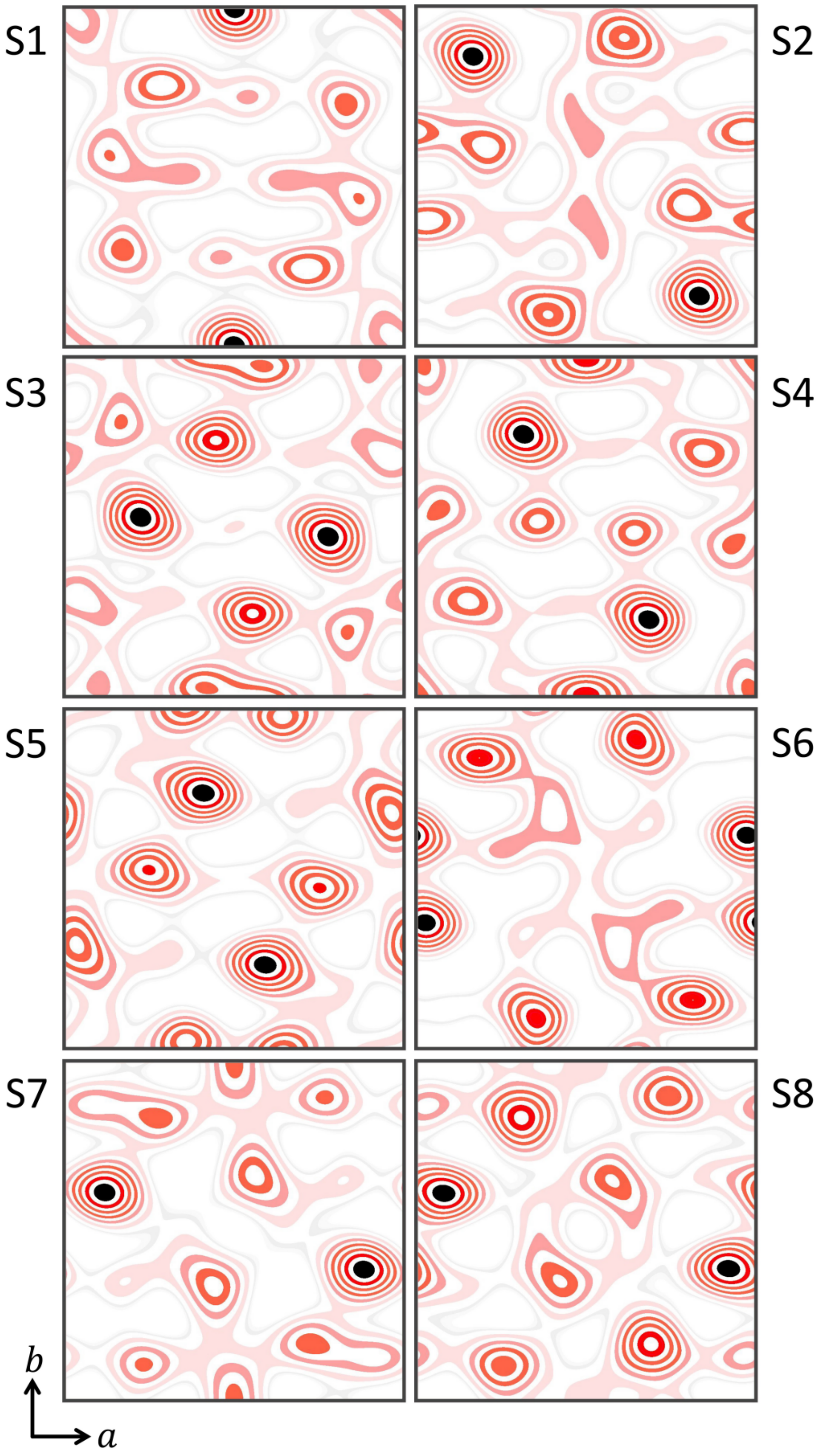

Figure B1 Fourier maps of the eight potential solutions for the two-dimensional model structure of example 2. The maps were calculated with the E(hk) values and phases listed in table B3. Comparison with the structural model in Figure 4 shows that Fourier map S6 corresponds to the correct solution.

Table B5 Positional accuracy of the atom peaks in Fourier map S6 (see Figure B1), obtained from 22 normalised structure factors, compared to the underlying model in Figure 4.

Model Example 1		Fourier map S4		Difference Model – S4		Difference in Å
x	y	x	y	Δx	Δy	
0.04429	0.38934	0.02456	0.37141	0.01973	0.01793	0.082
0.33778	0.08633	0.35031	0.08787	-0.01253	-0.00154	0.039
0.75582	0.14106	0.81454	0.14326	-0.05872	-0.00220	0.182
0.95571	0.61066	0.97412	0.62990	-0.01841	-0.01924	0.082
0.66222	0.91367	0.64573	0.91213	0.01649	0.00154	0.051
0.24418	0.85894	0.18414	0.85674	0.06004	0.00220	0.186
