# Supplementary figures and images for: Revisiting symbolic addition: a step-by-step introduction to manual direct methods (part 2 of 2)
Source: Acta Crystallogr E Crystallogr Commun. 2026 Apr 10;82(Pt 5):534–43. doi: 10.1107/S2056989026003300 (PMC13148211; doi:10.1107/S2056989026003300)

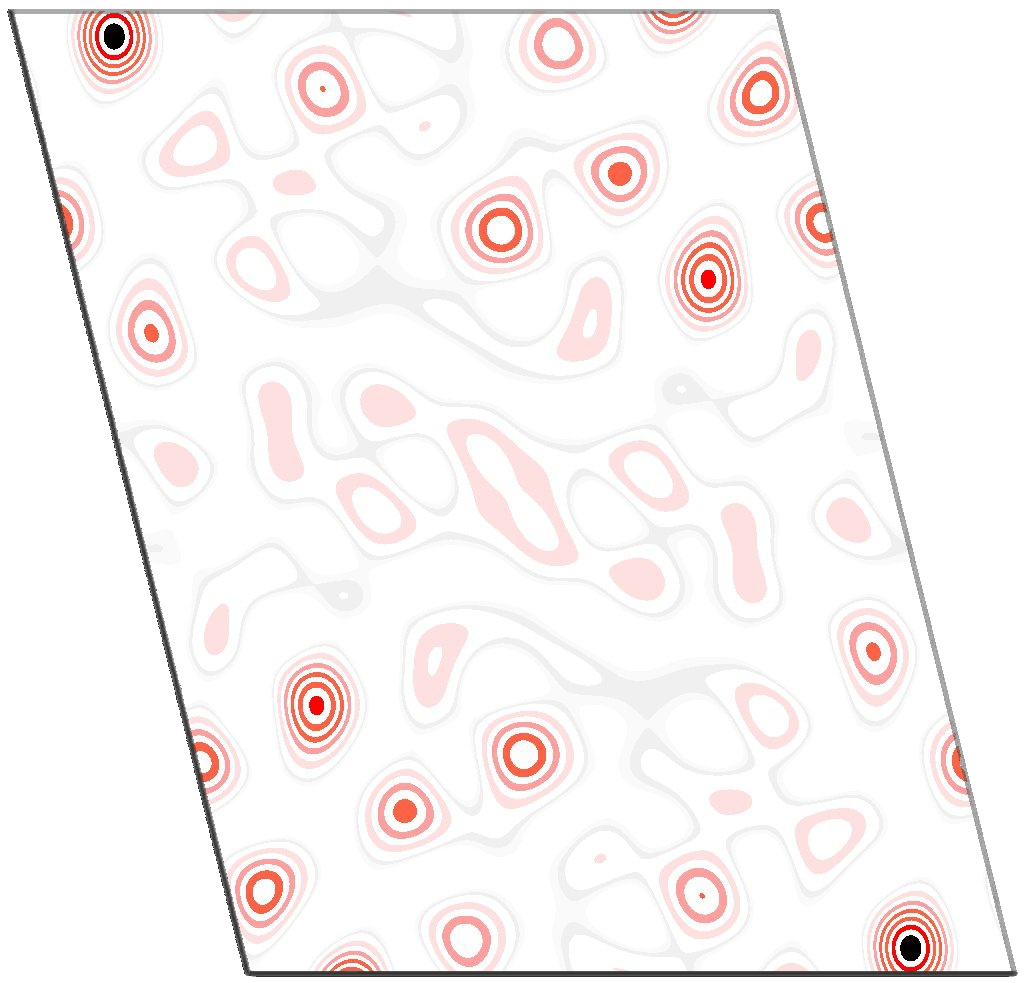

Supplement: Supplementary file 7 [file e-82-00534-sup8.zip › oi2035_SupportingMaterial/Example3/Example3 FOU Maps/Frame_S9 A=0 C=180 D=0 S=180 EFOU Fourier-Map_N48_32bit_gray_1024pix_LUT3.jpg]

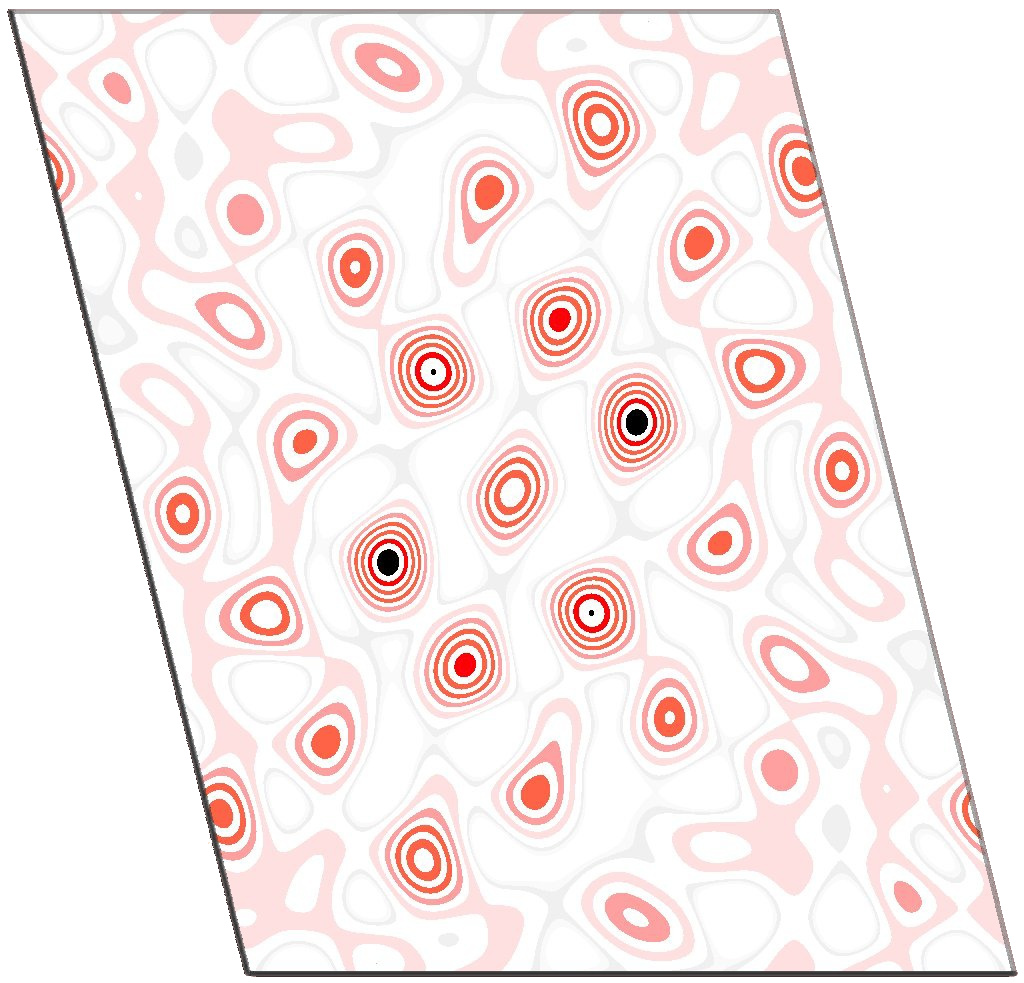

Supplement: Supplementary file 7 [file e-82-00534-sup8.zip › oi2035_SupportingMaterial/Example3/Example3 FOU Maps/Frame_S6 A=0 C=0 D=180 S=180 EFOU Fourier-Map_N48_32bit_gray_1024pix_LUT3.jpg]

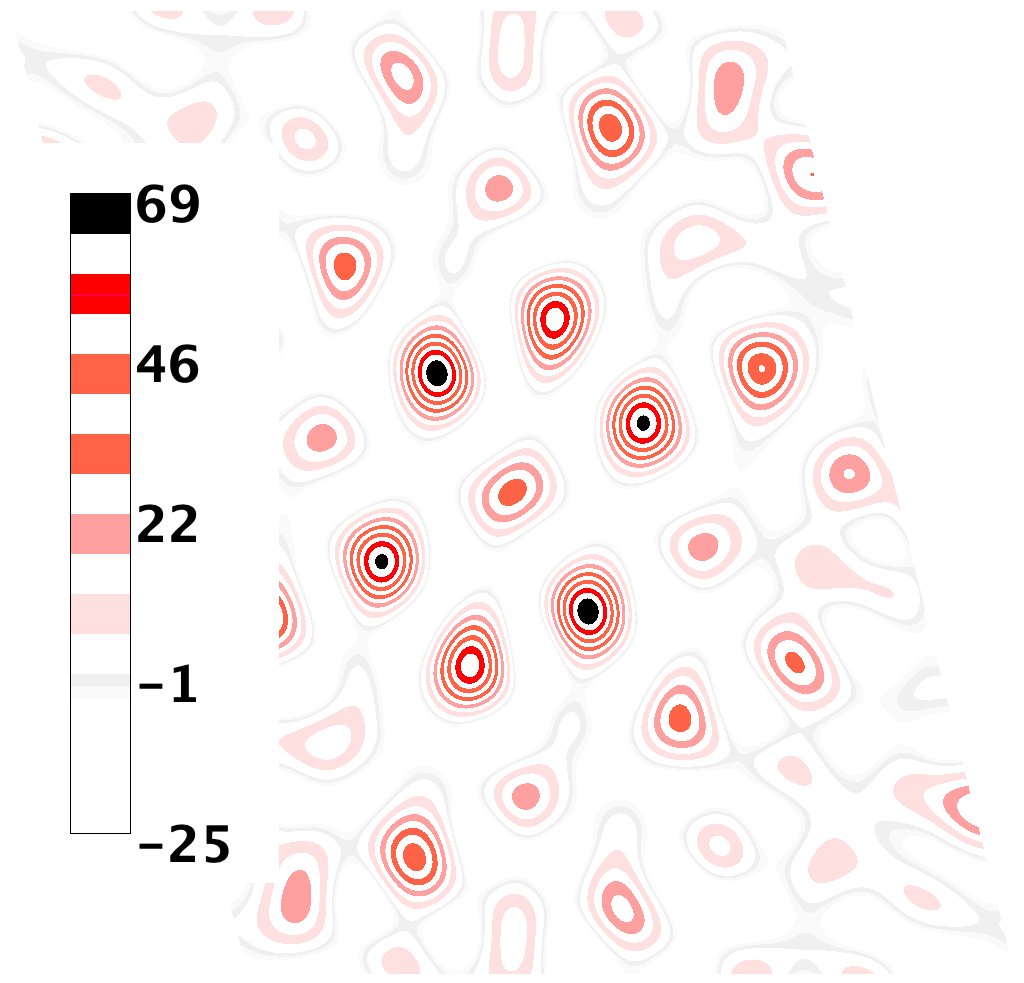

Supplement: Supplementary file 7 [file e-82-00534-sup8.zip › oi2035_SupportingMaterial/Example3/Example3 FOU Maps/S3 A=0 C=0 D=180 S=0 EFOU Fourier-Map_N48_32bit_gray_1024pix_ramp.jpg]

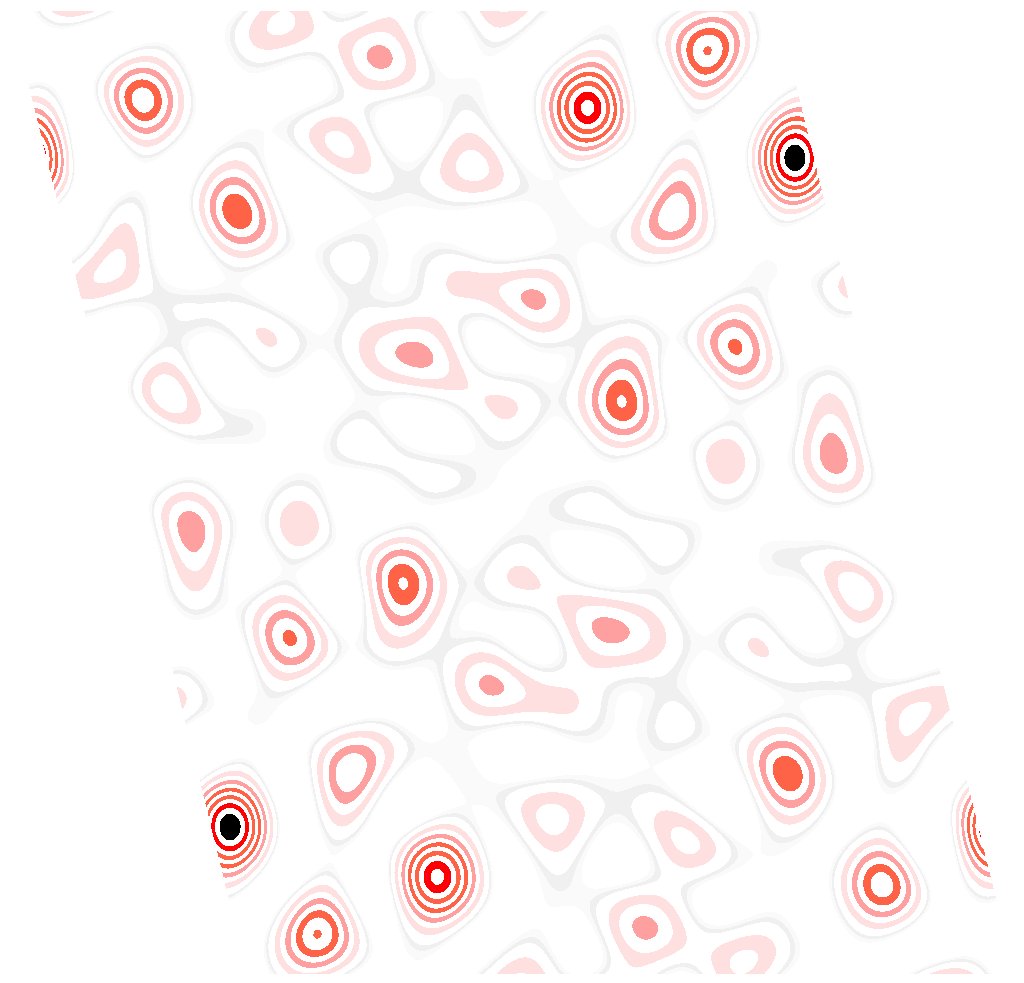

Supplement: Supplementary file 7 [file e-82-00534-sup8.zip › oi2035_SupportingMaterial/Example3/Example3 FOU Maps/S13 A=180 C=0 D=180 S=180 EFOU Fourier-Map_N48_32bit_gray_1024pix_LUT3.jpg]

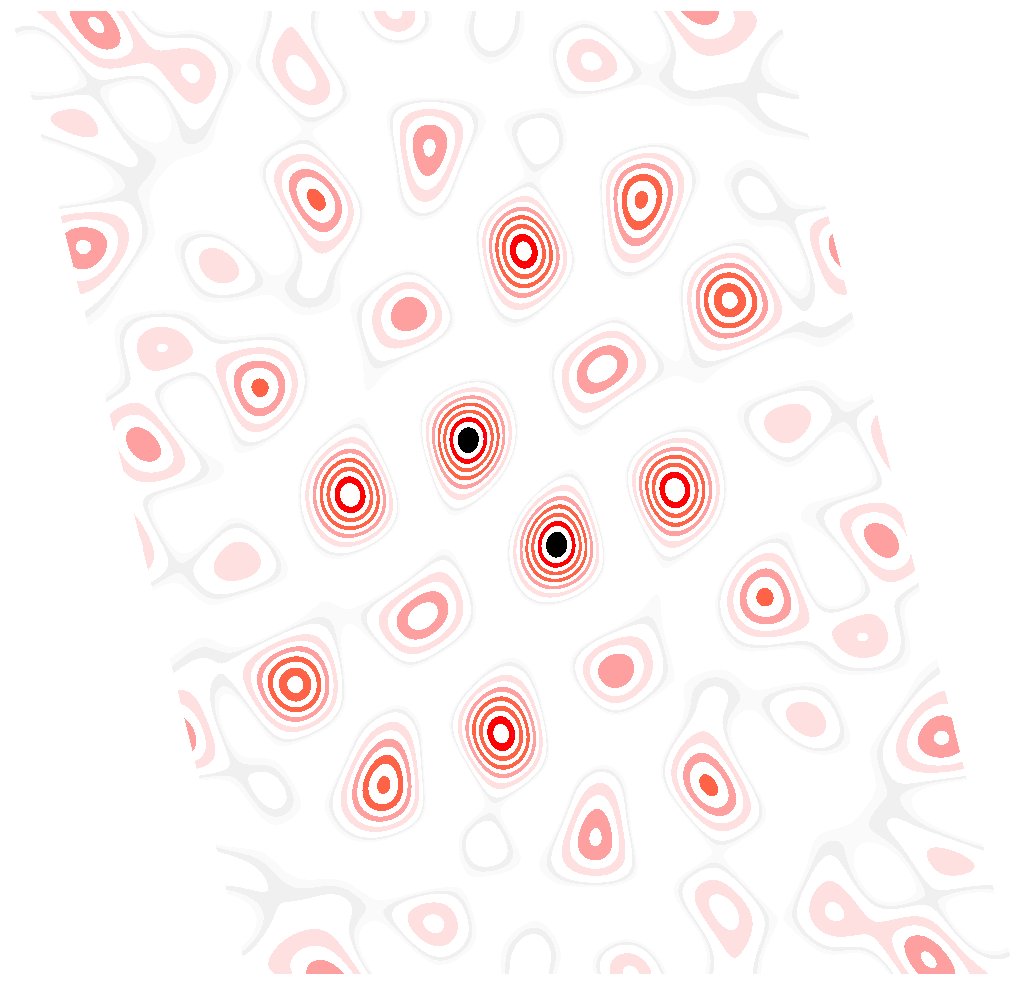

Supplement: Supplementary file 7 [file e-82-00534-sup8.zip › oi2035_SupportingMaterial/Example3/Example3 FOU Maps/S7 A=180 C=180 D=0 S=0 EFOU Fourier-Map_N48_32bit_gray_1024pix_LUT3.jpg]

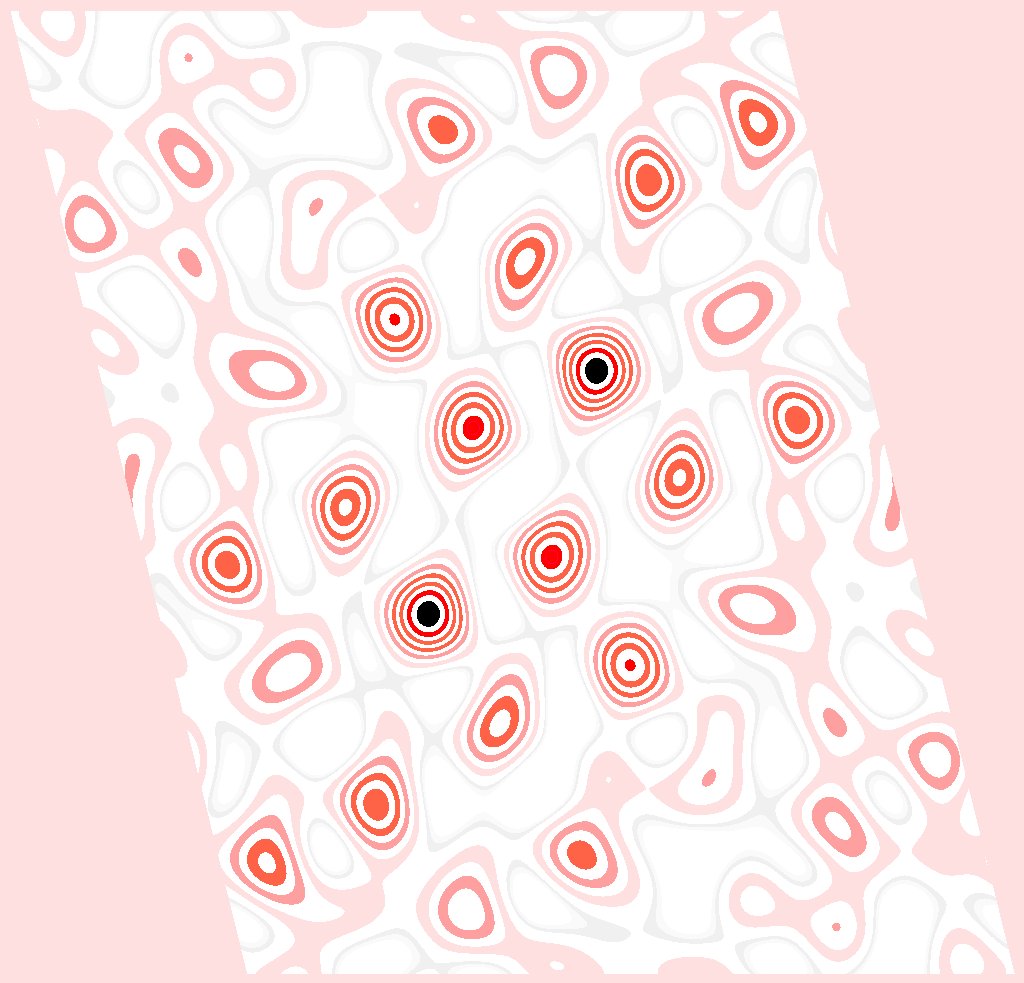

Supplement: Supplementary file 7 [file e-82-00534-sup8.zip › oi2035_SupportingMaterial/Example3/Example3 FOU Maps/S15 A=180 C=180 D=180 S=0 EFOU Fourier-Map_N48_32bit_gray_1024pix_LUT3.jpg]

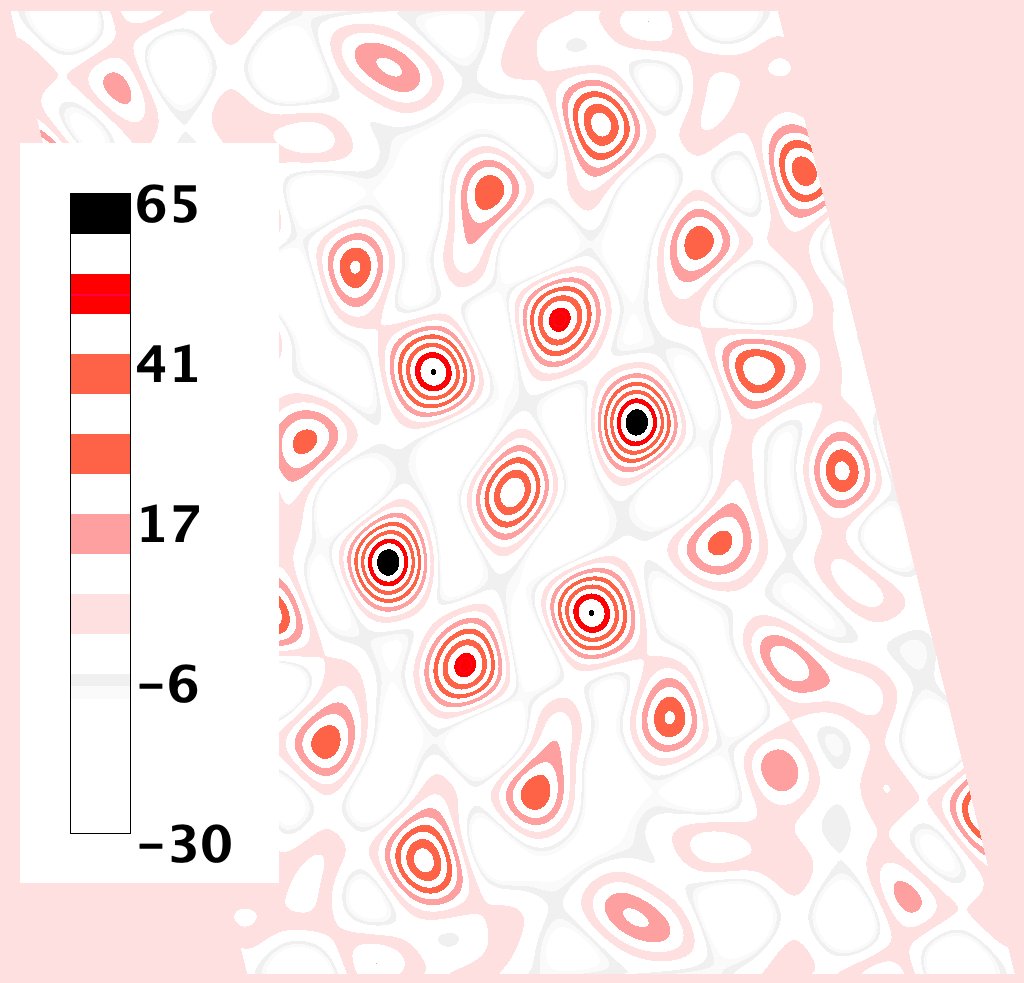

Supplement: Supplementary file 7 [file e-82-00534-sup8.zip › oi2035_SupportingMaterial/Example3/Example3 FOU Maps/S6 A=0 C=0 D=180 S=180 EFOU Fourier-Map_N48_32bit_gray_1024pix_ramp.jpg]

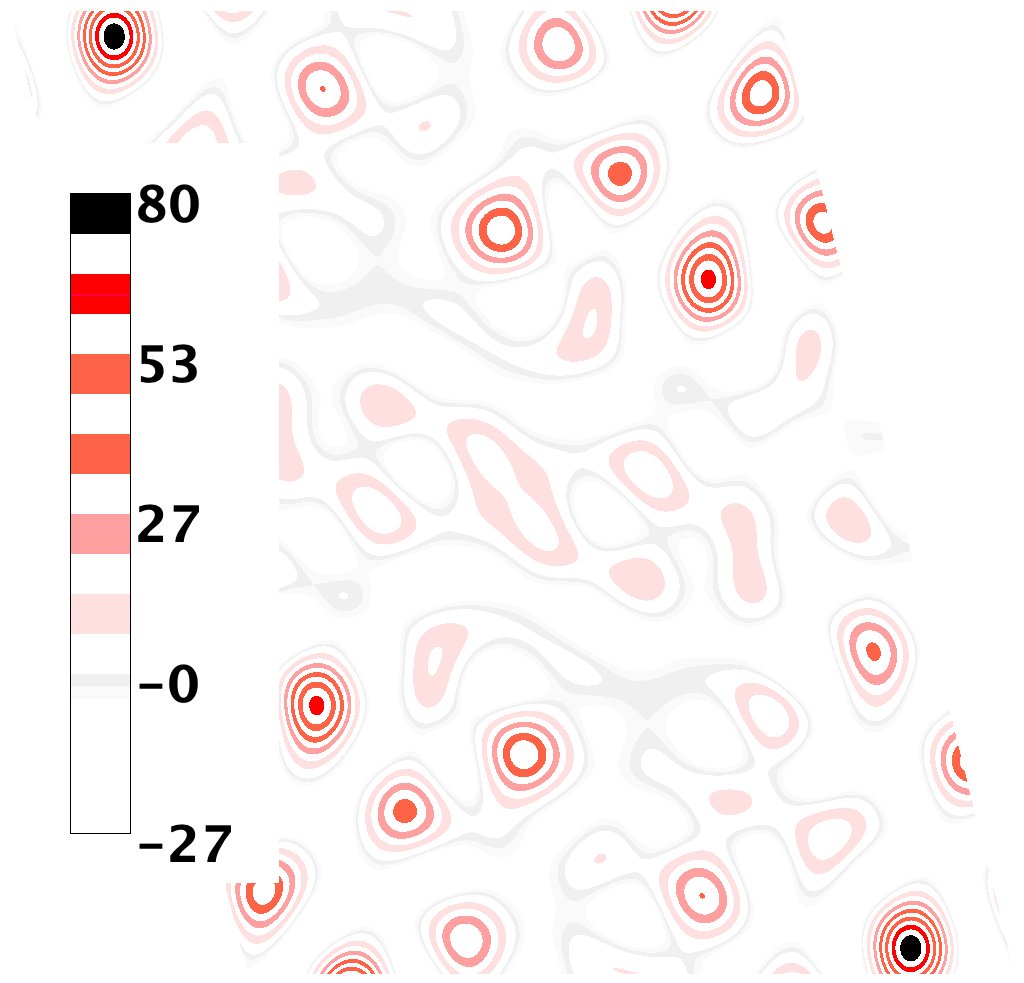

Supplement: Supplementary file 7 [file e-82-00534-sup8.zip › oi2035_SupportingMaterial/Example3/Example3 FOU Maps/S9 A=0 C=180 D=0 S=180 EFOU Fourier-Map_N48_32bit_gray_1024pix_ramp.jpg]
